# Phenanthroline-imine ligands for iron-catalyzed alkene hydrosilylation†

**DOI:** 10.1039/d1sc06727c

**Published:** 2022-02-10

**Authors:** Wei Sun, Ming-Peng Li, Lu-Jie Li, Qiang Huang, Meng-Yang Hu, Shou-Fei Zhu

**Affiliations:** Frontiers Science Center for New Organic Matter, State Key Laboratory and Institute of Elemento-Organic Chemistry, College of Chemistry, Nankai University Tianjin 300071 China sfzhu@nankai.edu.cn; Haihe Laboratory of Sustainable Chemical Transformations Tianjin 300192 China

## Abstract

Iron-catalyzed organic reactions have been attracting increasing research interest but still have serious limitations on activity, selectivity, functional group tolerance, and stability relative to those of precious metal catalysts. Progress in this area will require two key developments: new ligands that can impart new reactivity to iron catalysts and elucidation of the mechanisms of iron catalysis. Herein, we report the development of novel 2-imino-9-aryl-1,10-phenanthrolinyl iron complexes that catalyze both *anti*-Markovnikov hydrosilylation of terminal alkenes and 1,2-*anti*-Markovnikov hydrosilylation of various conjugated dienes. Specifically, we achieved the first examples of highly 1,2-*anti*-Markovnikov hydrosilylation reactions of aryl-substituted 1,3-dienes and 1,1-dialkyl 1,3-dienes with these newly developed iron catalysts. Mechanistic studies suggest that the reactions may involve an Fe(0)–Fe(ii) catalytic cycle and that the extremely crowded environment around the iron center hinders chelating coordination between the diene and the iron atom, thus driving migration of the hydride from the silane to the less-hindered, terminal end of the conjugated diene and ultimately leading to the observed 1,2-*anti*-Markovnikov selectivity. Our findings, which have expanded the types of iron catalysts available for hydrosilylation reactions and deepened our understanding of the mechanism of iron catalysis, may inspire the development of new iron catalysts and iron-catalyzed reactions.

## Abstract 2

Newly developed iron complexes bearing 2-imino-9-aryl-1,10-phenanthroline ligands were successfully used to catalyze hydrosilylation of terminal alkenes and conjugated dienes in high yields with excellent *anti*-Markovnikov selectivity.

## Graphical abstract

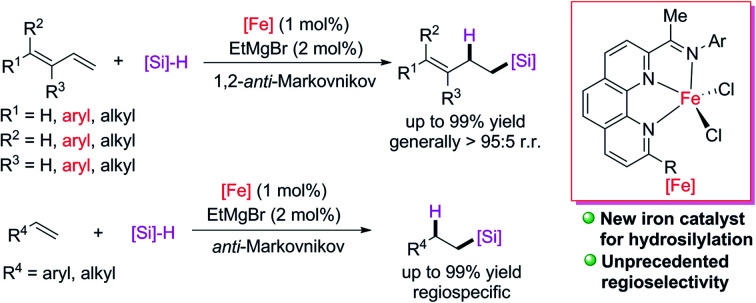

## Introduction

Iron is the most abundant transition metal in the earth's crust, and most of its oxides and salts are inexpensive, readily available, and biocompatible. Iron has various oxidation and spin states that are capable of many chemical transformations and can form complexes with most organic ligands. These characteristics give iron great potential as a catalyst, and thus iron catalysts and iron-catalyzed organic reactions have been attracting increasing research interest. However, despite the important breakthroughs that have been made in this area,^1^ most of the currently available iron catalysts have limited activity, selectivity, functional group tolerance, and stability relative to those of precious metal catalysts. Therefore, there have been only a few industrial applications of iron catalysts in organic synthesis.^2^ Progress in this area will require two key developments: new ligands that can impart new reactivity to iron catalysts and elucidation of the mechanisms of iron catalysis.

Our group is particularly interested in developing iron catalysts for alkene hydrosilylation reactions. Transition-metal-catalyzed alkene hydrosilylation reactions are among the most important homogeneous catalytic reactions, providing an efficient method for the industrial synthesis of organosilicon compounds; and large quantities of platinum catalysts are consumed for this purpose.^3^ However, platinum is an expensive precious metal, and because its abundance in the earth's crust is extremely low, resource depletion is a problem. In addition, platinum is biotoxic, and its large-scale use may lead to environmental pollution. Therefore, the development of methods for iron-catalyzed alkene hydrosilylation reactions, particularly those that cannot be achieved with other metal catalysts, would be of great value, and important progress has been made in this area.^4,5^

**Scheme 1:**
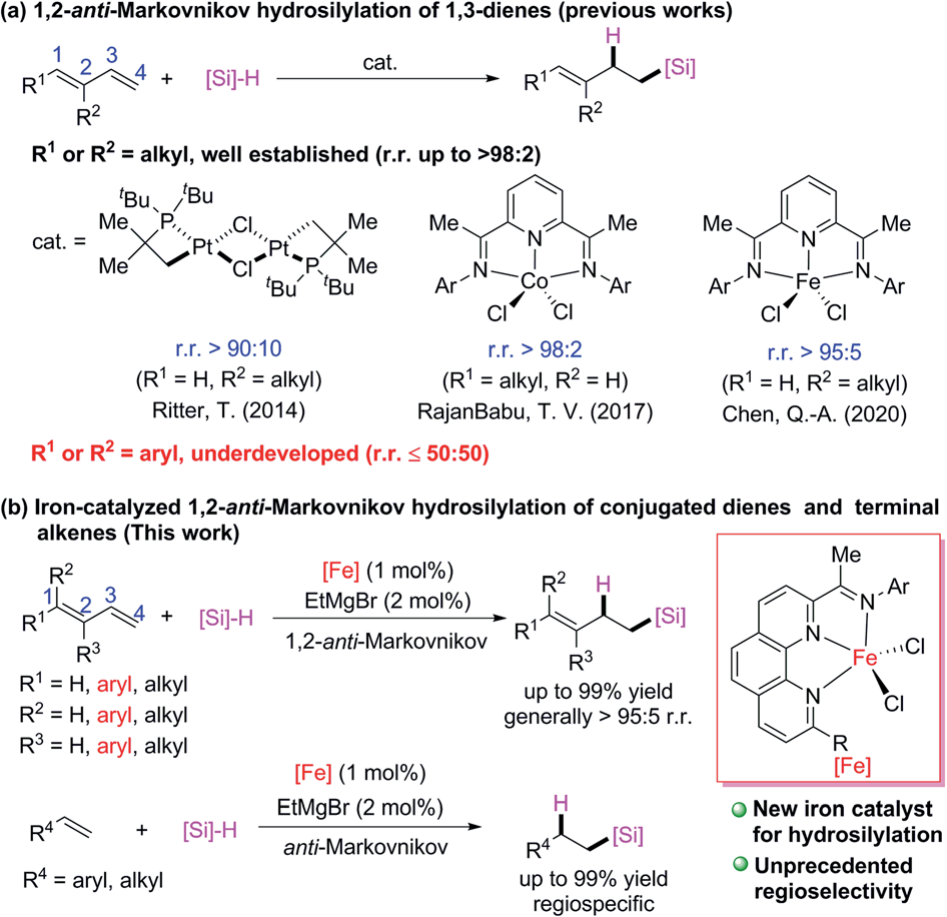
Transition-metal-catalyzed hydrosilylation reactions of conjugated dienes and terminal alkenes. The r.r. = regioisomeric ratio.

Transition-metal-catalyzed hydrosilylation reactions of conjugated dienes have multiple possible coordination and insertion modes because of the conjugated C=C bonds, therefore the regiospecificity and chemoselectivity are difficult to control. Because relatively stable π-allyl metal intermediates readily form in transition-metal-catalyzed hydrosilylation reactions of conjugated dienes, 1,4-addition products are favored over 1,2-addition products. Several transition-metal catalysts have been developed for 1,4-hydrosilylation of conjugated dienes to generate products of addition of a silyl group^6^ or a hydrogen atom^7^ to the terminal carbon (C4) of the conjugated diene. In contrast, relatively few catalytic 1,2-hydrosilylation reactions of conjugated dienes have been achieved. Some progress has recently been made in 1,2-hydrosilylation of C1- and C2-alkyl conjugated dienes (Scheme 1a). For example, Ritter and co-workers^8^ developed a dinuclear platinum catalyst with bulky ligands, which allowed them to realize 1,2-*anti*-Markovnikov hydrosilylation of isoprene; and RajanBabu and coworkers^9^ used a cobalt catalyst with a pyridine-diimine ligand to achieve 1,2-*anti*-Markovnikov hydrosilylation reactions of various C1-alkyl-substitute conjugated dienes. Quite recently, Chen and coworkers^10^ used an iron catalyst bearing a pyridine-diimine ligand to achieve highly selective 1,2-*anti*-Markovnikov hydrosilylation reactions of isoprene and its C2-alkyl derivatives. Highly Markovnikov selective 1,2-hydrosilylation reactions of aryl-substituted conjugated dienes have been accomplished with iron or cobalt complexes bearing bidentate *N*,*N*- or *P*,*P*-ligands.^5*i*,11^ However, selective 1,2-*anti*-Markovnikov addition to such dienes has not yet been achieved (at most 50 : 50 r.r.).^9,12^ Herein, we report that newly developed iron complexes with 2-imino-9-aryl-1,10-phenanthroline ligands catalyzed hydrosilylation reactions of various alkyl- and aryl-substituted conjugated dienes and terminal alkenes with excellent 1,2-*anti*-Markovnikov selectivity (Scheme 1b). In particular, hydrosilylation reactions of aryl-substituted 1,3-dienes and 1,1-dialkyl 1,3-dienes with high 1,2-*anti*-Markovnikov selectivity were realized for the first time to our best knowledge.

## Results and discussion

**Scheme 2:**
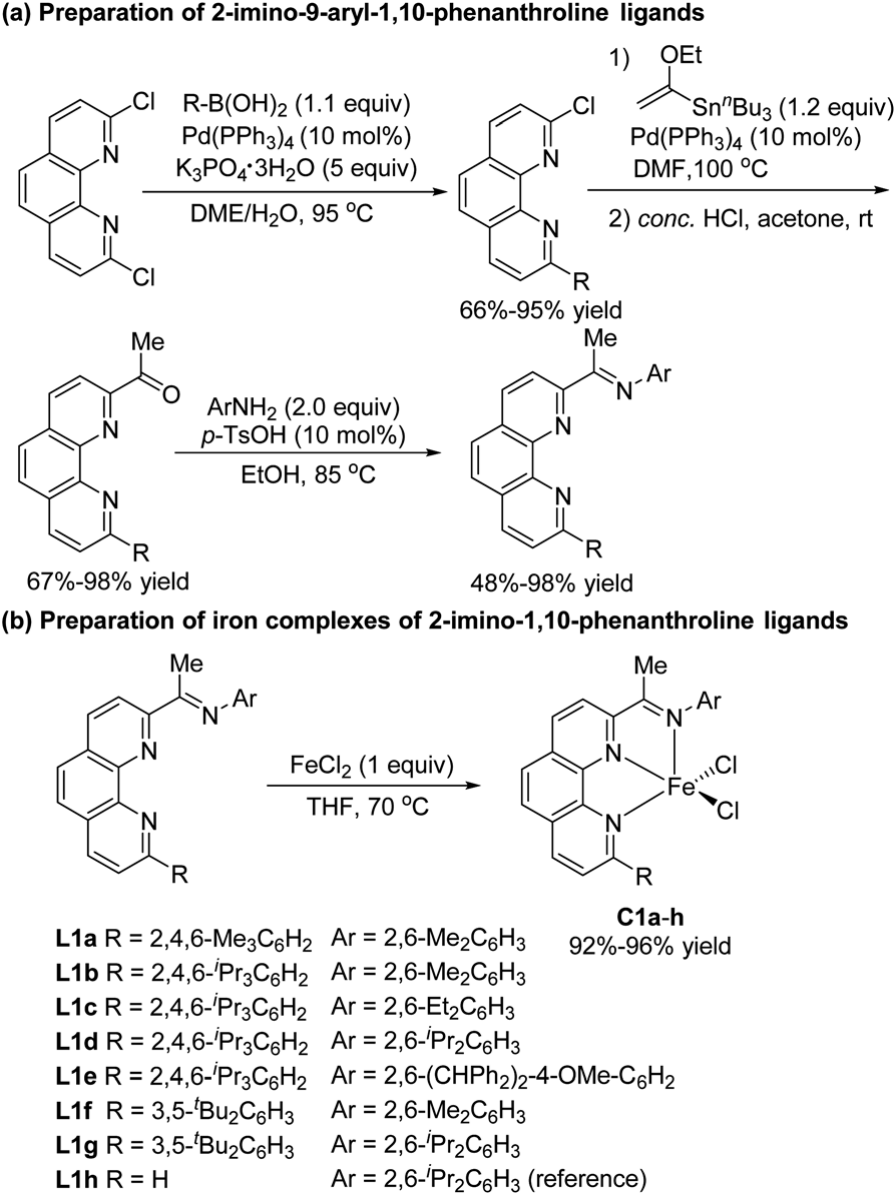
Preparation of 2-imino-1,10-phenanthrolines and their iron complexes.

In recent studies, we showed that iron complexes with 1,10-phenanthroline ligands efficiently catalyze hydrosilylation of alkenes and alkynes, exhibiting unique chemoselectivity or regioselectivity.^5*i*,*k*,13^ We also found that phenanthroline ligands gave good results in other hydride transfer reactions.^14^ In the current study, we carried out hydrosilylation reactions catalyzed by iron complexes of 2-imino-9-aryl-1,10-phenanthrolines, a new class of ligands that were prepared in four simple steps—Suzuki coupling, Stille coupling, hydrolysis, and condensation—from commercially available 2,9-dichloro-1,10-phenanthroline (Scheme 2a). The electronic and steric properties of these ligands could easily be tuned by modification of the *N*-aryl and 9-aryl groups. Iron complexes of the ligands were conveniently prepared by complexation with FeCl_2_ (Scheme 2b). To examine the effect of the 9-aryl group of the ligand on the catalytic properties of the complexes, we also synthesized 2-imino-1,10-phenanthroline iron complex C1h, which has previously been used to catalyze the oligomerization of ethylene.^15^

We used model substrates 1-phenyl-1,3-butadiene (1a) and phenylsilane (2a) to systematically evaluate the catalytic activities of iron complexes bearing 2-imino-9-aryl-1,10-phenanthroline ligands with various substituents (Table 1). Hydrosilylation reactions catalyzed by complexes C1a and C1b proceeded smoothly in 2 h at room temperature in THF with nearly identical yields and regioselectivities (entries 1 and 2). Increasing the steric bulk of the *N*-aryl substituent (C1c and C1d) markedly improved the regioselectivity (to 94 : 6 and 99 : 1, respectively), as well as the yield (to 98% and 99%, respectively) (entries 3 and 4). Further increasing the steric bulk of the *N*-aryl group (C1e) substantially decreased the catalytic activity (as indicated by an increase in the reaction time to 24 h) and slightly decreased the regioselectivity (entry 5). The steric bulk of the R group slightly affected the regioselectivity (compare entry 6 to entry 1). Complex C1g exhibited regioselectivity similar to that obtained with C1d, which indicates that the *N*-aryl group of the ligand was the main determinant of the regioselectivity (compare entry 7 to entry 4). Reference catalyst C1h, which had a ligand without a substituent at the 9-position, showed poor regioselectivity, giving a mixture of 1,2- and 1,4-hydrosilylation products (entry 8), which indicates that the 9-aryl group was necessary for 1,2-hydrosilylation. Additional experiments revealed that adding LiAlH_4_, Et_2_Zn, NaBEt_3_H, lithium diisopropylamide, or KO^*t*^Bu reduced catalyst activity and selectivity; and changing the solvent from THF to ether, toluene, or hexane resulted in poor conversion (Table S1†). The complexes prepared *in situ* from phenanthroline-imine ligand L1d and other metals (Co, Ni, Mn, Cu, and Zn) were totally inactive in this reaction (Tables S2†). This results clearly demonstrated the superiority of iron catalysts in the current study. We also evaluated some other iron complexes (C2–C4, entries 9–11). Complex C2 promoted the hydrosilylation reaction but gave 3aa, 5aa, and 6aa in a 63 : 15 : 21 ratio (entry 9). Complexes with a bisphosphine ligand (C3), or a tripyridine ligand (C4) failed to catalyze the reaction (entries 10 and 11). The above results clearly showed that the 2-imino-9-aryl-1,10-phenanthroline ligand plays critical roles in this reaction.

**Table 1:** Effects of ligands on iron-catalyzed hydrosilylation of conjugated diene 1a with phenylsilane (2a)

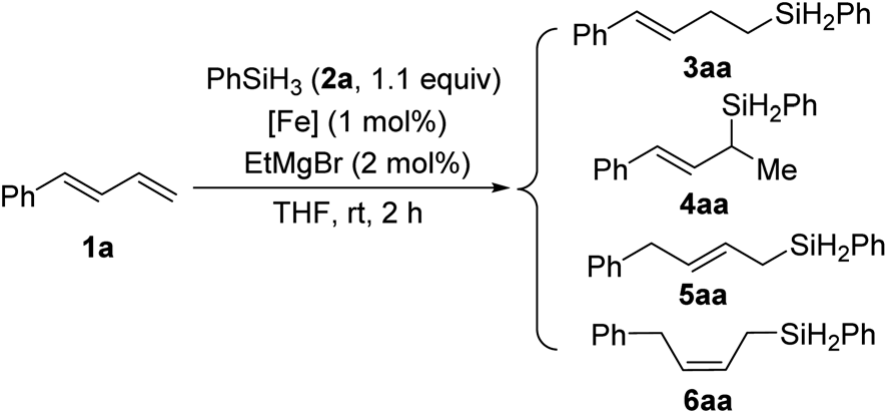				
Entry^a^	[Fe]	Conv. (%)	Yield (%)	3aa/4aa/5aa/6aa
1^b^	C1a	>95	86	84 : 16 : 0 : 0
2	C1b	>95	87	83 : 17 : 0 : 0
3	C1c	>95	98	94 : 6 : 0 : 0
4	C1d	>95	99	99 : 1 : 0 : 0
5^b^	C1e	>95	98	96 : 4 : 0 : 0
6	C1f	>95	94	71 : 29 : 0 : 0
7	C1g	>95	98	98 : 2 : 0 : 0
8	C1h	>95	96	61 : 35 : 4 : 0
9	C2	>95	95	63 : 0 : 15 : 21
10	C3	<5	ND	NA
11	C4	<5	ND	NA
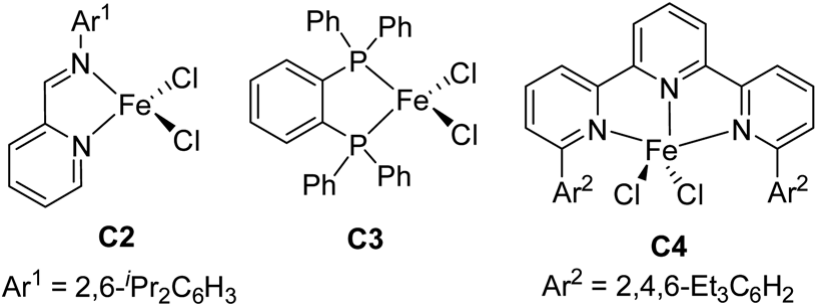				

a Reaction conditions, unless otherwise noted: 1a (0.5 mmol), 2a (0.55 mmol), [Fe] (1 mol%), EtMgBr (2 mol%), THF (1 mL), rt, 2 h. Conversions and product yields and ratios were determined by ^1^H NMR spectroscopy with 1,3,5-trimethoxybenzene as an internal standard. ND, not detected; NA, not applicable.

b Reaction time, 24 h.

Next, we used the optimal conditions (Table 1, entry 4) to evaluate reactions of conjugated diene 1a with various primary silanes 2 (Table 2). Perhaps because of steric hindrance, 2-methylphenylsilane (2b) was less reactive than phenylsilane (2a), and the regioselectivity of the hydrosilylation reaction of 2b was lower than that for the reaction of 2a (compare entries 1 and 2). 3-Methylphenylsilane (2c), 4-methoxyphenylsilane (2d), and 4-*tert*-butylphenylsilane (2e) gave results similar to those of 2a (compare entries 3–5 with entry 1). 4-Fluorophenylsilane (2f) and 4-chlorophenylsilane (2g) were also giving excellent yields and regioselectivity (entries 6 and 7). The above results indicated that the electrical properties of the substituents on the phenylsilane had no effect on the activity and selectivity of the hydrosilylation reaction. In addition, aliphatic silanes, 4-methylbenzylsilane (2h) and octylsilane (2i) also gave satisfactory results (entries 8 and 9). However, this catalytic method was applicable only to monosubstituted silanes; the iron complexes did not catalyze reactions of disubstituted silanes 2j and trisubstituted silane 2k (entries 10 and 11), perhaps due to the highly steric hindrance of these silanes.

**Table 2:** Iron-catalyzed hydrosilylation of conjugated diene 1a with silanes 2

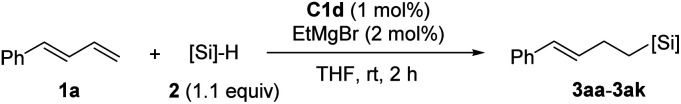				
Entry^a^	[Si]–H	Product	Yield^b^ (%)	r.r.^c^
1	PhSiH_3_2a	3aa	98	>98 : 2
2^d^	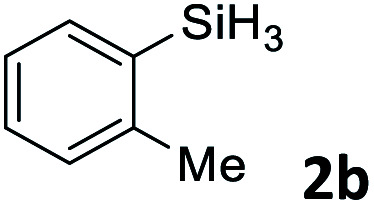	3ab	95	94 : 6
3	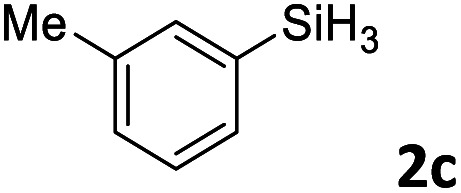	3ac	93	>98 : 2
4	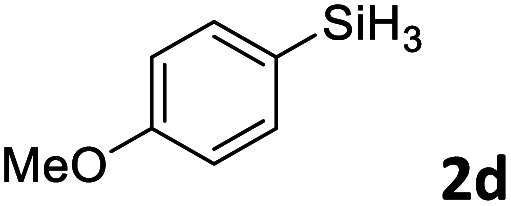	3ad	95	>98 : 2
5	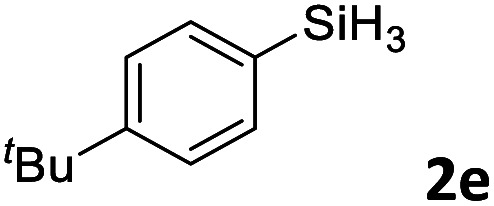	3ae	99	>98 : 2
6	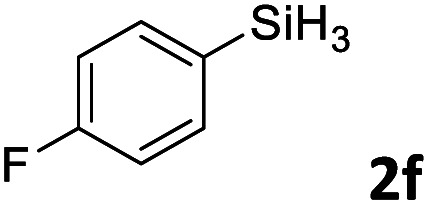	3af	94	>98 : 2
7	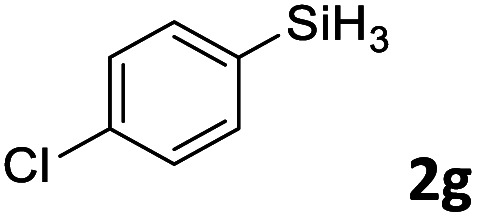	3ag	97	>98 : 2
8	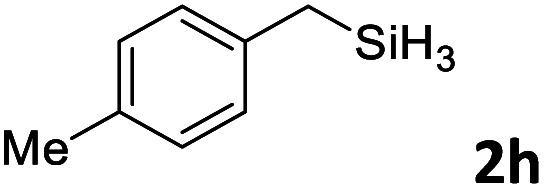	3ah	96	>98 : 2
9	C_8_H_17_SiH_3_2i	3ai	91	98 : 2
10	PhMeSiH_2_2j	3aj	ND	NA
11	(MeO)_2_MeSiH 2k	3ak	ND	NA

a Reaction conditions, unless otherwise noted: 1a (0.7 mmol), 2 (0.77 mmol), C1d (1 mol%), EtMgBr (2 mol%), THF (1 mL), rt, 2 h.

b Isolated yields were given.

c The r.r. values (1,2-*anti*-Markovnikov/1,2-Markovnikov product ratios) were determined by ^1^H NMR spectroscopy.

d Reaction time, 24 h.

**Scheme 3:**
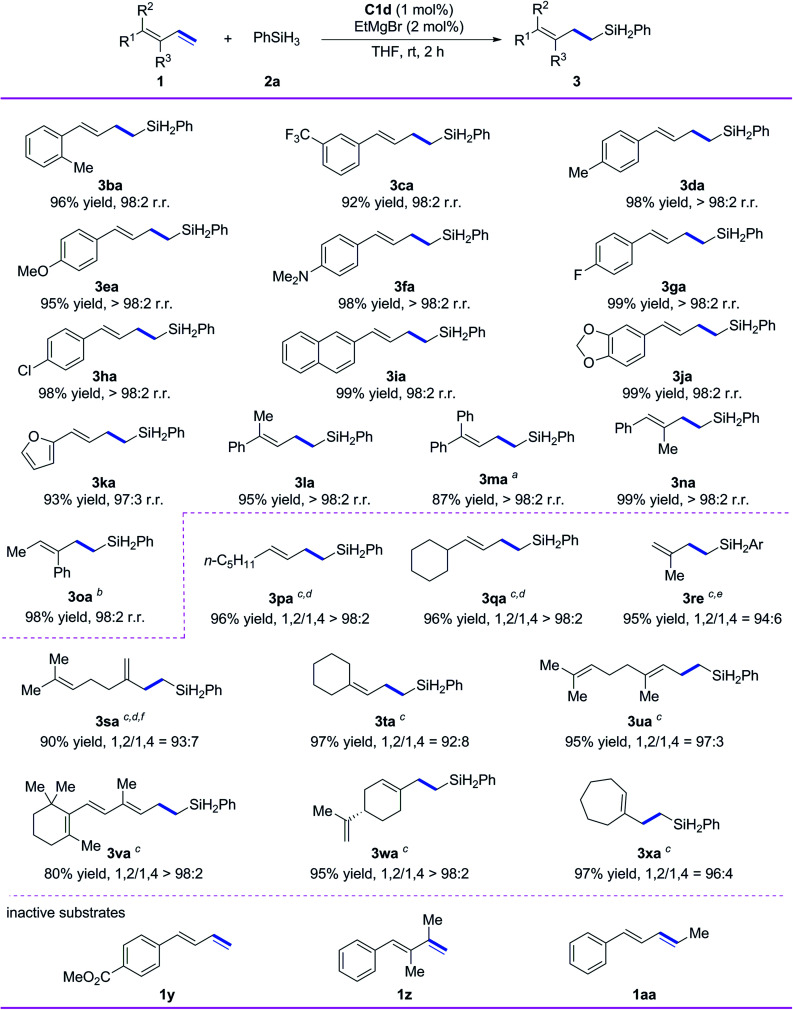
Iron-catalyzed hydrosilylation of conjugated dienes 1 with phenylsilane (2a). Reaction conditions, unless otherwise noted: 1 (0.7 mmol), 2a (0.77 mmol), C1d (1 mol%), EtMgBr (2 mol%), THF (1 mL), rt, 2 h. Isolated yields were given, and r.r. values (1,2-*anti*-Markovnikov/1,2-Markovnikov product ratios) were determined by ^1^H NMR spectroscopy. ^*a*^Complex C1b was used as the catalyst, and the reaction time was increased to 4 h. ^*b*^Amount of C1b, 2 mol%. ^*c*^The notation 1,2/1,4 refers to the ratio of 1,2- and 1,4-*anti*-Markovnikov hydrosilylation products. ^*d*^Complex C1e was used as the catalyst, EtMgBr (4 mol%) was added at −30 °C, and then the mixture was stirred at 0 °C for 10 h. ^*e*^Silane 2e was used instead of 2a. ^*f*^Complex C1d was used as the catalyst.

Subsequently, we evaluated the substrate scope of the reaction with respect to the conjugated diene (Scheme 3). All the tested 1-aryl-substituted 1,3-dienes gave the corresponding 1,2-*anti*-Markovnikov hydrosilylation products (3ba–3ka) with excellent regioselectivity, and neither the electronic properties nor the steric properties of the substituent on the phenyl ring markedly affected the substrate reactivity or the selectivity of the reaction. Notably, 1-naphthyl- and 1-piperonyl-substituted 1,3-dienes gave high yields of 1,2-*anti*-Markovnikov products 3ia and 3ja under the standard conditions. In addition to monoaryl-substituted 1,3-dienes, polysubstituted 1,3-dienes with at least one phenyl substituent—including 1-methyl-1-phenyl-substituted 1,3-diene 1l, 1,1-diphenyl-substituted 1,3-diene 1m, 1-phenyl-2-methyl-1,3-diene 1n, and 1-methyl-2-phenyl-1,3-diene 1o—gave high yields (87–99%) and 1,2-*anti*-Markovnikov selectivity (r.r. ≥ 98 : 2).

We also systematically evaluated reactions of alkyl-substituted conjugated dienes using our catalytic system (Scheme 3). 1-*n*-Pentyl-1,3-diene and 1-cyclohexyl-1,3-diene underwent hydrosilylation smoothly to give 3pa and 3qa in high yields with excellent chemoselectivity and regioselectivity (>98% 1,2-*anti*-Markovnikov selectivity in both cases) when C1e was used as the catalyst and the reaction temperature was decreased. Isoprene was used as a substrate to give the product 3re with 94% selectivity of 1,2-*anti*-Markovnikov. Myrcene gave 1,2-*anti*-Markovnikov product 3sa with 93% selectivity. Both 1,1-dialkyl-substituted 1,3-dienes 1t–1v and 1,2-dialkyl-substituted 1,3-dienes 1w and 1x reacted smoothly under the standard conditions to give target products 3ta–3xa in high yields (80–97%) with 1,2-*anti*-Markovnikov selectivity ranging from 92 : 8 to >98 : 2. It is worth mentioning that 1,2-*anti*-Markovnikov hydrosilylation reactions of 1,1-dialkyl-substituted 1,3-dienes have not been reported previously, and the hydrosilylation of 1x with another catalysts shows a maximum 1,2-*anti*-Markovnikov selectivity of only 67%.^9^ Unfortunately, the catalytic system is inactive to the conjugated diene substrates having strong coordinative functional groups (*e.g.*, 1y), the diene having disubstituted terminal alkene (1z), and the internal diene (1aa).

**Scheme 4:**
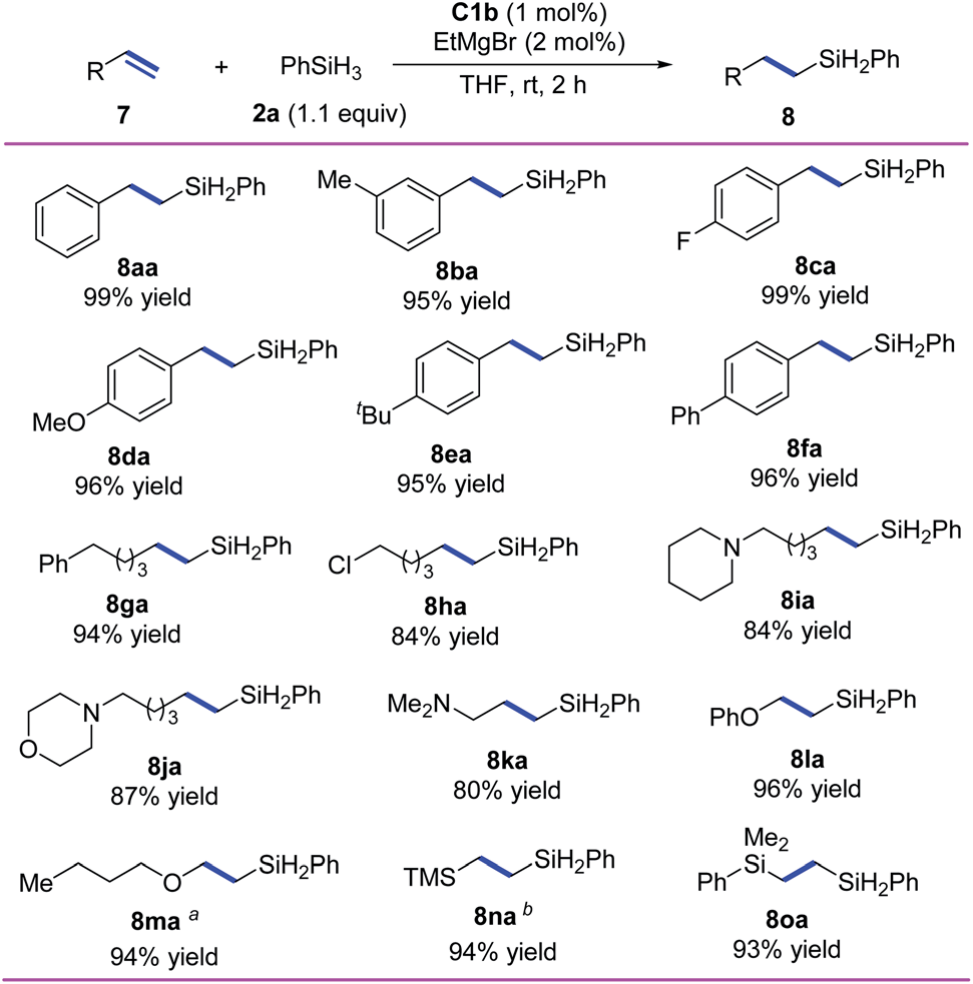
Iron-catalyzed *anti*-Markovnikov hydrosilylation of 1-substituted ethylenes. Reaction conditions, unless otherwise noted: 7 (0.7 mmol), 2a (0.77 mmol), C1b (1 mol%), EtMgBr (2 mol%), THF (1 mL), rt, 2 h. Isolated yields were given, and r.r. values (>98 : 2 in all cases) were determined by ^1^H NMR spectroscopy. ^*a*^Amount of EtMgBr, 5 mol%. ^*b*^Complex C1d was used as the catalyst.

In addition, we investigated the hydrosilylation of simple 1-substituted ethylenes and found that *anti*-Markovnikov selectivity could be achieved by using iron catalyst C1b, which has a smaller substituent than C1d (Scheme 4). All the tested aryl ethylenes smoothly gave the corresponding *anti*-Markovnikov hydrosilylation products (8aa–8fa) in high yields (95–99%), and the tested alkyl ethylenes gave equally good results (8ga–8ka). Notably, the presence of a chlorine or an amine substituent on the alkyl side chain had negligible effects on the reaction (8ha–8ka). In addition, our catalytic system could be used for hydrosilylation reactions of phenyl vinyl ether, vinyl *n*-butyl ether, vinyl trimethylsilane, or dimethyl phenyl vinyl silane with O or Si directly attached to the C=C bond, giving target products 8la–8oa in 93–96% yields with *anti*-Markovnikov selectivity.

**Scheme 5:**
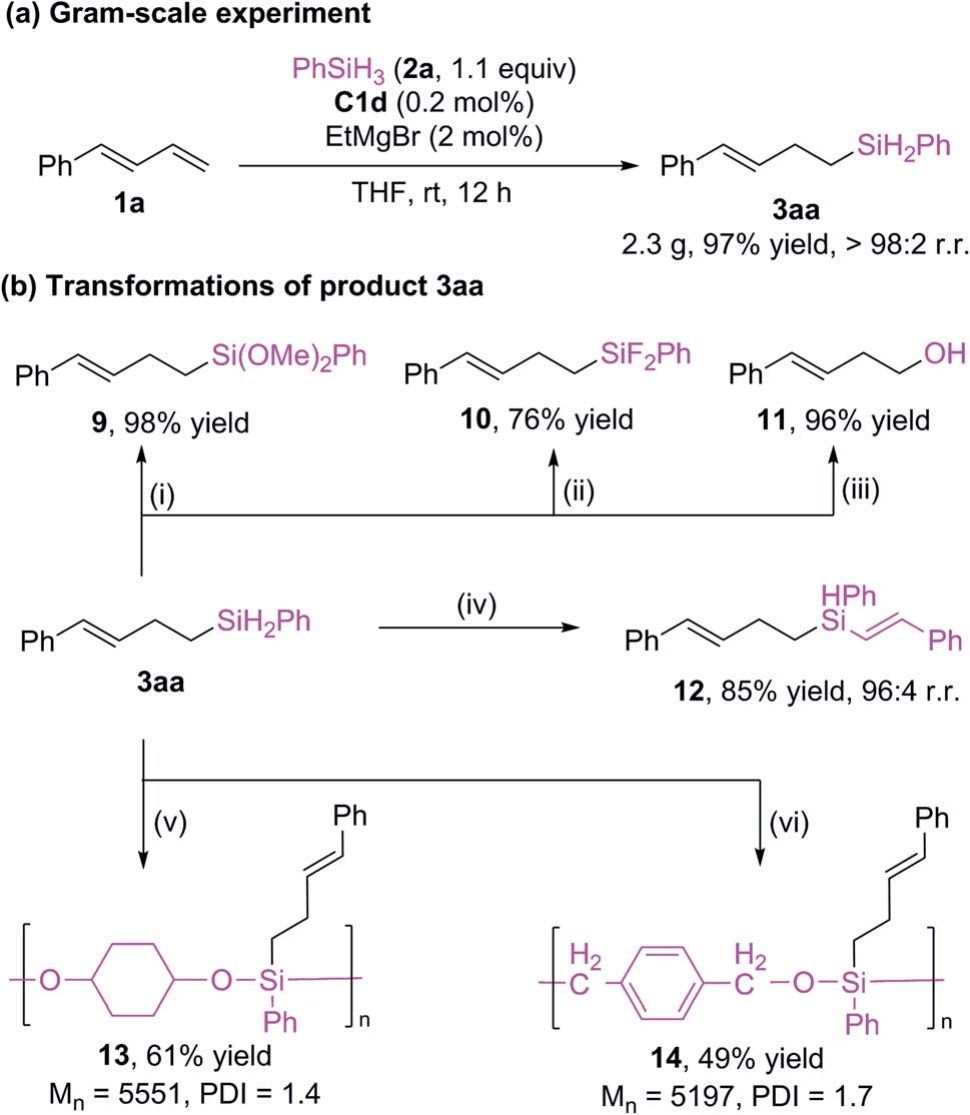
Gram-scale experiment and product transformations. Reaction conditions: (i) 0.5 mol% [RuCl_2_(*p*-cymene)]_2_, MeOH, 0 °C, 10 min. (ii) 5 mol% CuI, 4.2 equiv. CuCl_2_, 2.5 equiv CsF, THF, rt, 18 h. (iii) 5 equiv. K_2_CO_3_, 5 equiv. H_2_O_2_, MeOH/THF (1 : 1, v/v), 50 °C, 8 h. (iv) 2 mol% CoBr_2_, 2.2 mol% Xantphos, 6 mol% NaBHEt_3_, 1 equiv. phenylacetylene, THF, rt, 5 h. (v) 1 equiv. 1,4-cyclohexanediol, 0.5 mol% B(C_6_F_5_)_3_, toluene, rt, 48 h. (vi) 5 mol% [Co], 15 mol% NaBHEt_3_, 1 equiv. terephthalaldehyde, toluene, rt, 48 h.

We reduced the catalyst dosage to 0.2 mol% for the gram-scale experiment and were able to obtain the product with up to 97% yield and >98% r.r. (Scheme 5a). The hydrosilylation product can undergo various transformations (Scheme 5b). The Si–H bonds of 3aa could be transformed to Si–O^16^ bonds (9) and Si–F bonds^5*i*^ (10) with good yields. The silyl group could be oxidized to alcohol 11.^5*i*^ In addition, the Si–H bonds can be further added to alkyne to give a new alkenyl silane 12 with excellent regioselectivity.^17^ Alkylsilane 3aa could also be easily converted to polyorganicsiloxane 13^18^ and 14 ^13*a*^ by means of dehydrogenation coupling with cyclohexanediol and cobalt-catalyzed hydrosilylation reaction with terephthalaldehyde, respectively. These results imply that the current protocol may be used in materials science.

**Scheme 6:**
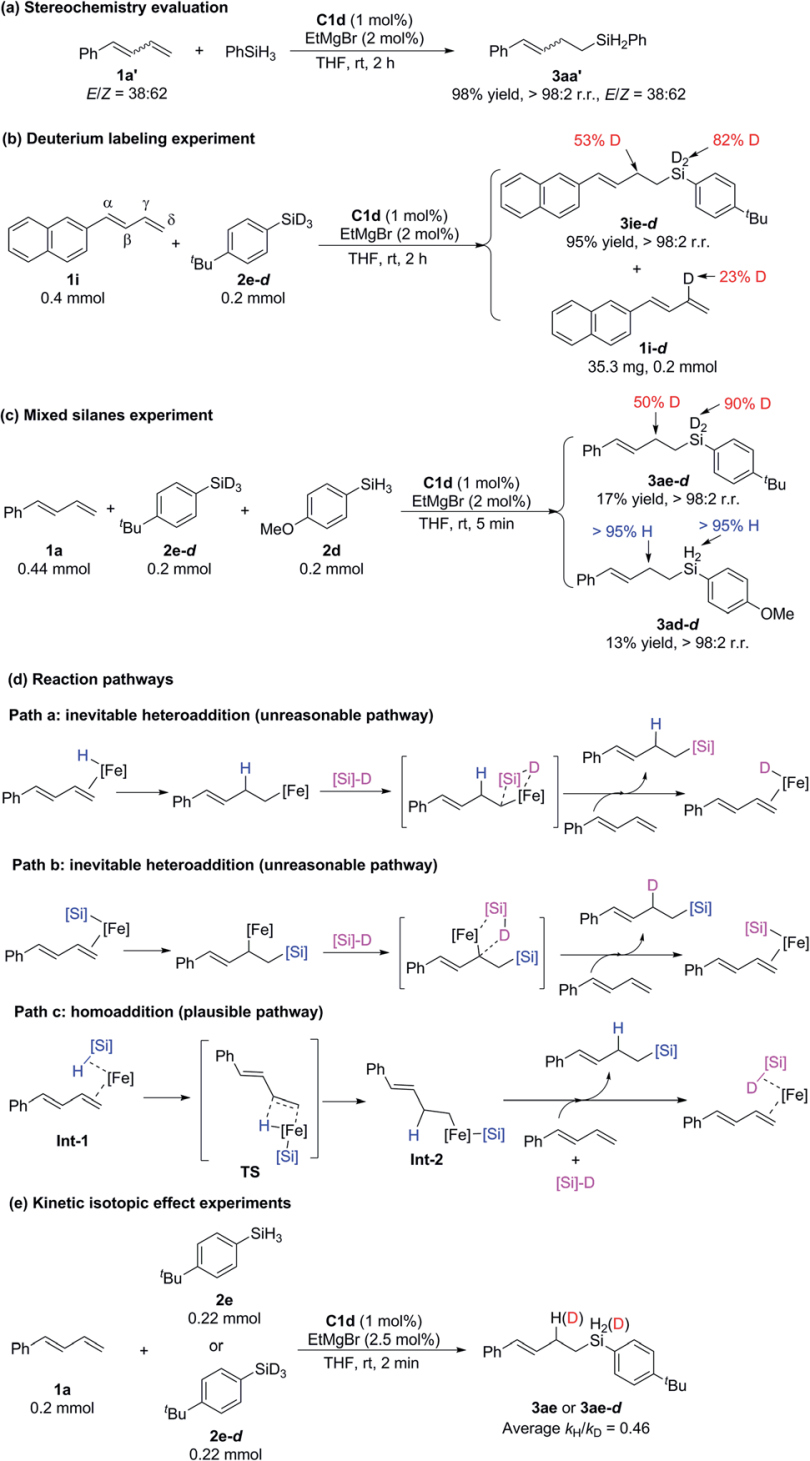
Control experiments and possible reaction pathways.

To investigate the mechanism of these iron-catalyzed hydrosilylation reactions, we performed a series of control experiments (Scheme 6). First, we subjected phenyl-substituted conjugated diene 1a′ (*E*/*Z* = 38 : 62) to a reaction with phenylsilane under the standard conditions and found that the *E*/*Z* ratio of hydrosilylation product 3aa′ was the same as that of the substrate (Scheme 6a). This experimental result showed that *E*/*Z* isomerization of the substrate did not occur during the reaction and that the *Z*-conjugated diene underwent hydrosilylation. This result allows us to exclude any mechanism involving an allylic iron intermediate and also indicates that complexation with the 2-imino-9-aryl-1,10-phenanthroline ligand resulted in crowding around the iron center, such that it could coordinate only to the less sterically bulky, terminal end of the alkene substrate. Second, reaction of 1-naphthyl-1,3-diene 1i with deuterated silane 2e-*d* (98% D) under the standard conditions produced hydrosilylation product 3ie-*d* with D only on the silicon atom (82% D) and the γ carbon atom (53% D), along with recovered substrate 1i-*d* with 23% D on the γ carbon (Scheme 6b). The total deuterium atom was essentially conserved in the reaction shown in Scheme 6b. On the basis of these results, we speculated that the hydrogen transfer step in this reaction was probably reversible. Third, a reaction of 1a with a 1 : 1 mixture of silane 2e-*d* (98% D) and 2d gave 17% and 13% yields of hydrosilylation products 3ae-*d* and 3ad-*d*, respectively, within 5 min (Scheme 6c). This result suggests that the silicon group and the H atom in the product came from the same molecule of silane without generation of crossover products and thus allows us to exclude the possibility that the catalytic cycle was initiated by an Fe(i)–H or Fe(i)–Si species (Scheme 6d, paths a and b, respectively), but by an Fe(0) specie (Scheme 6d, path c). Parallel kinetic isotope effect experiments were performed based on the hydrosilylation reactions between 1a and 2e or 2e-*d* and an inverse KIE (*k*_H_/*k*_D_ = 0.46) was observed (Scheme 6e). According to the literature,^19^ the inverse KIE experiment indicates that the hydrogen transfer step of the hydrosilylation reaction might be a fast and reversible process, which is consistent with the findings of the deuterium labeling experiment (Scheme 6b).

**Scheme 7:**
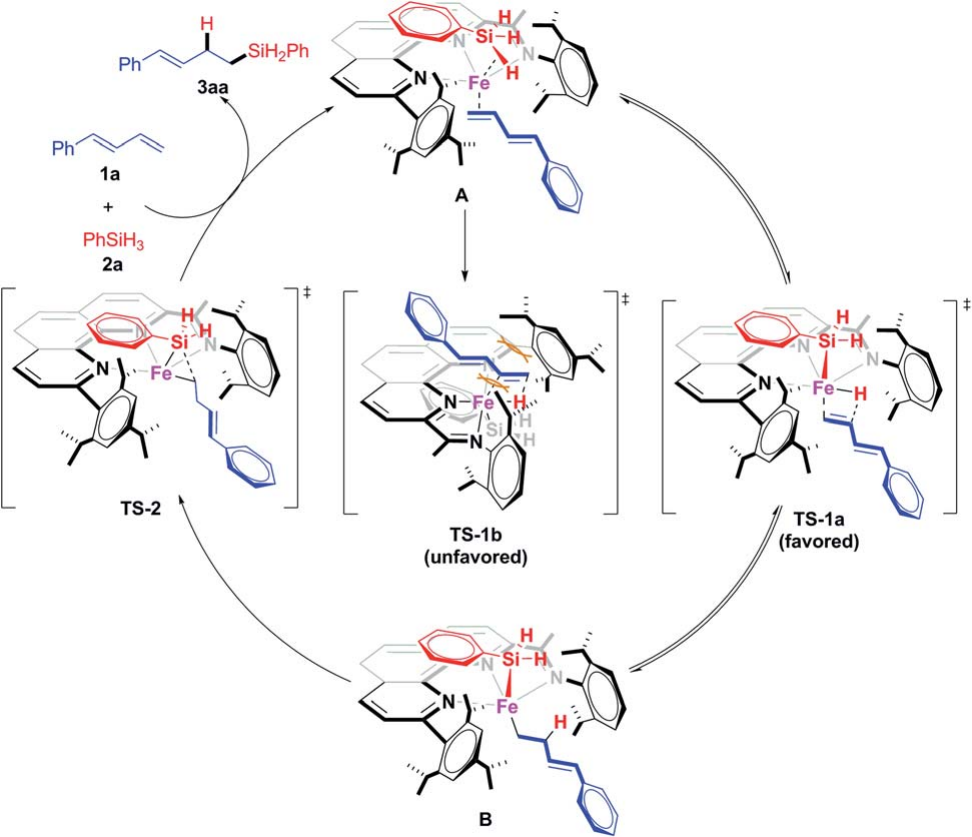
Proposed catalytic cycle and plausible model for regioselectivity.

Thus we proposed that the iron-catalyzed hydrosilylation reactions proceed *via* a Chalk–Harrod-type catalytic mechanism (Scheme 7).^13*a*,20^ That is, the iron catalyst coordinates to the olefin and the silane to form A, which subsequently undergoes a ligand–ligand hydrogen transfer process (*via* transition state TS-1) to generate Fe(ii) intermediate B. This transfer process is the determination step of the regioselectivity. In TS-1b, the conjugated diene has a distinct repulsion interaction with both the aryl groups at the 9-position and 2-imino of the 1,10-phenanthroline ligand, while such interaction is absent in the dominant TS-1a. The iron catalyst modified with 2-imino-9-aryl-1,10-phenanthroline ligands can precisely differentiate the steric hindrance of reaction sites due to the extremely crowded environment around the small iron center, which determined the excellent regioselectivity of this hydrosilylation reactions *via* kinetic control. The intermediate B undergoes reductive elimination *via*Ts-2, releasing the hydrosilylation product 3aa and regenerating the active catalyst. The deuteration labeling experiment and KIE experiment (Scheme 6b and e) show that the transformation from A to B is reversible.

## Conclusions

In summary, newly developed iron complexes bearing 2-imino-9-aryl-1,10-phenanthroline ligands were successfully used to catalyze hydrosilylation of terminal alkenes and conjugated dienes in high yields with excellent *anti*-Markovnikov selectivity. In particular, we achieved the first highly 1,2-*anti*-Markovnikov hydrosilylation reactions of aryl-substituted 1,3-dienes and 1,1-dialkyl-1,3-dienes using these iron catalysts. Mechanistic studies indicated that the reactions involve an Fe(0)–Fe(ii) redox cycle and that the iron center is extremely crowded by the ligand, which accounts for the 1,2-*anti*-Markovnikov selectivity. The relatively small size of iron atom comparing to 4d or 5d metals makes the steric effect of the ligand more remarkable, and accounts for the unprecedented selectivity.

## Data availability

All the data associated with this manuscript were provided in ESI.†

## Author contributions

S.-F. Z. and W. S. conceived the research program and designed and directed the investigations. W. S and M.-P. L carried out the hydrosilylation reactions. W. S., M.-P. L., L.-J. L., Q. H. and M.-Y. H. prepared the ligands and substrates. S.-F. Z. and W. S. wrote the manuscript.

## Conflicts of interest

The authors declare no competing financial interest.

## Supplementary Material

SC-013-D1SC06727C-s001

## References

[cit1] Bolm C., Legros J., Le Paih J., Zani L. (2004). Chem. Rev..

[cit2] Appl M. , Ullmann's Encyclopedia of Industrial Chemistry, Wiley-VCH, Weinheim, 7th edn, 2011, vol. 3, pp. 107–261

[cit3] Marciniec B. (2005). Coord. Chem. Rev..

[cit4] Du X.-Y., Huang Z. (2017). ACS Catal..

[cit5] Bart S. C., Lobkovsky E., Chirik P. J. (2004). J. Am. Chem. Soc..

[cit6] Lappert M. F., Nile T. A., Takahashi S. (1974). J. Organomet. Chem..

[cit7] Hatanaka Y., Goda K.-I., Yamashita F., Hiyama T. (1994). Tetrahedron Lett..

[cit8] Parker S. E., Börgel J., Ritter T. (2014). J. Am. Chem. Soc..

[cit9] Raya B., Jing S., Balasanthiran V., RajanBabu T. V. (2017). ACS Catal..

[cit10] Kuai C.-S., Ji D.-W., Zhao C.-Y., Liu H., Hu Y.-C., Chen Q.-A. (2020). Angew. Chem., Int. Ed..

[cit11] Sang H.-L., Yu S.-J., Ge S.-Z. (2018). Chem. Sci..

[cit12] Greenhalgh M. D., Frank D. J., Thomas S. P. (2014). Adv. Synth. Catal..

[cit13] Hu M.-Y., He P., Qiao T.-Z., Sun W., Li W.-T., Lian J., Li J.-H., Zhu S.-F. (2020). J. Am. Chem. Soc..

[cit14] Guo N., Hu M.-Y., Feng Y., Zhu S.-F. (2015). Org. Chem. Front..

[cit15] Sun W.-H., Jie S.-Y., Zhang S., Zhang W., Song Y.-X., Ma H.-W., Chen J.-T., Wedeking K., Fröhlich R. (2006). Organometallics.

[cit16] Ojima Y., Yamaguchi K., Mizuno N. (2009). Adv. Synth. Catal..

[cit17] Wu C.-Z., Teo W. J., Ge S.-Z. (2018). ACS Catal..

[cit18] Gevorgyan V., Liu J.-X., Rubin M., Benson S., Yamamoto Y. (1999). Tetrahedron Lett..

[cit19] Jones W. D. (2003). Acc. Chem. Res..

[cit20] Chalk A. J., Harrod J. F. (1965). J. Am. Chem. Soc..

