# Supplementary material for: Phenanthroline-imine ligands for iron-catalyzed alkene hydrosilylation
Source: Chem Sci. 2022 Feb 10;13(9):2721–8. doi: 10.1039/d1sc06727c (PMC8890093; doi:10.1039/d1sc06727c)
Supplement: SC-013-D1SC06727C-s001 [file SC-013-D1SC06727C-s001.pdf]

Electronic Supplementary Material (ESI) for Chemical Science.
This journal is © The Royal Society of Chemistry 2022

Supplementary Information for:

Phenanthroline-Imine Ligands for Iron-Catalyzed Alkene Hydrosilylation

Wei Sun,[a] Ming-Peng Li,[a] Lu-Jie Li,[a] Qiang Huang,[a] Meng-Yang Hu,[a] Shou-Fei Zhu*,[a,b]
[a] *Frontiers Science Center for New Organic Matter, State Key Laboratory and Institute of Elemento-Organic Chemistry, College of Chemistry, Nankai University, Tianjin 300071, China.*
[b] *Haihe Laboratory of Sustainable Chemical Transformations, Tianjin 300070, China.*
Email: sfzhu@nankai.edu.cn

Table of Contents

1. Materials and Methods……………………………………………..…………………S2

2. Synthesis of New Ligands and Catalysts..……………………………………..…...S3

3. Additional Optimization of Reaction Conditions….……………………………………S17

4. Typical Procedures for Conjugated Dienes and Alkenes Hydrosilylation…............S20

5. Analytical Data of the Hydrosilylation Products…………………………………..…..S21

6. Gram-scale Experiment and Product Transformations…………………………..….S41

7. Mechanistic Studies……………………….....………………………….……….......S48

8. NMR Spectras of All the Products…….………………………………...…...……....S56

9. References………………………………………………………………………...……S129

1. Materials and methods

All manipulations were carried out using standard Schlenk, high-vacuum, and glovebox techniques. THF, Et_2O, 1,4-dioxane, and toluene were distilled from sodium benzophenone ketyl prior to use. Iron(II) chloride (99.99%) was purchased from Sigma-Aldrich and used as received. The conjugated dienes **1a**, **1c**, **1d**, **1e**, **1g**, **1h**, **1k**, **1n**, **1p**, **1w, 1y, 1z** and **1aa** used for hydrosilylation were synthesized according to reported procedures.[1] The conjugated dienes **1b**, **1f**, **1i**, **1j and 1q** were synthesized according to reported procedures.[1,2] The conjugated dienes **1l**,[3] **1m**,[4] **1o**,[1,5] **1t**,[6] **1u**,[7] and **1x**[8] were synthesized according to corresponding literatures. The silanes **2a**, **2j**, **2k** conjugated diene **1r**, **1s**, and alkenes **7a**-**7h**, **7k**-**7o** were purchased from Sigma-Aldrich, Alfa Aesar, Acros, TCI and other reagent companies. Alkenes **7i** and **7j** were synthesized according to reported procedures.[9] The silanes **2b-2i** and **2e-*d*** were synthesized according to reported procedures.[10] Conjugated dienes, alkenes, and silanes were dried over $LiAlH_4$ or CaH_2 and distilled prior to use. 2,9-dichloro-1,10-phenanthroline was prepared according to reported procedures.[11]

Melting points were measured on a RY-I apparatus and uncorrected. Infrared spectra were recorded on a Bruker Fourier transform spectrometric (FT-IR) and reported in wave number. High resolution mass spectrometric (HRMS) were determined on an IonSpec FT-ICR mass spectrometer or a Waters GCT Premier mass spectrometer. The molar masses and their distribution for the polymer samples were determined by GPC on a Waters system equipped with a set of three Ultrastyragel columns HT2 30 cm x 7.8 mm; 10 µm particles; exclusion limits: 100-10000 g/mol, respectively), THF was used as the mobile phase (1 mL/min), and polystyrene samples as the standards in the calibration of the molar masses. ^1H NMR, ^{13}C NMR and ^{19}F NMR spectra were recorded with a Bruker AV 400 spectrometer at 400 MHz (^1H NMR), 101 MHz (^{13}C NMR) and 376 MHz (^{19}F NMR), respectively in $CDCl_3$. ^2H NMR spectra was recorded with a Bruker AV 600 spectrometer at 92 MHz in DCM. Chemical shifts were reported in ppm down field from internal Me_4Si (^1H NMR) and $CDCl_3$ (^{13}C NMR).

2. Synthesis of new ligands and catalysts

2.1. Synthesis of 2-chloro-9-aryl-1,10-phenanthrolines

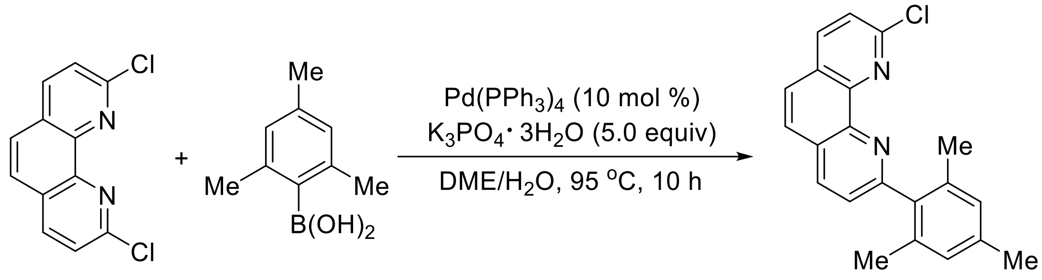

1 (1.1 equiv) **2a**

To a 100 mL three-necked round-bottom flask fitted with a reflux condenser tube, 2,9-dichloro-1,10-phenanthroline (1.24 g, 5 mmol), 2,4,6-trimethylphenylboronic acid (0.9 g, 5.5 mmol, 1.1 equiv), $Pd(PPh_3)_4$ (577 mg, 0.5 mmol, 10 mol%), K_3PO_4•$3H_2O$ (6.65 g, 25 mmol, 5 equiv), DME (50 mL), and water (5 mL) were introduced. The resulted reaction mixture was degassed three times through freeze degassing, replaced with an argon atmosphere, and placed in an oil bath, and heated to 95 ˚C until the reaction was finished. After cooling to room temperature, the reaction mixture was neutralized with saturated NH_4Cl (aq., 50 mL) and extracted with CH_2Cl_2 (100 mL × 3). The combined organic layer was dried over anhydrous $MgSO_4$ and concentrated under reduced pressure. The residue was purified by silica-gel column chromatography with petroleum ether/ethyl acetate (PE/EA) = 10:1 (v/v) as eluent to give 2-chloro-9-(2,4,6- trimethylphenyl)-1,10-phenanthroline (**2a**) as a white solid (1.1 g, 66% yield), melting point: 231.2 – 233.0 °C.

^{1}H NMR (400 MHz, $CDCl_3$) δ 8.28 (d, J = 8.2 Hz, 1H), 8.20 (d, J = 8.4 Hz, 1H), 7.89 – 7.84 (m, 1H), 7.82 – 7.77 (m, 1H), 7.63 – 7.55 (m, 2H), 6.97 (s, 2H), 2.35 (s, 3H), 2.14 (s, 6H).

^{13}C NMR (101 MHz, $CDCl_3$) δ 160.7 (1C), 151.2 (1C), 146.3 (1C), 145.0 (1C), 138.6 (2C), 137.9 (1C), 137.5 (1C), 136.1 (1C), 135.8 (1C), 128.4 (2C), 127.5 (1C), 127.3 (1C), 126.8 (1C), 125.4 (2C), 124.1 (1C), 21.1 (1C), 20.6 (2C).

HRMS (ESI) calcd for [M+H, $C_{21}H_{18}ClN_2$]$^+$: 333.1153, found: 333.1156.

2-chloro-9-(2,4,6-triisopropylphenyl)-1,10-phenanthroline (**2b**)

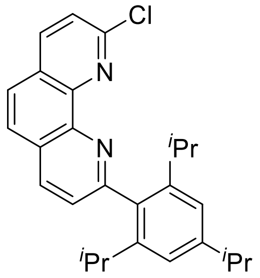

White solid, 83% yield, melting point: 248.0 – 250.0 °C.

^{1}H NMR (400 MHz, $CDCl_3$) δ 8.26 (d, *J* = 8.1 Hz, 1H), 8.17 (d, *J* = 8.4 Hz, 1H), 7.86 (d, *J* = 8.7 Hz, 1H), 7.79 (d, *J* = 8.7 Hz, 1H), 7.65 (d, *J* = 8.2 Hz, 1H), 7.58 (d, *J* = 8.4 Hz, 1H), 7.12 (s, 2H), 2.96 (hept, *J* = 6.9 Hz, 1H), 2.59 (hept, *J* = 6.9 Hz, 2H), 1.32 (d, *J* = 6.9 Hz, 6H), 1.19 (d, *J* = 6.8 Hz, 6H), 1.12 (d, *J* = 6.9 Hz, 6H).

^{13}C NMR (101 MHz, $CDCl_3$) δ 161.1 (1C), 151.2 (1C), 148.9 (1C), 146.5 (1C), 146.4 (2C), 144.9 (1C), 138.4 (1C), 137.0 (1C), 135.2 (1C), 127.6 (1C), 127.4 (1C), 126.8 (1C), 125.6 (1C), 125.4 (1C), 124.2 (1C), 120.8 (2C), 34.5 (1C), 30.5 (2C), 24.2 (6C).

HRMS (ESI) calcd for [M+H, $C_{27}H_{30}ClN_2$]$^+$: 417.2092, found 417.2093.

2-chloro-9-(3,5-di-*tert*-butylphenyl)-1,10-phenanthroline (**2c**)

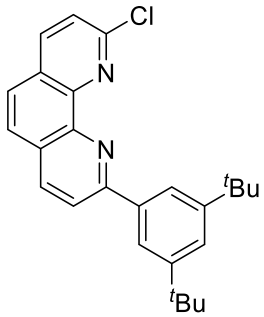

White solid, 95% yield, melting point: 285.5 – 287.9 °C.

^{1}H NMR (400 MHz, $CDCl_3$) δ 8.33 (d, *J* = 8.4 Hz, 1H), 8.20 (d, *J* = 8.4 Hz, 1H), 8.15 – 8.06 (m, 3H), 7.86 (d, *J* = 8.7 Hz, 1H), 7.77 (d, *J* = 8.7 Hz, 1H), 7.63 (d, *J* = 8.3 Hz, 1H), 7.58 (t, *J* = 1.8 Hz, 1H), 1.45 (s, 18H).

^{13}C NMR (101 MHz, $CDCl_3$) δ 159.3 (1C), 151.4 (1C), 151.2 (2C), 146.3 (1C), 144.9 (1C), 139.2 (1C), 138.6 (1C), 136.6 (1C), 127.7 (1C), 127.6 (1C), 126.7 (1C), 125.1 (1C),

124.1 (1C), 123.8 (1C), 122.4 (2C), 121.6 (1C), 35.1 (2C), 31.6 (6C).

HRMS (ESI) calcd for [M+H, $C_{26}H_{28}ClN_2$]$^+$: 403.1936, found 403.1938.

2.2. Synthesis of 2-acetyl-9-aryl-1,10-phenanthrolines

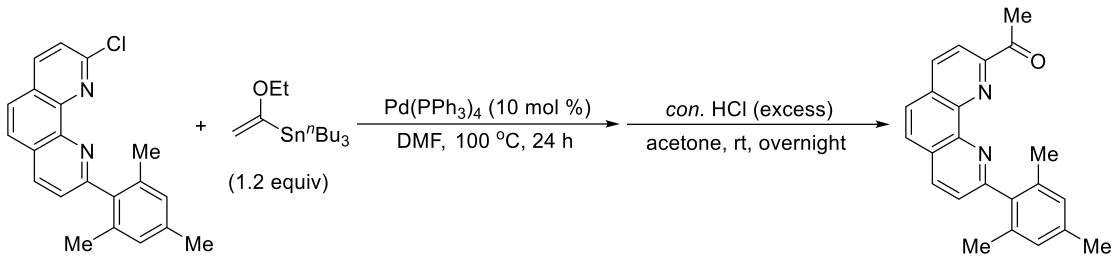

2a **3a**

Compound **2a** (0.8 g, 2.4 mmol) and Pd(PPh_3)$_4$ (277 mg, 0.24 mmol, 10 mol%) were introduced into a 100 mL three-necked round-bottom flask fitted with a reflux condenser tube. The reaction system was replaced with an argon atmosphere, followed by the addition of 1-ethoxyvinyltri-*n*-butylstannane (1.04 g, 2.88 mmol, 1.2 equiv) and anhydrous DMF (30 ml) using a syringe, respectively, and then stirred at 100 °C for 24 h until the reaction was finished. After cooling to room temperature, the reaction mixture was quenched with saturated KF (aq., 50 mL) and extracted with Et_2O (100 mL × 3). The combined organic layer was dried over anhydrous Na_2SO_4 and concentrated under reduced pressure. The crude product was dissolved in acetone (30 mL), and was added dropwise concentrated hydrochloric acid (6 mL) with stirring overnight. Complete consumption of the reactants was determined by TLC. The reaction mixture was neutralized with aqueous $NaHCO_3$ solution, the acetone was removed under reduced pressure, extracted with DCM, dried with anhydrous Na_2SO_4, and concentrated under reduced pressure. The residue was purified by silica-gel column chromatography with PE/EA = 10:1 (v/v) as eluent to give the target product 1-(9-mesityl-1,10-phenanthrolin-2-yl)ethan-1-one (**3a**) as a white solid (628 mg, 76% yield), melting point: 237.5 – 238.2 °C.

^{1}H NMR (400 MHz, $CDCl_3$) δ 8.41 – 8.26 (m, 3H), 7.93 (d, *J* = 8.8 Hz, 1H), 7.84 (d, *J* = 8.8 Hz, 1H), 7.66 (d, *J* = 8.2 Hz, 1H), 7.02 (s, 2H), 2.97 (s, 3H), 2.37 (s, 3H), 2.27 (s, 6H).

^{13}C NMR (101 MHz, $CDCl_3$) δ 201.2 (1C), 160.3 (1C), 153.0 (1C), 145.9 (1C), 145.4 (1C), 137.7 (1C), 137.6 (1C), 136.8 (1C), 136.4 (1C), 135.6 (1C), 130.6 (1C), 128.8 (3C), 128.6 (1C), 127.0 (1C), 125.8 (1C), 125.6 (1C), 120.0 (1C), 25.9 (1C), 21.1 (1C), 20.9 (2C).

HRMS (ESI) calcd for $[M+H, C_{23}H_{21}N_2O]^+$: 341.1648, found 341.1652.

1-(9-(2,4,6-triisopropylphenyl)-1,10-phenanthrolin-2-yl)ethan-1-one (3b)

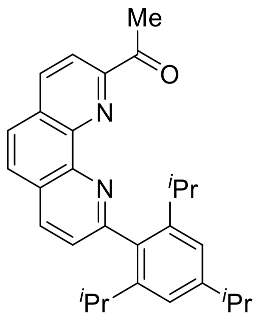

White solid, 98% yield, melting point: 185.7 – 186.1 °C.

^{1}H NMR (400 MHz, $CDCl_3$) δ 8.34 (q, J = 8.3 Hz, 2H), 8.28 (d, J = 8.1 Hz, 1H), 7.95 (d, J = 8.7 Hz, 1H), 7.85 (d, J = 8.8 Hz, 1H), 7.68 (d, J = 8.1 Hz, 1H), 7.18 (s, 2H), 3.07 – 2.96 (m, 1H), 2.94 (s, 3H), 2.69 (hept, J = 6.0 Hz, 2H), 1.36 (d, J = 6.8 Hz, 6H), 1.26 (d, J = 6.7 Hz, 6H), 1.19 (d, J = 6.8 Hz, 6H).

^{13}C NMR (101 MHz, $CDCl_3$) δ 201.5 (1C), 160.6 (1C), 153.0 (1C), 148.8 (1C), 146.7 (2C), 145.9 (1C), 145.6 (1C), 136.7 (1C), 136.6 (1C), 135.2 (1C), 130.7 (1C), 128.6 (1C), 127.1 (1C), 125.8 (1C), 125.5 (1C), 120.9 (2C), 119.9 (1C), 34.4 (1C), 30.7 (2C), 26.2 (1C), 24.9 (2C), 24.1 (4C).

HRMS (ESI) calcd for $[M+H, C_{29}H_{33}N_2O]^+$: 425.2587, found 425.2589.

1-(9-(3,5-di-*tert*-butylphenyl)-1,10-phenanthrolin-2-yl)ethan-1-one (3c)

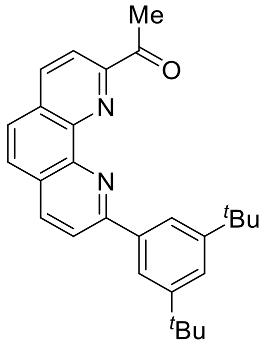

White solid, 67% yield, melting point: 160.2 – 162.1 °C.

^{1}H NMR (400 MHz, $CDCl_3$) δ 8.40 – 8.31 (m, 5H), 8.22 (d, J = 8.4 Hz, 1H), 7.92 (d, J = 8.8 Hz, 1H), 7.81 (d, J = 8.7 Hz, 1H), 7.61 – 7.58 (m, 1H), 3.14 (s, 3H), 1.47 (s, 18H).

^{13}C NMR (101 MHz, $CDCl_3$) δ 200.9 (1C), 158.2 (1C), 152.7 (1C), 151.3 (2C), 145.9 (1C), 145.4 (1C), 138.4 (1C), 136.9 (1C), 136.8 (1C), 130.9 (1C), 128.6 (1C), 127.6 (1C), 125.5 (1C), 124.1 (1C), 122.0 (2C), 120.3 (1C), 120.0 (1C), 35.1 (2C), 31.5 (6C), 25.4 (1C).

HRMS (ESI) calcd for [M+H, $C_{28}H_{31}N_2O$]$^+$: 411.2431, found 411.2433.

2.3 Synthesis of 2-imino-9-aryl-1,10-phenanthrolines ligands

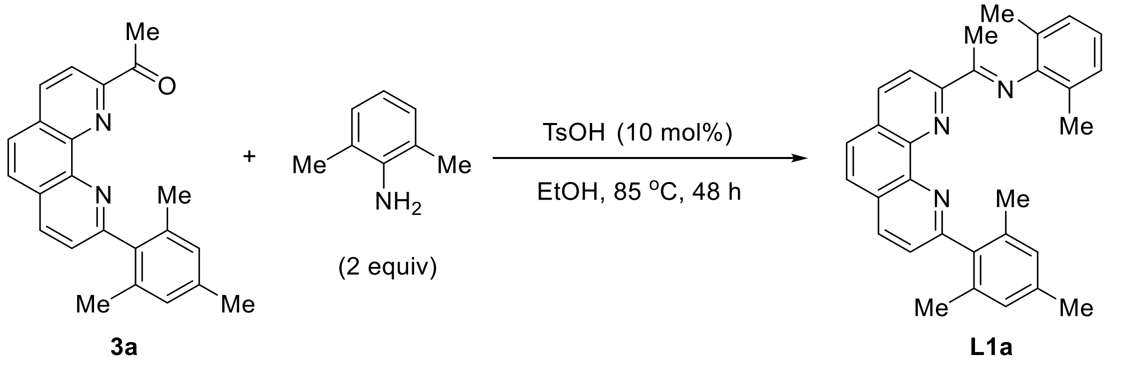

To a 100 mL three-necked round-bottom flask fitted with a reflux condenser tube, was added the compound **3a** (342 mg, 1.0 mmol) and the catalyst TsOH (17.2 mg, 0.1 mmol, 10 mol%). The resulted mixture was replaced with an argon atmosphere, added 2,6-dimethylaniline (242 mg, 2.0 mmol, 2.0 equiv) and anhydrous ethanol (10 mL), and stirred in an oil bath at 85 °C for 48 h until the reaction was finished. The reaction mixture was

concentrated under reduced pressure, and the residue was purified by silica-gel column chromatography with PE/EA/Et_3N= 50:10:1 (v/v/v) as eluent to give the target product (*E*)-N-(2,6-dimethylphenyl)-1-(9-mesityl-1,10-phenanthrolin-2-yl)ethan-1-imine (**L1a**) as a yellow solid (330 mg, 74% yield), melting point: 236.0 – 237.0 °C.

^{1}H NMR (400 MHz, $CDCl_3$) δ 8.76 (d, *J* = 8.4 Hz, 1H), 8.31 (d, *J* = 8.4 Hz, 1H), 8.26 (d, *J* = 8.2 Hz, 1H), 7.88-7.80 (m, 2H), 7.61 (d, *J* = 8.2 Hz, 1H), 7.07 (d, *J* = 7.5 Hz, 2H), 6.99 (s, 2H), 6.97-6.90 (m, 1H), 2.44 (s, 3H), 2.34 (s, 3H), 2.27 (s, 6H), 2.04 (s, 6H).

^{13}C NMR (101 MHz, $CDCl_3$) δ 168.3 (1C), 159.9 (1C), 155.9 (1C), 149.0 (1C), 146.0 (1C), 145.4 (1C), 137.7 (1C), 137.6 (1C), 136.5 (2C), 136.3 (1C), 135.7 (1C), 129.6 (1C), 128.8 (2C), 127.9 (2C), 127.4 (1C), 127.0 (1C), 126.1 (1C), 125.3 (3C), 123.0 (1C), 120.6 (1C), 21.2 (1C), 21.1 (2C), 18.1 (2C), 16.8 (1C).

HRMS (ESI) calculated for [M+H, $C_{31}H_{30}N_3$]$^+$: 444.2434, found: 444.2438.

(*E*)-N-(2,6-dimethylphenyl)-1-(9-(2,4,6-triisopropylphenyl)-1,10-phenanthrolin-2-yl)ethan-1-imine (L1b)

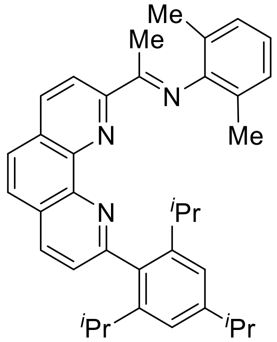

Yellow solid, 64% yield, melting point: 211.2 – 213.4 °C.

^{1}H NMR (400 MHz, $CDCl_3$) δ 8.73 (d, *J* = 8.4 Hz, 1H), 8.33 (d, *J* = 8.4 Hz, 1H), 8.27 (d, *J* = 8.2 Hz, 1H), 7.92 – 7.84 (m, 2H), 7.65 (d, *J* = 8.1 Hz, 1H), 7.15 (s, 2H), 7.09 – 7.04 (m, 2H), 6.97 – 6.91 (m, 1H), 2.99 (hept, *J* = 6.9 Hz, 1H), 2.73 (p, *J* = 6.8 Hz, 2H), 2.39 (s, 3H), 2.05 (s, 6H), 1.34 (d, *J* = 6.9 Hz, 6H), 1.24 (d, *J* = 6.7 Hz, 6H), 1.17 (d, *J* = 6.9 Hz, 6H).

^{13}C NMR (101 MHz, $CDCl_3$) δ 168.6 (1C), 160.2 (1C), 155.9 (1C), 149.0 (1C), 148.7 (1C),

146.8 (2C), 145.9 (1C), 145.6 (1C), 136.7 (1C), 136.1 (1C), 135.1 (1C), 129.6 (1C), 127.8 (2C), 127.3 (1C), 127.0 (2C), 126.0 (1C), 125.4 (1C), 125.2 (1C), 122.9 (1C), 120.9 (2C), 120.4 (1C), 34.3 (1C), 30.6 (2C), 24.9 (2C), 24.2 (2C), 24.1 (2C), 18.0 (2C), 17.0 (1C).

HRMS (ESI) calcd for [M+H, $C_{37}H_{42}N_3$]$^+$: 528.3373, found: 528.3375.

(*E*)-N-(2,6-diethylphenyl)-1-(9-(2,4,6-triisopropylphenyl)-1,10-phenanthrolin-2-yl)ethan-1-imine (**L1c**)

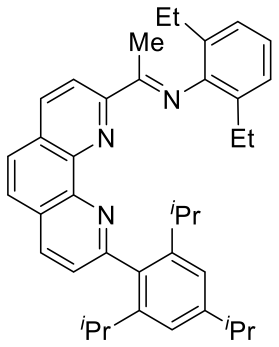

Yellow solid, 67% yield, melting point: 204.9 – 206.2 °C.

^{1}H NMR (400 MHz, $CDCl_3$) δ 8.72 (d, J = 8.4 Hz, 1H), 8.32 (d, J = 8.4 Hz, 1H), 8.26 (d, J = 8.2 Hz, 1H), 7.92 – 7.82 (m, 2H), 7.64 (d, J = 8.2 Hz, 1H), 7.15 (s, 2H), 7.13 – 7.08 (m, 2H), 7.06 – 7.00 (m, 1H), 2.99 (hept, J = 6.9 Hz, 1H), 2.73 (hept, J = 6.8 Hz, 2H), 2.50 – 2.29 (m, 7H), 1.34 (d, J = 6.9 Hz, 6H), 1.24 (d, J = 6.8 Hz, 6H), 1.19 – 1.11 (m, 12H).

^{13}C NMR (101 MHz, $CDCl_3$) δ 168.3 (1C), 160.2 (1C), 156.0 (1C), 148.7 (1C), 148.0 (1C), 146.8 (2C), 146.0 (1C), 145.6 (1C), 136.8 (1C), 136.1 (1C), 135.1 (1C), 131.2 (2C), 129.6 (1C), 127.3 (1C), 127.0 (1C), 126.0 (1C), 125.9 (2C), 125.2 (1C), 123.2 (1C), 120.9 (2C), 120.3 (1C), 34.4 (1C), 30.6 (2C), 24.9 (2C), 24.6 (2C), 24.1 (4C), 17.3 (1C), 13.7 (2C).

HRMS (ESI) calcd for [M+H, $C_{39}H_{46}N_3$]$^+$: 556.3686, found: 556.3689.

(*E*)-N-(2,6-diisopropylphenyl)-1-(9-(2,4,6-triisopropylphenyl)-1,10-phenanthrolin-2-yl)ethan-1-imine (**L1d**)

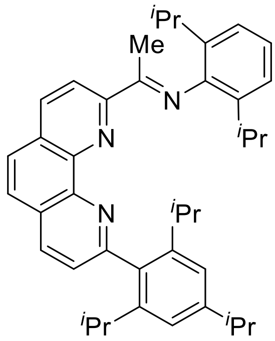

Yellow solid, 90% yield, melting point: 249.0 – 251.2 °C.

^{1}H NMR (400 MHz, $CDCl_3$) δ 8.72 (d, *J* = 8.4 Hz, 1H), 8.33 (d, *J* = 8.4 Hz, 1H), 8.26 (d, *J* = 8.1 Hz, 1H), 7.93 – 7.83 (m, 2H), 7.64 (d, *J* = 8.2 Hz, 1H), 7.19 – 7.14 (m, 4H), 7.13 – 7.06 (m, 1H), 2.99 (hept, *J* = 6.9 Hz, 1H), 2.77 (dp, *J* = 24.9, 6.8 Hz, 4H), 2.42 (s, 3H), 1.34 (d, *J* = 6.9 Hz, 6H), 1.24 (d, *J* = 6.8 Hz, 6H), 1.19 – 1.11 (m, 18H).

^{13}C NMR (101 MHz, $CDCl_3$) δ 168.3 (1C), 160.1 (1C), 155.9 (1C), 148.6 (1C), 146.8 (2C), 146.7 (1C), 146.0 (1C), 145.6 (1C), 136.8 (1C), 136.1 (1C), 135.7 (2C), 135.1 (1C), 129.6 (1C), 127.3 (1C), 127.0 (1C), 126.0 (1C), 125.2 (1C), 123.5 (1C), 122.9 (2C), 120.9 (2C), 120.4 (1C), 34.3 (1C), 30.6 (2C), 28.2 (2C), 24.9 (2C), 24.2 (2C), 24.1 (2C), 23.2 (2C), 22.9 (2C), 17.6 (1C).

HRMS (ESI) calcd for $[M+H, C_{41}H_{50}N_3]^+$: 584.3999, found 584.4003.

(*E*)-N-(2,6-dibenzhydryl-4-methoxyphenyl)-1-(9-(2,4,6-triisopropylphenyl)-1,10-phenanthrolin-2-yl)ethan-1-imine (**L1e**)

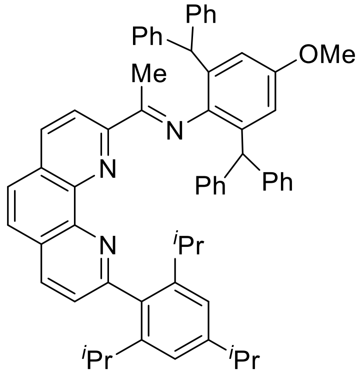

Yellow solid, 66% yield, melting point: 127.1 – 129.0 °C. The amount of arylamine is 1.2 equivalents.

^{1}H NMR (400 MHz, $CDCl_3$) δ 8.24 (q, *J* = 9.0, 8.5 Hz, 3H), 7.85 (q, *J* = 8.8 Hz, 2H), 7.62 (d, *J* = 8.1 Hz, 1H), 7.24 – 6.92 (m, 22H), 6.46 (s, 2H), 5.33 (s, 2H), 3.56 (s, 3H), 3.02 (p, *J* = 7.0 Hz, 1H), 2.62 (p, *J* = 6.9 Hz, 2H), 1.38 (d, *J* = 6.9 Hz, 6H), 1.32 (s, 3H), 1.15 (dd, *J* = 16.5, 6.8 Hz, 12H).

^{13}C NMR (101 MHz, $CDCl_3$) δ 171.0 (1C), 159.2 (1C), 155.0 (1C), 154.0 (1C), 147.6 (1C), 145.7 (2C), 145.0 (1C), 144.5 (1C), 142.5 (2C), 141.3 (1C), 141.1 (2C), 136.0 (1C), 134.7 (1C), 134.0 (1C), 132.6 (2C), 128.7 (4C), 128.4 (4C), 128.3 (1C), 127.4 (4C), 126.9 (4C), 126.1 (1C), 125.9 (1C), 125.2 (2C), 125.0 (3C), 123.8 (1C), 119.7 (2C), 119.3 (1C), 112.7 (2C), 54.1 (1C), 51.1 (2C), 33.4 (1C), 29.7 (2C), 23.9 (2C), 23.2 (2C), 22.8 (2C), 16.3 (1C).

HRMS (ESI) calcd for $[M+H, C_{62}H_{60}N_3O]^+$: 862.4731, found: 862.4735.

(*E*)-1-(9-(3,5-di-*tert*-butylphenyl)-1,10-phenanthrolin-2-yl)-N-(2,6-dimethylphenyl)ethan-1-imine (L1f)

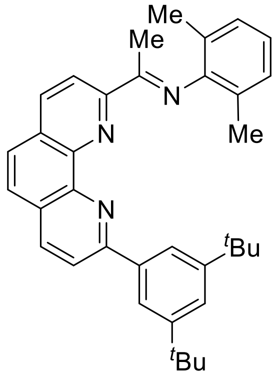

Yellow solid, 48% yield, melting point: 202.8 – 203.3 °C.

^{1}H NMR (400 MHz, $CDCl_3$) δ 8.80 (d, *J* = 8.4 Hz, 1H), 8.37 – 8.31 (m, 4H), 8.21 (d, *J* = 8.4 Hz, 1H), 7.91 – 7.81 (m, 2H), 7.59 – 7.54 (m, 1H), 7.12 (d, *J* = 7.5 Hz, 2H), 7.02 – 6.95 (m, 1H), 2.64 (s, 3H), 2.10 (s, 6H), 1.43 (s, 18H).

^{13}C NMR (101 MHz, $CDCl_3$) δ 168.0 (1C), 157.8 (1C), 155.5 (1C), 151.1 (2C), 149.1 (1C), 145.9 (1C), 145.2 (1C), 138.5 (1C), 136.8 (1C), 136.3 (1C), 129.7 (1C), 127.9 (2C),

127.5 (1C), 127.3 (1C), 125.7 (1C), 125.3 (2C), 123.9 (1C), 123.0 (1C), 121.9 (2C), 120.3 (1C), 119.9 (1C), 35.0 (2C), 31.5 (6C), 18.0 (2C), 16.4 (1C).

HRMS (ESI) calcd for [M+H, $C_{36}H_{40}N_3]^+$: 514.3217, found: 514.3220.

(*E*)-1-(9-(3,5-di-*tert*-butylphenyl)-1,10-phenanthrolin-2-yl)-N-(2,6-diisopropylphenyl)ethan-1-imine (L1g)

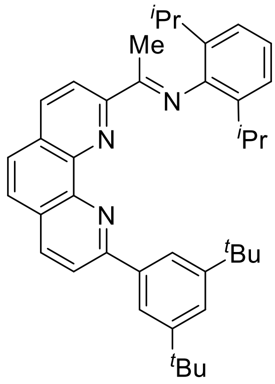

Yellow solid, 98% yield, melting point: 281.5 – 282.9 ºC.

^{1}H NMR (400 MHz, $CDCl_3$) δ 8.79 (d, *J* = 8.4 Hz, 1H), 8.40 – 8.28 (m, 4H), 8.21 (d, *J* = 8.4 Hz, 1H), 7.86 (q, *J* = 8.7 Hz, 2H), 7.55 (s, 1H), 7.24 – 7.18 (m, 2H), 7.18 – 7.09 (m, 1H), 2.85 (p, *J* = 6.9 Hz, 2H), 2.66 (s, 3H), 1.42 (s, 18H), 1.17 (d, *J* = 6.8 Hz, 12H).

^{13}C NMR (101 MHz, $CDCl_3$) δ 167.8 (1C), 157.8 (1C), 155.6 (1C), 151.2 (2C), 146.8 (1C), 145.9 (1C), 145.2 (1C), 138.5 (1C), 136.8 (1C), 136.3 (1C), 135.8 (2C), 129.7 (1C), 127.5 (1C), 127.2 (1C), 125.7 (1C), 123.9 (1C), 123.6 (1C), 123.0 (2C), 121.9 (2C), 120.3 (1C), 119.9 (1C), 35.1 (2C), 31.5 (6C), 28.3 (2C), 23.3 (2C), 22.9 (2C), 17.1 (1C).

HRMS (ESI) calcd for [M+H, $C_{40}H_{48}N_3]^+$: 570.3843, found: 570.3846.

2.4 Preparation of iron complexes with 2-imino-9-aryl-1,10-phenanthroline ligands

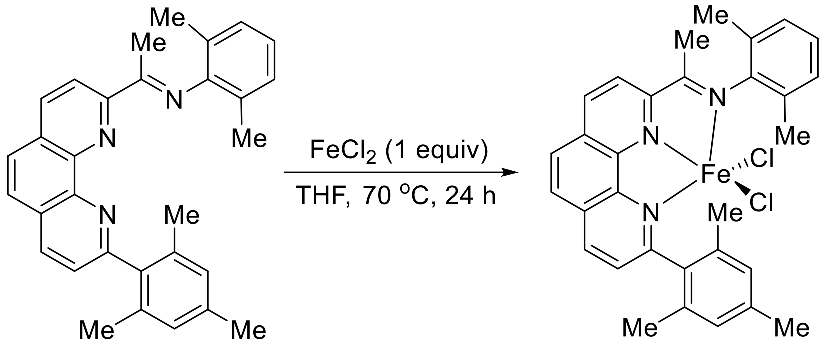

L1a **C1a**

In an argon-filled glovebox, a tube with branch seal (125 mL) was charged with **L1a** (90 mg, 0.2 mmol), $FeCl_2$ (25.3 mg, 1 equiv) and dry THF (20 mL). The reaction mixture was stirred at 70 ˚C for 24 h until the free ligand was fully consumed according to ^{1}H NMR analysis. The solvent was partially removed under vacuum (about 5 mL left), then dry *n*-hexane (15 mL) was added, and solids precipitated. The product was collected by filtration, washed with 20 mL *n*-hexane, and dried under vacuum to give dark blue solid **C1a** (110 mg, 96% yield), decomposition temperature: 270 $^{\circ}$C

<u>^{1}H NMR</u> (400 MHz, $CDCl_3$) δ 70.82, 47.63, 30.79, 28.78, 18.25, 9.08, 7.84, 4.21, 3.77, 2.78, 2.04, 1.86, 1.26, 0.88, -3.47, -5.90, -20.93.

IR (neat): 2912w, 1617m, 1558m, 1500m, 1435m, 1371m, 1295m, 1255m, 1204m, 1158m, 1140m, 1112m, 1091m, 1066m, 1034m, 989w, 912w, 898w, 866s, 846m, 789m, 777m, 764m, 752w, 734m, 652w, 619m cm^{-1}.

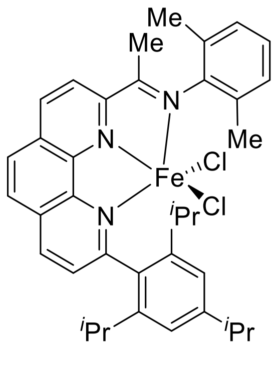

C1b, gray-green solid, 96% yield, decomposition temperature: 260 $^{\circ}$C.

^{1}H NMR (400 MHz, $CDCl_3$) δ 69.70, 46.31, 39.20, 32.23, 31.79, 19.29, 6.03, 5.82, 5.48, 4.20, 1.75, 1.41, 1.37, 1.34, 1.25, 1.19, 0.87, -3.49, -6.23, -28.34, -48.96.

IR (KBr): 3053w, 2959s, 2926m, 2868m, 1611m, 1499m, 1465m, 1442m, 1376m, 1304m, 1266m, 1206m, 1149w, 867m, 792w, 765s, 742s, 703w cm^{-1}.

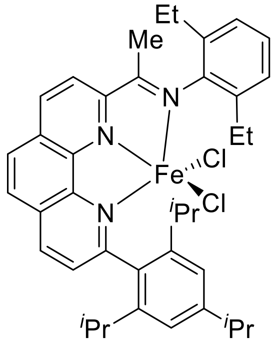

C1c, green solid, 94% yield, decomposition temperature: 236 °C.

^{1}H NMR (400 MHz, $CDCl_3$) δ 69.47, 46.22, 39.59, 34.02, 31.55, 19.33, 6.12, 5.88, 5.68, 4.17, 2.04, -2.61, -4.24, -6.39, -13.52, -28.93, -48.95.

IR (KBr): 2959s, 2930m, 2868m, 2361s, 2342s, 1609m, 1575m, 1507s, 1458s, 1374s, 1299s, 1267s, 1245s, 1192s, 1059m, 865s, 785s, 735m cm^{-1}.

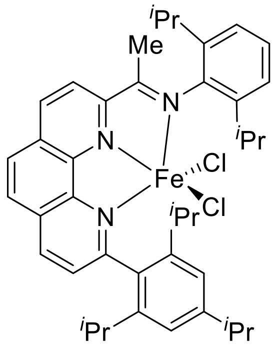

C1d, dark blue solid, 96% yield, decomposition temperature: 290 °C.

^{1}H NMR (400 MHz, $CDCl_3$) δ 69.49, 53.02, 47.24, 45.42, 32.26, 19.67, 7.52, 6.16, 5.62, 5.42, 2.51, -0.58, -2.10, -8.17, -9.74, -17.41, -33.98, -36.73, -62.40.

IR (KBr): 2959s, 2927m, 2867m, 2359w, 2333w, 1609m, 1559m, 1497m, 1461m, 1382m, 1303m, 1189m, 1139w, 1109w, 1057w, 864m, 783m, 735w cm^{-1}.

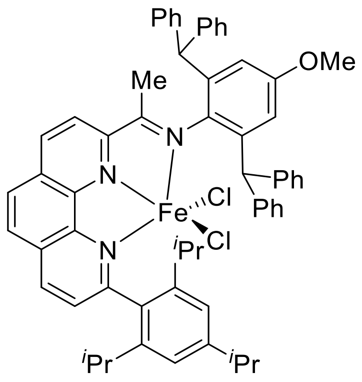

C1e, dark green solid, 96% yield, decomposition temperature: >320 °C.

<u>^{1}H NMR</u> (400 MHz, $CDCl_3$) δ 63.39, 56.47, 51.54, 49.42, 33.93, 21.38, 11.52, 10.71, 8.67, 7.99, 7.30, 6.11, 3.84, 1.72, 0.88, -9.20, -11.60, -23.81, -38.84, -65.42.

<u>IR (KBr):</u> 3547m, 3478s, 3414s, 3240w, 2958w, 2360w, 2341w, 1638m, 1617m, 1495w, 1436w, 1373w, 1305w, 1205w, 864w, 703m, 624m, 605m, 484w cm^{-1}.

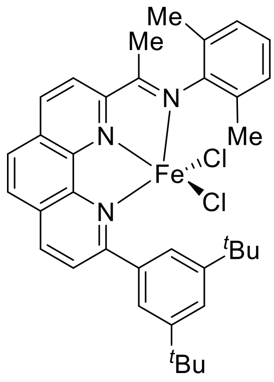

C1f, green solid, 94% yield, decomposition temperature: 220 °C.

<u>^{1}H NMR</u> (400 MHz, $CDCl_3$) δ 79.50, 44.28, 31.28, 27.29, 15.77, 15.00, 10.21, 4.02, 1.92, -1.58, -5.02, -6.13, -22.41, -27.33.

<u>IR (KBr)</u>: 3062w, 2958s, 2907m, 2867m, 1615m, 1597m, 1557m, 1500s, 1470m, 1420m, 1371m, 1297w, 1267w, 1203m, 1140w, 1094w, 862s, 799w, 768s, 709m, 629w cm^{-1}.

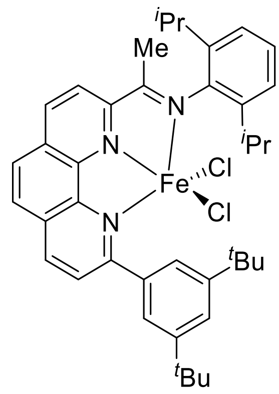

C1g, dark green solid, 92% yield, decomposition temperature: 248 ^{o}C.

<u>^{1}H NMR</u> (400 MHz, $CDCl_3$) δ 82.81, 42.19, 34.99, 27.35, 13.57, 10.43, 7.28, 4.08, 3.46, -5.84, -6.55, -6.70, -9.97, -26.40, -37.71, -39.22.

<u>IR (KBr)</u>: 3060w, 2962s, 2867m, 2360s, 2341s, 1605m, 1557m, 1498m, 1458m, 1371s, 1298s, 1244m, 1188m, 1057w, 934w, 863s, 809m, 794m, 779m, 731w cm^{-1}.

3. Additional optimization of reaction conditions

3.1 Effect of additives and solvents

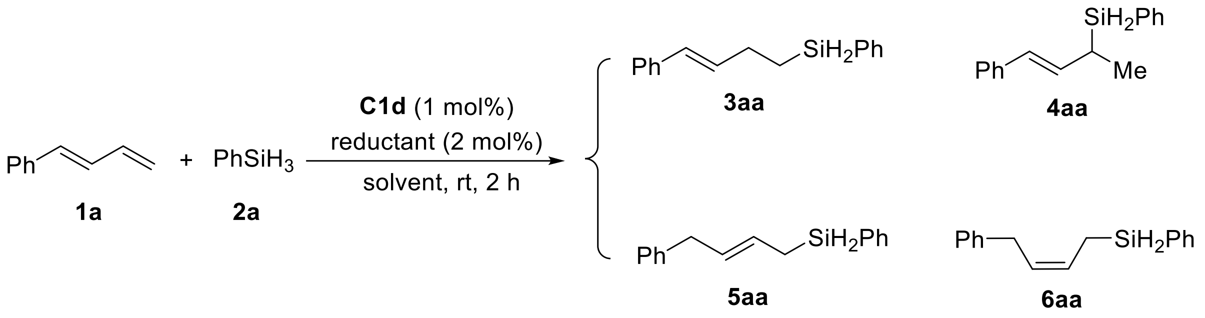

General procedure: In an argon-filled glovebox, a vial (10 mL) was charged with **C1d** (3.6 mg, 0.005 mmol, 1 mol%) and solvent (1 mL). The reaction mixture was stirred at room temperature for 1 min, then was added the conjugated diene **1a** (65 mg, 0.5 mmol), $PhSiH_3$ (59.4 mg, 0.55 mmol) and additive (0.01 mmol, 2 mol%). After stirring for 2 h at room temperature, the vial was removed from the glovebox and the reaction mixture was concentrated by rotary evaporation. Iron species were removed by flash column chromatography with DCM as eluent. The raw product was detected by ^1H NMR with 1,3,5-trimethoxybenzene as internal standard. The results were listed as following.

Table S1 Effect of additives and solvents

entry	[Fe]	reductant	solvent	conv. (%)	yield (%)	**3aa**/**4aa**/**5aa**/**6aa**
1	**C1d**	EtMgBr	THF	>95	99	99:1:0:0
2	**C1d**	MeMgBr	THF	>95	98	98:2:0:0
3	**C1d**	MeMgCl	THF	>95	97	98.5:1.5:0:0
4	**C1d**	PhMgBr	THF	>95	99	98:2:0:0
5	**C1d**	$ZnEt_2$	THF	<5	ND^a	NA^b
6	**C1d**	$LiAlH_4$	THF	<5	ND	NA
7	**C1d**	$NaHBEt_3$	THF	56	22	88:12:0:0
8	**C1d**	^{t}BuOK	THF	75	38	91:9:0:0
9	**C1d**	LDA	THF	>95	20	93:7:0:0

10	**C1d**	none	THF	<5	ND	NA
11	**C1d**	EtMgBr	Et_2O	59	45	98:2:0:0
12	**C1d**	EtMgBr	dioxane	<5	ND	NA
13	**C1d**	EtMgBr	toluene	62	40	98:2:0:0
14	**C1d**	EtMgBr	hexane	67	57	98:2:0:0

[a] ND, not detected. [b] NA, not applicable.

3.2 Effect of metal salts

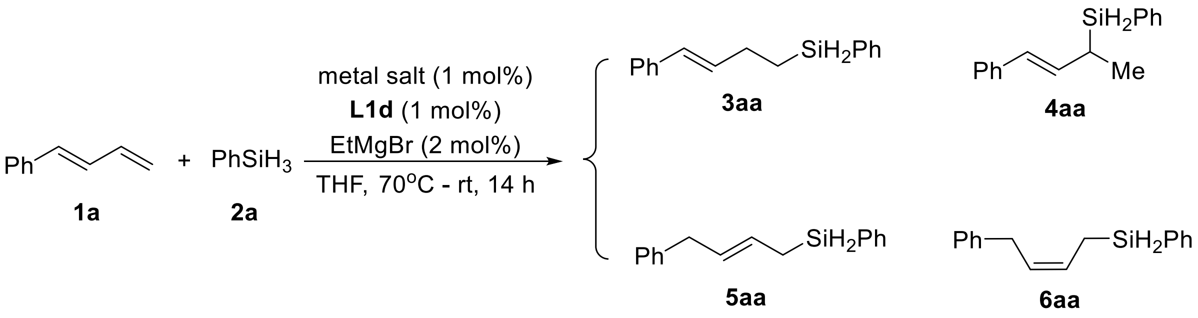

General procedure: In an argon-filled glovebox, a vial (10 mL) was charged with metal salt (0.005 mmol, 1 mol%), **L1d** (2.9 mg, 0.005 mmol, 1 mol%) and THF (1 mL). The reaction mixture was stirred at 70 ˚C for 12 h, and then cooled to room temperature. The conjugated diene **1a** (65 mg, 0.5 mmol), $PhSiH_3$ (59.4 mg, 0.55 mmol, 1.1 equiv) and EtMgBr (10 μL, 0.01 mmol, 2 mol%) was added and the mixture was stirred for 2 h at room temperature. The vial was removed from the glovebox and the reaction mixture was concentrated by rotary evaporation. Metal species were removed by flash column chromatography with DCM as eluent. The raw product was detected by ^{1}H NMR with 1,3,5-trimethoxybenzene as internal standard. The results were listed as following.

Table S2 Effect of metal salts

entry	metal salt	conv. (%)	yield (%)	**3aa/4aa/5aa/6aa**
1	$FeCl_2$	>95	98	98:2:0:0
2	$Fe(acac)_2$	<5	ND[a]	NA[b]

3	$CoCl_2$	<5	ND	NA
4	$NiCl_2$	<5	ND	NA
5	$MnCl_2$	<5	ND	NA
6	$CuCl_2$	<5	ND	NA
7	$ZnCl_2$	<5	ND	NA
8	none	<5	ND	NA

[a] ND, not detected. [b] NA, not applicable.

4. Typical procedures for hydrosilylation reactions

4.1. Hydrosilylation of conjugated dienes

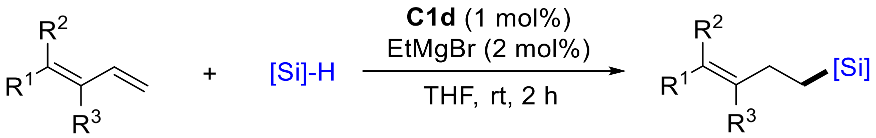

1 **2** **3**

In an argon-filled glovebox, a vial (10 mL) was charged with complexes **C1d** (5.0 mg, 0.007 mmol), dry anhydrous THF (1 mL), conjugated dienes **1** (0.7 mmol) and silanes **2** (0.77 mmol, 1.1 equiv) successively. The vial was removed from the glovebox after EtMgBr (1 M in THF, 14 µL, 0.014 mmol, 2.0 mol%) was added and the reaction mixture was stirred at room temperature for 2 h. When the reaction was finished, the mixture was concentrated by rotating evaporation. The residue was purified by column chromatography to afford the desired products **3**.

4.2 Hydrosilylation of terminal alkenes

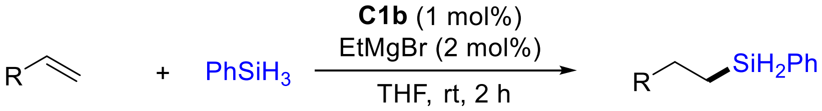

7 **2a** **8**

In an argon-filled glovebox, a vial (10 mL) was charged with complexes **C1b** (4.6 mg, 0.007 mmol), dry anhydrous THF (1 mL), alkene **7** (0.7 mmol) and phenylsilane (0.77 mmol, 1.1 equiv) successivelly. The vial was removed from the glovebox after EtMgBr (1 M in THF, 14 µL, 0.014 mmol, 2.0 mol%) was added and the reaction mixture was stirred at room temperature for 2 h. When the reaction was finished, the mixture was concentrated by rotating evaporation. The residue was purified by column chromatography to afford the desired product **8**.

5. Analytical data of hydrosilylation products

(*E*)-phenyl(4-phenylbut-3-en-1-yl)silane (3aa)[12]

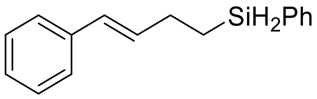

Serial number: sw-2-76, 162.6 mg, 98% yield, >98:2 r.r., colorless oil, R_f= 0.52 (PE)

<u>^{1}H NMR</u> (400 MHz, $CDCl_3$) δ 7.62 – 7.55 (m, 2H), 7.43 – 7.26 (m, 7H), 7.23 – 7.15 (m, 1H), 6.37 (d, *J* = 15.8 Hz, 1H), 6.25 (dt, *J* = 15.7, 6.5 Hz, 1H), 4.34 (t, *J* = 3.6 Hz, 2H), 2.41 – 2.32 (m, 2H), 1.18 – 1.10 (m, 2H).

<u>^{13}C NMR</u> (101 MHz, $CDCl_3$) δ 137.7 (1C), 135.3 (2C), 132.2 (2C), 129.6 (1C), 129.2 (1C), 128.4 (2C), 128.0 (2C), 126.9 (1C), 126.0 (2C), 28.4 (1C), 9.9 (1C).

(*E*)-(4-phenylbut-3-en-1-yl)(*o*-tolyl)silane (3ab)

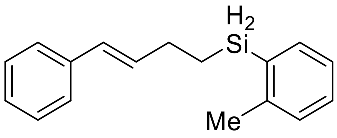

Serial number: sw-3-43, 167.0 mg, 95% yield, 94:6 r.r., colorless oil. R_f= 0.59 (PE).

<u>^{1}H NMR</u> (400 MHz, $CDCl_3$) δ 7.56 – 7.50 (m, 1H), 7.34 – 7.25 (m, 5H), 7.22 – 7.14 (m, 3H), 6.37 (d, *J* = 15.7 Hz, 1H), 6.25 (dt, *J* = 15.8, 6.5 Hz, 1H), 4.37 (t, *J* = 3.7 Hz, 2H), 2.45 (s, 3H), 2.40 – 2.32 (m, 2H), 1.19 – 1.09 (m, 2H).

<u>^{13}C NMR</u> (101 MHz, $CDCl_3$) δ 144.0 (1C), 137.7 (1C), 136.4 (1C), 132.2 (1C), 131.6 (1C), 130.1 (1C), 129.4 (1C), 129.2 (1C), 128.4 (2C), 126.8 (1C), 126.0 (2C), 125.2 (1C), 28.6 (1C), 22.6 (1C), 9.6 (1C).

<u>HRMS (EI)</u> calculated for [M, $C_{17}H_{20}Si$]$^+$:252.1334, found: 252.1328.

(*E*)-(4-phenylbut-3-en-1-yl)(*m*-tolyl)silane (3ac)

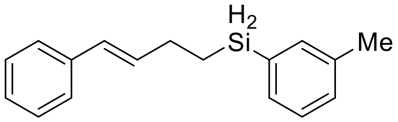

Serial number: sw-3-44, 163.6 mg, 93% yield, >98:2 r.r., colorless oil. R_f= 0.50 (PE).

<u>^{1}H NMR</u> (400 MHz, $CDCl_3$) δ 7.42 – 7.35 (m, 2H), 7.33 – 7.24 (m, 5H), 7.23 – 7.15 (m, 2H), 6.37 (d, *J* = 15.7 Hz, 1H), 6.25 (dt, *J* = 15.8, 6.5 Hz, 1H), 4.32 (t, *J* = 3.7 Hz, 2H), 2.42 – 2.29 (m, 5H), 1.18 – 1.05 (m, 2H).

<u>^{13}C NMR</u> (101 MHz, $CDCl_3$) δ 137.8 (1C), 137.5 (1C), 136.0 (1C), 132.3 (3C), 130.5 (1C), 129.3 (1C), 128.5 (2C), 128.0 (1C), 126.9 (1C), 126.0 (2C), 28.5 (1C), 21.5 (1C), 10.0 (1C).

<u>HRMS (EI)</u> calculated for [M, $C_{17}H_{20}Si$]$^+$: 252.1334, found: 252.1328.

(*E*)-(4-methoxyphenyl)(4-phenylbut-3-en-1-yl)silane (3ad)

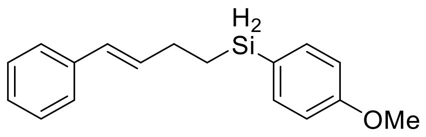

Serial number: sw-2-144, 178.0 mg, 95% yield, >98:2 r.r., colorless oil. R_f= 0.30 (PE).

<u>^{1}H NMR</u> (400 MHz, $CDCl_3$) δ 7.55 – 7.45 (m, 2H), 7.32 – 7.25 (m, 4H), 7.23 – 7.14 (m, 1H), 6.95 – 6.86 (m, 2H), 6.37 (d, *J* = 15.8 Hz, 1H), 6.24 (dt, *J* = 15.7, 6.5 Hz, 1H), 4.32 (t, *J* = 3.6 Hz, 2H), 3.81 (s, 3H), 2.40 – 2.31 (m, 2H), 1.15 – 1.07 (m, 2H).

<u>^{13}C NMR</u> (101 MHz, $CDCl_3$) δ 160.9 (1C), 137.7 (2C), 136.7 (1C), 132.3 (1C), 129.2 (1C), 128.4 (2C), 126.8 (1C), 126.0 (2C), 122.8 (1C), 113.9 (2C), 55.0 (1C), 28.4 (1C), 10.2 (1C).

<u>HRMS (EI)</u> calculated for [M, $C_{17}H_{20}OSi$]$^+$: 268.1283, found: 268.1278.

(*E*)-(4-(*tert*-butyl)phenyl)(4-phenylbut-3-en-1-yl)silane (3ae)

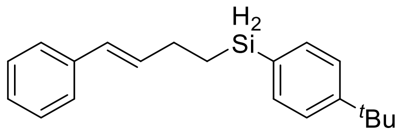

Serial number: sw-2-194, 204 mg, 99% yield, >98:2 r.r., colorless oil. R_f= 0.48 (PE).

<u>^{1}H NMR</u> (400 MHz, $CDCl_3$) 7.56 – 7.49 (m, 2H), 7.43 – 7.37 (m, 2H), 7.33 – 7.25 (m, 4H), 7.21 – 7.15 (m, 1H), 6.37 (d, *J* = 15.7 Hz, 1H), 6.24 (dt, *J* = 15.7, 6.5 Hz, 1H), 4.33

(t, J = 3.6 Hz, 2H), 2.42 – 2.32 (m, 2H), 1.32 (s, 9H), 1.18 – 1.08 (m, 2H).

^{13}C NMR (101 MHz, $CDCl_3$) δ 152.7 (1C), 137.7 (1C), 135.2 (2C), 132.3 (1C), 129.2 (1C), 128.6 (1C), 128.4 (2C), 126.8 (1C), 126.0 (2C), 125.0 (2C), 34.7 (1C), 31.2 (3C), 28.5 (1C), 10.0 (1C).

HRMS (EI) calculated for [M, $C_{20}H_{26}Si]^+$: 294.1804, found: 294.1796.

(*E*)-(4-fluorophenyl)(4-phenylbut-3-en-1-yl)silane (3af)

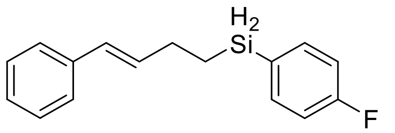

Serial number: sw-4-126, 169.4 mg, 94% yield, >98:2 r.r., colorless oil. R_f= 0.78 (PE).

^{1}H NMR (400 MHz, $CDCl_3$) δ 7.59 – 7.51 (m, 2H), 7.33 – 7.26 (m, 4H), 7.23 – 7.16 (m, 1H), 7.10 – 7.02 (m, 2H), 6.36 (d, J = 17.2 Hz, 1H), 6.23 (dt, J = 15.7, 6.6 Hz, 1H), 4.33 (t, J = 3.6 Hz, 2H), 2.41 – 2.30 (m, 2H), 1.17 – 1.07 (m, 2H).

^{13}C NMR (101 MHz, $CDCl_3$) δ 164.0 (J = 250.5 Hz, 1C), 162.8 (1C), 137.6 (1C), 137.2 (1C), 137.2 (1C), 131.9 (1C), 129.4 (1C), 128.5 (2C), 127.6 (1C), 126.9 (1C), 126.0 (2C), 115.4 (1C), 115.2 (1C), 28.3 (1C), 10.0 (1C).

^{19}F NMR (376 MHz, $CDCl_3$) δ -110.83.

HRMS (EI) calculated for [M, $C_{16}H_{17}FSi]^+$: 256.1084, found: 256.1078.

(*E*)-(4-chlorophenyl)(4-phenylbut-3-en-1-yl)silane (3ag)

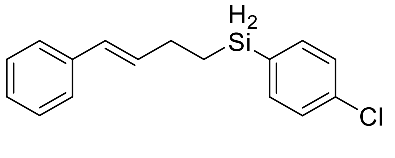

Serial number: sw-4-118, 185.2 mg, 97% yield, >98:2 r.r., colorless oil. R_f= 0.81 (PE).

^{1}H NMR (400 MHz, $CDCl_3$) δ 7.49 (d, J = 8.2 Hz, 2H), 7.33 (d, J = 8.1 Hz, 2H), 7.31 – 7.25 (m, 4H), 7.22 – 7.16 (m, 1H), 6.36 (d, J = 15.7 Hz, 1H), 6.21 (dt, J = 15.8, 6.6 Hz, 1H), 4.32 (t, J = 3.6 Hz, 2H), 2.41 – 2.27 (m, 2H), 1.18 – 1.06 (m, 2H).

^{13}C NMR (101 MHz, $CDCl_3$) δ 137.5 (1C), 136.6 (2C), 136.0 (1C), 131.8 (1C), 130.5 (1C),

129.4 (1C), 128.5 (2C), 128.3 (2C), 126.9 (1C), 125.9 (2C), 28.3 (1C), 9.8 (1C).

HRMS (EI) calculated for [M, $C_{16}H_{17}ClSi$]$^+$: 272.0788, found: 272.0783.

(*E*)-(4-methylbenzyl)(4-phenylbut-3-en-1-yl)silane (3ah)

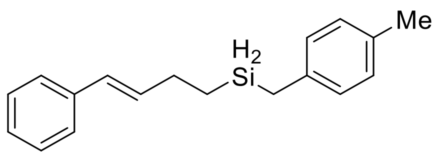

Serial number: sw-2-146, 178.8 mg, 96% yield, >98:2 r.r., colorless oil. R_f= 0.55 (PE).

^{1}H NMR (400 MHz, $CDCl_3$) δ 7.36 – 7.27 (m, 4H), 7.23 – 7.14 (m, 1H), 7.09 – 6.94 (m, 4H), 6.34 (d, *J* = 15.8 Hz, 1H), 6.20 (dt, *J* = 15.8, 6.6 Hz, 1H), 3.83 (p, *J* = 3.6 Hz, 2H), 2.32 – 2.24 (m, 5H), 2.22 (t, *J* = 3.7 Hz, 2H), 0.91 – 0.80 (m, 2H).

^{13}C NMR (101 MHz, $CDCl_3$) δ 137.7 (1C), 136.4 (1C), 134.0 (1C), 132.2 (1C), 129.2 (2C), 129.1 (1C), 128.5 (2C), 128.0 (2C), 126.9 (1C), 126.0 (2C), 28.5 (1C), 20.9 (1C), 18.4 (1C), 8.7 (1C).

HRMS (EI) calculated for [M, $C_{18}H_{22}Si$]$^+$: 266.1491, found: 266.1486.

(*E*)-octyl(4-phenylbut-3-en-1-yl)silane (3ai)

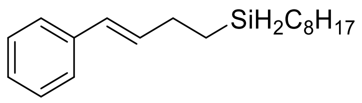

Serial number: sw-4-179, 175.3 mg, 91% yield, 98:2 r.r., colorless oil. R_f= 0.72 (PE).

^{1}H NMR (400 MHz, $CDCl_3$) δ 7.36 – 7.27 (m, 4H), 7.22 – 7.16 (m, 1H), 6.38 (d, *J* = 15.8 Hz, 1H), 6.26 (dt, *J* = 15.7, 6.5 Hz, 1H), 3.70 (p, *J* = 3.6 Hz, 2H), 2.37 – 2.25 (m, 2H), 1.42 – 1.25 (m, 12H), 0.92 – 0.85 (m, 5H), 0.76 – 0.67 (m, 2H).

^{13}C NMR (101 MHz, $CDCl_3$) δ 137.8 (1C), 132.6 (1C), 129.0 (1C), 128.5 (2C), 126.8 (1C), 126.0 (2C), 32.9 (1C), 31.9 (1C), 29.3 (1C), 29.2 (1C), 28.8 (1C), 25.4 (1C), 22.7 (1C), 14.1 (1C), 9.1 (1C), 9.0 (1C).

HRMS (EI) calculated for [M, $C_{18}H_{30}Si$]$^+$: 274.2117, found: 274.2112.

(*E*)-phenyl(4-(*o*-tolyl)but-3-en-1-yl)silane (3ba)

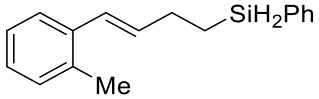

Serial number: sw-2-107, 170.4 mg, 96% yield, 98:2 r.r., colorless oil. R_f = 0.52 (PE).

<u>^{1}H NMR</u> (400 MHz, $CDCl_3$) δ 7.63 – 7.54 (m, 2H), 7.43 – 7.32 (m, 4H), 7.16 – 7.08 (m, 3H), 6.57 (d, *J* = 15.6 Hz, 1H), 6.11 (dt, *J* = 15.6, 6.7 Hz, 1H), 4.35 (t, *J* = 3.7 Hz, 2H), 2.44 – 2.35 (m, 2H), 2.31 (s, 3H), 1.19 – 1.11 (m, 2H).

<u>^{13}C NMR</u> (101 MHz, $CDCl_3$) δ 136.8 (1C), 135.3 (2C), 135.0 (1C), 133.5 (1C), 132.3 (1C), 130.1 (1C), 129.6 (1C), 128.0 (2C), 127.1 (1C), 126.8 (1C), 126.0 (1C), 125.5 (1C), 28.7 (1C), 19.8 (1C), 10.0 (1C).

<u>HRMS (EI)</u> calculated for [M, $C_{17}H_{20}Si]^+$: 252.1334, found: 252.1327.

(*E*)-phenyl(4-(3-(trifluoromethyl)phenyl)but-3-en-1-yl)silane (3ca)

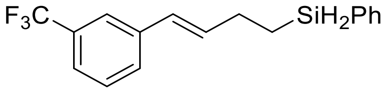

Serial number: sw-2-138, 196.8 mg, 92% yield, 98:2 r.r., colorless oil. R_f = 0.48 (PE).

<u>^{1}H NMR</u> (400 MHz, $CDCl_3$) δ 7.62 – 7.49 (m, 3H), 7.48 – 7.31 (m, 6H), 6.39 (d, *J* = 15.9 Hz, 1H), 6.30 (dt, *J* = 15.7, 6.2 Hz, 1H), 4.35 (t, *J* = 3.6 Hz, 2H), 2.39 (q, *J* = 7.1 Hz, 2H), 1.21 – 1.07 (m, 2H).

<u>^{13}C NMR</u> (101 MHz, $CDCl_3$) δ 138.4 (1C), 135.2 (2C), 134.2 (1C), 132.1 (1C), 130.9 (q, *J* = 32.1 Hz, 1C)., 129.7 (1C), 129.1 (d, *J* = 1.4 Hz, 1C), 128.8 (1C), 128.1 (3C), 124.2 (q, *J* = 270 Hz, 1C), 123.4 (q, *J* = 3.8 Hz, 1C), 122.6 (q, *J* = 4.0 Hz, 1C), 28.4 (1C), 9.7 (1C).

<u>^{19}F NMR</u> (376 MHz, $CDCl_3$) δ -62.73.

<u>HRMS (ESI)</u> calculated for [M-H, $C_{17}H_{16}F_3Si]^-$: 305.0979, found: 305.0965.

(*E*)-phenyl(4-(*p*-tolyl)but-3-en-1-yl)silane (3da)

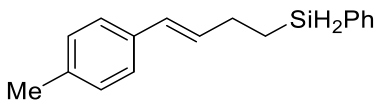

Serial number: sw-2-108, 172.1 mg, 98% yield, >98:2 r.r., colorless oil. R_f = 0.52 (PE).

^{1}H NMR (400 MHz, $CDCl_3$) δ 7.62 – 7.54 (m, 2H), 7.44 – 7.31 (m, 3H), 7.23 – 7.18 (m, 2H), 7.12 – 7.06 (m, 2H), 6.34 (d, J = 15.8 Hz, 1H), 6.19 (dt, J = 15.7, 6.6 Hz, 1H), 4.34 (t, J = 3.6 Hz, 2H), 2.39 – 2.33 (m, 2H), 2.32 (s, 3H), 1.17 – 1.07 (m, 2H).

^{13}C NMR (101 MHz, $CDCl_3$) δ 136.6 (1C), 135.3 (2C), 134.9 (1C), 132.3 (1C), 131.2 (1C), 129.6 (1C), 129.2 (2C), 129.1 (1C), 128.1 (2C), 125.9 (2C), 28.4 (1C), 21.2 (1C), 10.0 (1C).

HRMS (EI) calculated for [M, $C_{17}H_{20}Si$]$^+$: 252.1334, found: 252.1327.

(*E*)-(4-(4-methoxyphenyl)but-3-en-1-yl)(phenyl)silane (3ea)

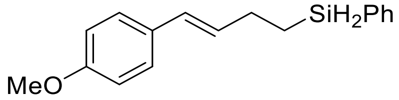

Serial number: sw-2-86, 178.2 mg, 95% yield, >98:2 r.r., colorless oil. R_f = 0.53 (PE/EA = 20:1, v/v).

^{1}H NMR (400 MHz, $CDCl_3$) δ 7.64 – 7.52 (m, 2H), 7.43 – 7.32 (m, 3H), 7.26 – 7.22 (m, 2H), 6.88 – 6.76 (m, 2H), 6.31 (d, J = 15.6, 1H), 6.10 (dt, J = 15.7, 6.7 Hz, 1H), 4.33 (t, J = 3.6 Hz, 2H), 3.79 (d, J = 1.4 Hz, 3H), 2.35 (dtd, J = 7.8, 6.4, 1.4 Hz, 2H), 1.18 – 1.05 (m, 2H).

^{13}C NMR (101 MHz, $CDCl_3$) δ 158.6 (1C), 135.2 (2C), 132.3 (1C), 130.5 (1C), 130.0 (1C), 129.6 (1C), 128.6 (1C), 128.0 (2C), 127.0 (2C), 113.9 (2C), 55.2 (1C), 28.3 (1C), 10.0 (1C).

HRMS (EI) calculated for [M, $C_{17}H_{20}OSi$]$^+$: 268.1283, found 268.1278.

(*E*)-N,N-dimethyl-4-(4-(phenylsilyl)but-1-en-1-yl)aniline (3fa)

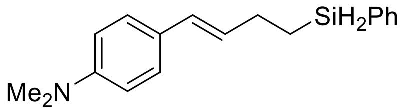

Serial number: sw-2-160, 193 mg, 98% yield, >98:2 r.r., yellow oil. R_f= 0.24 (PE).

^{1}H NMR (400 MHz, $CDCl_3$) δ 7.62 – 7.52 (m, 2H), 7.42 – 7.31 (m, 3H), 7.24 – 7.15 (m, 2H), 6.70 – 6.62 (m, 2H), 6.28 (d, J = 15.7 Hz, 1H), 6.04 (dt, J = 15.9, 6.7 Hz, 1H), 4.33 (t, J = 3.5 Hz, 2H), 2.92 (s, 6H), 2.39 – 2.28 (m, 2H), 1.16 – 1.07 (m, 2H).

^{13}C NMR (101 MHz, $CDCl_3$) δ 149.7 (1C), 135.3 (2C), 132.5 (1C), 129.5 (1C), 129.0 (1C), 128.0 (3C), 126.8 (2C), 126.4 (1C), 112.6 (2C), 40.6 (2C), 28.4 (1C), 10.2 (1C).

HRMS (EI) calculated for [M, $C_{18}H_{23}NSi]^+$: 281.1600, found: 281.1592.

(*E*)-(4-(4-fluorophenyl)but-3-en-1-yl)(phenyl)silane (3ga)

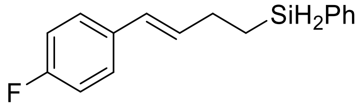

Serial number: sw-2-99, 177.2 mg, 99% yield, >98:2 r.r., colorless oil. R_f= 0.59 (PE).

^{1}H NMR (400 MHz, $CDCl_3$) δ 7.63 – 7.53 (m, 2H), 7.46 – 7.30 (m, 3H), 7.29 – 7.21 (m, 2H), 7.03 – 6.88 (m, 2H), 6.32 (d, J = 15.7 Hz, 1H), 6.14 (dt, J = 15.8, 6.6 Hz, 1H), 4.34 (t, J = 3.6 Hz, 2H), 2.41 – 2.30 (m, 2H), 1.18 – 1.07 (m, 2H).

^{13}C NMR (101 MHz, $CDCl_3$) δ 161.9 (J = 244.1 Hz, 1C), 135.2 (2C), 133.8 (1C), 132.2 (1C), 131.9 (1C), 129.6 (1C), 128.1 (1C), 128.0 (2C), 127.4 (1C), 127.3 (1C), 115.4 (1C), 115.2 (1C), 28.3 (1C), 9.9 (1C).

^{19}F NMR (376 MHz, $CDCl_3$) δ -115.71.

HRMS (EI) calculated for [M, $C_{16}H_{17}FSi]^+$: 256.1084, found: 256.1078.

(*E*)-(4-(4-chlorophenyl)but-3-en-1-yl)(phenyl)silane (3ha)

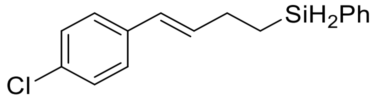

Serial number: sw-2-126, 186.9 mg, 98% yield, >98:2 r.r., colorless oil. R_f= 0.63 (PE).

^{1}H NMR (400 MHz, $CDCl_3$) δ 7.62 – 7.52 (m, 2H), 7.45 – 7.31 (m, 3H), 7.25 – 7.18 (m, 4H), 6.32 (d, J = 15.8, 1H), 6.21 (dt, J = 15.8, 6.4 Hz, 1H), 4.34 (t, J = 3.6 Hz, 2H), 2.44 – 2.29 (m, 2H), 1.18 – 1.08 (m, 2H).

^{13}C NMR (101 MHz, $CDCl_3$) δ 136.2 (1C), 135.2 (2C), 132.9 (1C), 132.4(1C), 132.1 (1C), 129.6 (1C), 128.6 (2C), 128.1 (1C), 128.0 (2C), 127.2 (2C), 28.4 (1C), 9.8 (1C).

HRMS (EI) calculated for [M, $C_{16}H_{17}ClSi]^+$: 272.0788, found: 272.0783.

(*E*)-(4-(naphthalen-2-yl)but-3-en-1-yl)(phenyl)silane (3ia)

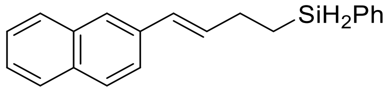

Serial number: sw-2-148, 199 mg, 99% yield, 98:2 r.r., colorless oil. R_f= 0.50 (PE).

^{1}H NMR (400 MHz, $CDCl_3$) δ 7.81 – 7.71 (m, 3H), 7.66 – 7.57 (m, 3H), 7.53 (dd, *J* = 8.5, 1.7 Hz, 1H), 7.47 – 7.32 (m, 5H), 6.53 (d, *J* = 15.7 Hz, 1H), 6.37 (dt, *J* = 15.7, 6.6 Hz, 1H), 4.37 (t, *J* = 3.6 Hz, 2H), 2.49 – 2.35 (m, 2H), 1.23 – 1.11 (m, 2H).

^{13}C NMR (101 MHz, $CDCl_3$) δ 135.3 (2C), 135.1 (1C), 133.7 (1C), 132.7 (1C), 132.6 (1C), 132.2 (1C), 129.6 (1C), 129.4 (1C), 128.0 (3C), 127.8 (1C), 127.6 (1C), 126.1 (1C), 125.4 (2C), 123.6 (1C), 28.5 (1C), 9.9 (1C).

HRMS (EI) calculated for [M, $C_{20}H_{20}Si]^+$: 288.1334, found: 288.1329.

(*E*)-(4-(benzo[d][1,3]dioxol-5-yl)but-3-en-1-yl)(phenyl)silane (3ja)

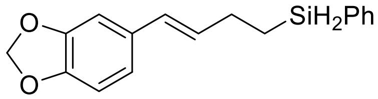

Serial number: sw-3-8, 196.2 mg, 99% yield, 98:2 r.r., colorless oil. R_f= 0.53 (PE/EA = 10:1, v/v).

^{1}H NMR (400 MHz, $CDCl_3$) δ 7.63 – 7.52 (m, 2H), 7.45 – 7.31 (m, 3H), 6.88 – 6.82 (m, 1H), 6.76 – 6.68 (m, 2H), 6.28 (d, *J* = 15.7, 1H), 6.07 (dt, *J* = 15.8, 6.8 Hz, 1H), 5.93 (s, 2H), 4.38 – 4.29 (m, 2H), 2.39 – 2.27 (m, 2H), 1.17 – 1.06 (m, 2H).

^{13}C NMR (101 MHz, $CDCl_3$) δ 147.9 (1C), 146.6 (1C), 135.2 (2C), 132.2 (2C), 130.4 (1C), 129.6 (1C), 128.8 (1C), 128.0 (2C), 120.3 (1C), 108.2 (1C), 105.4 (1C), 100.9 (1C), 28.3 (1C), 10.0 (1C).

HRMS (EI) calculated for [M, $C_{17}H_{18}O_2Si]^+$: 282.1076, found: 282.1069.

(*E*)-(4-(furan-2-yl)but-3-en-1-yl)(phenyl)silane (3ka)

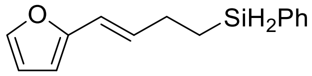

Serial number: sw-2-161, 148 mg, 93% yield, 97:3 r.r., colorless oil. R_f= 0.73 (PE).

^{1}H NMR (400 MHz, $CDCl_3$) δ 7.60 – 7.52 (m, 2H), 7.43 – 7.27 (m, 4H), 6.35 – 6.30 (m, 1H), 6.22 – 6.17 (m, 2H), 6.14 – 6.09 (m, 1H), 4.33 (t, *J* = 3.7 Hz, 2H), 2.39 – 2.27 (m, 2H), 1.17 – 1.05 (m, 2H).

^{13}C NMR (101 MHz, $CDCl_3$) δ 153.2 (1C), 141.3 (1C), 135.2 (2C), 132.2 (1C), 131.2 (1C), 129.6 (1C), 128.0 (2C), 118.0 (1C), 111.1 (1C), 106.2 (1C), 28.1 (1C), 9.7 (1C).

HRMS (EI) calculated for [M, $C_{14}H_{16}OSi$]$^+$: 228.0970, found 228.0966.

(*E*)-phenyl(4-phenylpent-3-en-1-yl)silane (3la)

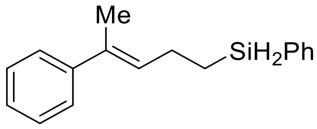

Serial number: sw-2-202, 167.0 mg, 95% yield, >98:2 r.r., colorless oil. R_f= 0.61 (PE).

^{1}H NMR (400 MHz, $CDCl_3$) δ 7.62 – 7.55 (m, 2H), 7.43 – 7.27 (m, 7H), 7.24 – 7.18 (m, 1H), 5.83 – 5.74 (m, 1H), 4.34 (t, *J* = 3.6, 2H), 2.42 – 2.30 (m, 2H), 1.98 (s, 3H), 1.18 – 1.07 (m, 2H).

^{13}C NMR (101 MHz, $CDCl_3$) δ 143.8 (1C), 135.2 (2C), 134.3 (1C), 132.4 (1C), 129.9 (1C), 129.6 (1C), 128.1 (2C), 128.0 (2C), 126.5 (1C), 125.6 (2C), 24.1 (1C), 15.8 (1C), 10.3 (1C).

HRMS (EI) calculated for [M, $C_{17}H_{20}Si$]$^+$: 252.1334, found: 252.1329.

(4,4-diphenylbut-3-en-1-yl)(phenyl)silane (3ma)

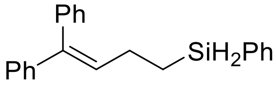

Serial number: sw-3-190, 191.7 mg, 87% yield, >98:2 r.r., colorless oil. Complex **C1b** was used as the catalyst. R_f= 0.29 (PE).

^{1}H NMR (400 MHz, $CDCl_3$) δ 7.52 – 7.46 (m, 2H), 7.40 – 7.11 (m, 13H), 6.08 (t, *J* = 7.4 Hz, 1H), 4.28 (t, *J* = 3.7 Hz, 2H), 2.29 (q, *J* = 7.7 Hz, 2H), 1.15 – 1.03 (m, 2H).

^{13}C NMR (101 MHz, $CDCl_3$) δ 142.7 (1C), 141.1 (1C), 140.0 (1C), 135.2 (2C), 132.3 (1C), 131.3 (1C), 129.9 (2C), 129.6 (1C), 128.2 (2C), 128.1 (2C), 128.0 (2C), 127.3 (2C), 126.9 (1C), 126.9 (1C), 25.2 (1C), 10.7 (1C).

HRMS (EI) calculated for [M, $C_{22}H_{22}Si]^+$: 314.1491, found: 314.1481.

(*E*)-(3-methyl-4-phenylbut-3-en-1-yl)(phenyl)silane (3na)[13]

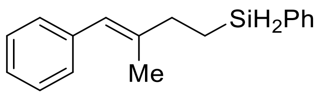

Serial number: sw-2-162, 176.2 mg, 99% yield, >98:2 r.r., colorless oil. R_f= 0.69 (PE).

^{1}H NMR (400 MHz, $CDCl_3$) δ 7.62 – 7.55 (m, 2H), 7.43 – 7.25 (m, 5H), 7.24 – 7.13 (m, 3H), 6.29 (s, 1H), 4.34 (t, *J* = 3.6 Hz, 2H), 2.36 – 2.26 (m, 2H), 1.84 (s, 3H), 1.24 – 1.12 (m, 2H).

^{13}C NMR (101 MHz, $CDCl_3$) δ 140.2 (1C), 138.5 (1C), 135.3 (2C), 132.4 (1C), 129.6 (1C), 128.8 (2C), 128.0 (4C), 125.9 (1C), 124.4 (1C), 35.8 (1C), 17.5 (1C), 8.7 (1C).

HRMS (EI) calculated for [M, $C_{17}H_{20}Si]^+$: 252.1334, found: 252.1328.

(*Z*)-phenyl(3-phenylpent-3-en-1-yl)silane (3oa)

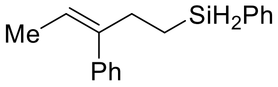

Serial number: llj-1-106, 172.9 mg, 98% yield, 98:2 r.r., colorless oil. Complex **C1b** (2 mol%) was used as the catalyst. R_f= 0.59 (PE).

^{1}H NMR (400 MHz, $CDCl_3$) 7.56 – 7.48 (m, 2H), 7.43 – 7.28 (m, 5H), 7.22 (d, *J* = 7.3 Hz, 1H), 7.15 – 7.08 (m, 2H), 5.59 – 5.51 (m, 1H), 4.26 (t, *J* = 3.7 Hz, 2H), 2.53 – 2.43 (m, 2H), 1.54 (d, *J* = 6.9 Hz, 3H), 1.04 – 0.90 (m, 2H).

^{13}C NMR (101 MHz, $CDCl_3$) δ 142.8 (1C), 140.6 (1C), 135.2 (1C), 132.4 (1C), 129.5 (1C), 128.6 (1C), 128.0 (1C), 127.9 (1C), 126.4 (1C), 120.6 (1C), 34.4 (1C), 14.6 (1C), 8.9

(1C).

HRMS (EI) calculated for [M, $C_{17}H_{20}Si$]$^+$: 252.1334, found: 252.1328.

(*E*)-non-3-en-1-yl(phenyl)silane (3pa)[13]

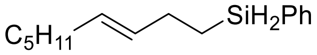

Serial number: sw-4-79A, 156.7 mg, 96% yield, 1,2/1,4 > 98:2 (the 1,2/1,4 refers to the ratio of 1,2- and 1,4-*anti*-Markovnikov hydrosilylation product), colorless oil. Complex **C1e** was used as the catalyst; EtMgBr (4 mol%) was added at −30 °C, and then the mixture was stirred at 0 °C for 10 h. R_f= 0.85 (PE).

^{1}H NMR (400 MHz, $CDCl_3$) δ 7.60 – 7.52 (m, 2H), 7.43 – 7.31 (m, 3H), 5.50 – 5.34 (m, 2H), 4.29 (t, *J* = 3.7 Hz, 2H), 2.15 (td, *J* = 7.6, 5.3 Hz, 2H), 1.96 (q, *J* = 6.6 Hz, 2H), 1.37 – 1.20 (m, 6H), 1.07 – 0.97 (m, 2H), 0.88 (t, *J* = 6.8 Hz, 3H).

^{13}C NMR (101 MHz, $CDCl_3$) δ 135.3 (2C), 132.6 (1C), 131.5 (1C), 130.1 (1C), 129.5 (1C), 128.0 (2C), 32.4 (1C), 31.4 (1C), 29.3 (1C), 27.9 (1C), 22.6 (1C), 14.1 (1C), 10.1 (1C).

(*E*)-(4-cyclohexylbut-3-en-1-yl)(phenyl)silane (3qa)[13]

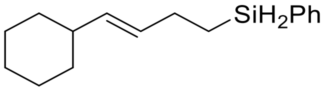

Serial number: sw-4-79B, 164.0 mg, 96% yield, 1,2/1,4 > 98:2 (the 1,2/1,4 refers to the ratio of 1,2- and 1,4-*anti*-Markovnikov hydrosilylation product), colorless oil. Complex **C1e** was used as the catalyst; EtMgBr (4 mol%) was added at −30 °C, and then the mixture was stirred at 0 °C for 10 h. R_f= 0.86 (PE).

^{1}H NMR (400 MHz, $CDCl_3$) δ 7.61 – 7.51 (m, 2H), 7.42 – 7.30 (m, 3H), 5.46 – 5.29 (m, 2H), 4.28 (t, *J* = 3.7 Hz, 2H), 2.18 – 2.09 (m, 2H), 1.94 – 1.81 (m, 1H), 1.74 – 1.58 (m, 5H), 1.29 – 0.98 (m, 7H).

^{13}C NMR (101 MHz, $CDCl_3$) δ 136.0 (1C), 135.3 (2C), 132.6 (1C), 129.5 (1C), 128.9 (1C), 128.0 (2C), 40.6 (1C), 33.2 (2C), 28.0 (1C), 26.3 (1C), 26.1 (2C), 10.1 (1C).

(4-(*tert*-butyl)phenyl)(3-methylbut-3-en-1-yl)silane (3re)

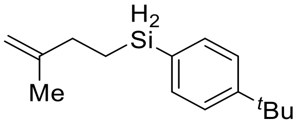

Serial number: sw-3-167, 154.1 mg, 95% yield, 1,2/1,4 = 94:6 (the 1,2/1,4 refers to the ratio of 1,2- and 1,4-*anti*-Markovnikov hydrosilylation product), colorless oil. Silane **2e** was used instead of **2a**. R_f= 0.56 (PE).

^{1}H NMR (400 MHz, $CDCl_3$) δ 7.54 – 7.49 (m, 2H), 7.42 – 7.37 (m, 2H), 4.75 – 4.68 (m, 2H), 4.29 (t, *J* = 3.7 Hz, 2H), 2.20 – 2.10 (m, 2H), 1.73 (s, 3H), 1.32 (s, 9H), 1.13 – 1.02 (m, 2H).

^{13}C NMR (101 MHz, $CDCl_3$) δ 152.7 (1C), 147.4 (1C), 135.2 (2C), 128.8 (1C), 125.0 (2C), 109.1 (1C), 34.7 (1C), 33.0 (1C), 31.2 (3C), 22.2 (1C), 8.3 (1C).

HRMS (EI) calculated for [M, $C_{15}H_{24}Si]^+$: 232.1647, found: 232.1641.

(7-methyl-3-methyleneoct-6-en-1-yl)(phenyl)silane (3sa)[13]

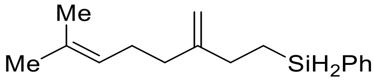

Serial number: sw-4-79C, 153.7 mg, 90% yield, 1,2/1,4 = 93:7 (the 1,2/1,4 refers to the ratio of 1,2- and 1,4-*anti*-Markovnikov hydrosilylation product), colorless oil. Complex **C1d** was used as the catalyst; EtMgBr (4 mol%) was added at −30 °C, and then the mixture was stirred at 0 °C for 10 h. R_f= 0.61 (PE).

^{1}H NMR (400 MHz, $CDCl_3$) δ 7.61 – 7.54 (m, 2H), 7.43 – 7.32 (m, 3H), 5.14 – 5.04 (m, 1H), 4.77 (s, 1H), 4.73 (s, 1H), 4.30 (t, *J* = 3.6 Hz, 2H), 2.18 – 2.00 (m, 6H), 1.68 (s, 3H), 1.60 (s, 3H), 1.14 – 1.03 (m, 2H).

^{13}C NMR (101 MHz, $CDCl_3$) δ 151.0 (1C), 135.2 (2C), 132.4 (1C), 131.6 (1C), 129.6 (1C), 128.0 (2C), 124.1 (1C), 108.3 (1C), 35.8 (1C), 31.4 (1C), 26.4 (1C), 25.7 (1C), 17.7 (1C), 8.3 (1C).

(3-cyclohexylidenepropyl)(phenyl)silane (3ta)

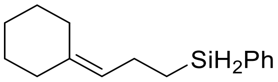

Serial number: sw-3-147, 155.7 mg, 97% yield, 1,2/1,4 = 92:8 (the 1,2/1,4 refers to the ratio of 1,2- and 1,4-*anti*-Markovnikov hydrosilylation product), colorless oil. R_f= 0.64 (PE).

^{1}H NMR (400 MHz, $CDCl_3$) δ 7.61 – 7.52 (m, 2H), 7.42 – 7.30 (m, 3H), 5.10 (t, *J* = 7.1 Hz, 1H), 4.28 (t, *J* = 3.7 Hz, 2H), 2.15 (q, *J* = 7.5 Hz, 2H), 2.11 – 1.99 (m, 4H), 1.58 – 1.41 (m, 6H), 1.06 – 0.94 (m, 2H).

^{13}C NMR (101 MHz, $CDCl_3$) δ 139.2 (1C), 135.2 (2C), 132.7 (1C) 129.5 (1C), 127.9 (2C), 122.9 (1C), 37.1 (1C), 28.7 (1C), 28.6 (1C), 27.7 (1C), 27.0 (1C), 22.5 (1C), 10.9 (1C).

HRMS (EI) calculated for [M, $C_{15}H_{22}Si$]$^+$: 230.1491, found: 230.1488.

(*E*)-(4,8-dimethylnona-3,7-dien-1-yl)(phenyl)silane (3ua)

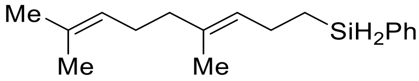

Serial number: sw-3-75, 172.3 mg, 95% yield, 1,2/1,4 = 97:3 (the 1,2/1,4 refers to the ratio of 1,2- and 1,4-*anti*-Markovnikov hydrosilylation product),colorless oil. R_f= 0.81 (PE).

^{1}H NMR (400 MHz, $CDCl_3$) δ 7.60 – 7.53 (m, 2H), 7.42 – 7.31 (m, 3H), 5.16 (tq, *J* = 7.1, 1.4 Hz, 1H), 5.10 (dtd, *J* = 6.7, 5.2, 4.1, 2.6 Hz, 1H), 4.29 (t, *J* = 3.7 Hz, 2H), 2.15 (q, *J* = 7.5 Hz, 2H), 2.09 – 2.00 (m, 2H), 1.99 – 1.92 (m, 2H), 1.68 (s, 3H), 1.60 (s, 3H), 1.56 (s, 3H), 1.05 – 0.96 (m, 2H).

^{13}C NMR (101 MHz, $CDCl_3$) δ 135.2 (2C), 134.7 (1C), 132.7 (1C), 131.3 (1C), 129.5 (1C), 128.0 (2C), 126.1 (1C), 124.4 (1C), 39.7 (1C), 26.7 (1C), 25.7 (1C), 23.2 (1C), 17.7 (1C), 16.0 (1C), 10.5 (1C).

HRMS (EI) calculated for [M, $C_{17}H_{26}Si$]$^+$: 258.1804, found: 258.1793.

((3*E*,5*E*)-4-methyl-6-(2,6,6-trimethylcyclohex-1-en-1-yl)hexa-3,5-dien-1-yl)(phenyl)silane (3va)

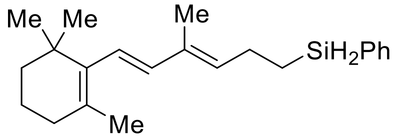

Serial number: llj-1-19, 182.0 mg, 80% yield, 1,2/1,4 > 98:2 (the 1,2/1,4 refers to the ratio of 1,2- and 1,4-*anti*-Markovnikov hydrosilylation product), colorless oil. R_f= 0.44 (PE).

^{1}H NMR (400 MHz, $CDCl_3$) δ 7.60 – 7.53 (m, 2H), 7.43 – 7.31 (m, 3H), 5.97 (s, 2H), 5.43 (td, *J* = 7.3, 1.4 Hz, 1H), 4.31 (t, *J* = 3.7 Hz, 2H), 2.30 (q, *J* = 7.6 Hz, 2H), 1.99 (t, *J* = 6.3 Hz, 2H), 1.74 (s, 3H), 1.68 (s, 3H), 1.64 – 1.57 (m, 2H), 1.48 – 1.43 (m, 2H), 1.11 – 1.02 (m, 2H), 1.00 (s, 6H).

^{13}C NMR (101 MHz, $CDCl_3$) δ 137.9 (1C), 137.8 (1C), 135.3 (2C), 133.6 (1C), 132.6 (1C), 132.4 (1C), 129.6 (1C), 128.3 (1C), 128.0 (2C), 124.5 (1C), 39.6 (1C), 34.2 (1C), 32.9 (1C), 28.9 (2C), 23.6 (1C), 21.7 (1C), 19.3 (1C), 12.3 (1C), 10.4 (1C).

HRMS (EI) calculated for [M, $C_{22}H_{32}Si$]$^+$: 324.2273, found: 324.2266.

(*S*)-phenyl(2-(4-(prop-1-en-2-yl)cyclohex-1-en-1-yl)ethyl)silane (3wa)[13]

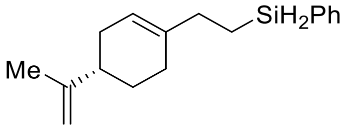

Serial number: sw-3-10, 170.0 mg, 95% yield, 1,2/1,4 > 98:2 (the 1,2/1,4 refers to the ratio of 1,2- and 1,4-*anti*-Markovnikov hydrosilylation product), colorless oil. R_f= 0.78 (PE).

^{1}H NMR (400 MHz, $CDCl_3$) δ 7.61 – 7.51 (m, 2H), 7.43 – 7.30 (m, 3H), 5.43 (s, 1H), 4.74 – 4.65 (m, 2H), 4.28 (t, *J* = 3.6 Hz, 2H), 2.13 – 1.75 (m, 8H), 1.72 (s, 3H), 1.47 – 1.33 (m, 1H), 1.12 – 1.01 (m, 2H).

^{13}C NMR (101 MHz, $CDCl_3$) δ 150.3 (1C), 138.6 (1C), 135.2 (2C), 132.6 (1C), 129.5 (1C), 128.0 (2C), 120.0 (1C), 108.4 (1C), 41.2 (1C), 32.7 (1C), 30.7 (1C), 28.6 (1C), 27.8 (1C), 20.8 (1C), 8.2 (1C).

HRMS (EI) calculated for [M, $C_{17}H_{24}Si$]$^+$: 256.1647, found: 256.1653.

(2-(cyclohept-1-en-1-yl)ethyl)(phenyl)silane (3xa)[13]

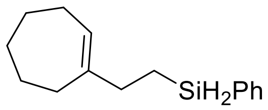

Serial number: sw-4-60, 156.1 mg, 97% yield, 1,2/1,4 = 96:4 (the 1,2/1,4 refers to the ratio of 1,2- and 1,4-*anti*-Markovnikov hydrosilylation product), colorless oil. R_f= 0.68 (PE).

^{1}H NMR (400 MHz, $CDCl_3$) δ 7.63 – 7.50 (m, 2H), 7.44 – 7.31 (m, 3H), 5.55 (t, J = 6.5 Hz, 1H), 4.28 (t, J = 3.7 Hz, 2H), 2.14 – 2.01 (m, 6H), 1.71 (p, J = 5.9 Hz, 2H), 1.50 – 1.40 (m, 4H), 1.09 – 0.98 (m, 2H).

^{13}C NMR (101 MHz, $CDCl_3$) δ 145.9 (1C), 135.2 (2C), 132.7 (1C), 129.5 (1C), 127.9 (2C), 125.1 (1C), 35.2 (1C), 32.6 (2C), 28.2 (1C), 27.3 (1C), 26.9 (1C), 8.6 (1C).

phenethyl(phenyl)silane (8aa)[14]

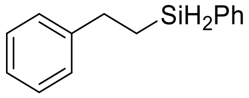

Serial number: lmp-1-109, 147 mg, 99% yield, colorless oil. R_f= 0.64 (PE).

^{1}H NMR (400 MHz, $CDCl_3$) δ 7.59 – 7.52 (m, 2H), 7.43 – 7.31 (m, 3H), 7.30 – 7.23 (m, 2H), 7.22 – 7.13 (m, 3H), 4.32 (t, J = 3.6 Hz, 2H), 2.80 – 2.72 (m, 2H), 1.33 – 1.25 (m, 2H).

^{13}C NMR (101 MHz, $CDCl_3$) δ 143.9 (1C), 135.2 (2C), 132.1 (1C), 129.6 (1C), 128.3 (2C), 128.0 (2C), 127.9 (2C), 125.8 (1C), 31.1 (1C), 12.1 (1C).

(3-methylphenethyl)(phenyl)silane (8ba)[3]

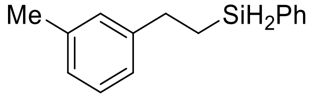

Serial number: sw-3-183, 150.0 mg, 95% yield, colorless oil. R_f= 0.32 (PE).

^{1}H NMR (400 MHz, $CDCl_3$) δ 7.60 – 7.52 (m, 2H), 7.44 – 7.31 (m, 3H), 7.21 – 7.12 (m, 1H), 7.02 – 6.95 (m, 3H), 4.31 (t, J = 3.6 Hz, 2H), 2.77 – 2.68 (m, 2H), 2.31 (s, 3H), 1.33

– 1.24 (m, 2H).

<u>^{13}C NMR</u> (101 MHz, $CDCl_3$) δ 143.9 (1C), 137.9 (1C), 135.2 (2C), 132.2 (1C), 129.6 (1C), 128.7 (1C), 128.2 (1C), 128.0 (2C), 126.5 (1C), 124.9 (1C), 31.0 (1C), 21.4 (1C), 12.1 (1C).

(4-fluorophenethyl)(phenyl)silane (8ca)[14]

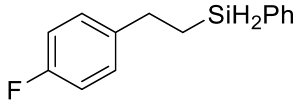

Serial number: lmp-2-99, 159 mg, 99% yield, colorless oil. R_f= 0.75 (PE)

<u>^{1}H NMR</u> (400 MHz, $CDCl_3$) δ 7.60 – 7.51 (m, 2H), 7.44 – 7.31 (m, 3H), 7.16 – 7.06 (m, 2H), 7.00 – 6.89 (m, 2H), 4.30 (t, J = 3.6 Hz, 2H), 2.78 – 2.68 (m, 2H), 1.32 – 1.22 (m, 2H).

<u>^{13}C NMR</u> (101 MHz, $CDCl_3$) δ 161.2 (J = 242.4 Hz, 1C), 139.5 (1C), 135.2 (2C), 132.0 (1C), 129.7 (1C), 129.2 (2C), 128.0 (2C), 115.1 (1C), 114.9 (1C), 30.3 (1C), 12.3 (1C).

(4-methoxyphenethyl)(phenyl)silane(8da)[14]

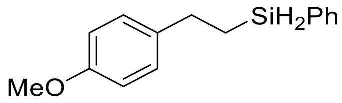

Serial number: lmp-4-87, 163.0 mg, 96% yield, colorless oil. R_f= 0.61 (PE/EA = 20:1, *v*/*v*).

<u>^{1}H NMR</u> (400 MHz, $CDCl_3$) δ 7.59 – 7.54 (m, 2H), 7.41 – 7.33 (m, 3H), 7.13 – 7.08 (m, 2H), 6.84 – 6.79 (m, 2H), 4.30 (t, J = 3.6 Hz, 2H), 3.79 (s, 3H), 2.75 – 2.68 (m, 2H), 1.31 – 1.23 (m, 2H).

<u>^{13}C NMR</u> (101 MHz, $CDCl_3$) δ 157.7 (1C), 136.0 (1C), 135.2 (2C), 132.2 (1C), 129.6 (1C), 128.8 (2C), 128.0 (2C), 113.7 (2C), 55.2 (1C), 30.2 (1C), 12.3 (1C).

(4-(*tert*-butyl)phenethyl)(phenyl)silane (8ea)[15]

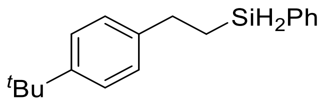

Serial number: sw-3-175, 178.6 mg, 95% yield, colorless oil. R_f= 0.45 (PE).

1H NMR (400 MHz, $CDCl_3$) δ 7.58 – 7.51 (m, 2H), 7.42 – 7.32 (m, 3H), 7.32 – 7.27 (m, 2H), 7.15 – 7.09 (m, 2H), 4.32 (t, *J* = 3.6 Hz, 2H), 2.78 – 2.69 (m, 2H), 1.33 – 1.26 (m, 11H).

13C NMR (101 MHz, $CDCl_3$) δ 148.6 (1C), 141.0 (1C), 135.3 (2C), 132.3 (1C), 129.7 (1C), 128.1 (2C), 127.6 (2C), 125.3 (2C), 34.4 (1C), 31.5 (3C), 30.6 (1C), 12.0 (1C).

(2-([1,1'-biphenyl]-4-yl)ethyl)(phenyl)silane (8fa)

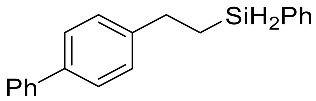

Serial number: sw-3-176, 194.2 mg, 96% yield, colorless oil. R_f= 0.50 (PE).

1H NMR (400 MHz, $CDCl_3$) δ 7.62 – 7.54 (m, 4H), 7.53 – 7.47 (m, 2H), 7.46 – 7.29 (m, 6H), 7.28 – 7.23 (m, 2H), 4.35 (t, *J* = 3.6 Hz, 2H), 2.87 – 2.74 (m, 2H), 1.39 – 1.29(m, 2H).

13C NMR (101 MHz, $CDCl_3$) δ 143.2 (1C), 141.2 (1C), 138.9 (1C), 135.3 (2C), 132.2 (1C), 129.7 (1C), 128.8 (2C), 128.4 (2C), 128.1 (2C), 127.2 (2C), 127.1 (3C), 30.8 (1C), 12.2 (1C).

HRMS (EI) calculated for [M, $C_{20}H_{20}Si]^+$: 288.1334, found: 288.1329.

phenyl(6-phenylhexyl)silane (8ga)

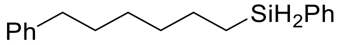

Serial number: lmp-4-68, 176.3 mg, 94% yield, colorless oil. R_f= 0.41 (PE).

1H NMR (400 MHz, $CDCl_3$) δ 7.59 – 7.53 (m, 2H), 7.42 – 7.32 (m, 3H), 7.30 – 7.24 (m, 2H), 7.17 (m, 3H), 4.28 (t, *J* = 3.7 Hz, 2H), 2.63 – 2.54 (m, 2H), 1.65 – 1.55 (m, 2H), 1.51 – 1.28 (m, 6H), 0.97 – 0.88 (m, 2H).

13C NMR (101 MHz, $CDCl_3$) δ 142.8 (1C), 135.2 (2C), 132.7 (1C), 129.5 (1C), 128.4 (2C), 128.2 (2C), 127.9 (2C), 125.5 (1C), 35.9 (1C), 32.6 (1C), 31.3 (1C), 28.9 (1C), 25.0 (1C), 10.0 (1C).

HRMS (EI) calculated for [M, $C_{18}H_{24}Si]^+$: 268.1647, found: 268.1642.

(6-chlorohexyl)(phenyl)silane (8ha)[3]

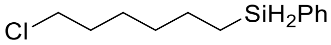

Serial number: lmp-4-6, 133.3 mg, 84% yield, colorless oil. R_f= 0.72 (PE).

^{1}H NMR (400 MHz, $CDCl_3$) δ 7.62 – 7.52 (m, 2H), 7.43 – 7.33 (m, 3H), 4.29 (t, J = 3.7 Hz, 2H), 3.52 (t, J = 6.7 Hz, 2H), 1.75 (p, J = 6.7 Hz, 2H), 1.53 – 1.34 (m, 6H), 0.98 – 0.91 (m, 2H).

^{13}C NMR (101 MHz, $CDCl_3$) δ 135.2 (2C), 132.6 (1C), 129.5 (1C), 127.9 (2C), 45.1 (1C), 32.5 (1C), 32.0 (1C), 26.5 (1C), 24.9 (1C), 9.9 (1C).

1-(6-(phenylsilyl)hexyl)piperidine (8ia)

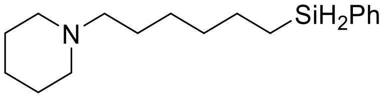

Serial number: lmp-4-1, 162.0 mg, 84% yield, colorless oil. R_f= 0.15 (PE/EA = 1:1, *v*/*v*).

^{1}H NMR (400 MHz, $CDCl_3$) δ 7.59 – 7.53 (m, 2H), 7.41 – 7.31 (m, 3H), 4.27 (t, J = 3.7 Hz, 2H), 2.34 (br, 4H), 2.28 – 2.21 (m, 2H), 1.58 (p, J = 5.7 Hz, 4H), 1.50 – 1.34 (m, 8H), 1.30 – 1.23 (m, 2H), 0.96 – 0.89 (m, 2H).

^{13}C NMR (101 MHz, $CDCl_3$) δ 135.1 (2C), 132.6 (1C), 129.4 (1C), 127.9 (2C), 59.6 (1C), 54.6 (2C), 32.7 (1C), 27.3 (1C), 26.8 (1C), 26.0 (2C), 24.9 (1C), 24.5 (1C), 9.9 (1C).

HRMS (EI) calcd for [M, $C_{17}H_{29}NSi]^+$: 275.2069, found: 275.2063.

4-(6-(phenylsilyl)hexyl)morpholine (8ja)[3]

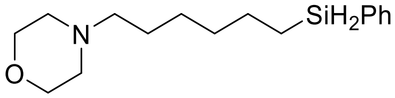

Serial number: lmp-4-22, 169 mg, 87% yield, yellow oil. R_f= 0.33 (PE/EA = 1:1, *v*/*v*)

^{1}H NMR (400 MHz, $CDCl_3$) δ 7.63 – 7.50 (m, 2H), 7.42 – 7.32 (m, 3H), 4.28 (t, J = 3.7 Hz, 2H), 3.71 (t, J = 4.7 Hz, 4H), 2.48 – 2.26 (m, 6H), 1.50 – 1.24 (m, 8H), 0.99 – 0.87

(m, 2H).

<u>13C NMR</u> (101 MHz, $CDCl_3$) δ 135.2 (2C), 132.7 (1C), 129.5 (1C), 127.9 (2C), 67.0 (2C), 59.2 (1C), 53.8 (2C), 32.7 (1C), 27.1 (1C), 26.4 (1C), 25.0 (1C), 9.9 (1C).

N,N-dimethyl-3-(phenylsilyl)propan-1-amine (8ka)[16]

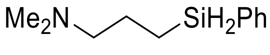

Serial number: sw-4-80, 108.1 mg, 80% yield, colorless oil. The reaction mixture was directly desolvated by vacuum under reduced pressure, and the resulting crude product was distilled under reduced pressure using a high vacuum pump, and the fraction was collected as the pure product.

<u>1H NMR</u> (400 MHz, $CDCl_3$) δ 7.59 – 7.53 (m, 2H), 7.42 – 7.31 (m, 3H), 4.31 (t, *J* = 3.7 Hz, 2H), 2.31 – 2.25 (m, 2H), 2.19 (s, 6H), 1.67 – 1.57 (m, 2H), 0.98 – 0.91 (m, 2H).

<u>13C NMR</u> (101 MHz, $CDCl_3$) δ 135.1 (2C), 133.0 (1C), 129.4 (1C), 127.9 (2C), 62.2 (1C), 45.4 (2C), 23.2 (1C), 7.8 (1C).

(2-phenoxyethyl)(phenyl)silane (8la)

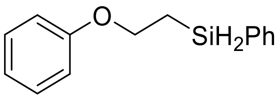

Serial number: sw-4-55, 153.2 mg, 96% yield, colorless oil. R_f = 0.61 (PE/EA = 20:1, *v*/*v*).

<u>1H NMR</u> (400 MHz, $CDCl_3$) δ 7.66 – 7.54 (m, 2H), 7.44 – 7.32 (m, 3H), 7.29 – 7.20 (m, 2H), 6.97 – 6.90 (m, 1H), 6.89 – 6.82 (m, 2H), 4.40 (t, *J* = 3.6 Hz, 2H), 4.13 (t, *J* = 7.7 Hz, 2H), 1.59 – 1.49 (m, 2H).

<u>13C NMR</u> (101 MHz, $CDCl_3$) δ 158.6 (1C), 135.3 (2C), 131.4 (1C), 129.8 (1C), 129.4 (2C), 128.1 (2C), 120.7 (1C), 114.6 (2C), 65.1 (1C), 11.7 (1C).

<u>HRMS (EI)</u> calcd for [M, $C_{14}H_{16}OSi$]$^+$: 228.0970, found: 228.0966.

(2-butoxyethyl)(phenyl)silane (8ma)

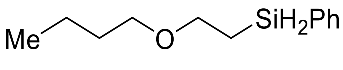

Serial number: lmp-3-144, 137.0 mg, 94% yield, colorless oil. Amount of EtMgBr, 5 mol%. R_f = 0.63 (PE/EA = 20:1, *v*/*v*).

<u>^{1}H NMR</u> (400 MHz, $CDCl_3$) δ 7.61 – 7.56 (m, 2H), 7.42 – 7.32 (m, 3H), 4.31 (t, *J* = 3.6 Hz, 2H), 3.55 (t, *J* = 7.8 Hz, 2H), 3.39 (t, *J* = 6.6 Hz, 2H), 1.59 – 1.49 (m, 2H), 1.41 – 1.31 (m, 4H), 0.91 (t, *J* = 7.3 Hz, 3H).

<u>^{13}C NMR</u> (101 MHz, $CDCl_3$) δ 135.2 (2C), 132.0 (1C), 129.6 (1C), 128.0 (2C), 70.3 (1C), 67.7 (1C), 31.8 (1C), 19.4 (1C), 13.9 (1C), 12.1 (1C).

<u>HRMS (EI)</u> calcd for [M, $C_{12}H_{20}OSi$]$^+$: 208.1283, found: 208.1288.

trimethyl(2-(phenylsilyl)ethyl)silane (8na)[17]

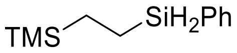

Serial number: sw-4-52, 136.8 mg, 94% yield, colorless oil. Complex **C1d** was used as the catalyst. R_f= 0.76 (PE).

<u>^{1}H NMR</u> (400 MHz, $CDCl_3$) δ 7.62 – 7.55 (m, 2H), 7.43 – 7.32 (m, 3H), 4.31 (t, *J* = 3.6 Hz, 2H), 0.91 – 0.82 (m, 2H), 0.62 – 0.53 (m, 2H), -0.02 (s, 9H).

<u>^{13}C NMR</u> (101 MHz, $CDCl_3$) δ 135.2 (2C), 132.9 (1C), 129.5 (1C), 127.9 (2C), 10.6 (1C), 2.7 (1C), -2.1 (3C).

dimethyl(phenyl)(2-(phenylsilyl)ethyl)silane (8oa)

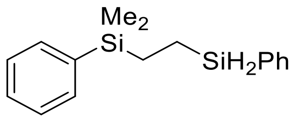

Serial number: sw-3-180, 176.0 mg, 93% yield, colorless oil. R_f= 0.45 (PE).

<u>^{1}H NMR</u> (400 MHz, $CDCl_3$) δ 7.55 – 7.46 (m, 4H), 7.42 – 7.30 (m, 6H), 4.27 (t, *J* = 3.3 Hz, 2H), 0.89 – 0.77 (m, 4H), 0.26 (s, 6H).

<u>^{13}C NMR</u> (101 MHz, $CDCl_3$) δ 139.0 (1C), 135.3 (2C), 133.7 (2C), 132.7 (1C), 129.6 (1C), 128.9 (1C), 128.0 (2C), 127.8 (2C), 9.8 (1C), 2.8 (1C), -3.5 (2C).

<u>HRMS (ESI)</u> calculated for [M-H, $C_{16}H_{21}Si_2$]$^-$: 269.1187, found: 269.1176.

6. Gram-scale experiment and product transformations

6.1 Gram-scale experiment

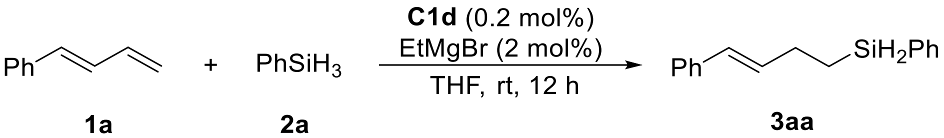

In an argon-filled glovebox, a vial (125 mL) was charged with complexes **C1d** (14.2 mg, 0.02 mmol), dry anhydrous THF (20 mL), conjugated diene **1a** (1.3 g, 10 mmol, 1.0 equiv) and phenylsilane **2a** (1.2 g, 11 mmol, 1.1 equiv) successively. The vial was removed from the glovebox after EtMgBr (1 M in THF, 200 μL, 0.2 mmol, 2.0 mol%) was added and the reaction mixture was stirred at room temperature for 12 h. When the reaction was finished, the mixture was concentrated by rotating evaporation. The residue was purified by column chromatography to afford the desired products **3aa** as a colorless oil (2.3 g, 97% yield, >98:2 r.r.).

6.2 Synthesis of (*E*)-dimethoxy(phenyl)(4-phenylbut-3-en-1-yl)silane (9)[3]

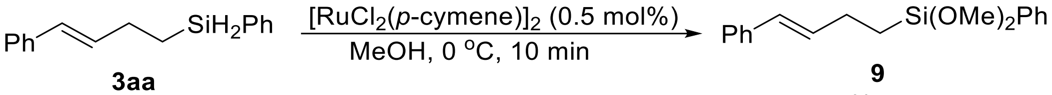

To a Schlenk tube (15 mL) was charged with $[RuCl_2(p\text{-cymene})]_2$ (1 mg, 0.0016 mmol), the tube was replaced with argon and anhydrous methanol (2 mL) was added throuth syringe. Then **3aa** (76.2 mg, 0.32 mmol) was added dropwise at 0 °C and stirred for 10 min. When the reaction was finished, the mixture was concentrated by rotating evaporation. The residue was purified by chromatography to afford the desired products **9** as a colorless oil (93.6 mg, 98% yield).

(*E*)-dimethoxy(phenyl)(4-phenylbut-3-en-1-yl)silane (9)

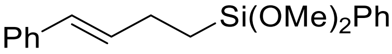

Serial number: sw-4-145, 93.6 mg, 98% yield, colorless oil. R_f = 0.5 (PE/EA = 20:1, *v:v*).

^{1}H NMR (400 MHz, $CDCl_3$) δ 7.56 – 7.51 (m, 2H), 7.40 – 7.27 (m, 3H), 7.24 – 7.15 (m, 4H), 7.13 – 7.06 (m, 1H), 6.26 (d, *J* = 15.8 Hz, 1H), 6.15 (dt, *J* = 15.7, 6.4 Hz, 1H), 3.53 (s, 6H), 2.28 – 2.14 (m, 2H), 1.03 – 0.91 (m, 2H).

^{13}C NMR (101 MHz, $CDCl_3$) δ 137.8 (1C), 134.3 (2C), 132.8 (1C), 130.2 (1C), 128.7 (1C), 128.4 (2C), 128.0 (2C), 126.7 (2C), 125.9 (2C), 50.7 (2C), 26.0 (1C), 12.0 (1C).

HRMS (EI) calculated for [M, $C_{18}H_{22}O_2Si$]$^+$: 298.1389, found: 298.1383.

6.3 Synthesis of (*E*)-difluoro(phenyl)(4-phenylbut-3-en-1-yl)silane (10)[3]

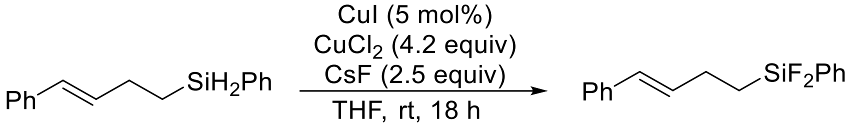

3aa

10
76% yield

To a Schlenk tube (15 mL) was charged with CuI (2.9 mg, 0.015 mmol, 5 mol%), $CuCl_2$ (168.8 mg, 1.26 mmol, 4.2 equiv) and CsF (115.5 mg, 0.76 mmol, 2.5 equiv), the tube was replaced with argon and anhydrous THF (2 mL) was added. Then **3aa** (71.4 mg, 0.3 mmol) was added dropwise at room temperature and stirred for 18 h. When the reaction was finished, the mixture was concentrated by rotating evaporation. The resulting crude product was distilled under reduced pressure using a high vacuum pump, and the fraction was collected as **10** as a colorless oil (62.5 mg, 76% yield).

(*E*)-difluoro(phenyl)(4-phenylbut-3-en-1-yl)silane (10)

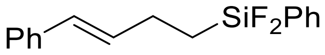

Serial number: sw-4-162, 62.5 mg, 76% yield, colorless oil.

^1H NMR (400 MHz, $CDCl_3$) δ 7.71 – 7.61 (m, 2H), 7.58 – 7.50 (m, 1H), 7.48 – 7.40 (m, 2H), 7.34 – 7.26 (m, 4H), 7.24 – 7.16 (m, 1H), 6.39 (d, J = 16.1 Hz, 1H), 6.22 (dt, J = 15.6, 6.6 Hz, 1H), 2.50 – 2.37 (m, 2H), 1.30 – 1.22 (m, 2H).

^{13}C NMR (101 MHz, $CDCl_3$) δ 137.4 (1C), 133.7 (1C), 131.9 (1C), 130.9 (1C), 129.9 (1C), 129.0 (J = 20.2 Hz, 1C), 128.5 (2C), 128.3 (2C), 127.1 (2C), 126.0 (2C), 24.9 (1C), 12.0 (J = 20.2 Hz, 1C).

HRMS (EI) calculated for [M, $C_{16}H_{16}F_2Si$]$^+$: 274.0989, found: 274.0985.

6.4 Synthesis of (*E*)-4-phenylbut-3-en-1-ol (11)[3]

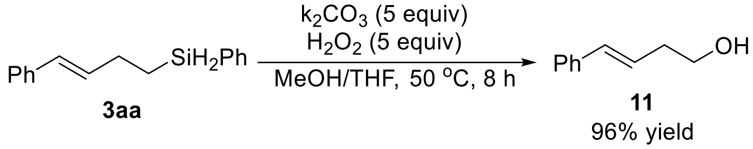

To a solution of **3aa** (71.4 mg, 0.3 mmol, 1.0 equiv) in MeOH and THF (3 mL, MeOH/THF = 1:1, *v*/*v*), K_2CO_3 (207 mg, 1.5 mmol, 5.0 equiv) and hydrogen peroxide (0.18 mL, 5 equiv, 30% aqueous solution) were added insequencely. Then the mixture was stirred at 50℃. After 8 h, the mixture was etracted with EtOAc and then the organic layer was separated and washed with brine, dried over anhydrous Na_2SO_4 and concentrated in vacuo. The residue was purified by column chromatography to afford the desired products **11** as a colorless oil (42.5 mg, 96% yield).

(*E*)-4-phenylbut-3-en-1-ol (11)

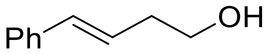

Serial number: sw-4-131, 42.5 mg, 96% yield, colorless oil. R_f= 0.35 (PE/EA = 5:1, *v*:*v*).

^{1}H NMR (400 MHz, $CDCl_3$) δ 7.39 – 7.28 (m, 4H), 7.25 – 7.18 (m, 1H), 6.50 (d, *J* = 15.8 Hz, 1H), 6.21 (dt, *J* = 15.8, 7.1 Hz, 1H), 3.76 (t, *J* = 6.3 Hz, 2H), 2.54 – 2.43 (m, 2H), 1.64 – 1.54 (br, 1H).

^{13}C NMR (101 MHz, $CDCl_3$) δ 137.2 (1C), 132.8 (1C), 128.5 (2C), 127.2 (1C), 126.3 (1C), 126.1 (2C), 62.0 (1C), 36.4 (1C).

6.5 Synthesis of phenyl((*E*)-4-phenylbut-3-en-1-yl)((*E*)-styryl)silane (12)[18]

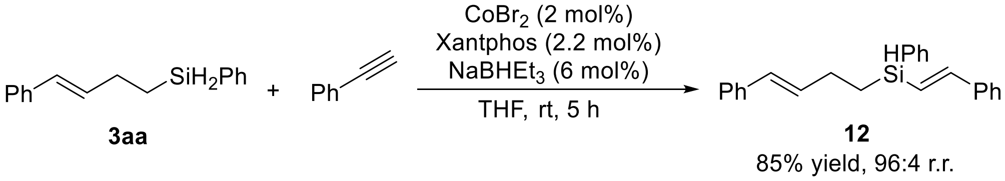

In an argon-filled glovebox, a vial (10 ml) was charged with $CoBr_2$ (1.3 mg, 0.006 mmol, 2 mol%), Xantphos (3.8 mg, 0.0066 mmol, 2.2 mol%) and anhydrous THF (2 mL), the mixture was stirred at room temperature for 2 h. Then **3aa** (71.4 mg, 0.3 mmol, 1.0 equiv) and phenylacetylene (30.6 mg, 0.3 mmol, 1.0 equiv) were added. The vial was removed from the glovebox after $NaBHEt_3$ (1 M in THF, 18 μL, 0.018 mmol, 6.0 mol%) was added and the

reaction mixture was stirred at room temperature for 3 h. When the reaction was finished, the mixture was concentrated by rotating evaporation. The residue was purified by chromatography to afford the desired products **12** as a colorless oil (86.3 mg, 85% yield, 96:4 r.r.).

phenyl((*E*)-4-phenylbut-3-en-1-yl)((*E*)-styryl)silane (12)

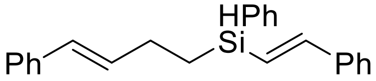

Serial number: sw-4-170, 86.3 mg, 85% yield, 96:4 r.r., colorless oil. R_f= 0.54 (PE).

<u>^{1}H NMR</u> (400 MHz, $CDCl_3$) δ 7.65 – 7.57 (m, 2H), 7.44 – 7.25 (m, 12H), 7.21 – 7.16 (m, 1H), 7.08 (d, *J* = 19.1 Hz, 1H), 6.56 (dd, *J* = 19.1, 3.4 Hz, 1H), 6.37 (d, *J* = 15.9 Hz, 1H), 6.28 (dt, *J* = 15.8, 6.3 Hz, 1H), 4.70 (q, *J* = 3.5 Hz, 1H), 2.39 (dt, *J* = 9.7, 6.5 Hz, 2H), 1.26 – 1.19 (m, 2H).

<u>^{13}C NMR</u> (101 MHz, $CDCl_3$) δ 147.9 (1C), 137.9 (1C), 137.7 (1C), 134.9 (2C), 134.4 (1C), 132.7 (1C), 129.6 (1C), 129.0 (1C), 128.6 (2C), 128.4 (3C), 128.0 (2C), 126.8 (1C), 126.6 (2C), 125.9 (2C), 122.3 (1C), 27.8 (1C), 12.1 (1C).

<u>HRMS (EI)</u> calculated for [M, $C_{24}H_{24}Si$]$^+$: 340.1647, found: 340.1640.

6.6 Synthesis of polyorganosiloxane 13

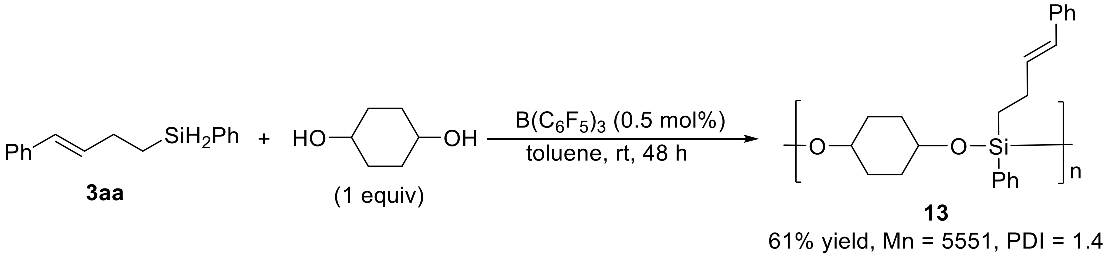

In an argon-filled glovebox, a Schlenk tube (25 mL) was charged with 1,4-cyclohexanediol (116 mg, 1 mmol, 1 equiv), $B(C_6F_5)_3$ (2.6 mg, 0.005 mmol, 0.5 mol%), anhydrous toluene (3 mL) and **3aa** (238 mg, 1 mmol, 1.0 equiv) successively. The tube was removed from the glovebox, connected to an argon filled balloon and stirred at room temperature for 48 h. When the reaction was finished, the mixture was concentrated by rotating evaporation. The residue was dissolved in THF (1 mL), which was added dropwise to hexane

(50 mL). The emulsion formed was centrifuged, and the residue obtained after removing the supernatant was re-precipitated and centrifuged twice more according to the above method. The final residue was collected and dried under vacuum as the target product **13** as solid-liquid mixtures (213.5 mg, 61% yield). The M_n and M_w/M_n values were determined by GPC with THF solvent and polystyrene standards, M_n = 5551, M_w/M_n = 1.4.

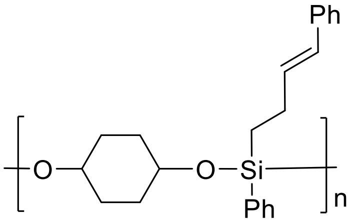

Serial number: sw-5-19B, 213.5 mg, 61% yield, solid-liquid mixtures.

^{1}H NMR (400 MHz, $CDCl_3$) δ 7.6 – 77.53 (m, 2H), 7.43 – 7.29 (m, 3H), 7.28 – 7.18 (m, 4H), 7.17 – 7.09 (m, 1H), 6.40 – 6.06 (m, 2H), 3.97 – 3.68 (m, 2H), 2.35 – 2.15 (m, 2H), 1.99 – 1.73 (m, 4H), 1.46 – 1.30 (m, 4H), 1.07 – 0.92 (m, 2H).

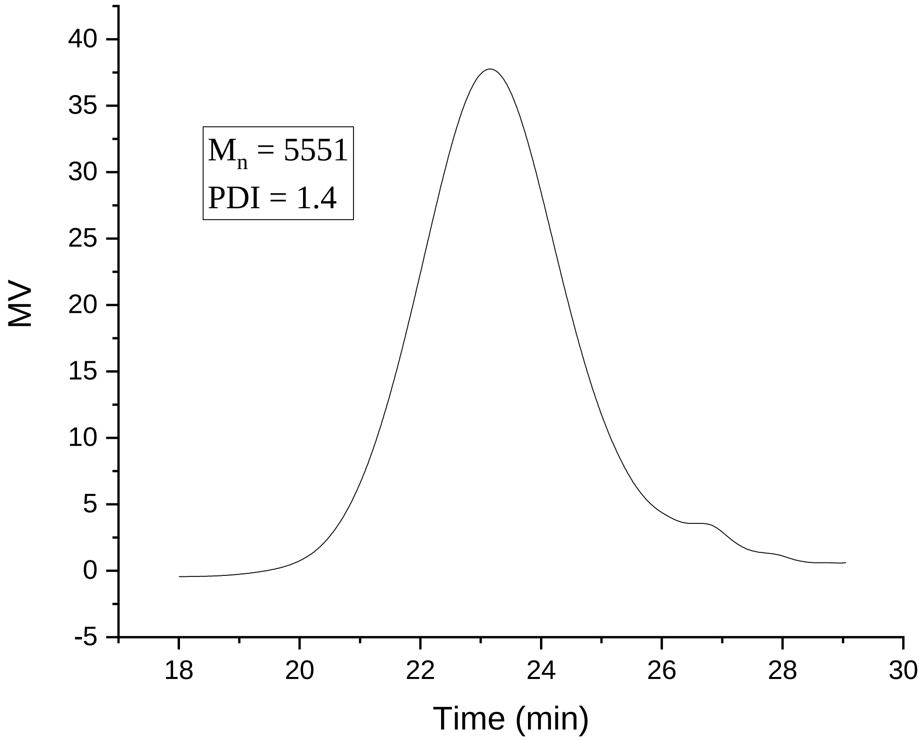

Scheme S1 GPC Spectra of Polymer13

6.7 Synthesis of polyorganosiloxane 14[19]

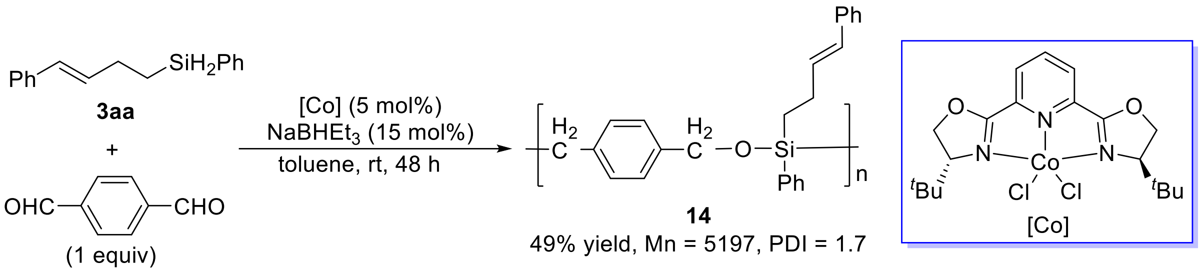

In an argon-filled glovebox, a vial (10 mL) was charged with [Co] (11.5 mg, 0.025 mmol, 5 mol%), anhydrous toluene (3 mL), **3aa** (119 mg, 0.5 mmol) and terephthalaldehyde (67 mg, 0.5 mmol, 1.0 equiv) successively. The vial was removed from the glovebox after $NaBEt_3H$ (1.0 M in THF, 75 μL, 0.075 mmol, 15 mol%) was added and stirred at room temperature for 48 h. When the reaction was finished, the mixture was concentrated by rotating evaporation. The residue was dissolved in THF (1 mL), which was added dropwise to hexane (50 mL). The emulsion formed was centrifuged, and the residue obtained after removing the supernatant was re-precipitated and centrifuged twice more according to the above method. The final residue was collected and dried under vacuum as the target product **14** as brown viscous liquid (91.2 mg, 49% yield). The M_n and M_w/M_n values were determined by GPC with THF solvent and polystyrene standards, M_n = 5197, M_w/M_n = 1.7.

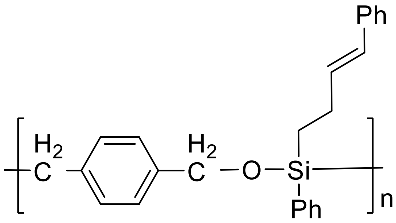

Serial number: sw-4-190, 91.2 mg, 49% yield, brown viscous liquid.

^{1}H NMR (400 MHz, $CDCl_3$) δ 7.73 – 7.19 (m, 14H), 6.50-5.95 (m, 2H), 5.00 – 4.55 (m, 4H), 2.50 – 2.00 (m, 2H), 1.22 – 0.98 (m, 2H).

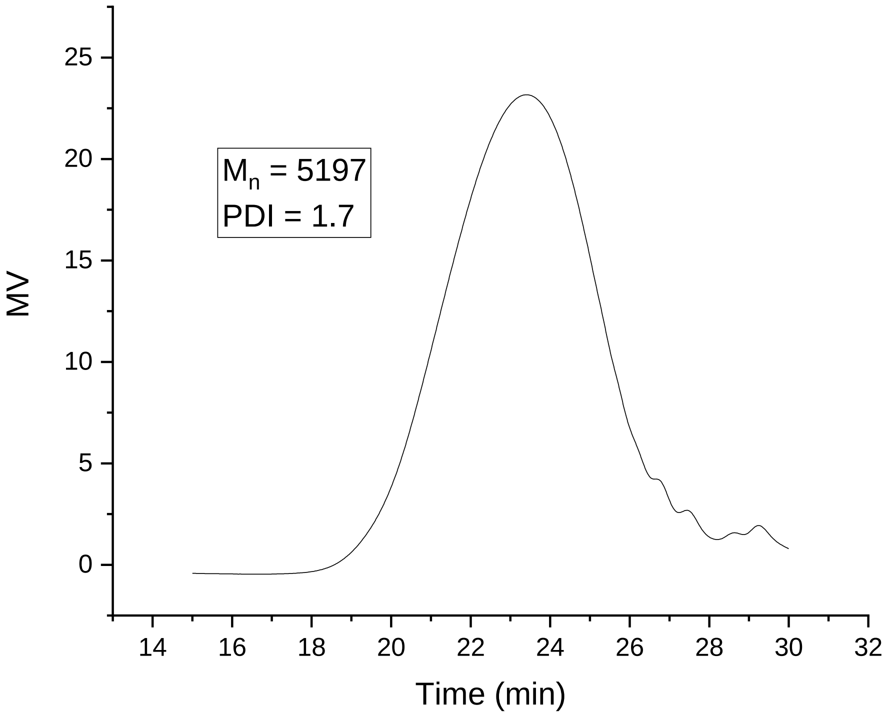

Scheme S2 GPC Spectra of Polymer 14

7. Mechanistic studies

7.1 Hydrosilylation of an *E*/*Z* mixture of 1,3-diene 1a’

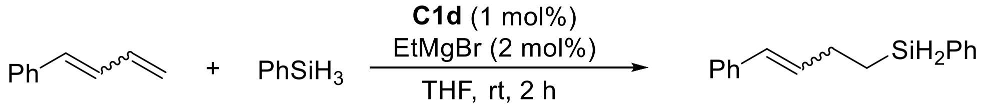

1a' *E*/*Z* = 38:62   **2a**   **3aa'** 98% yield, >98:2 r.r., *E*/*Z* = 38:62

In an argon-filled glovebox, a vial (10 mL) was charged with complexes **C1d** (3.6 mg, 0.005 mmol), dry anhydrous THF (1 mL), 1,3-dienes **1a’** (65 mg, 0.5 mmol, *E*/*Z* = 38:62) and silanes **2a** (59.4 mg, 0.55 mmol, 1.1 equiv) successively. The reaction mixture was stirred at room temperature for 2 h after EtMgBr (1 M in THF, 10 μL, 0.01 mmol, 2.0 mol%) was added. The vial was removed from the glovebox and the reaction mixture was concentrated by rotating evaporation. The residue was purified by column chromatography to afford the products **3aa’** as a colorless oil (117 mg, 98% yield, >98:2 r.r., *E/Z* = 38:62).

(*E*)-phenyl(4-phenylbut-3-en-1-yl)silane (***E*-3aa**)[12]

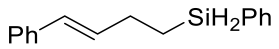

<u>^{1}H NMR</u> (400 MHz, $CDCl_3$) δ 7.62 – 7.56 (m, 2H), 7.44 – 7.15 (m, 8H), 6.43 – 6.33 (m, 1H), 6.25 (dt, *J* = 15.6, 6.5 Hz, 1H), 4.34 (t, *J* = 3.3 Hz, 2H), 2.37 (q, *J* = 7.4 Hz, 2H), 1.19 – 1.03 (m, 2H).

(*Z*)-phenyl(4-phenylbut-3-en-1-yl)silane (**Z-3aa**)[20]

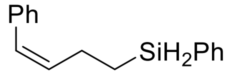

<u>^{1}H NMR</u> (400 MHz, $CDCl_3$) δ 7.56 – 7.49 (m, 2H), 7.44 – 7.15 (m, 8H), 6.43 – 6.33 (m, 1H), 5.70 (dt, *J* = 11.5, 7.3 Hz, 1H), 4.31 (q, *J* = 3.3 Hz, 2H), 2.48 (q, *J* = 7.7 Hz, 2H), 1.19 – 1.03 (m, 2H).

7.2 Deuterium labelling experiments

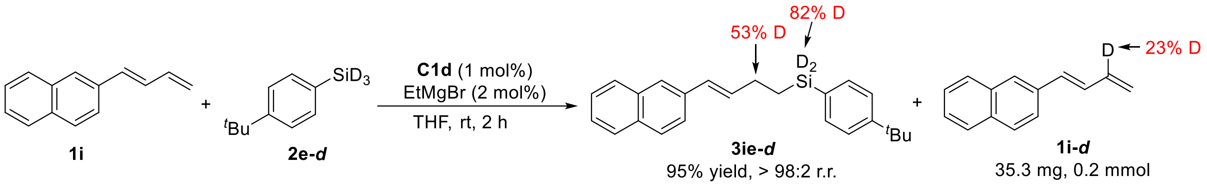

In an argon-filled glovebox, a vial (10 mL) was charged with complexes **C1d** (1.42 mg, 0.002 mmol), dry anhydrous THF (1 mL), **1i** (72 mg, 0.4 mmol, 2 equiv) and silane **2e-*d*** (33.4 mg, 0.20 mmol) successively. The reaction mixture was stirred at room temperature for 2 h after EtMgBr (1 M in THF, 4 μL, 0.004 mmol, 2.0 mol%) was added. The vial was removed from the glovebox and the reaction mixture was concentrated by rotating evaporation. The residue was purified by column chromatography to afford the products **3ie-*d*** (65.8 mg, 95% yield, >98:2 r.r.) and **1i-*d*** (35.3 mg, 98% recovery). The ^{2}H NMR spectroscopy was measured with $CDCl_3$ as an internal standard, and the 1,3,5-trimethoxybenzene was used as an internal standard to determine the ratio of product and $CDCl_3$ by ^{1}H NMR.

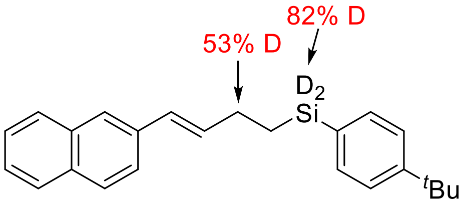

3ie-*d*, 65.8 mg, 95% yield, >98:2 r.r., colorless oil, R_f= 0.22 (PE).

^{1}H NMR (400 MHz, $CDCl_3$) δ 7.79 – 7.70 (m, 3H), 7.64 (s, 1H), 7.57 – 7.49 (m, 3H), 7.47 – 7.35 (m, 4H), 6.53 (d, *J* = 15.7 Hz, 1H), 6.37 (dt, *J* = 15.7, 6.3 Hz, 1H), 4.35 (q, *J* = 3.8 Hz, 0.36 H), 2.47 – 2.36 (m, 0.95 H), 1.32 (s, 9H), 1.14 (d, *J* = 8.0 Hz, 2H).

^{13}C NMR (101 MHz, $CDCl_3$) δ 152.8 (1C), 135.3 (2C), 135.2 (1C), 133.7 (1C), 132.8 (1C), 132.7 (1C), 129.4 (1C), 128.6 (1C), 128.1 (1C), 127.9 (1C), 127.7 (1C), 126.2 (1C), 125.5 (2C), 125.1 (2C), 123.6 (1C), 34.8 (1C), 31.3 (3C), 28.3 (1C), 9.9 (1C).

The ^{2}H NMR analysis is well consistent with the ^{1}H NMR analysis. See followed spectrum:

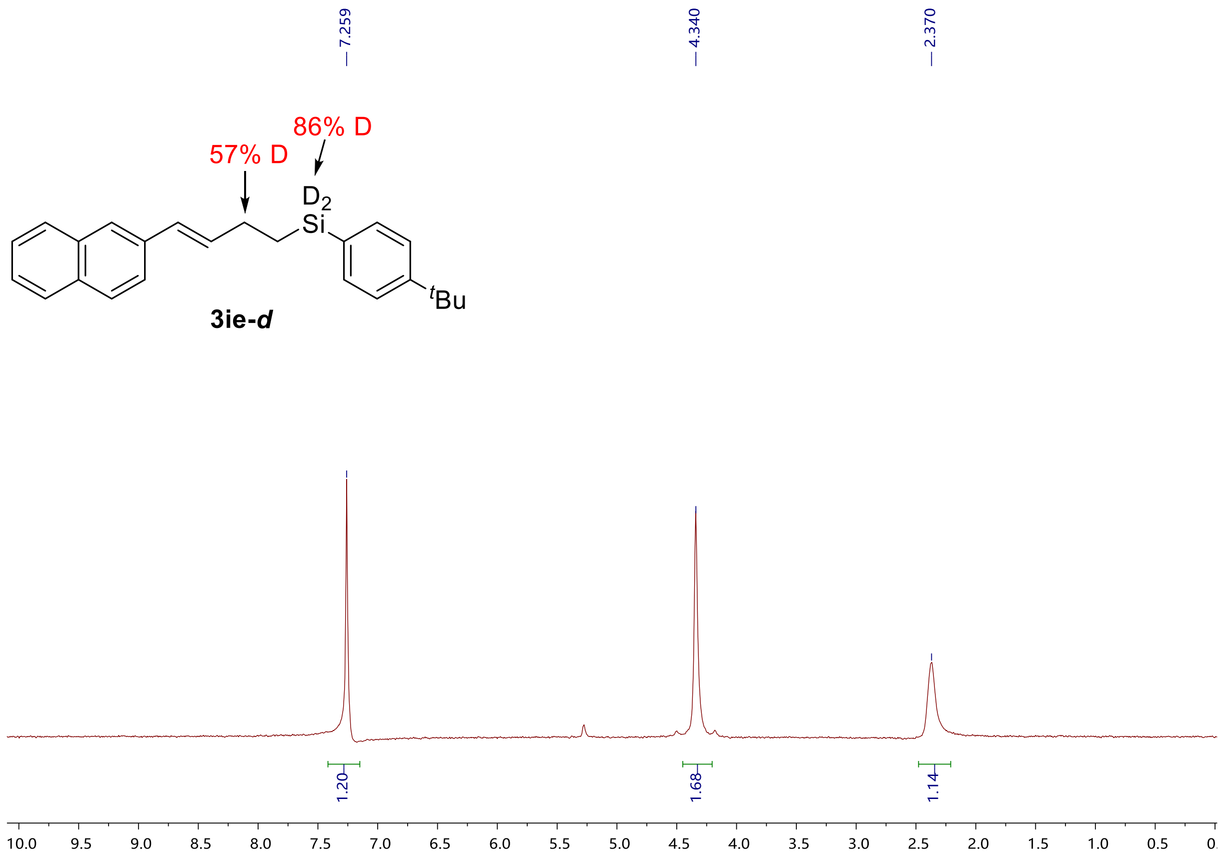

Figure S1. ^{2}H NMR of the product **3ie-*d*.** $CDCl_3$ (1.20 equiv) was used as an internal standard..

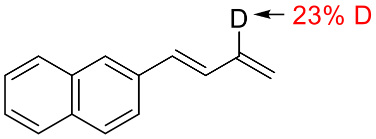

1i-*d*, 35.3 mg, 0.2 mmol, white solid.

^{1}H NMR (400 MHz, $CDCl_3$) δ 7.82 – 7.71 (m, 4H), 7.65 – 7.58 (m, 1H), 7.48 – 7.38 (m, 2H), 6.96 – 6.86 (m, 1H), 6.72 (d, *J* = 15.6 Hz, 1H), 6.56 (dt, *J* = 16.9, 10.2 Hz, 0.77 H), 5.38 (dd, *J* = 16.8, 1.4 Hz, 1H), 5.20 (dd, *J* = 9.9, 1.5 Hz, 1H).

^{13}C NMR (101 MHz, $CDCl_3$) δ 137.3 (1C), 134.6 (1C), 133.7 (1C), 133.0 (1C), 132.9 (1C) 130.0 (1C), 128.3 (1C), 128.0 (1C), 127.7 (1C), 126.6 (1C), 126.3 (1C), 125.9 (1C), 123.5 (1C), 117.8 (1C).

The ^{2}H NMR analysis is well consistent with the ^{1}H NMR analysis. See followed spectrum:

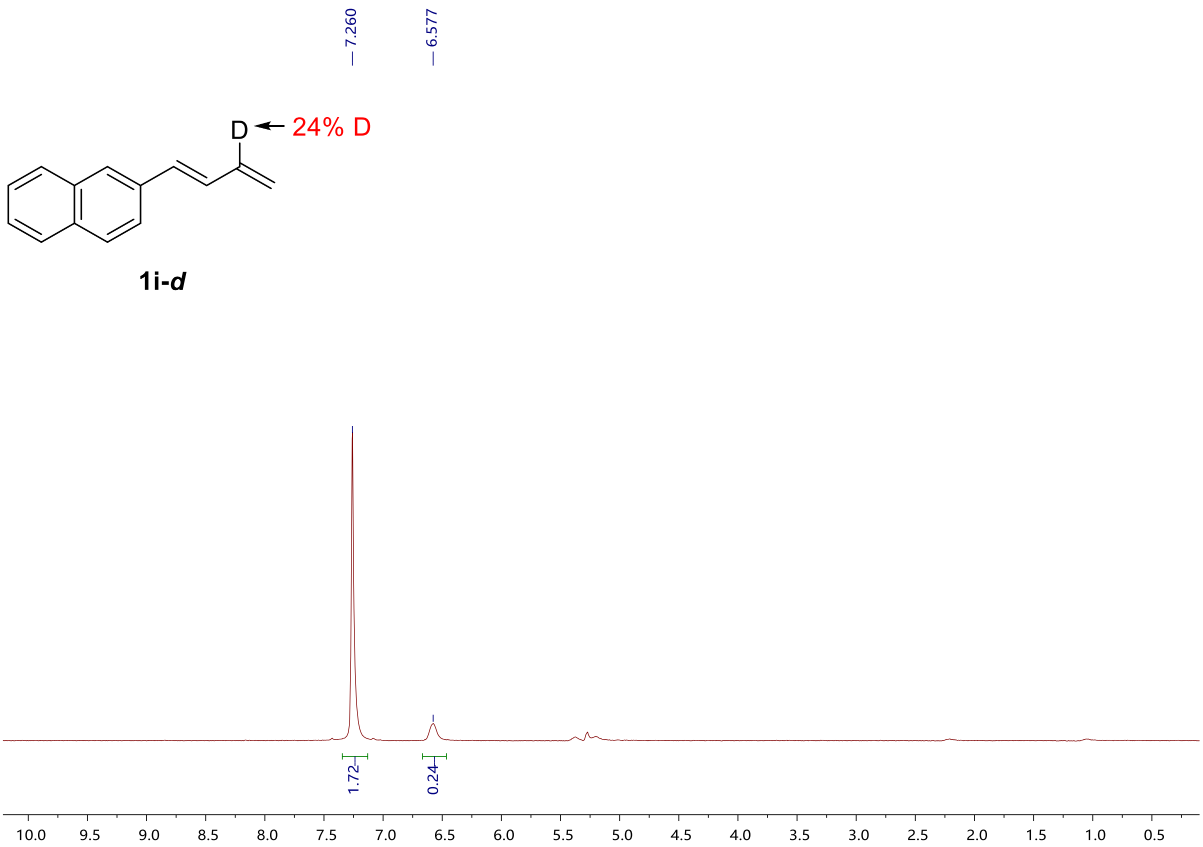

Figure S2. ^{2}H NMR of the product **1i-*d*.** $CDCl_3$ (1.72 equiv) was used as an internal standard.

7.3 Mixture silanes experiment

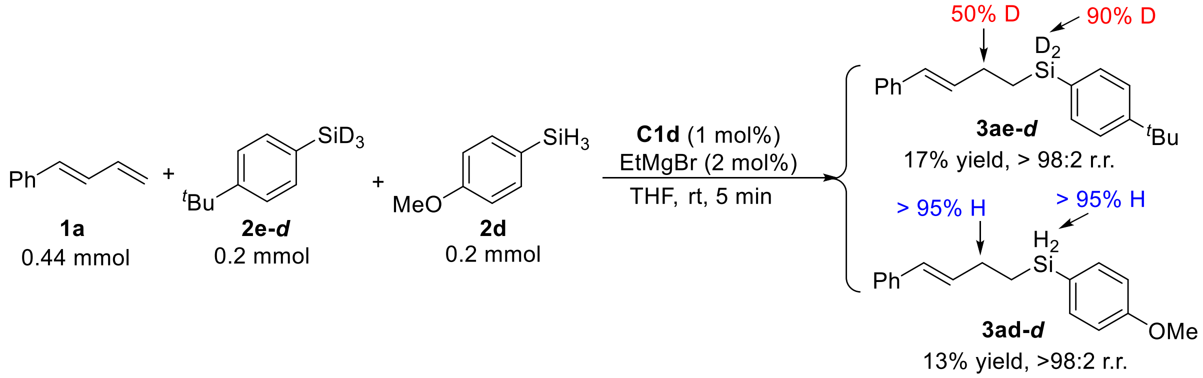

In an argon-filled glovebox, a vial (10 mL) was charged with complexes **C1d** (2.8 mg, 0.004 mmol), dry anhydrous THF (1 mL), conjugated diene **1a** (57.2 mg, 0.44 mmol, 2.2 equiv), silanes **2e-*d*** (33.4 mg, 0.2 mmol, 1.0 equiv) and **2d** (27.6 mg, 0.2 mmol, 1 equiv) successively. The reaction mixture was stirred at room temperature for 5 min after EtMgBr (1 M in THF, 8 μL, 0.004 mmol, 2.0 mol%) was added. The vial was removed from the glovebox and quenched with 2 drops water, the reaction mixture was concentrated by rotating evaporation. The residue was purified by column chromatography to afford the products **3ae-*d*** (20 mg, 17% yield, >98:2 r.r.) and **3ad-*d*** (14 mg, 13% yield, >98:2 r.r.). The ^{2}H NMR spectroscopy was measured with $CDCl_3$ as an internal standard, and the 1,3,5-trimethoxybenzene was used as an internal standard to determine the ratio of product and $CDCl_3$ by ^{1}H NMR.

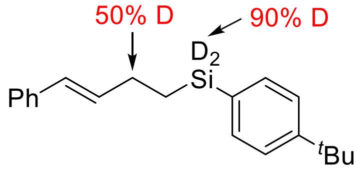

3ae-*d*, 20 mg, 17% yield, >98:2 r.r., colorless oil.

^{1}H NMR (400 MHz, $CDCl_3$) δ 7.55 – 7.50 (m, 2H), 7.43 – 7.37 (m, 2H), 7.33 – 7.26 (m, 4H), 7.22 – 7.16 (m, 1H), 6.38 (d, J = 16.4, 1H), 6.24 (dd, J = 15.8, 6.4 Hz, 1H), 4.32 (q, J = 3.8 Hz, 0.24 H), 2.42 – 2.31 (m, 0.98 H), 1.32 (s, 9H), 1.11 (d, J = 8.0 Hz, 2H).

^{13}C NMR (101 MHz, $CDCl_3$) δ 152.7 (1C), 137.7 (1C), 135.2 (2C), 132.3 (1C), 129.2 (1C), 128.6 (1C), 128.4 (2C), 126.8 (1C), 126.0 (2C), 125.0 (2C), 34.7 (1C), 31.2 (3C), 28.1 (1C) , 9.8 (1C).

The ^{2}H NMR analysis is well consistent with the ^{1}H NMR analysis. See followed spectrum:

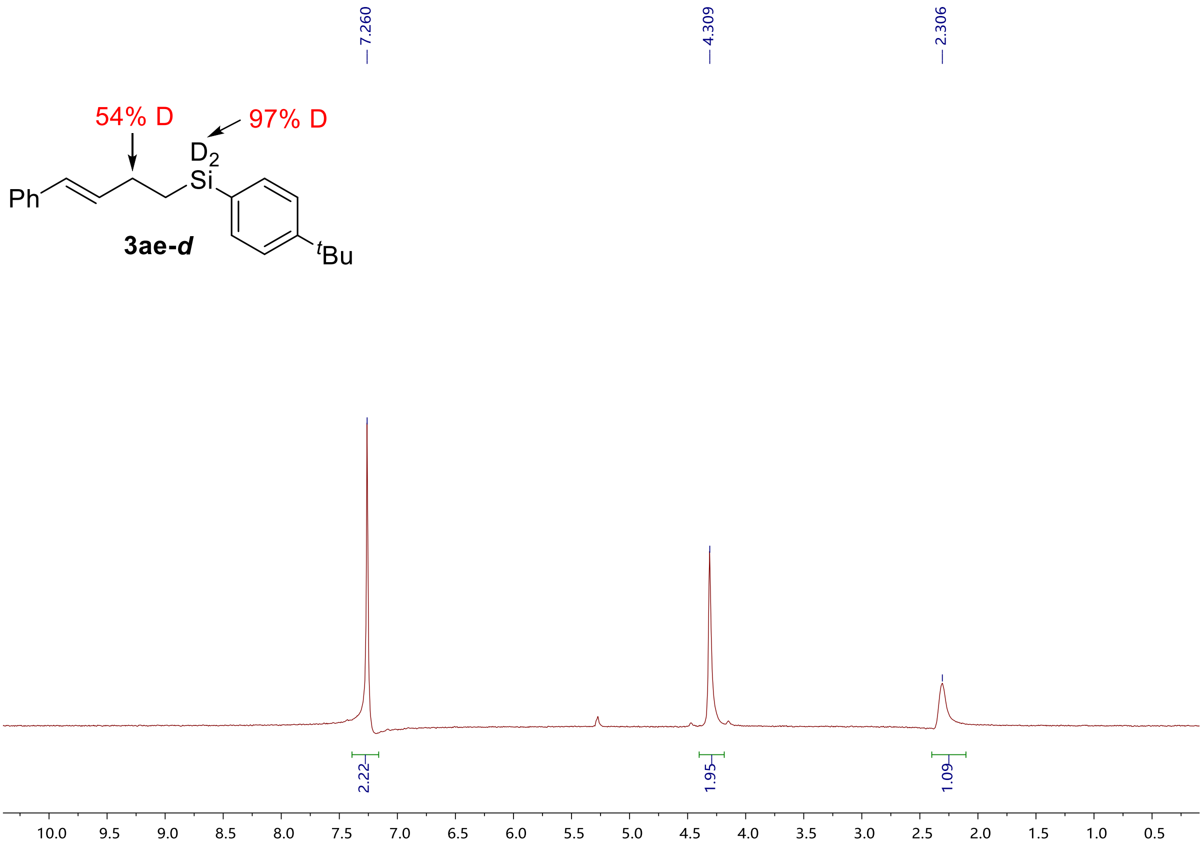

Figure S3. ^{2}H NMR of the product **3ae-*d*.** $CDCl_3$ (2.22 equiv) was used as an internal standard.

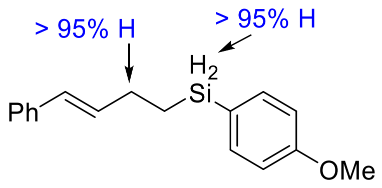

3ad-*d*, 14 mg, 13% yield, >98:2 r.r., colorless oil.

<u>^{1}H NMR</u> (400 MHz, $CDCl_3$) δ 7.53 – 7.47 (m, 2H), 7.33 – 7.27 (m, 4H), 7.22 – 7.15 (m, 1H), 6.95 – 6.88 (m, 2H), 6.37 (d, *J* = 15.8, 1H), 6.24 (dt, *J* = 15.7, 6.5 Hz, 1H), 4.32 (t, *J* = 3.6 Hz, 1.87 H), 3.82 (s, 3H), 2.40 – 2.31 (m, 1.94 H), 1.14 – 1.07 (m, 2H).

<u>^{13}C NMR</u> (101 MHz, $CDCl_3$) δ 160.9 (1C), 137.7 (1C), 136.7 (2C), 132.3 (1C), 129.2 (1C), 128.4 (2C), 126.8 (1C), 125.9 (2C), 122.8 (1C), 113.9 (2C), 55.0 (1C), 28.4 (1C), 10.2 (1C).

The ^{2}H NMR analysis is well consistent with the ^{1}H NMR analysis. See followed spectrum:

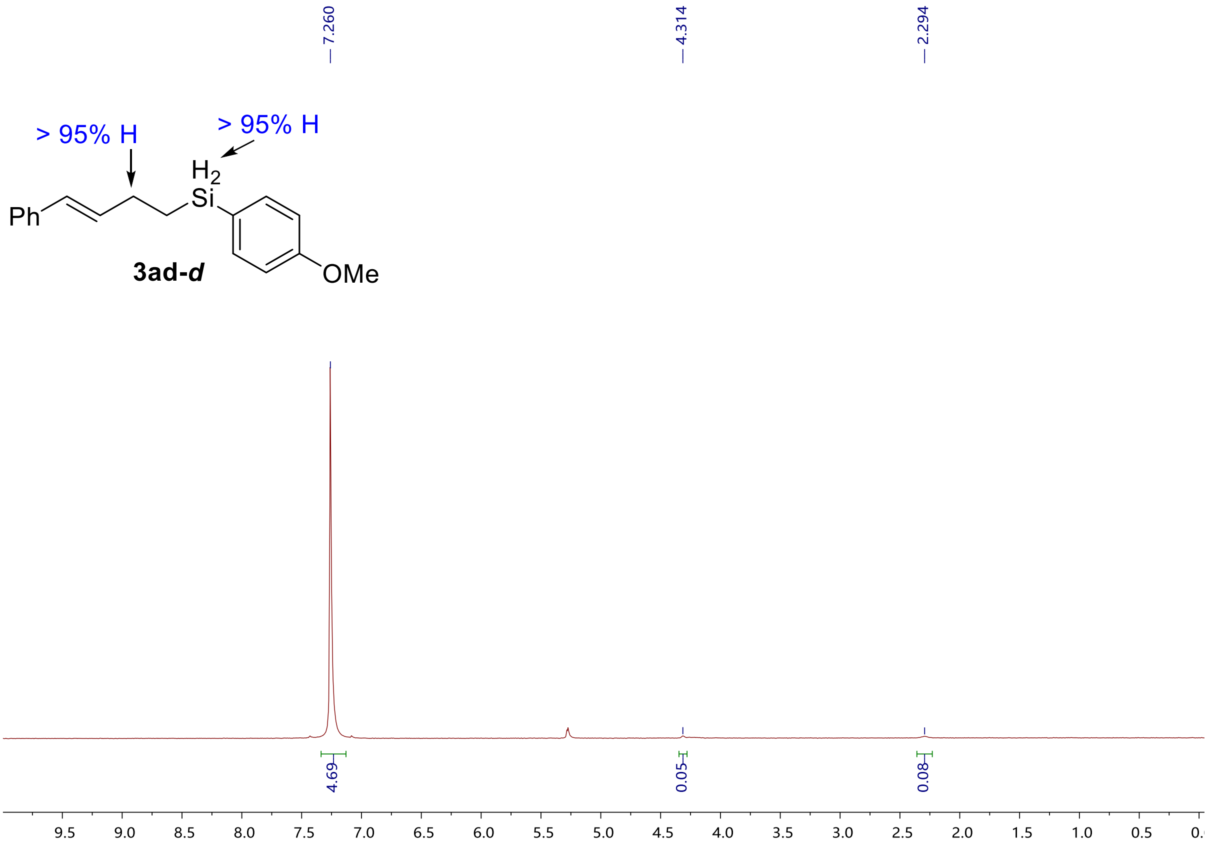

Figure S4. ^{2}H NMR of the product **3ad-*d*.** $CDCl_3$ (4.69 equiv) was used as an internal standard

7.4 Parallel kinetic isotope effect experiment

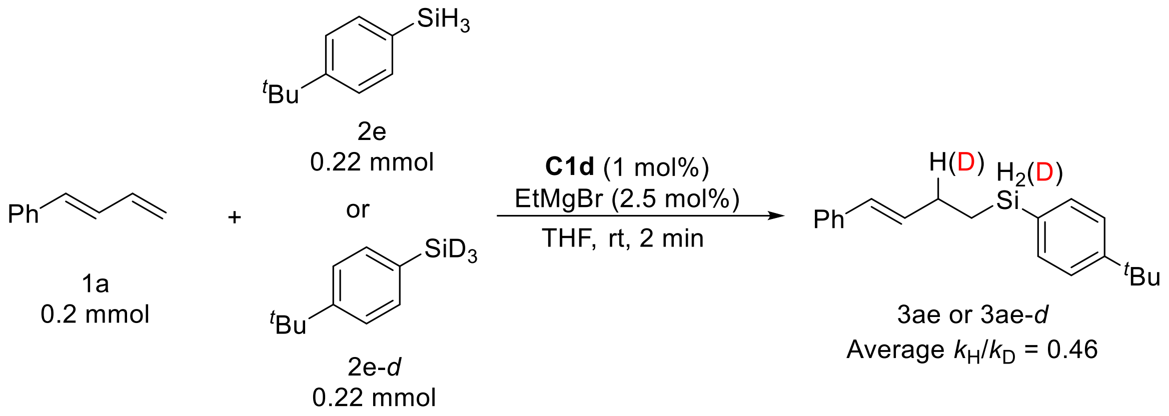

In an argon-filled glovebox, a vial (10 mL) was charged with complexes **C1d** (1.4 mg, 0.002 mmol), dry anhydrous THF (1 mL), conjugated diene **1a** (26.0 mg, 0.2 mmol), silane **2e** (36.1 mg, 0.22 mmol, 1.1 equiv) or **2e-*d*** (36.7 mg, 0.22 mmol, 1.1 equiv) successively. The reaction mixture was stirred at room temperature for 2 min after EtMgBr (1 M in THF, 5 μL, 0.005 mmol, 2.5 mol%) was added. The vial was removed from the glovebox and quenched with 2 drops water. Iron species were removed by flash column chromatography with DCM as eluent. The raw product was detected by ^{1}H NMR with 1,3,5-trimethoxybenzene as internal standard. We repeated the experiments for four times, respectively. The average of k_H/k_D is 0.46. The results were listed as following.

Table S3 Parallel kinetic isotope effect experiments

entry	% yield (**3ae**)	% yield (**3ae-*d***)
1	6.4	14.6
2	6.3	12.1
3	7.6	16.1
4	6.0	15.2
Average yield	6.6	14.5
k_H/k_D	0.46	

.

8 NMR spectra of all products

2-chloro-9-mesityl-1,10-phenanthroline (2a)

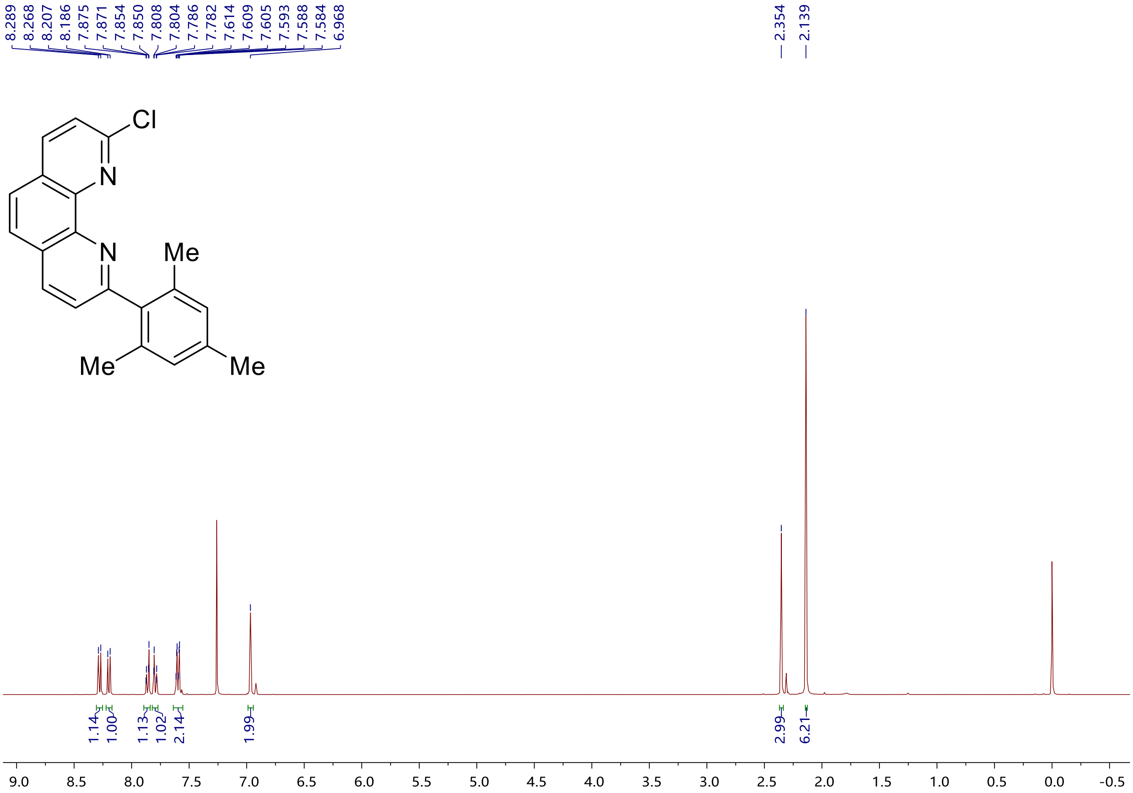

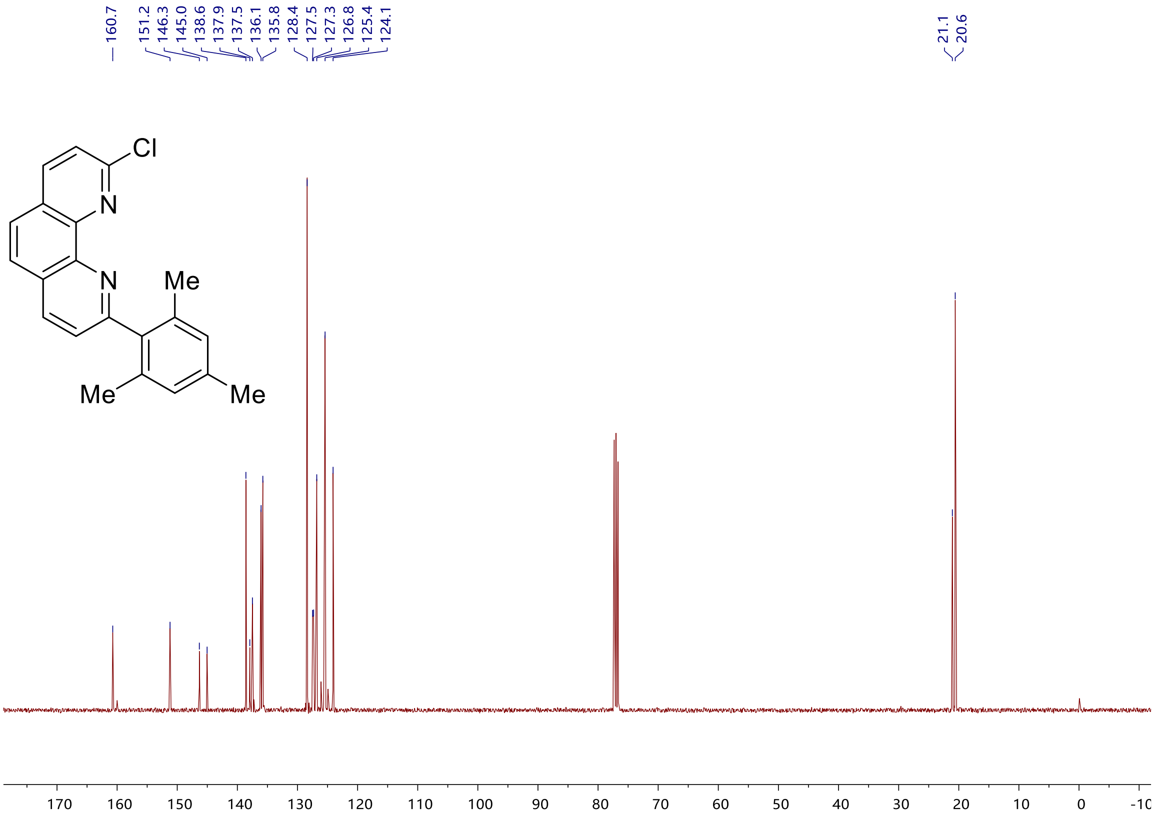

2-chloro-9-(2,4,6-triisopropylphenyl)-1,10-phenanthroline (2b)

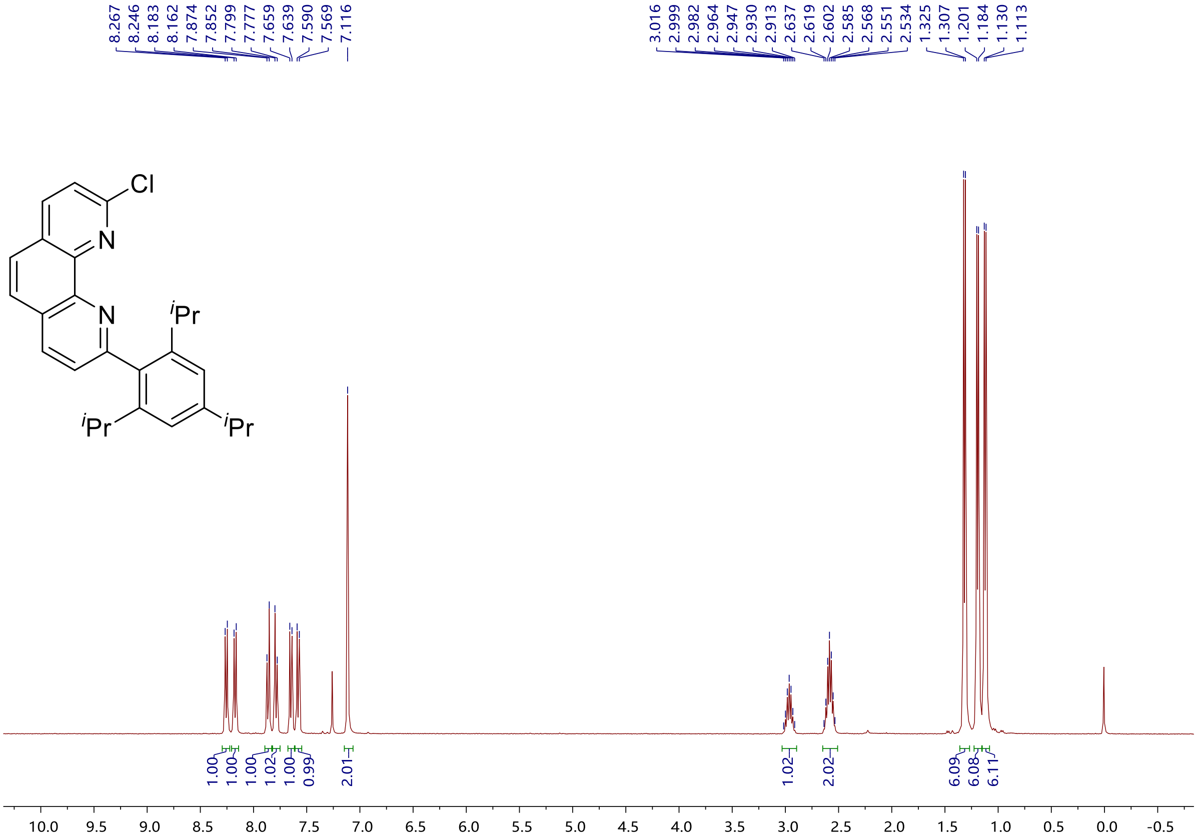

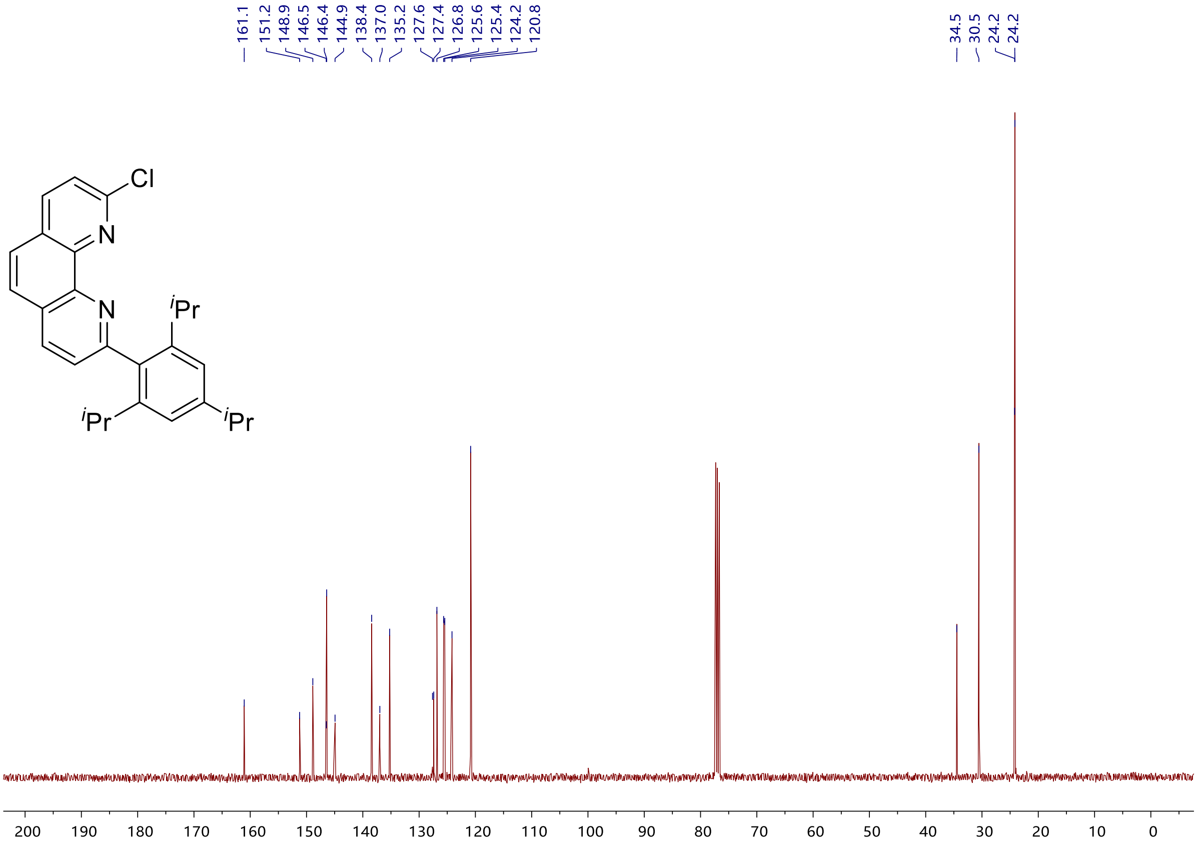

2-chloro-9-(3,5-di-*tert*-butylphenyl)-1,10-phenanthroline (2c)

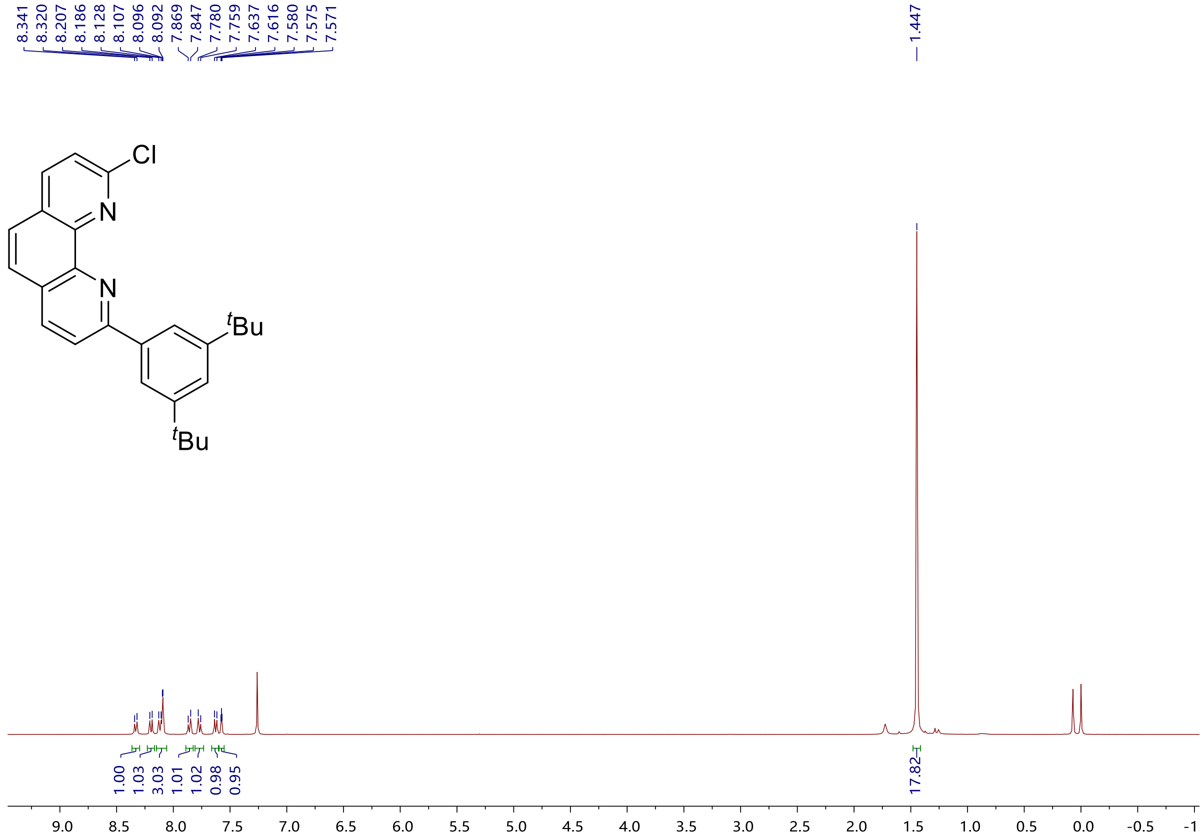

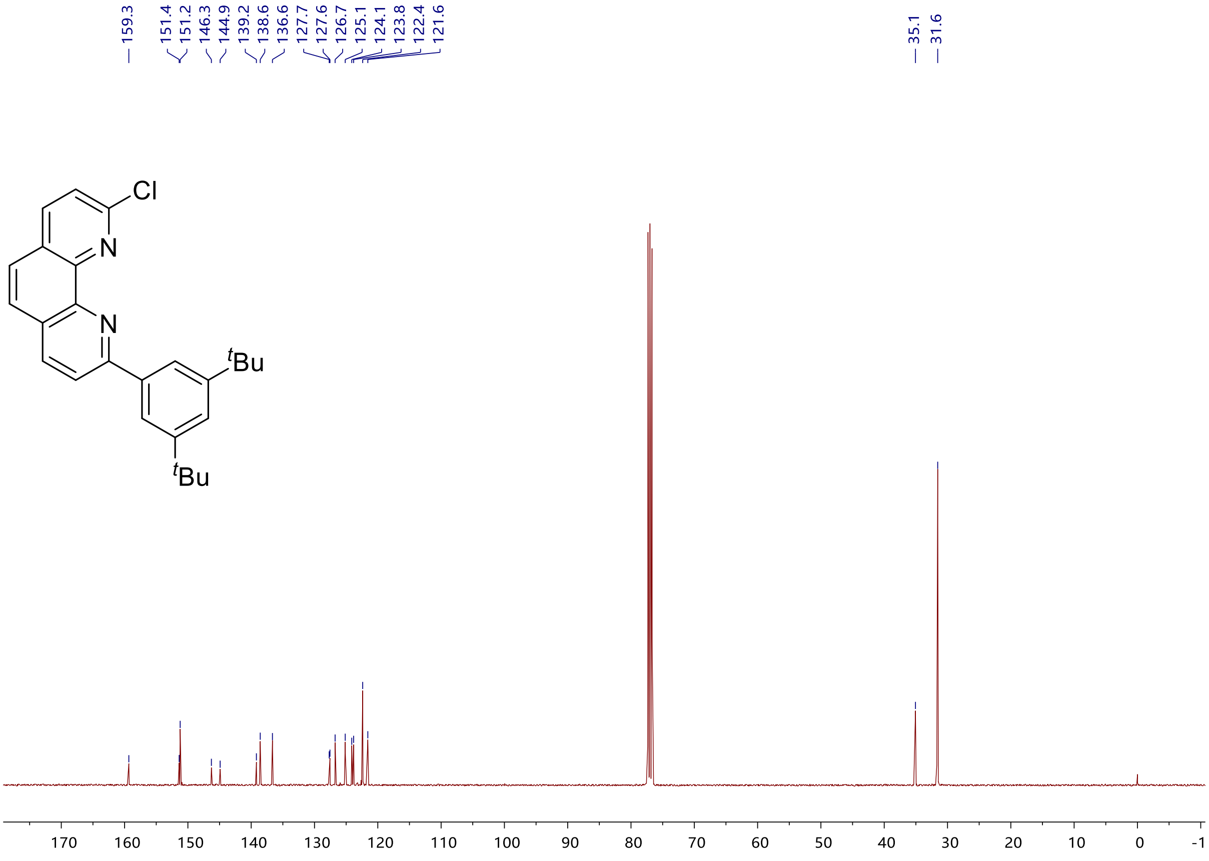

1-(9-mesityl-1,10-phenanthrolin-2-yl)ethan-1-one (3a)

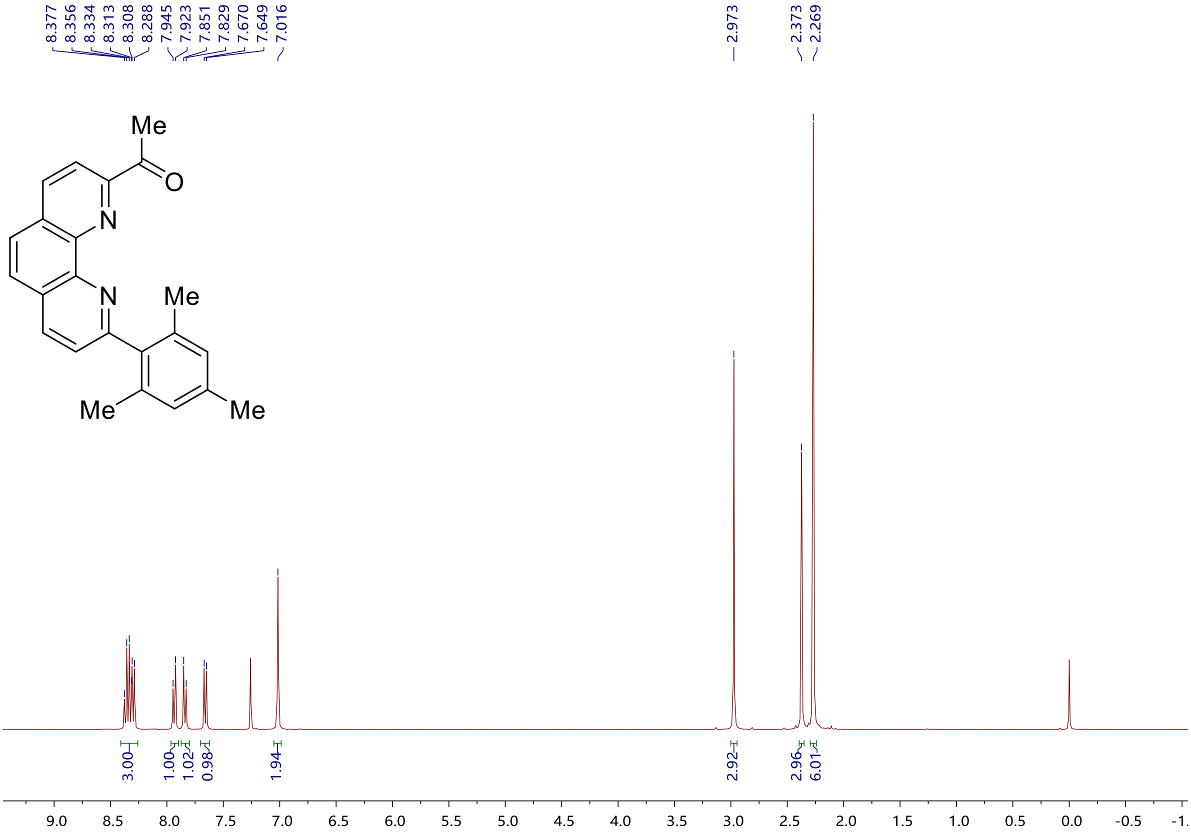

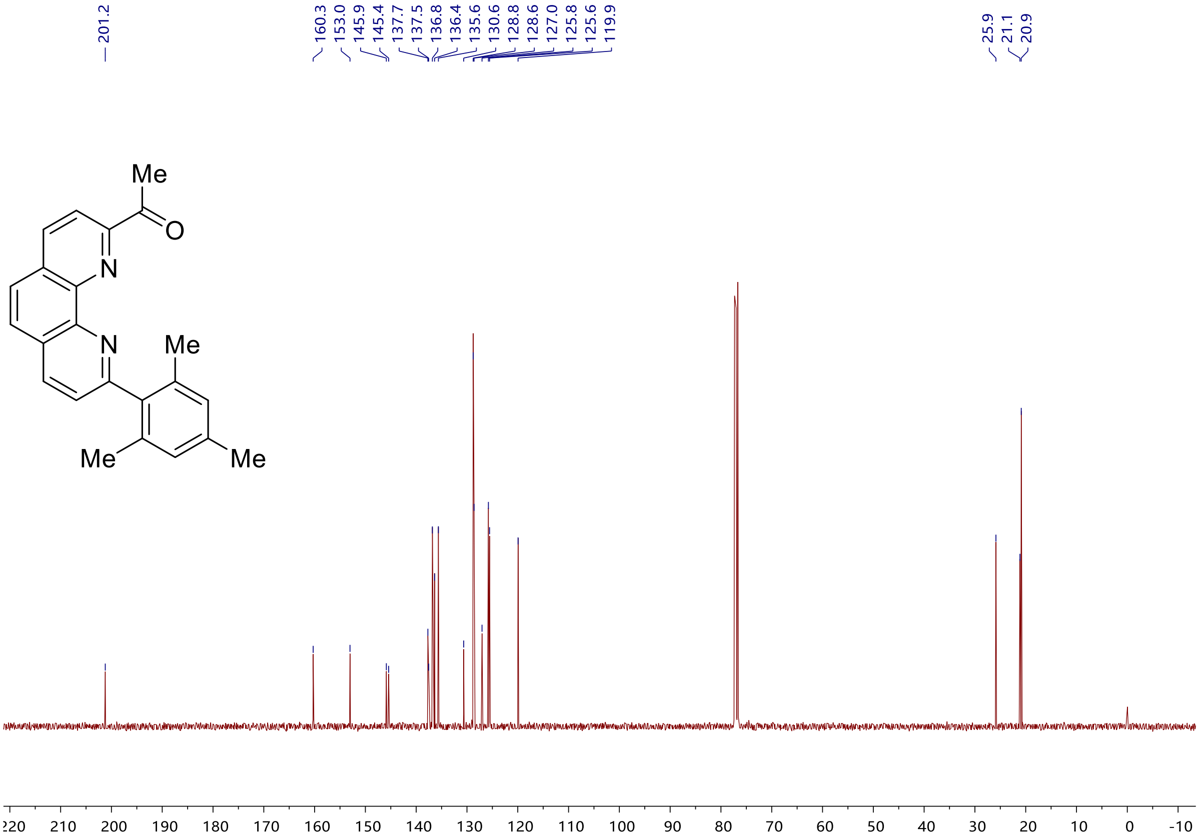

1-(9-(2,4,6-triisopropylphenyl)-1,10-phenanthrolin-2-yl)ethan-1-one (3b)

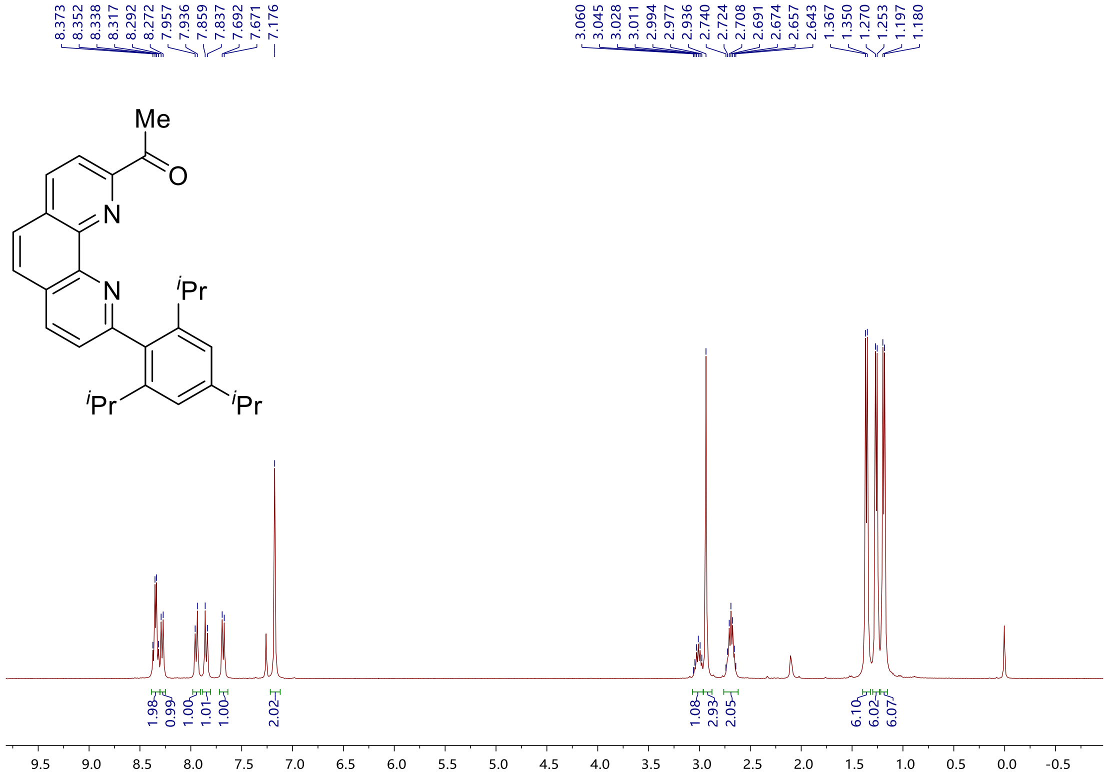

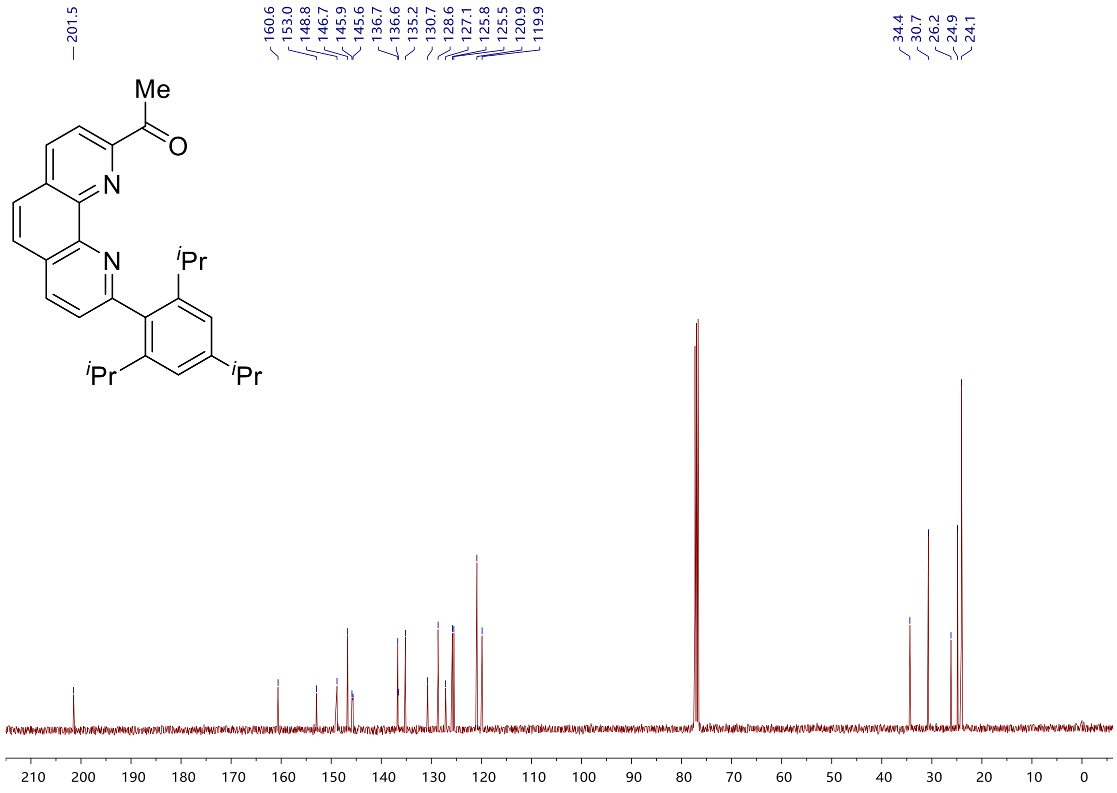

1-(9-(3,5-di-*tert*-butylphenyl)-1,10-phenanthrolin-2-yl)ethan-1-one (3c)

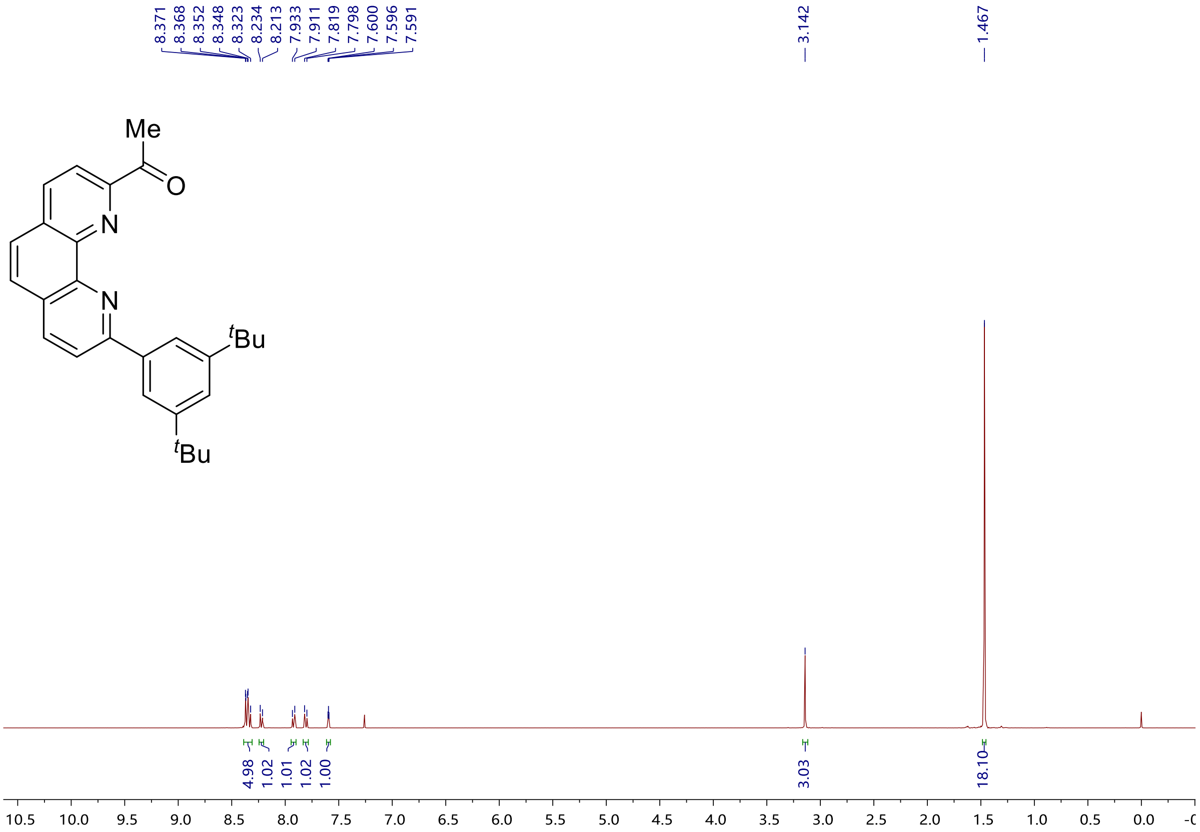

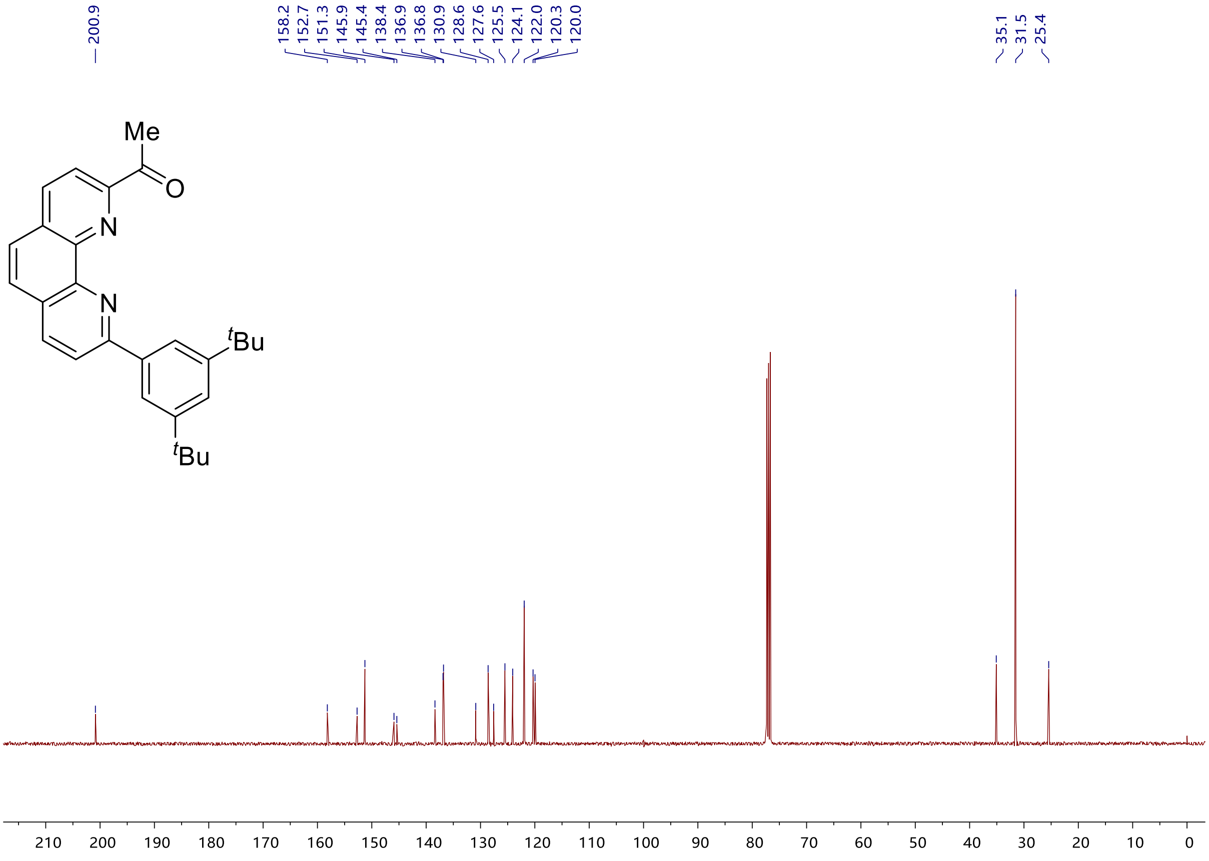

(*E*)-N-(2,6-dimethylphenyl)-1-(9-mesityl-1,10-phenanthrolin-2-yl)ethan-1-imine (L1a)

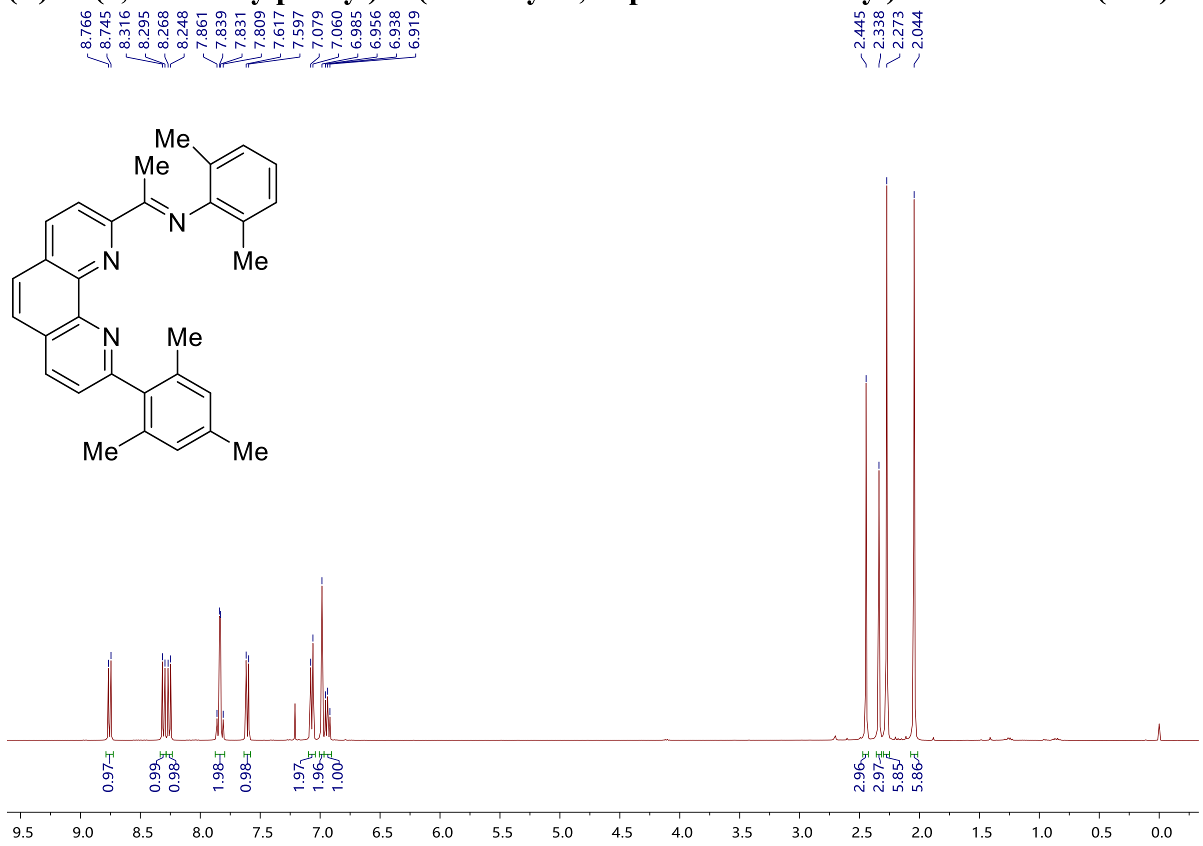

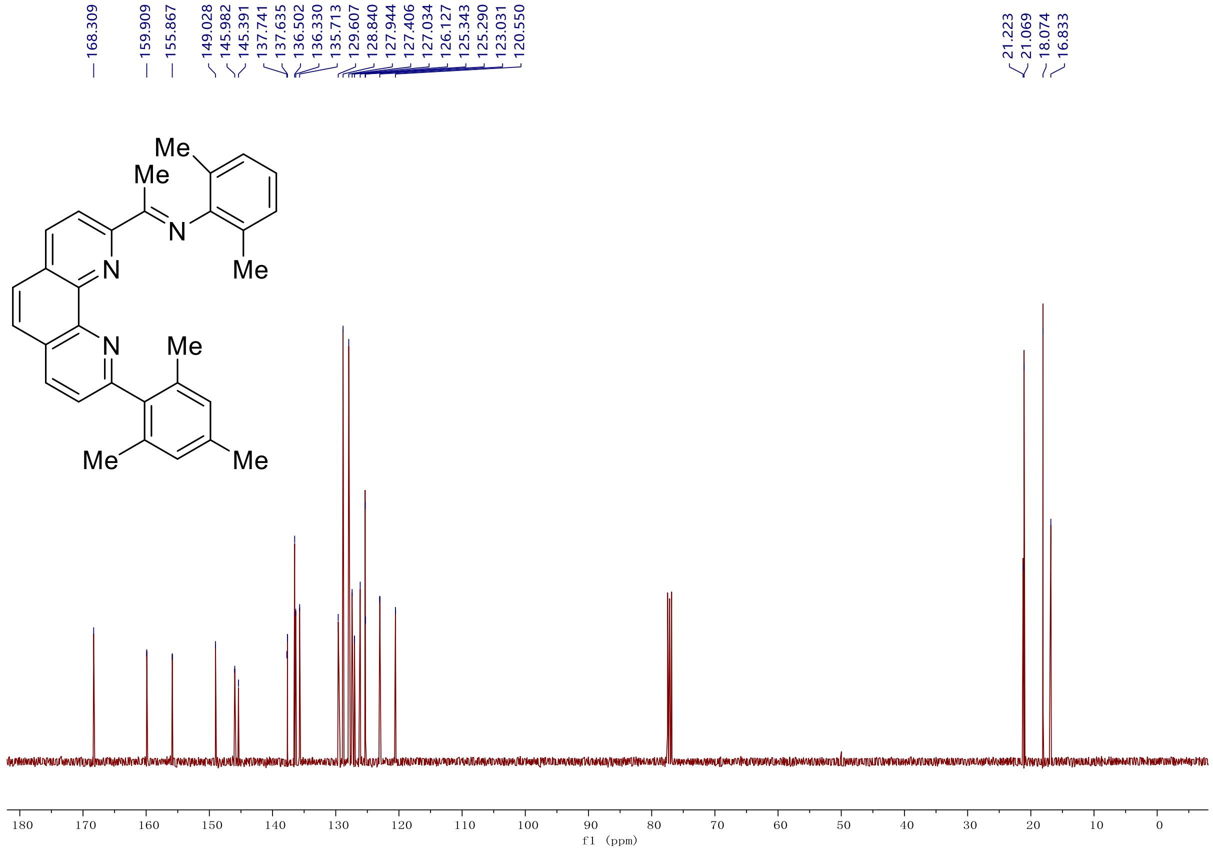

(*E*)-N-(2,6-dimethylphenyl)-1-(9-(2,4,6-triisopropylphenyl)-1,10-phenanthrolin-2-yl)ethan-1-imine (L1b)

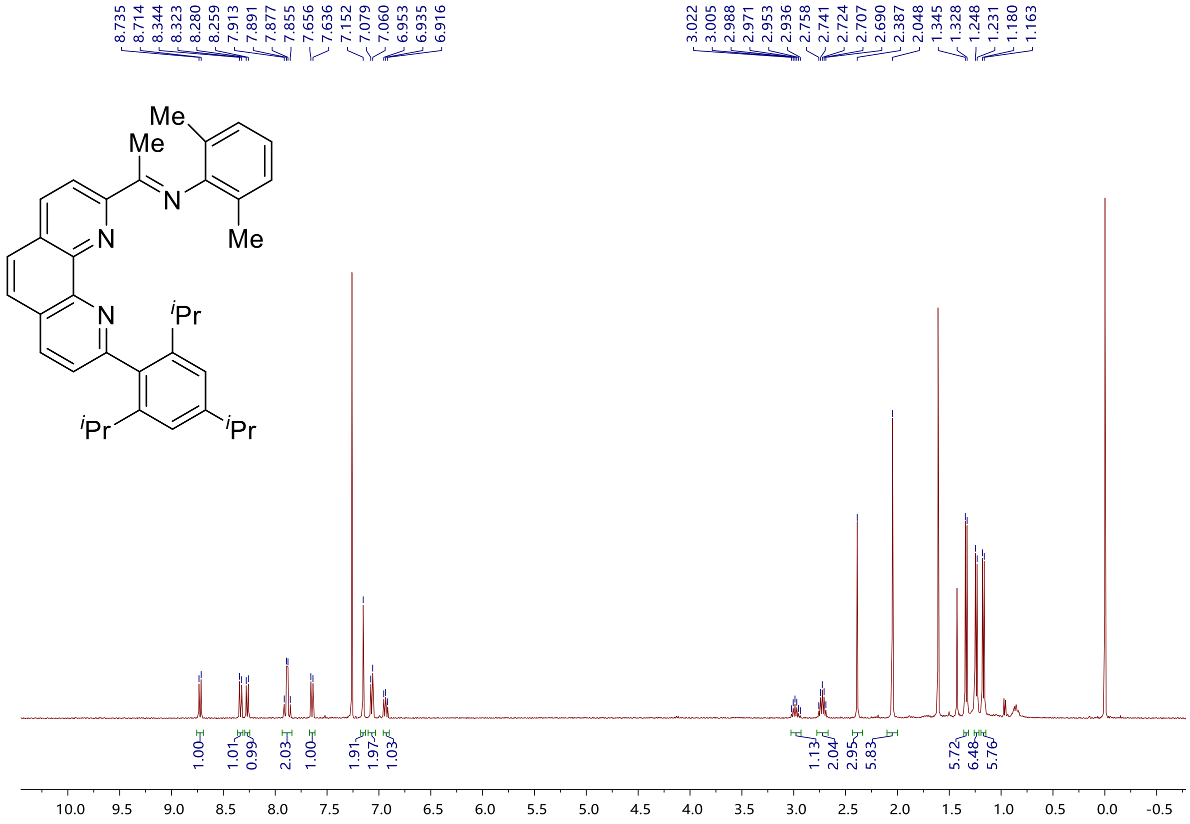

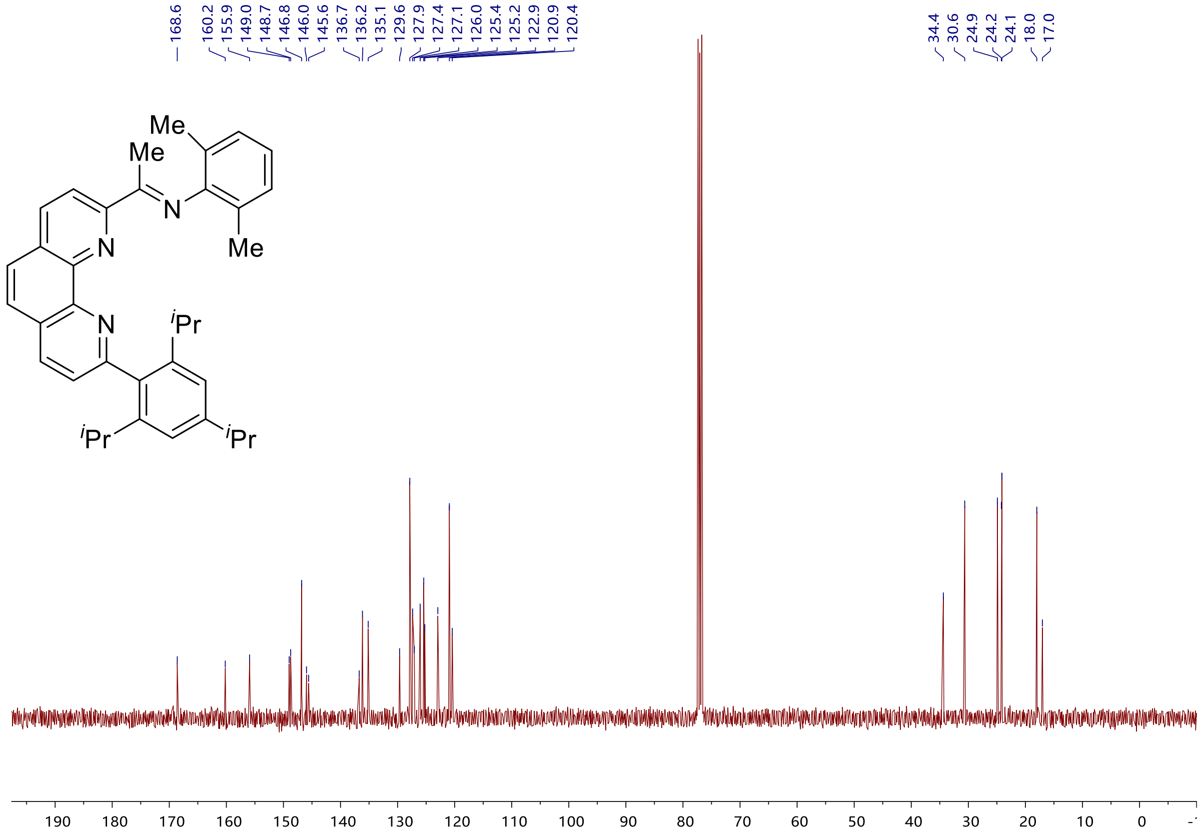

(*E*)-N-(2,6-diethylphenyl)-1-(9-(2,4,6-triisopropylphenyl)-1,10-phenanthrolin-2-yl)ethan-1-imine (L1c)

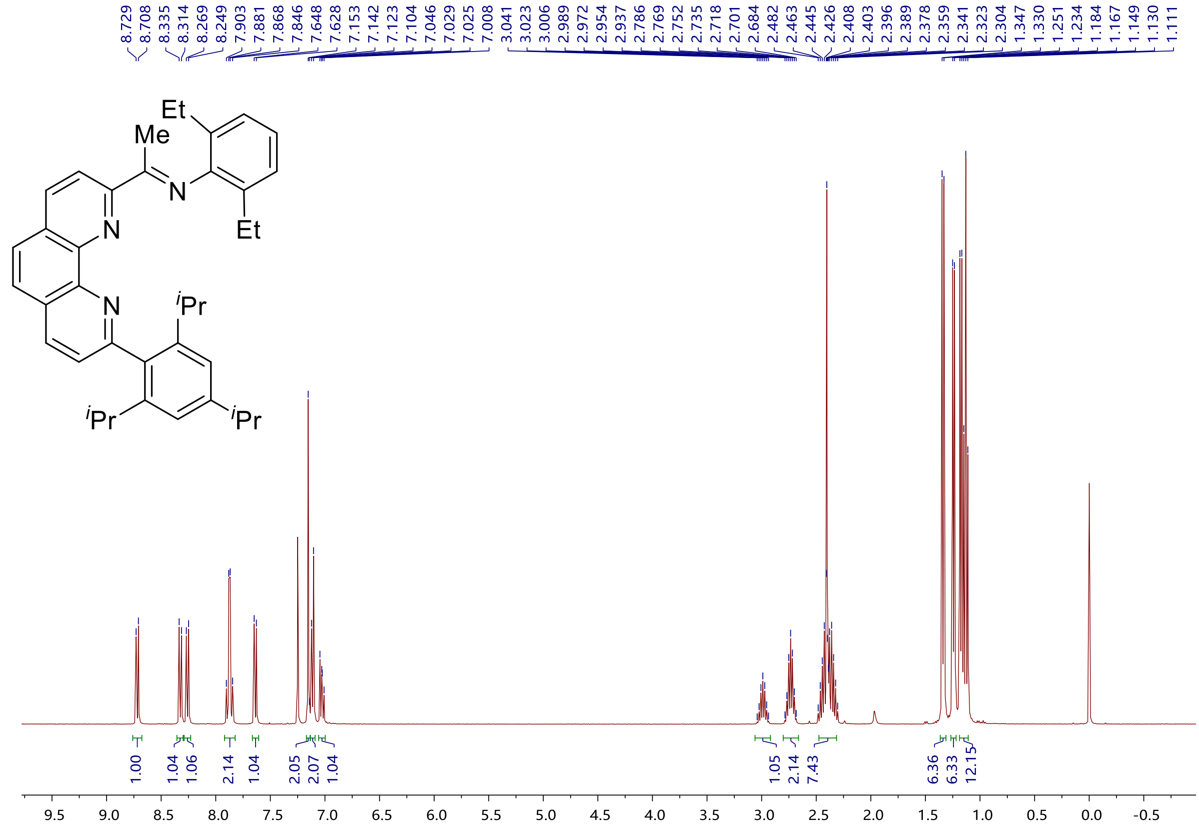

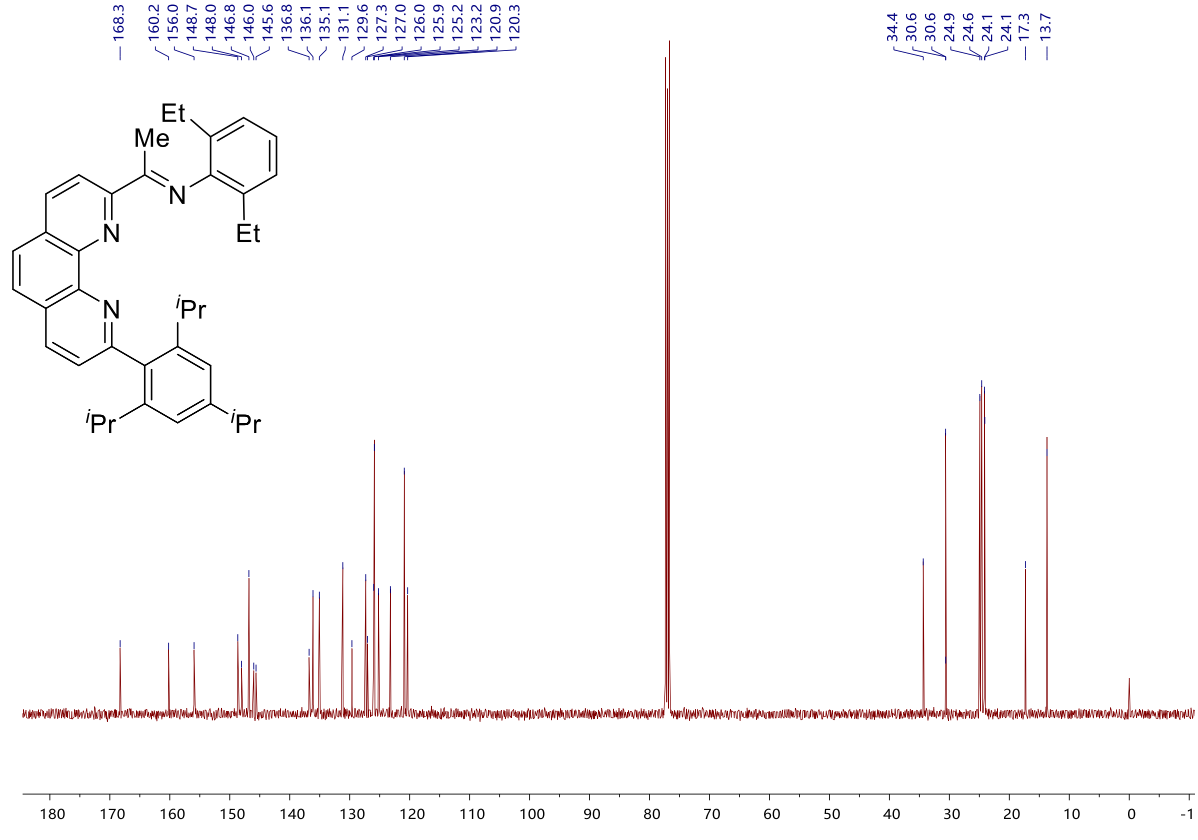

(*E*)-N-(2,6-diisopropylphenyl)-1-(9-(2,4,6-triisopropylphenyl)-1,10-phenanthrolin-2-yl)ethan-1-imine (L1d)

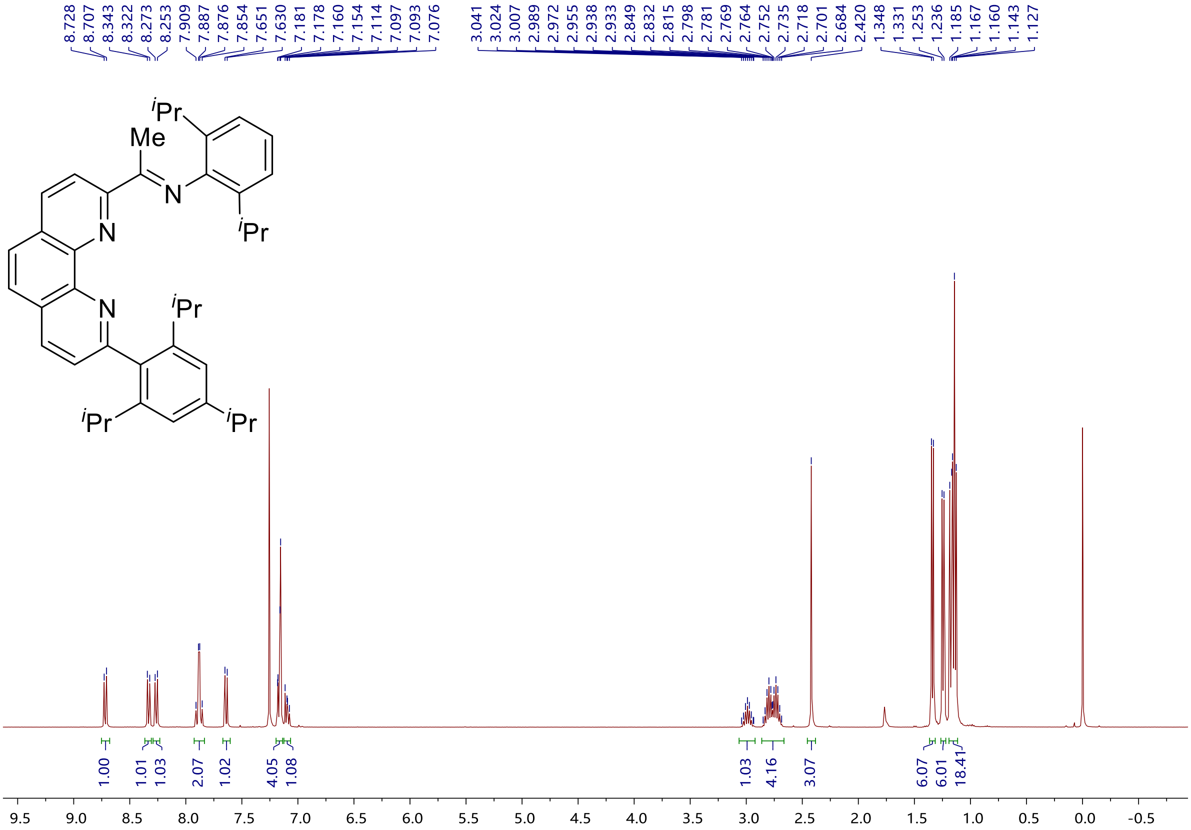

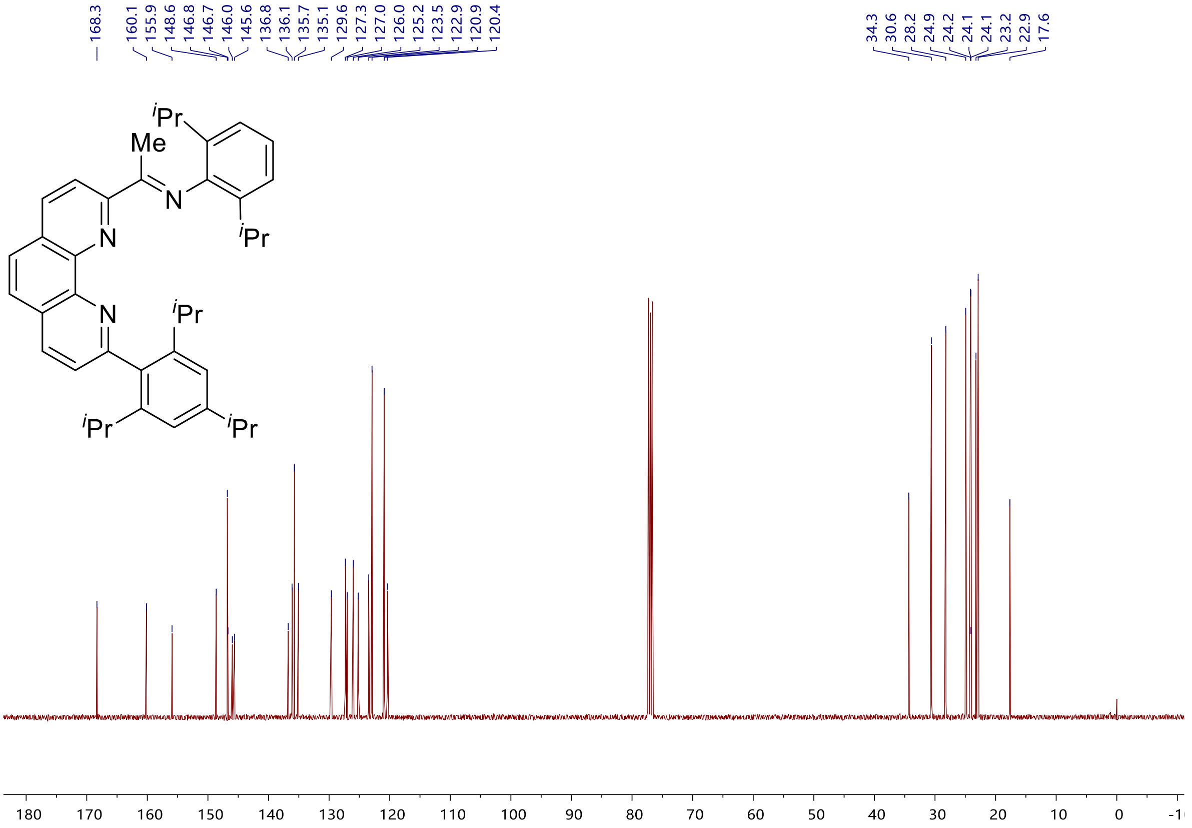

(*E*)-N-(2,6-dibenzhydryl-4-methoxyphenyl)-1-(9-(2,4,6-triisopropylphenyl)-1,10-phenanthrolin-2-yl)ethan-1-imine (L1e)

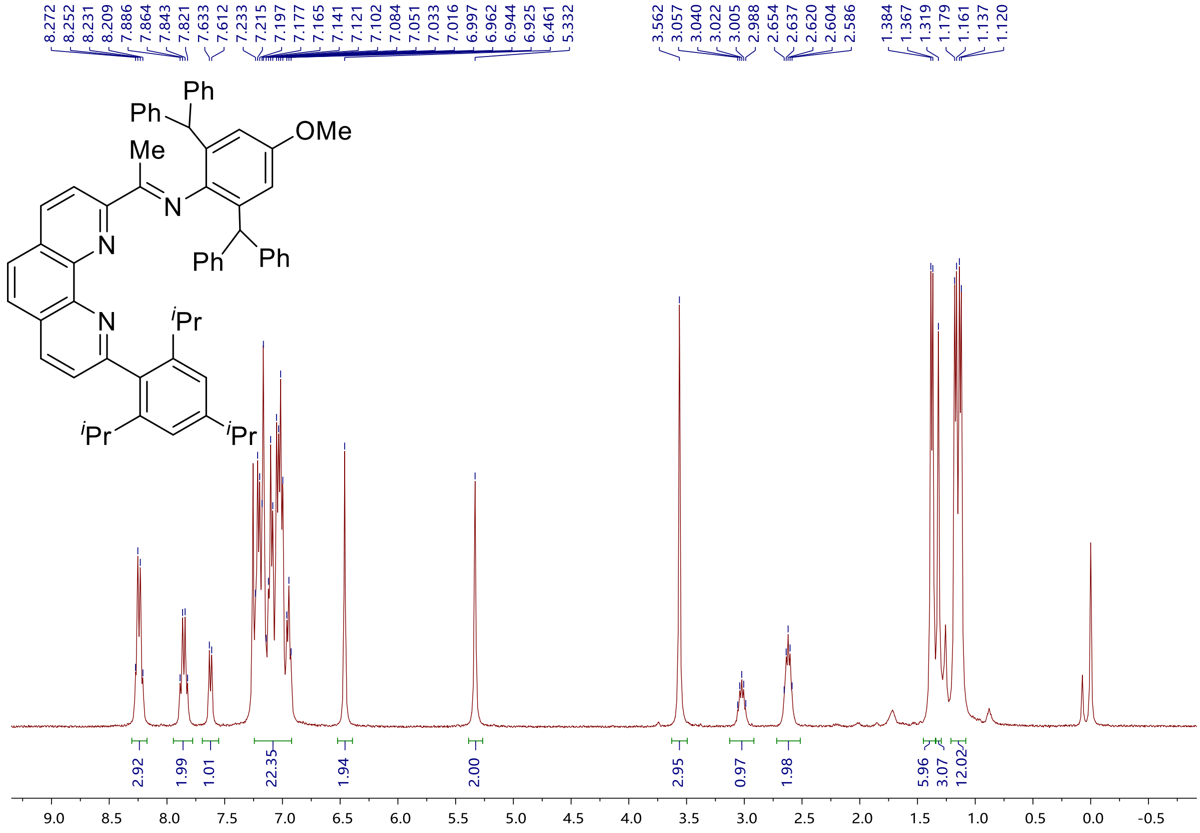

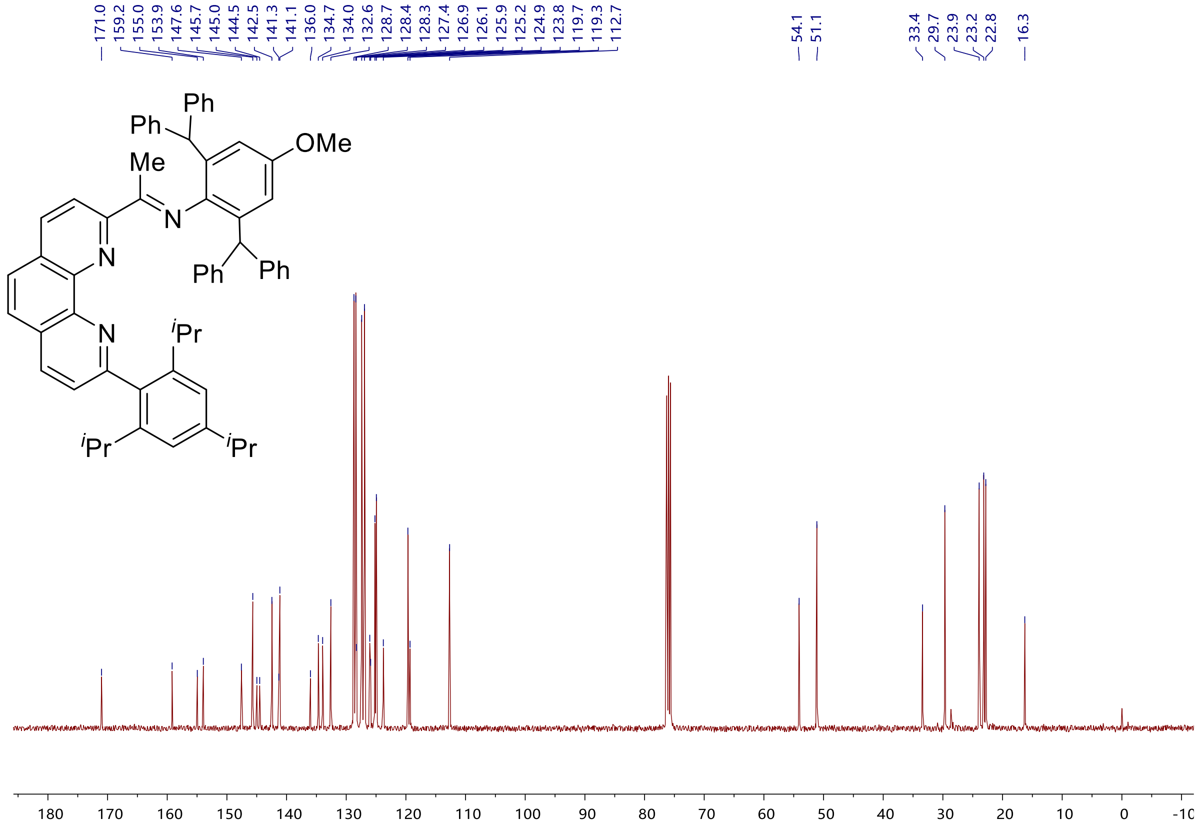

(*E*)-1-(9-(3,5-di-*tert*-butylphenyl)-1,10-phenanthrolin-2-yl)-N-(2,6-dimethylphenyl)ethan-1-imine (L1f)

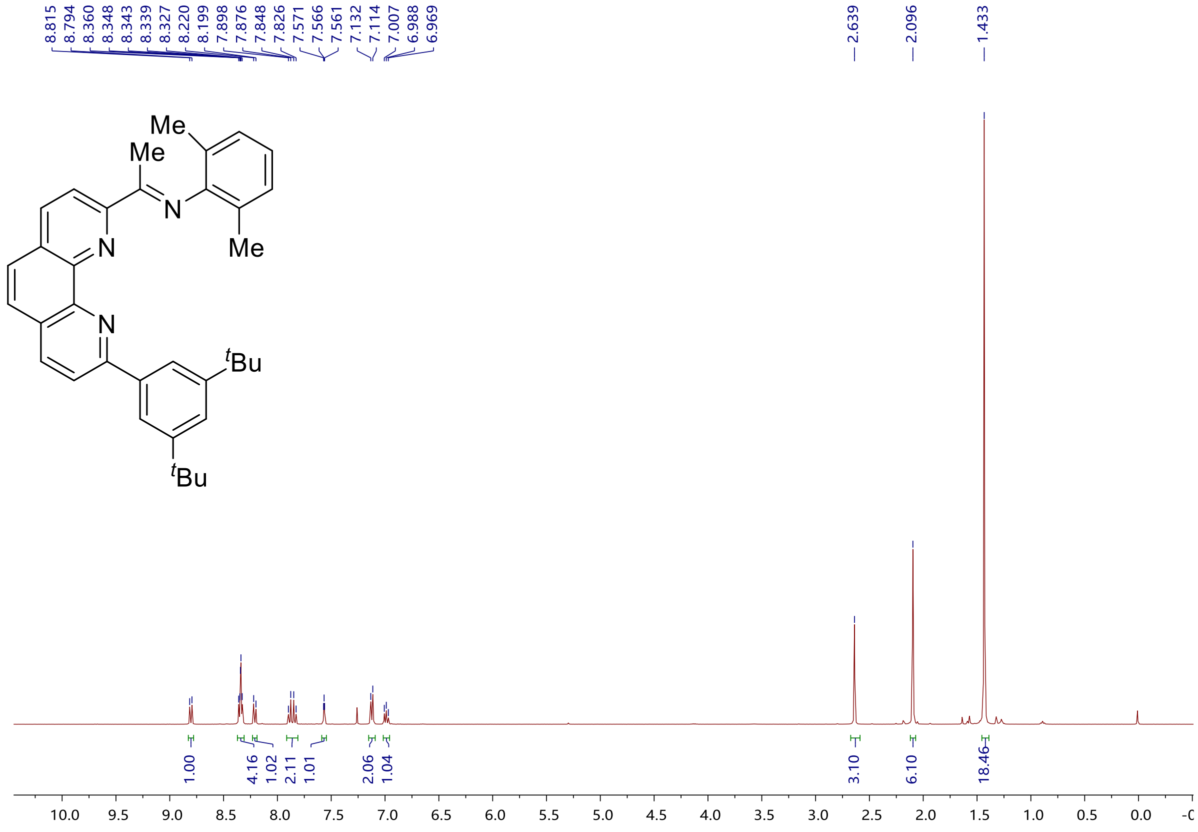

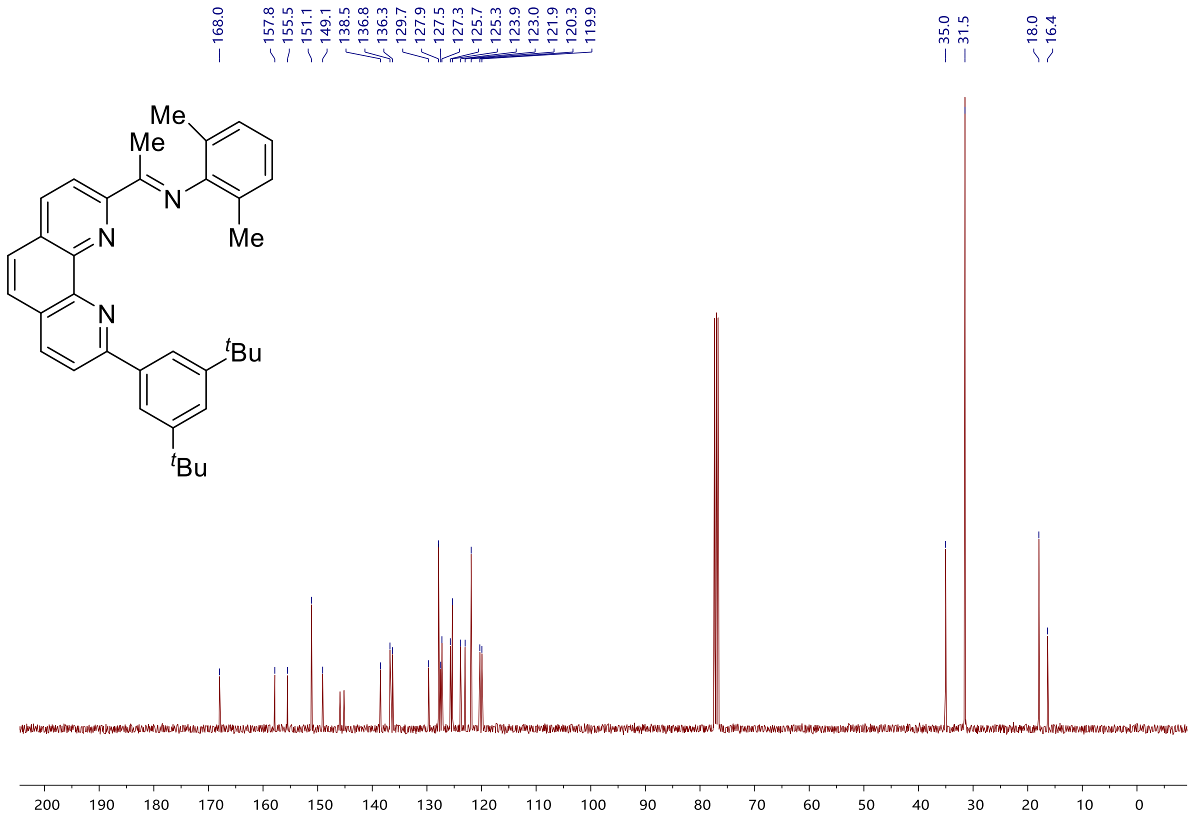

(*E*)-1-(9-(3,5-di-*tert*-butylphenyl)-1,10-phenanthrolin-2-yl)-N-(2,6-diisopropylphenyl)ethan-1-imine (L1g)

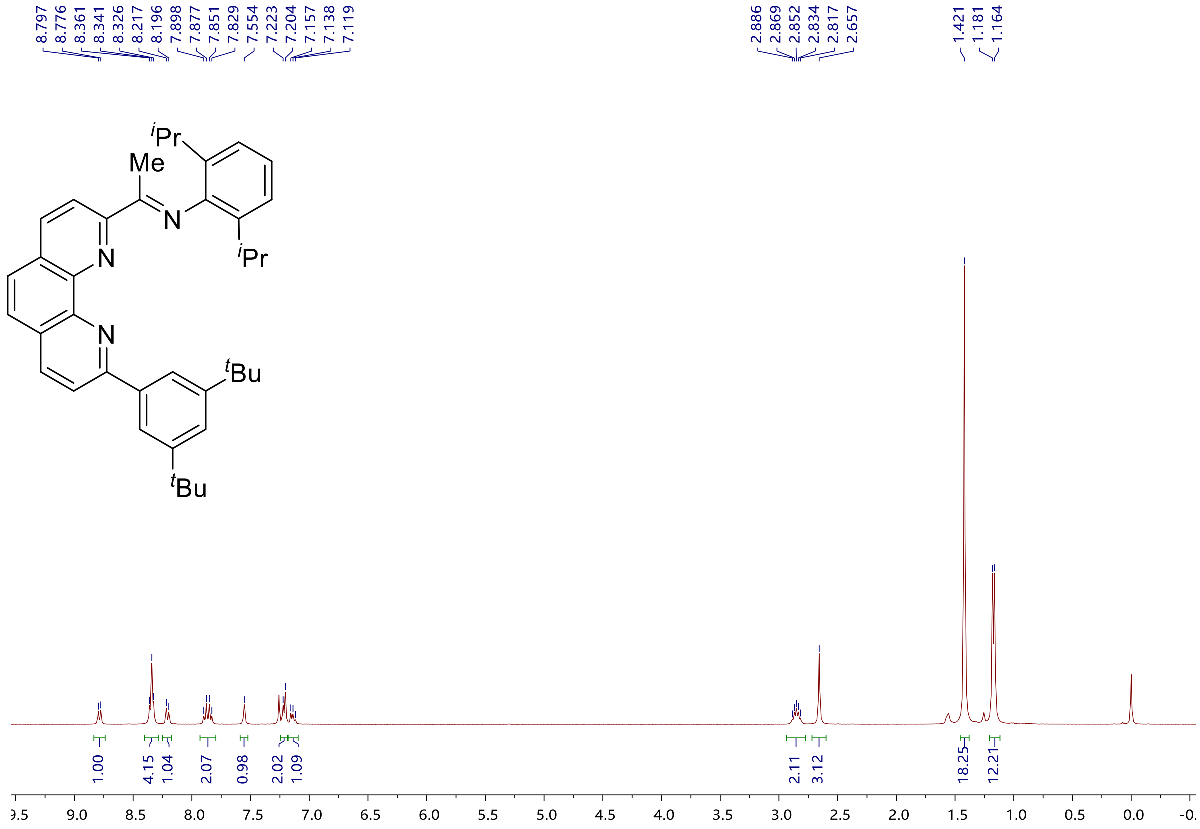

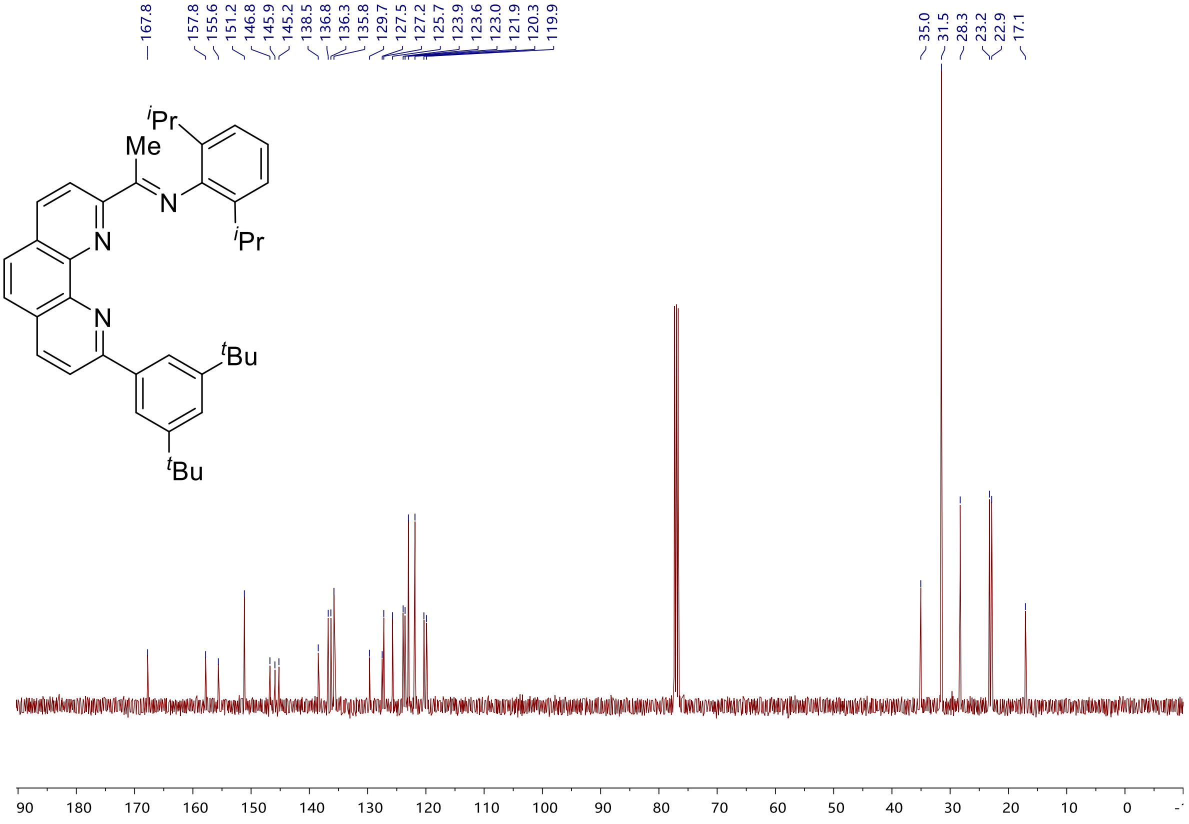

(*E*)-phenyl(4-phenylbut-3-en-1-yl)silane (3aa)

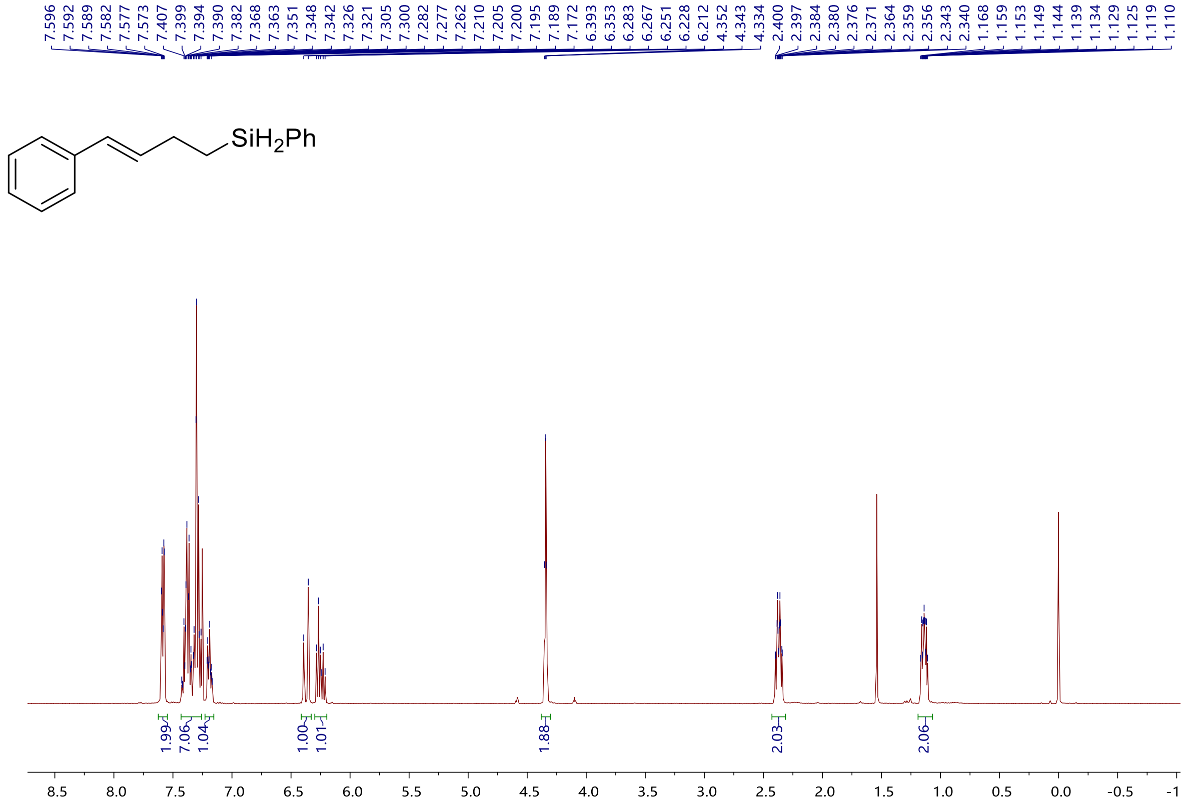

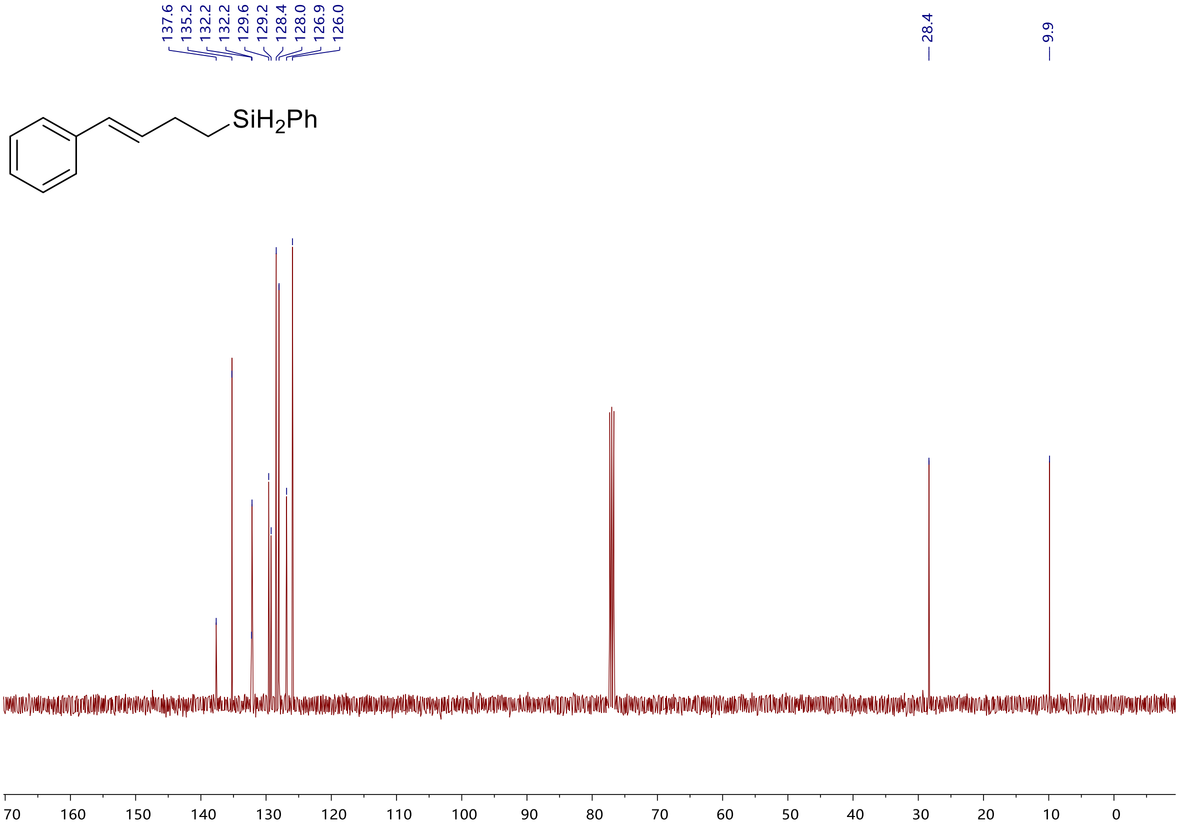

(*E*)-(4-phenylbut-3-en-1-yl)(o-tolyl)silane (3ab)

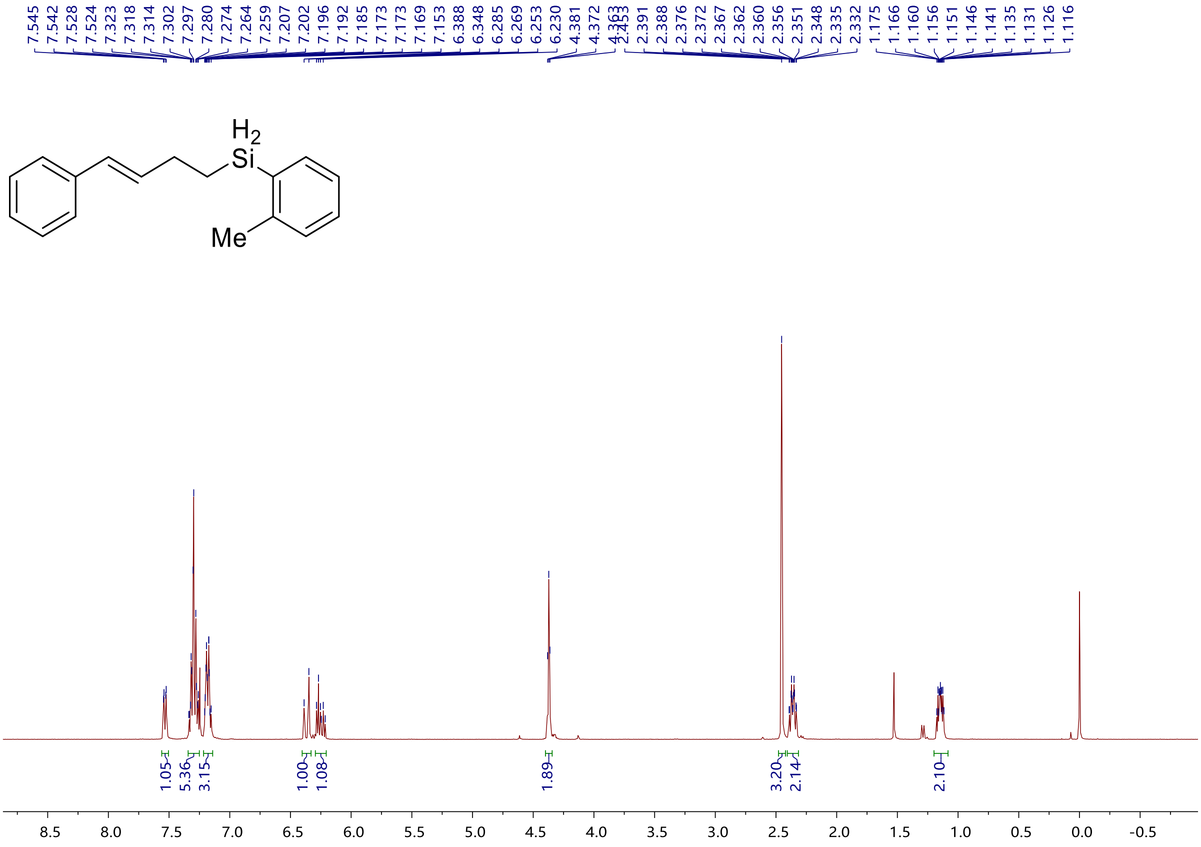

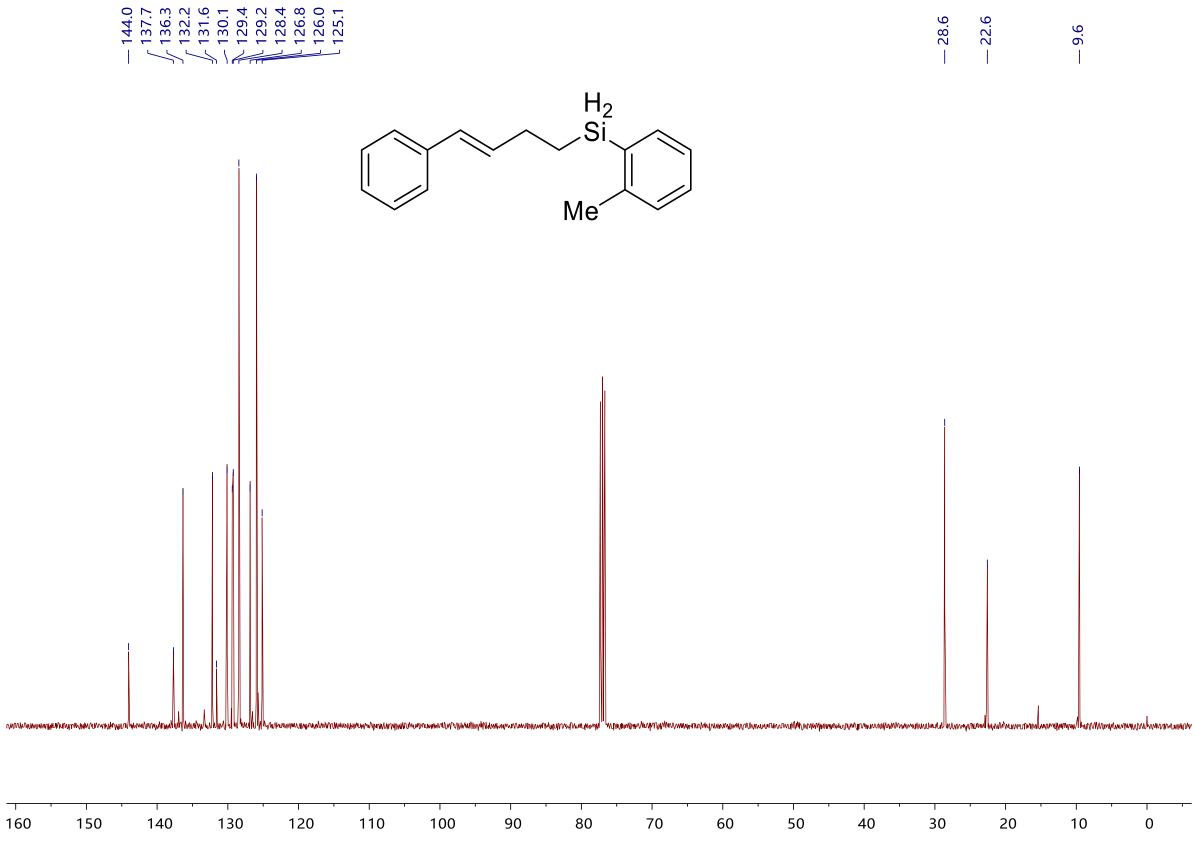

(*E*)-(4-phenylbut-3-en-1-yl)(m-tolyl)silane (3ac)

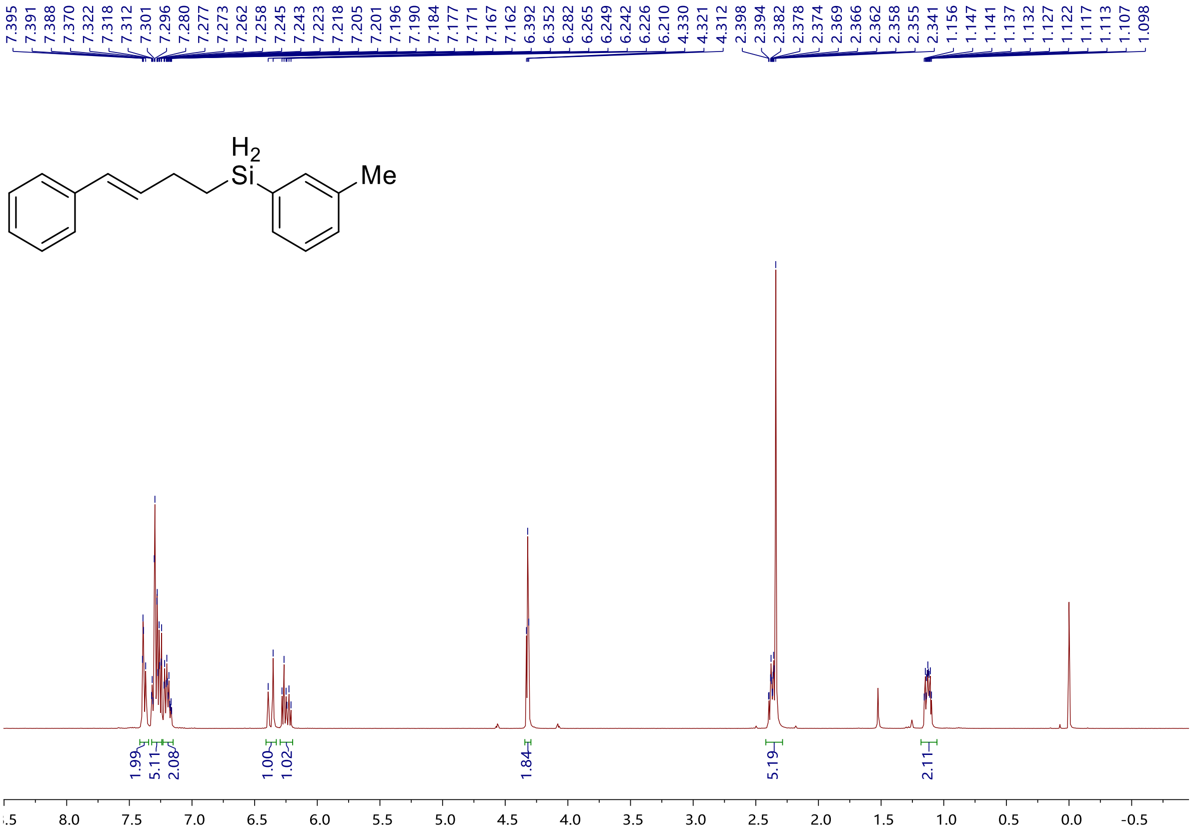

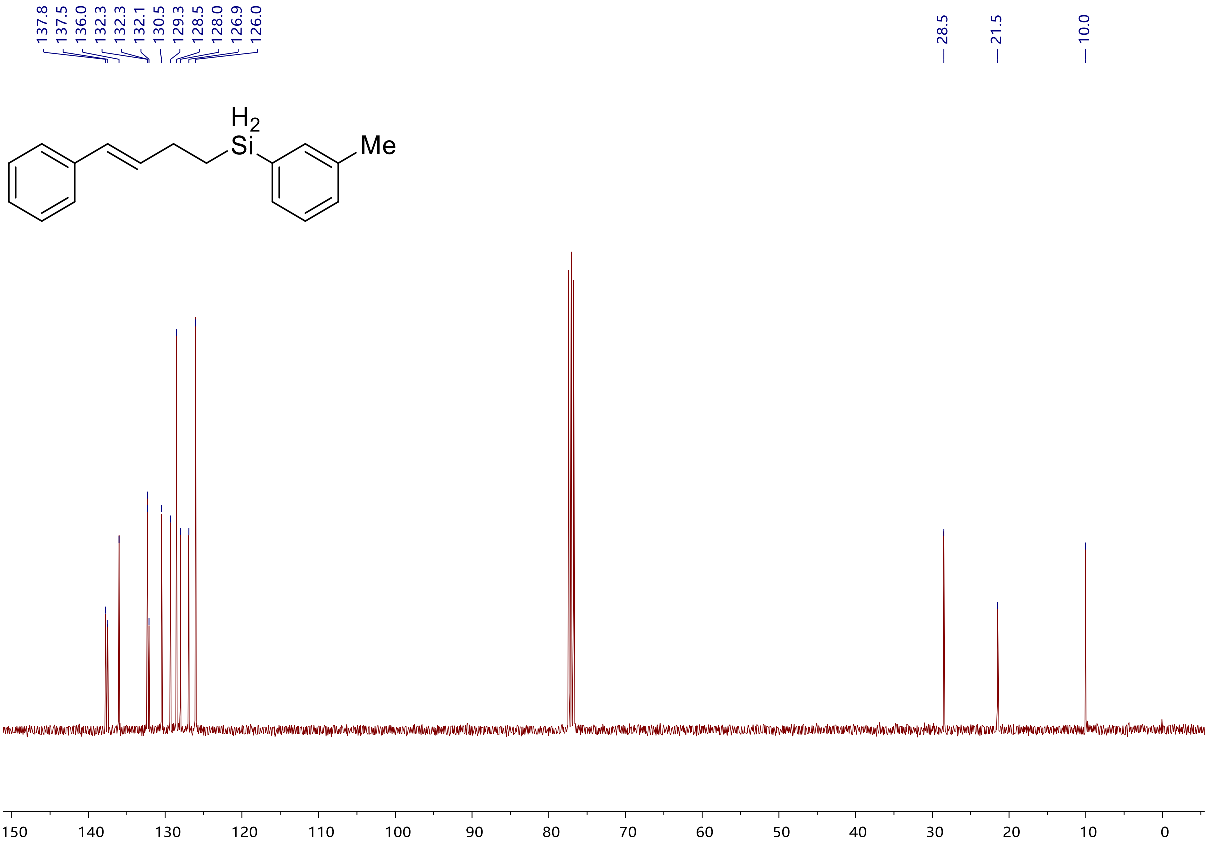

(*E*)-(4-methoxyphenyl)(4-phenylbut-3-en-1-yl)silane (3ad)

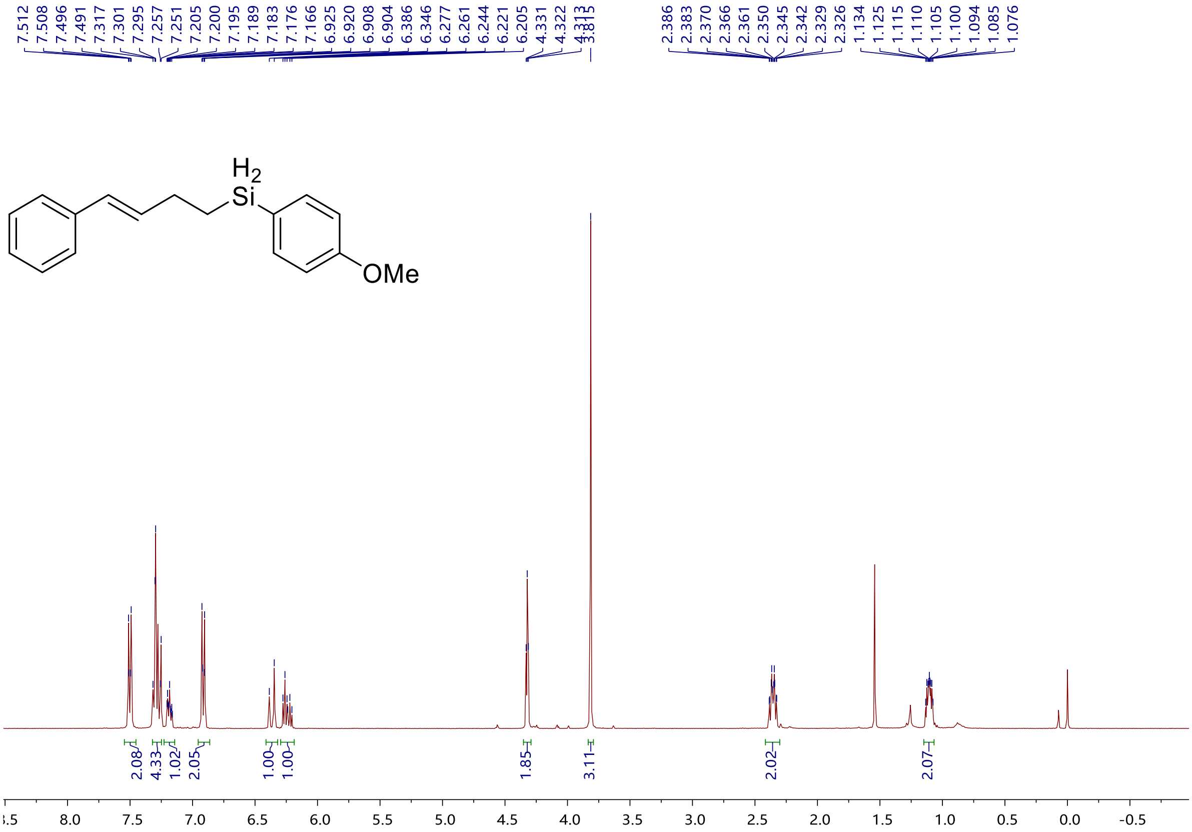

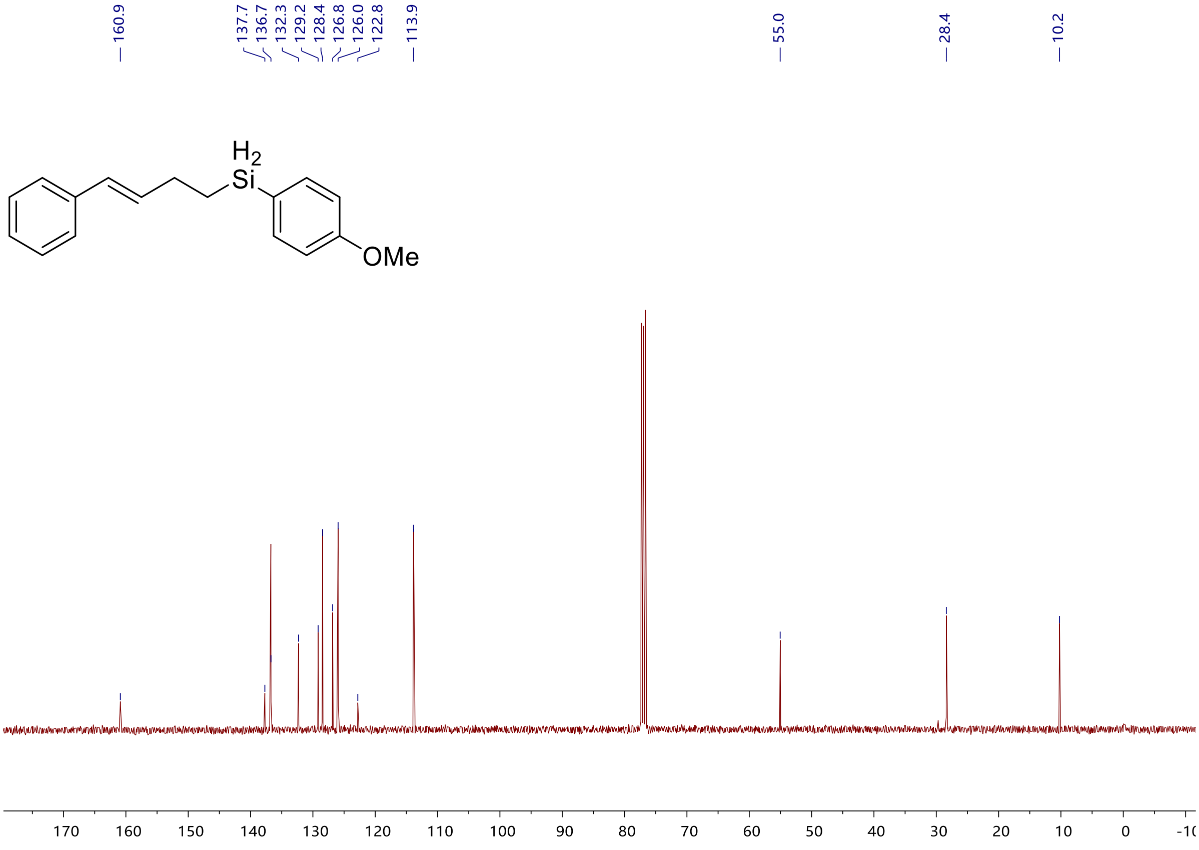

(*E*)-(4-(*tert*-butyl)phenyl)(4-phenylbut-3-en-1-yl)silane (3ae)

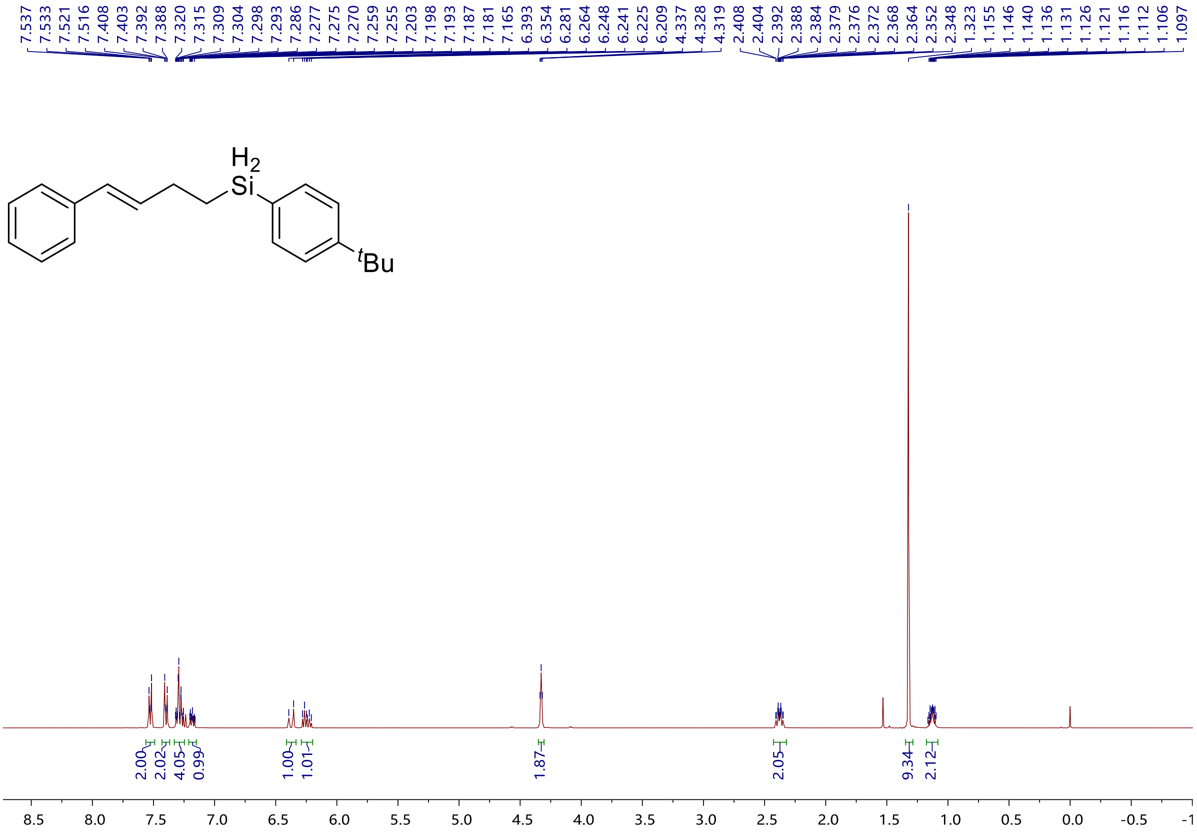

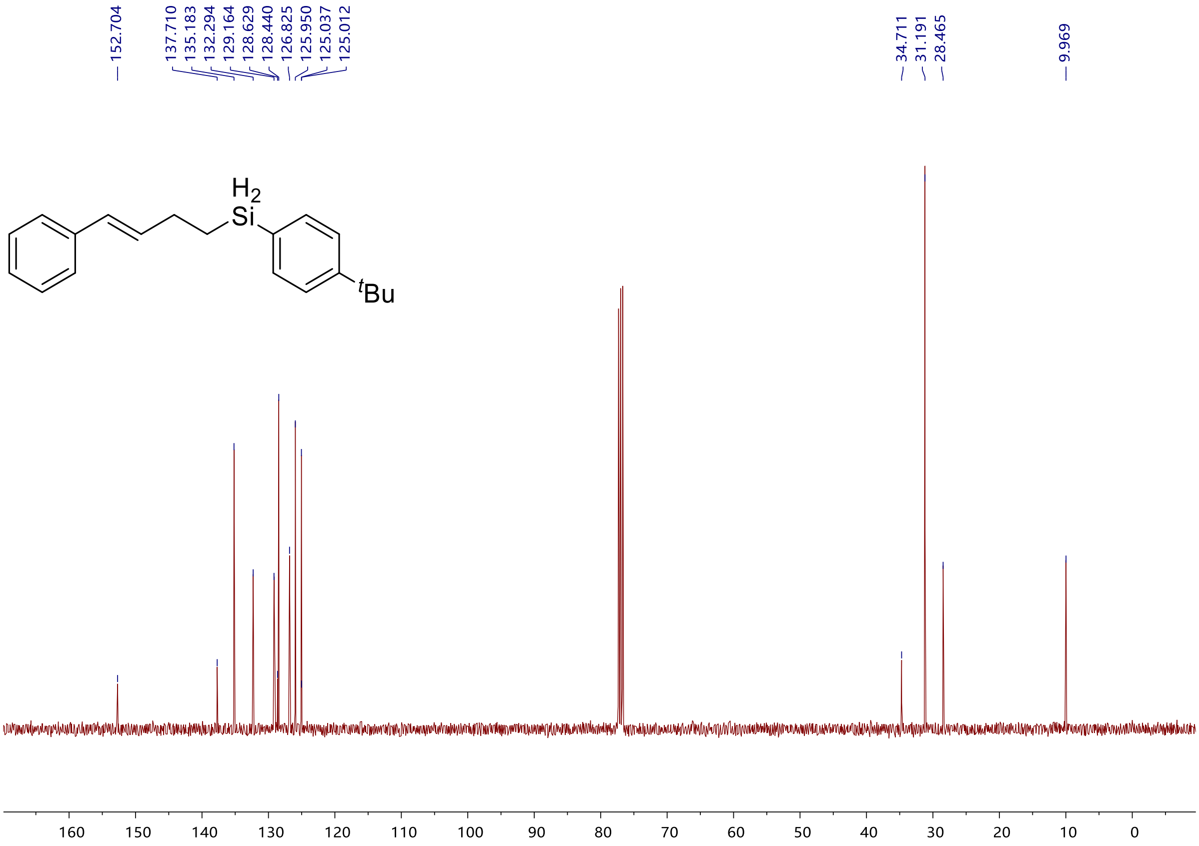

(*E*)-(4-fluorophenyl)(4-phenylbut-3-en-1-yl)silane (3af)

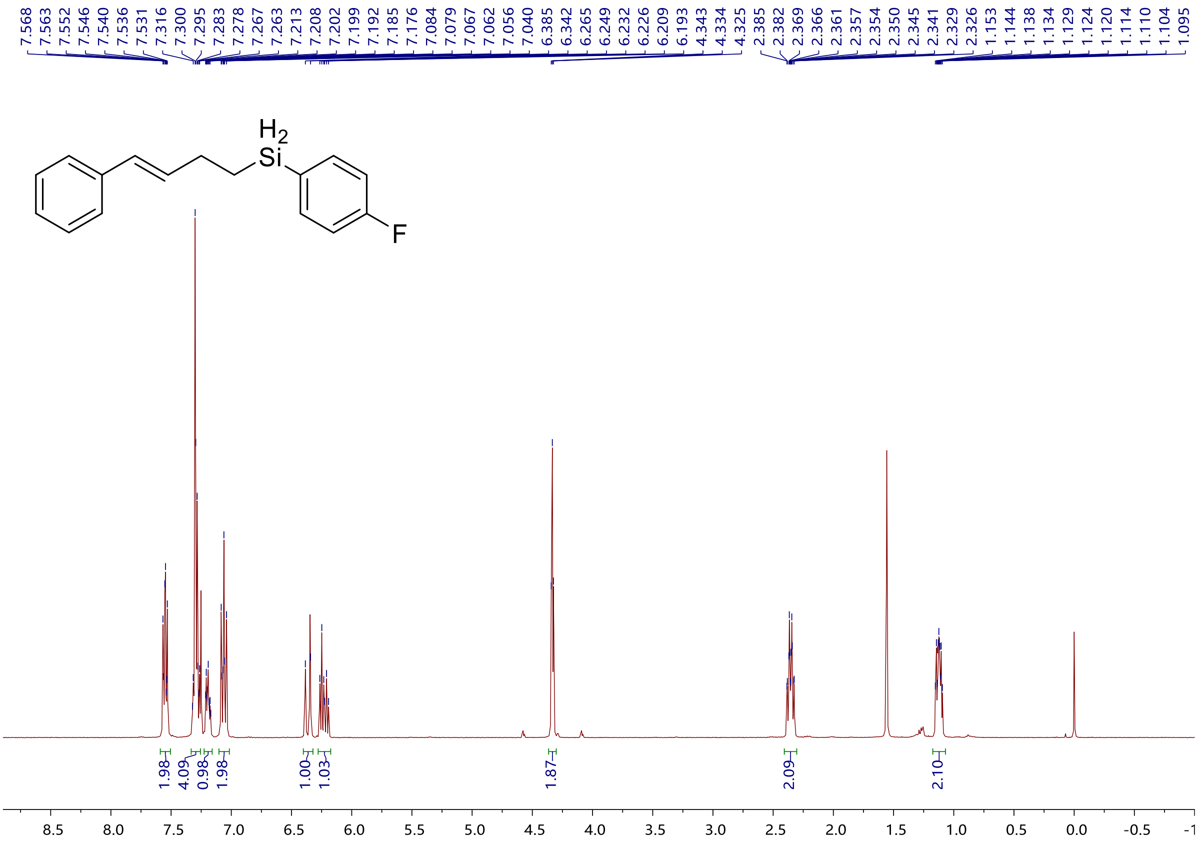

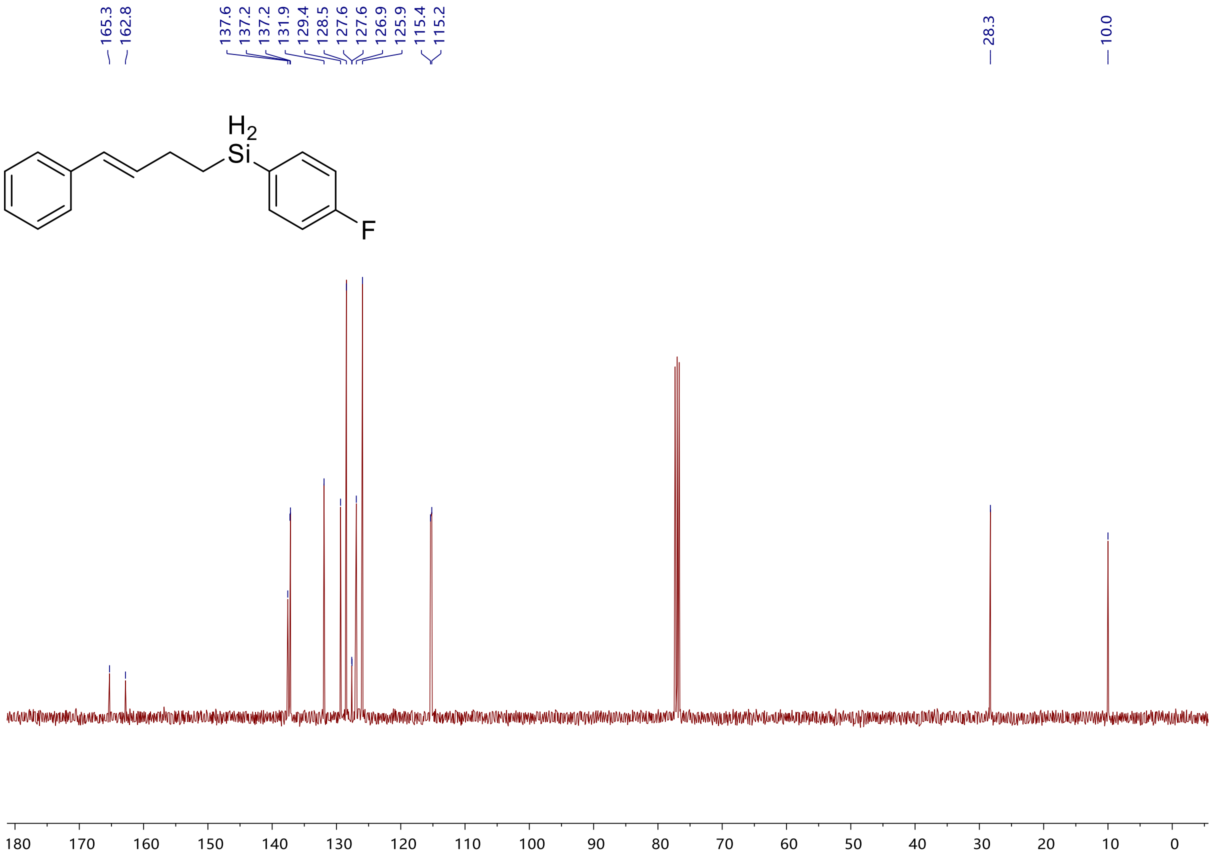

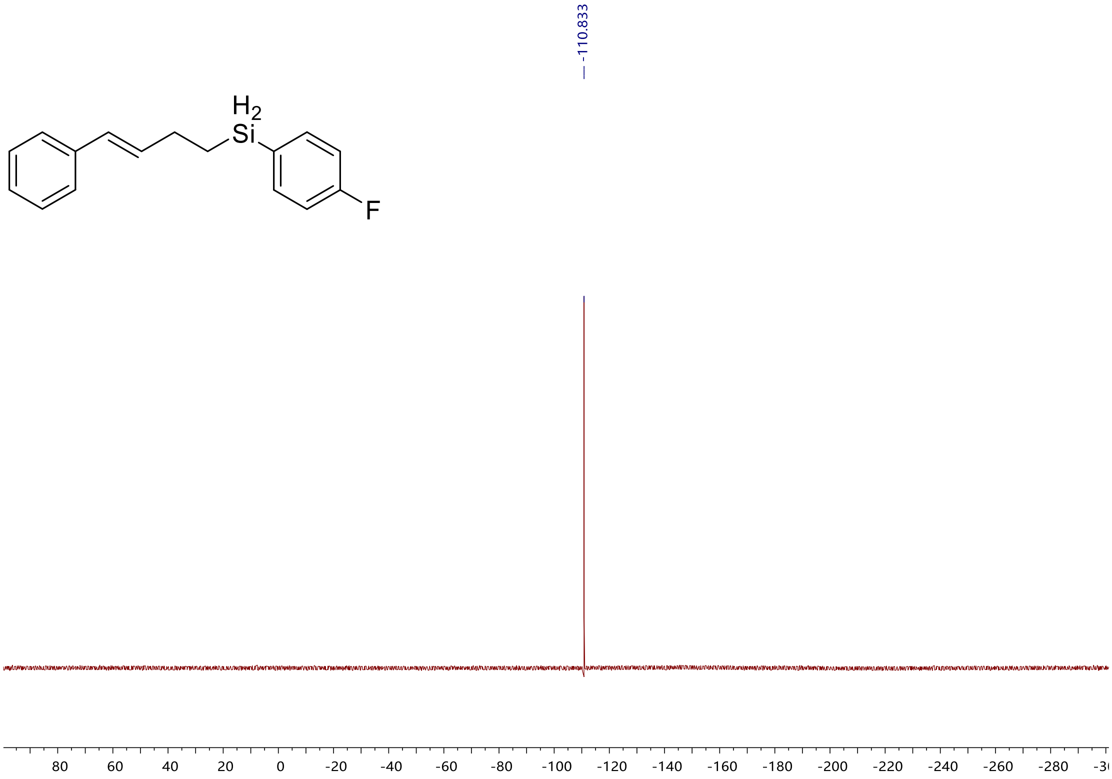

-110.833
H2
Si
F
80
60
40
20
0
-20
-40
-60
-80
-100
-120
-140
-160
-180
-200
-220
-240
-260
-280

(*E*)-(4-chlorophenyl)(4-phenylbut-3-en-1-yl)silane (3ag)

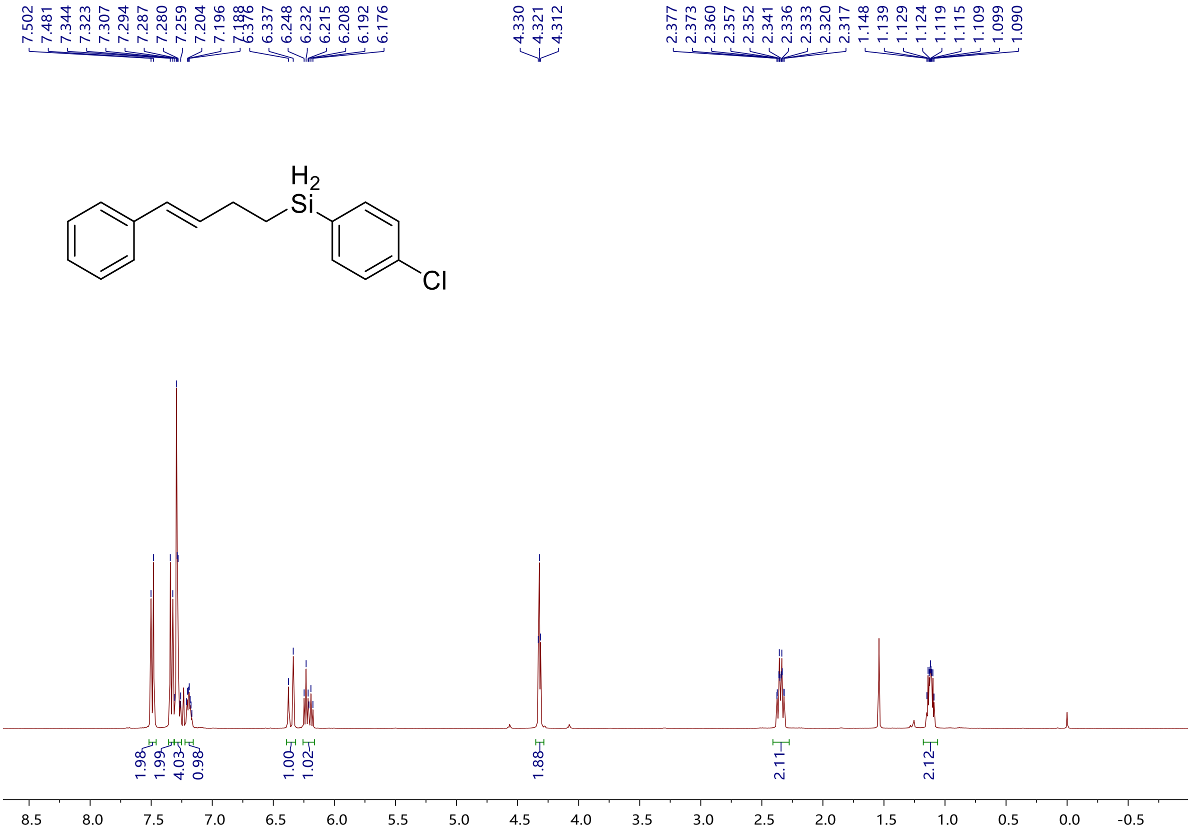

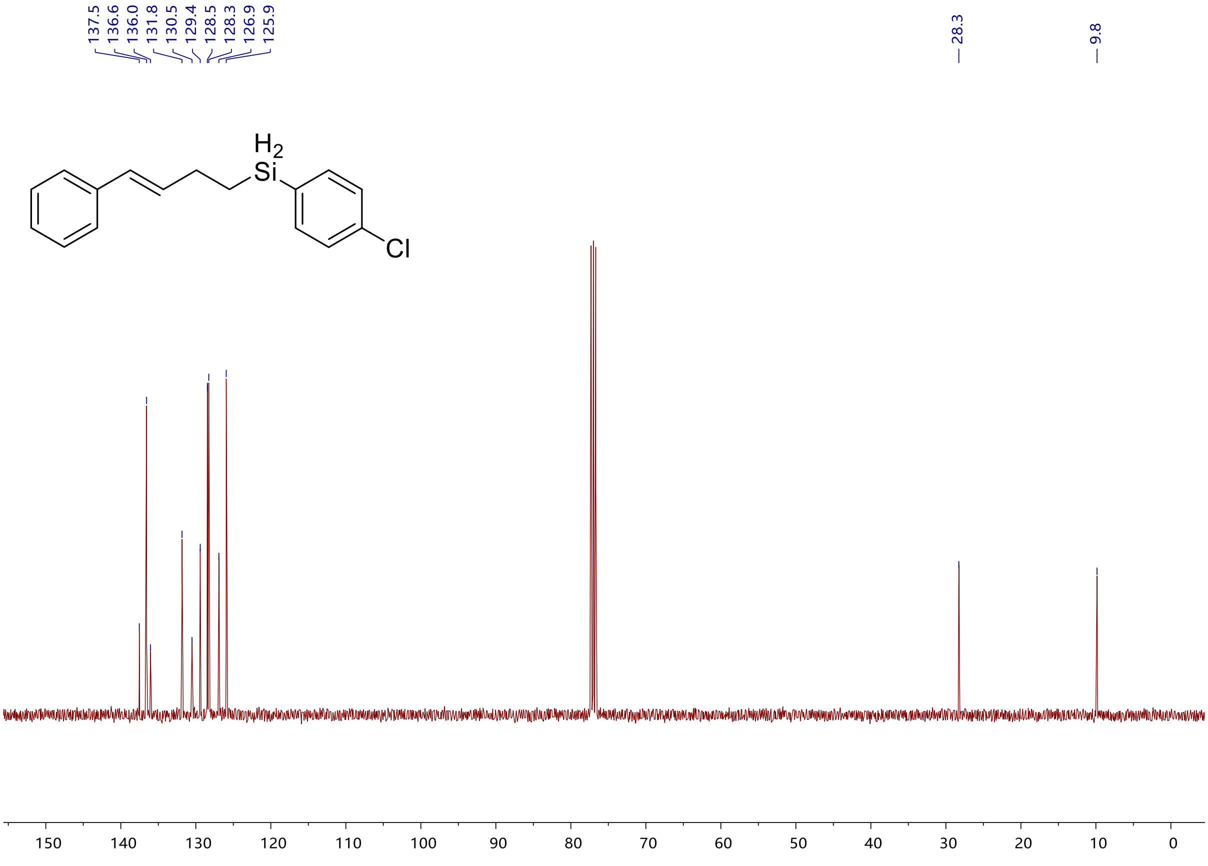

(*E*)-(4-methylbenzyl)(4-phenylbut-3-en-1-yl)silane (3ah)

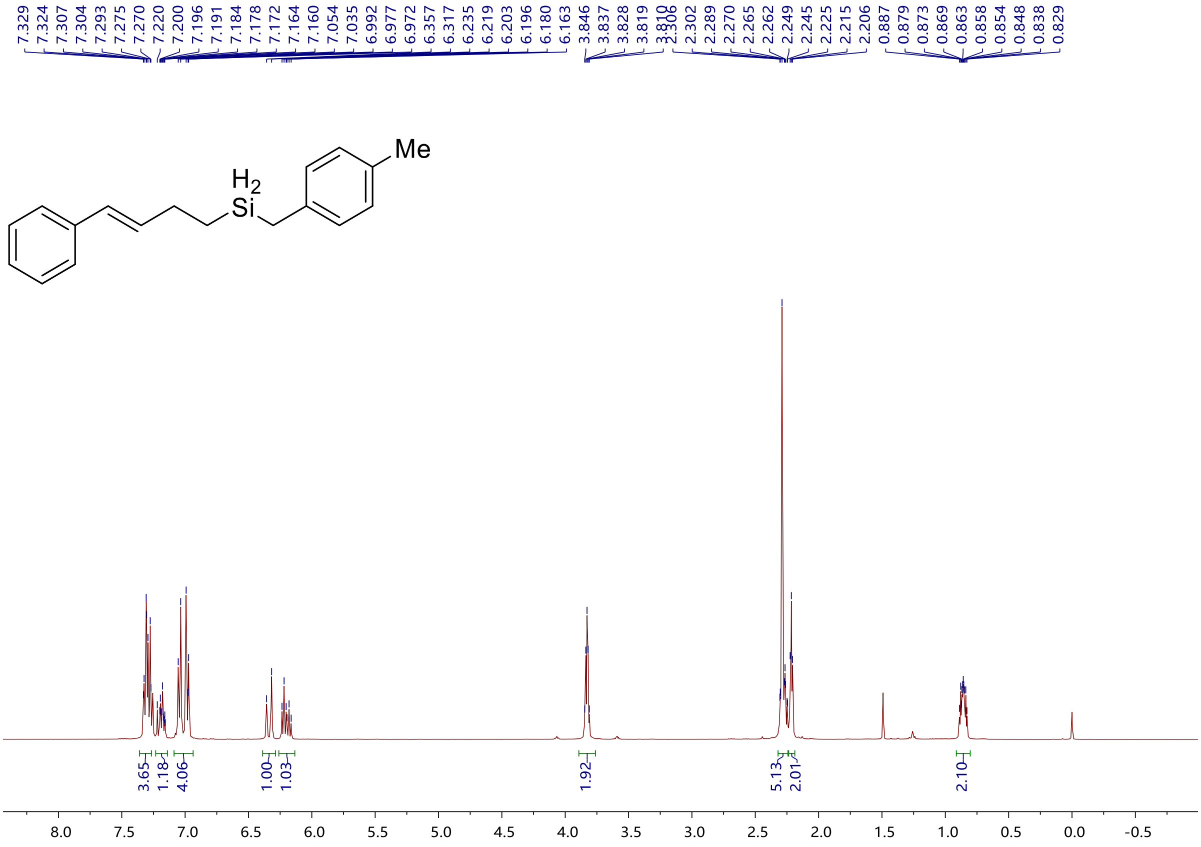

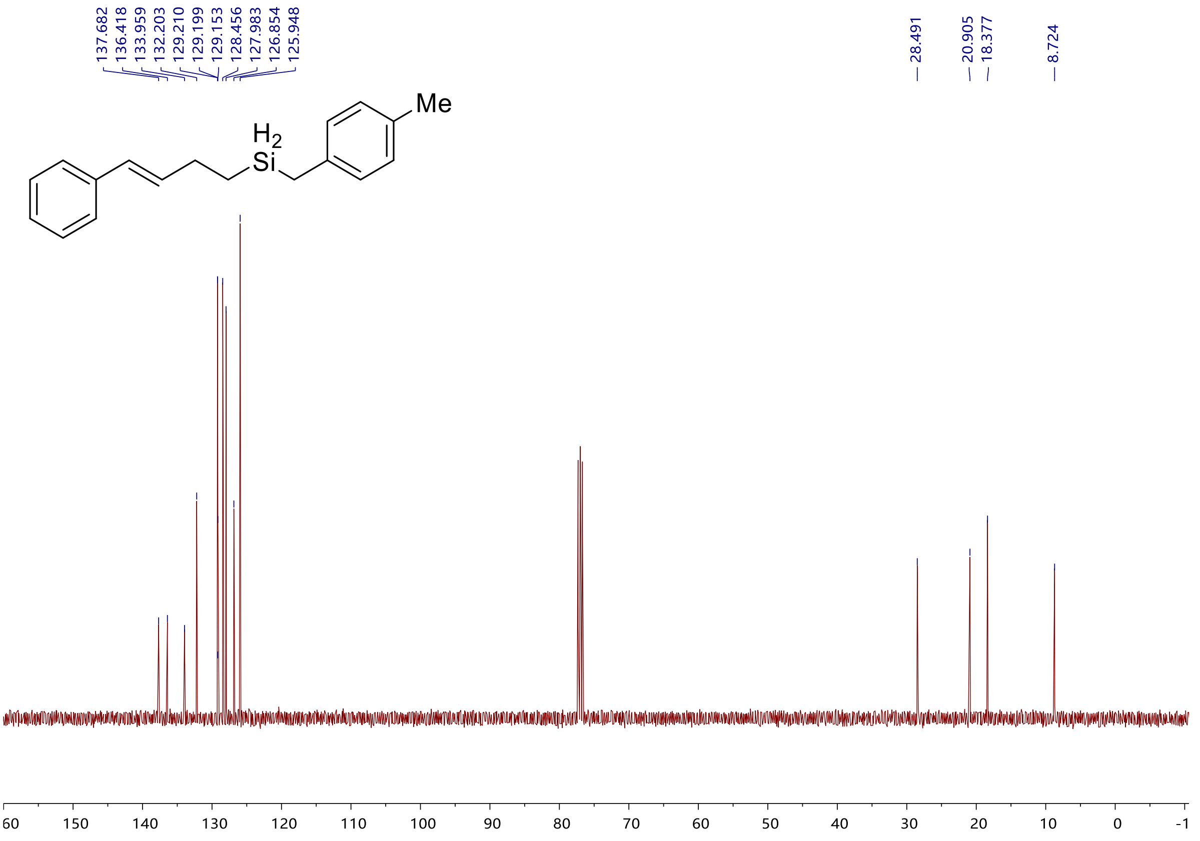

(*E*)-octyl(4-phenylbut-3-en-1-yl)silane (3ai)

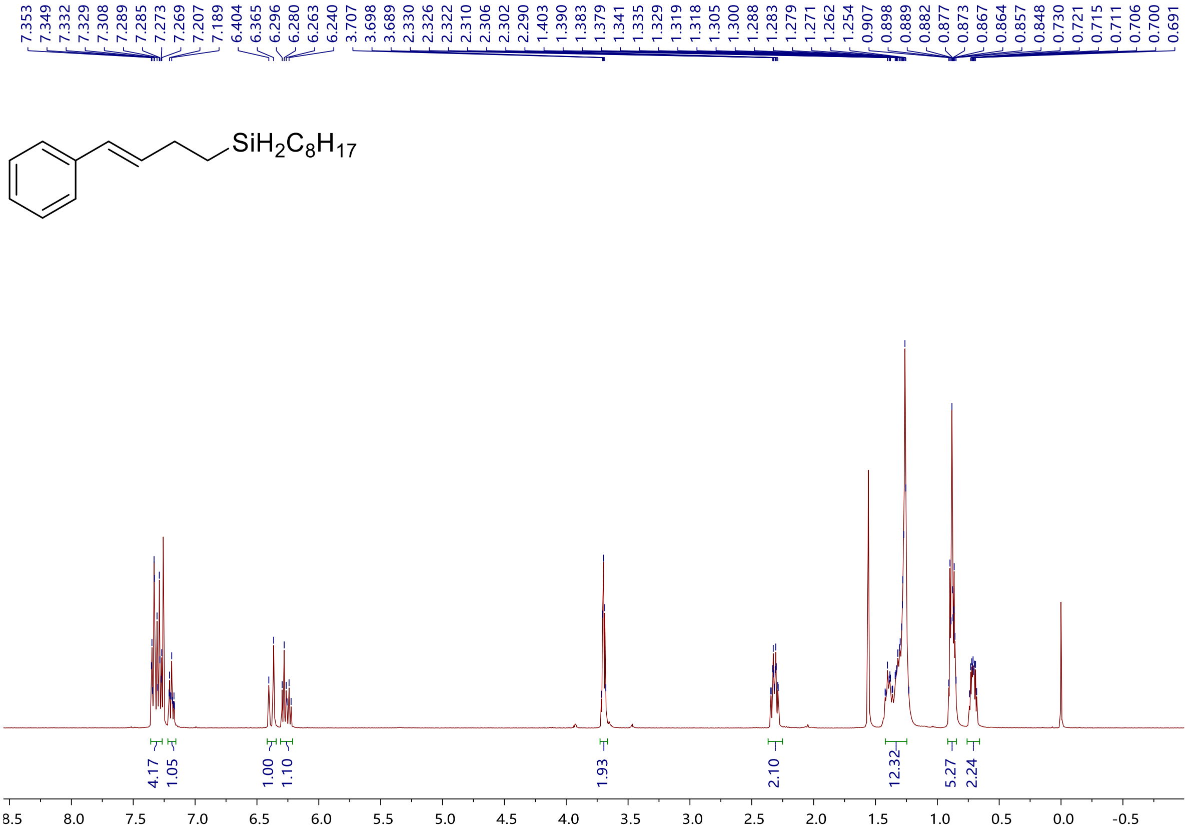

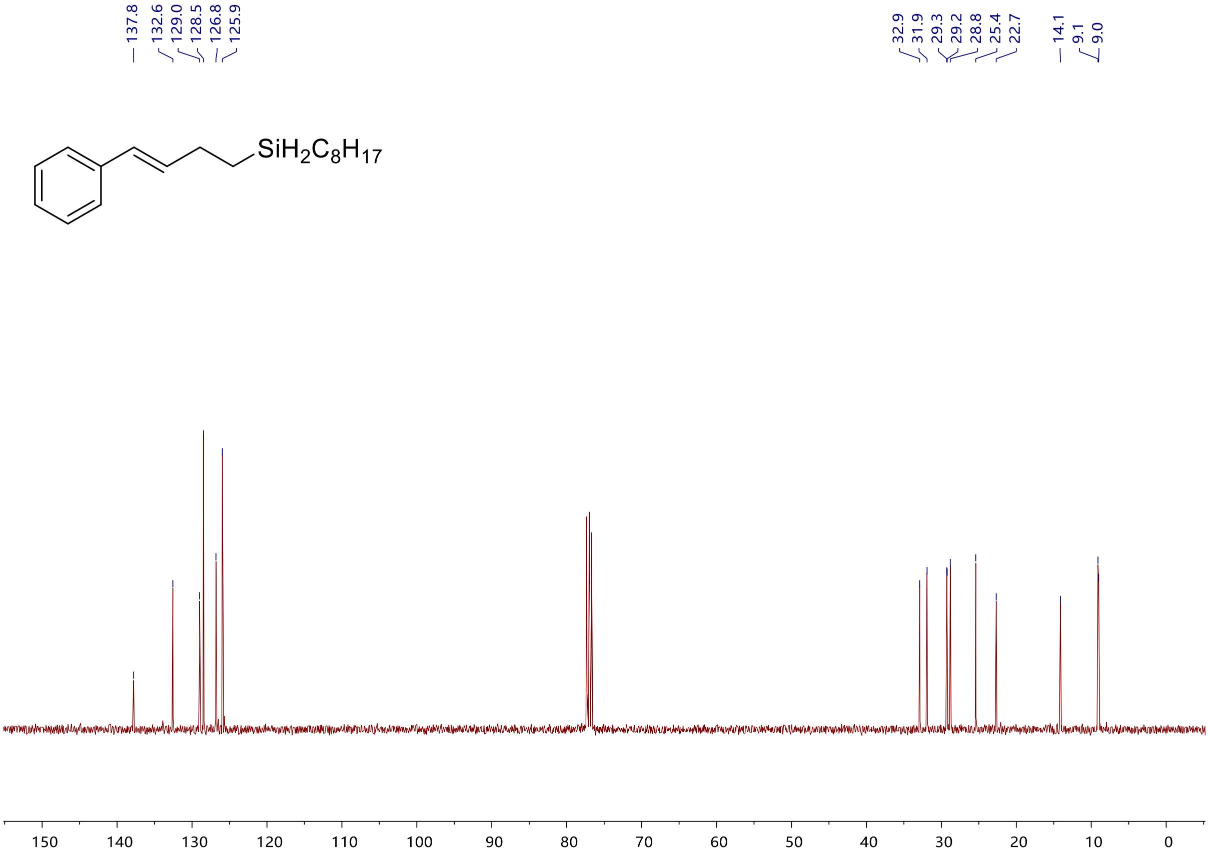

(*E*)-phenyl(4-(o-tolyl)but-3-en-1-yl)silane (3ba)

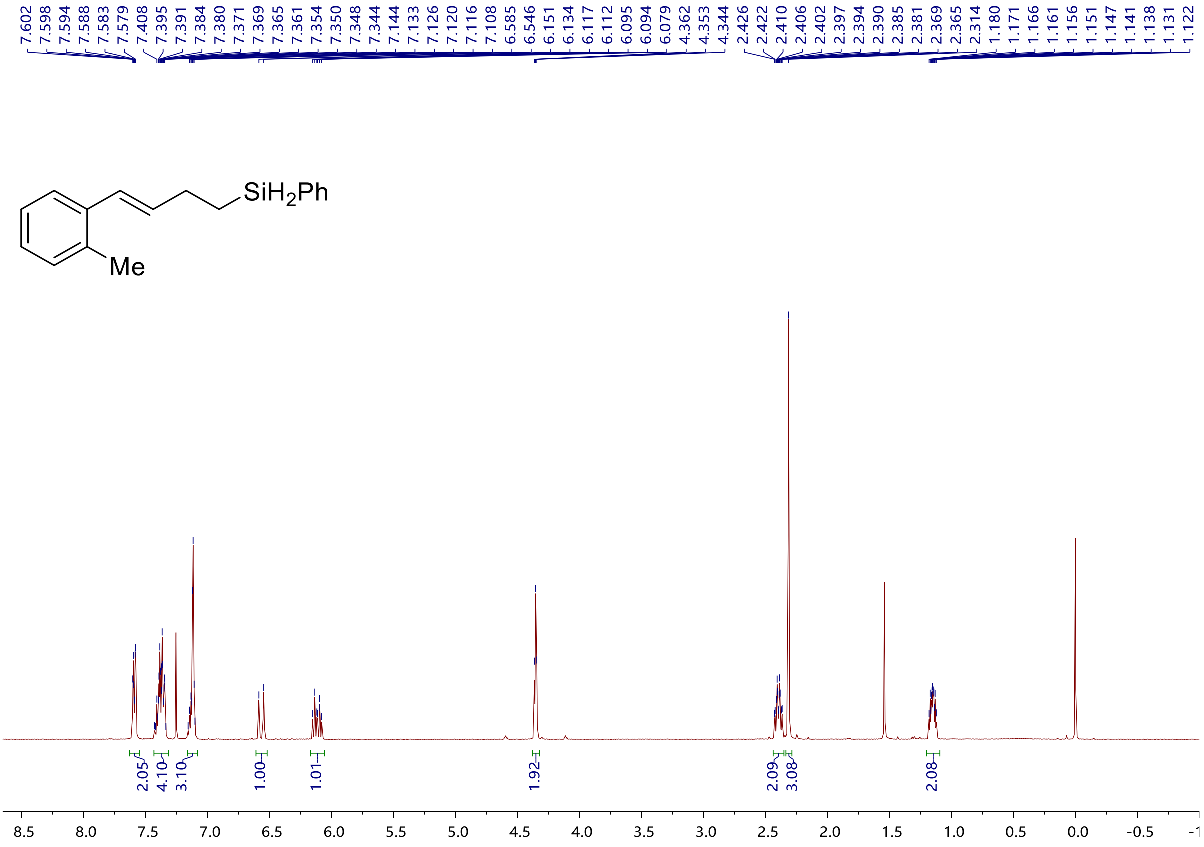

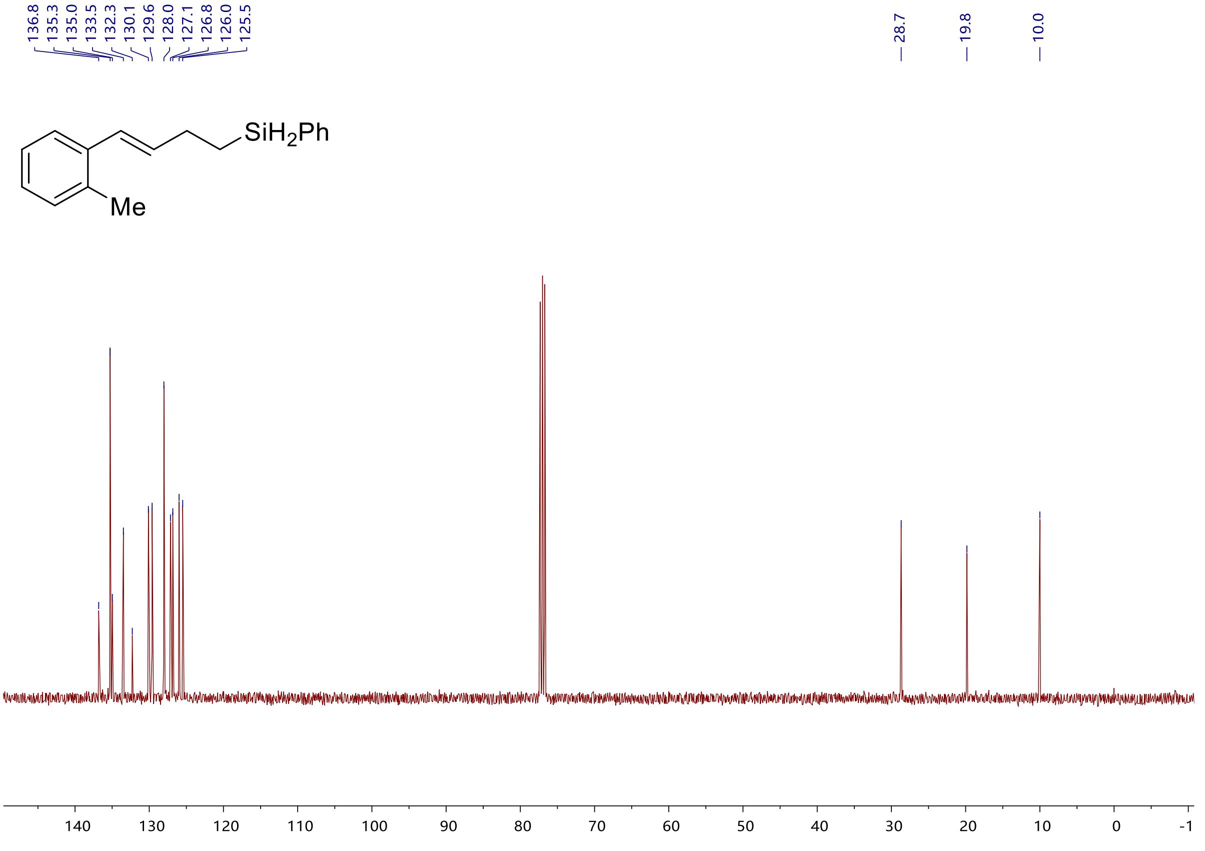

(*E*)-phenyl(4-(3-(trifluoromethyl)phenyl)but-3-en-1-yl)silane (3ca)

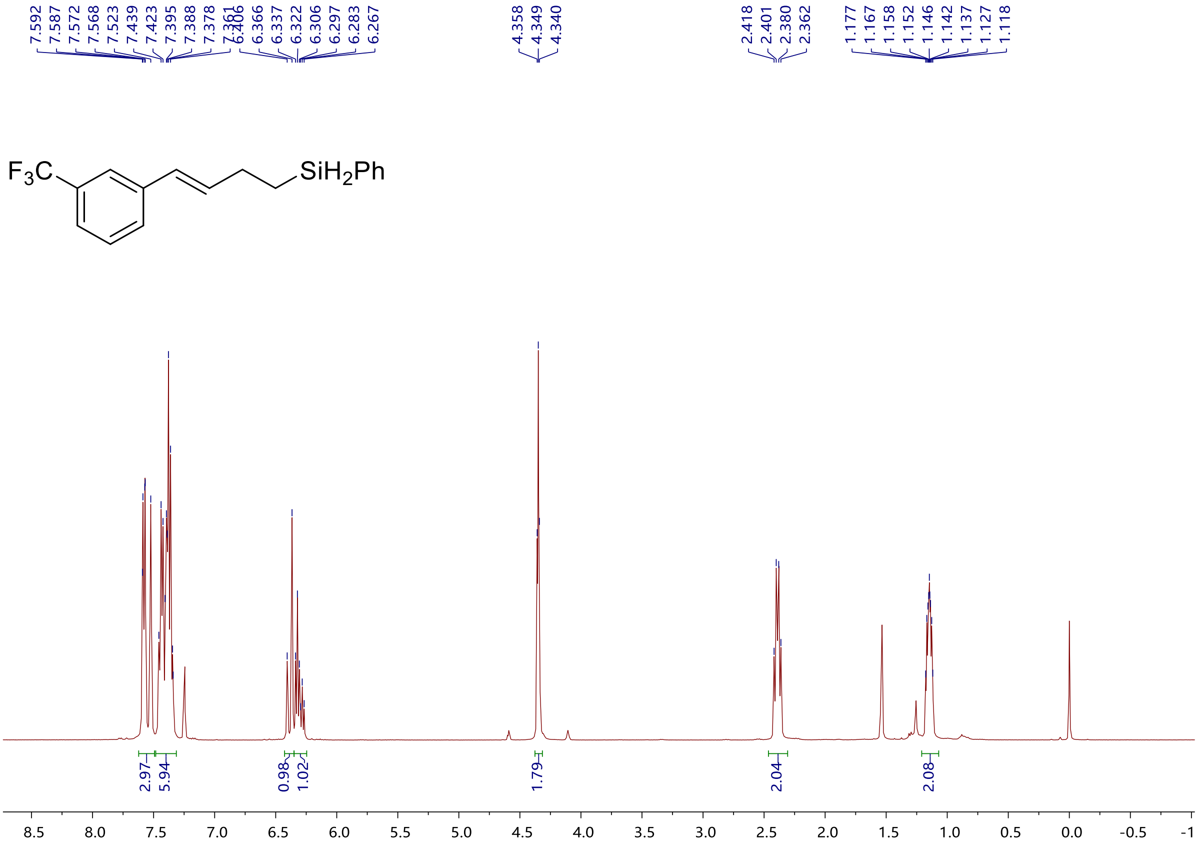

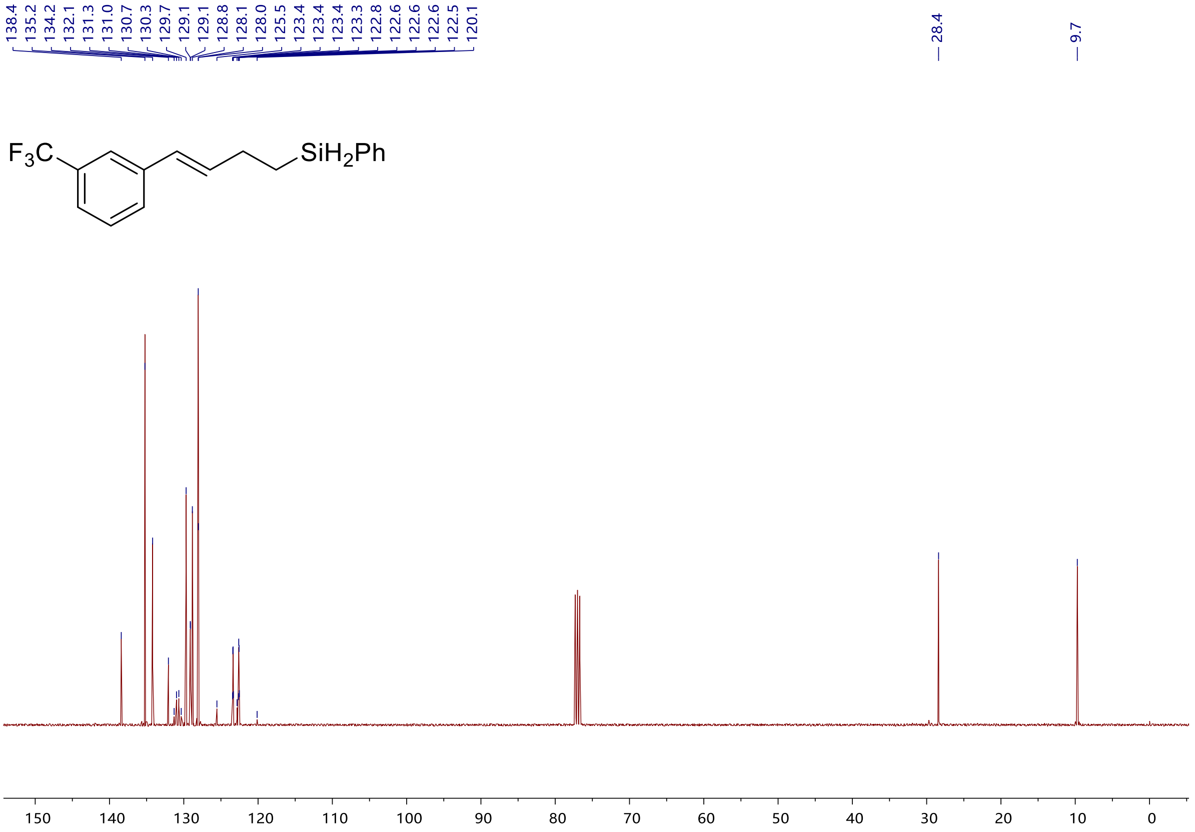

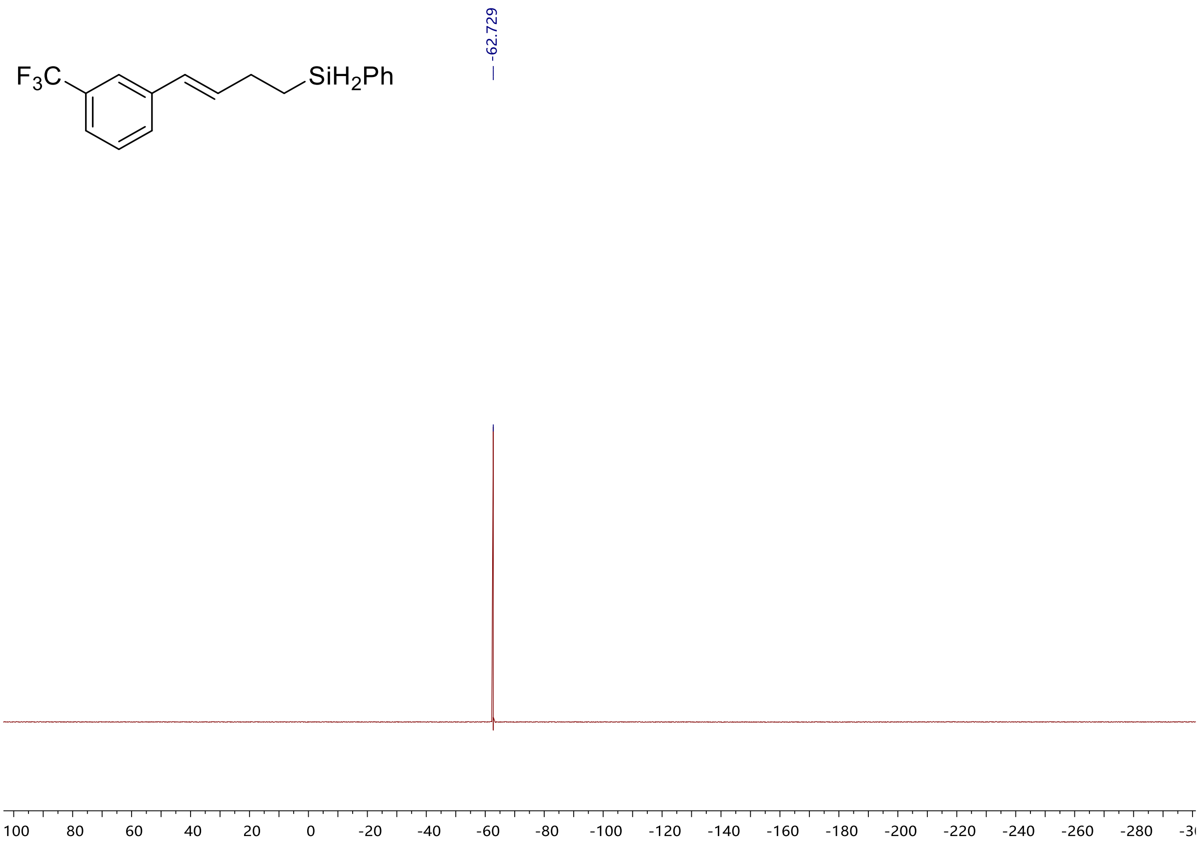

F_3C
SiH_2Ph
-62.729
100
80
60
40
20
0
-20
-40
-60
-80
-100
-120
-140
-160
-180
-200
-220
-240
-260
-280

(*E*)-phenyl(4-(p-tolyl)but-3-en-1-yl)silane (3da)

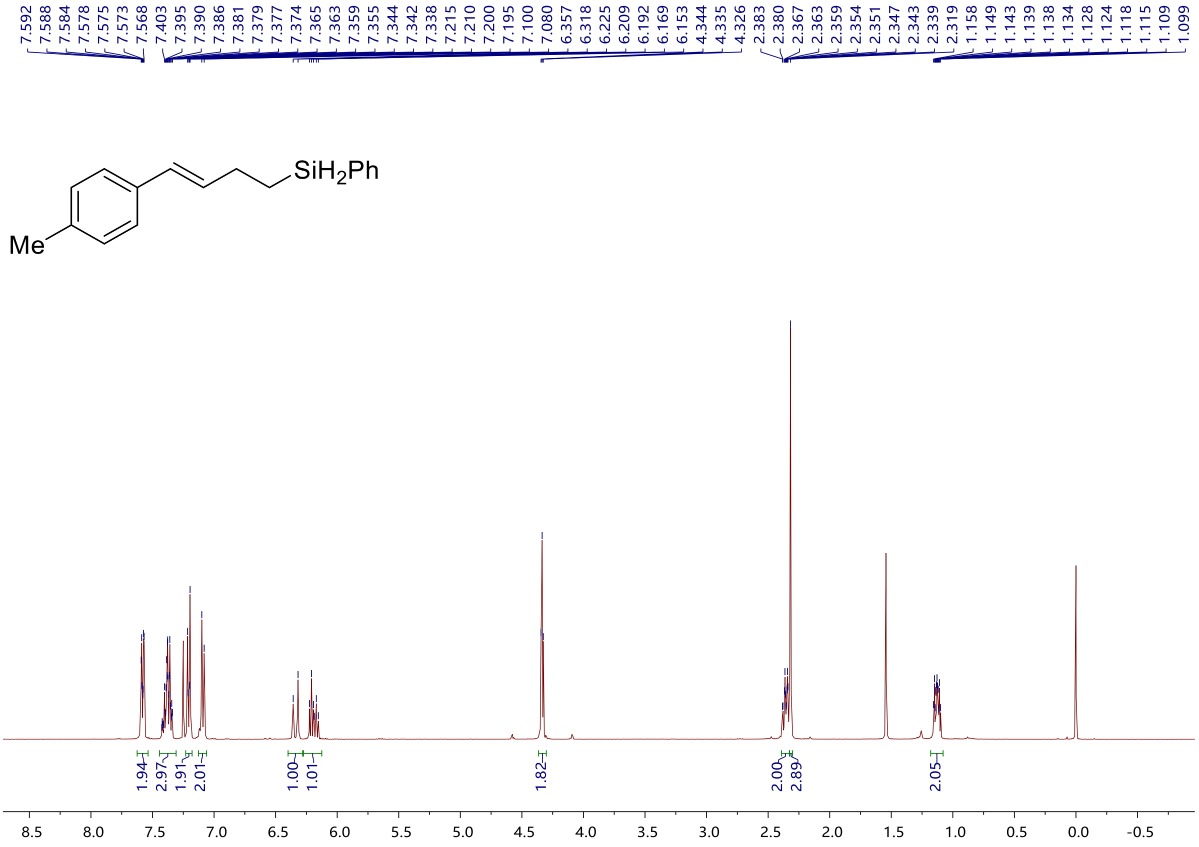

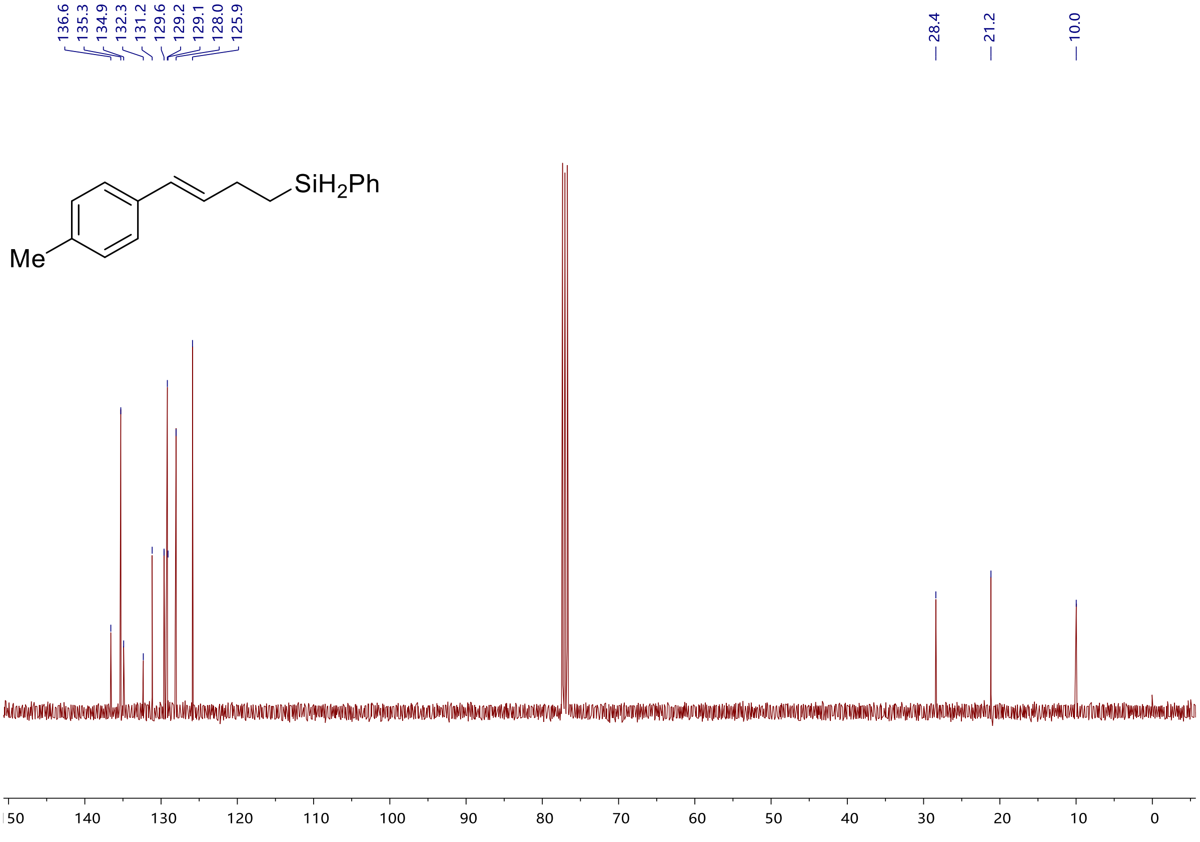

(*E*)-(4-(4-methoxyphenyl)but-3-en-1-yl)(phenyl)silane (3ea)

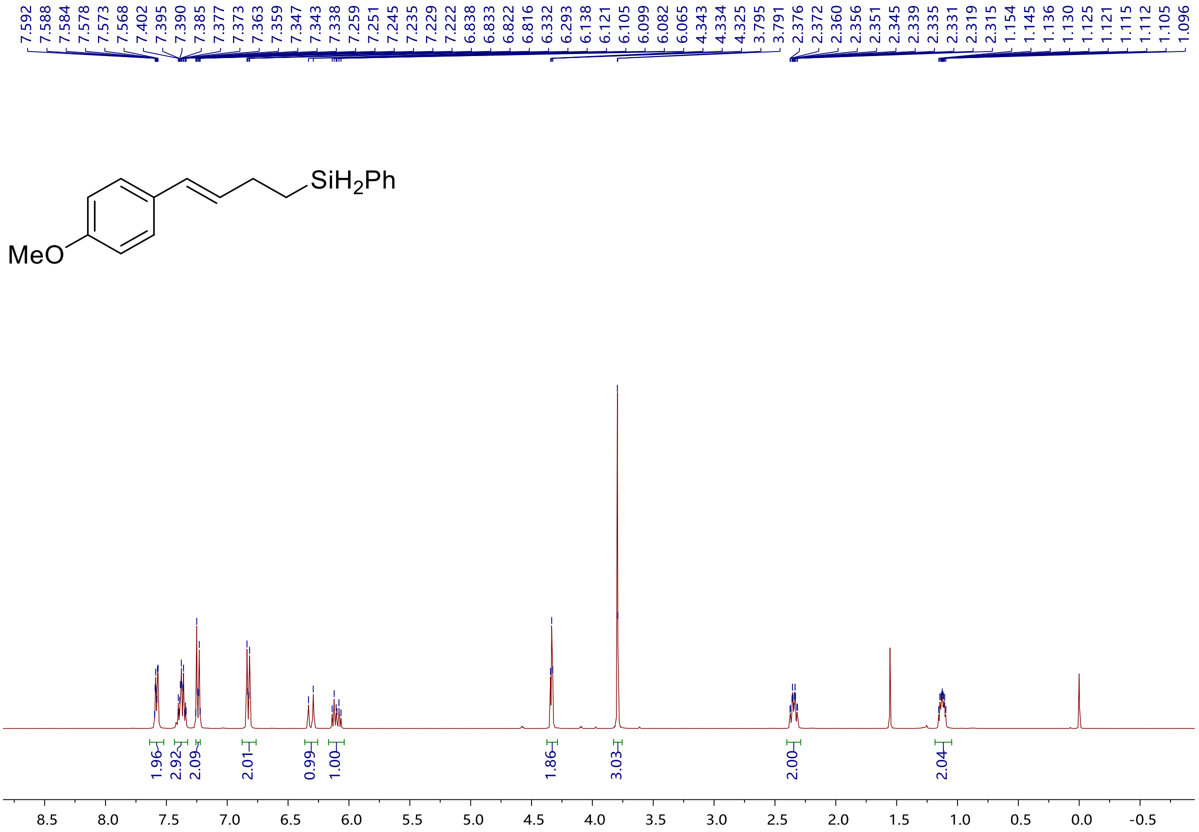

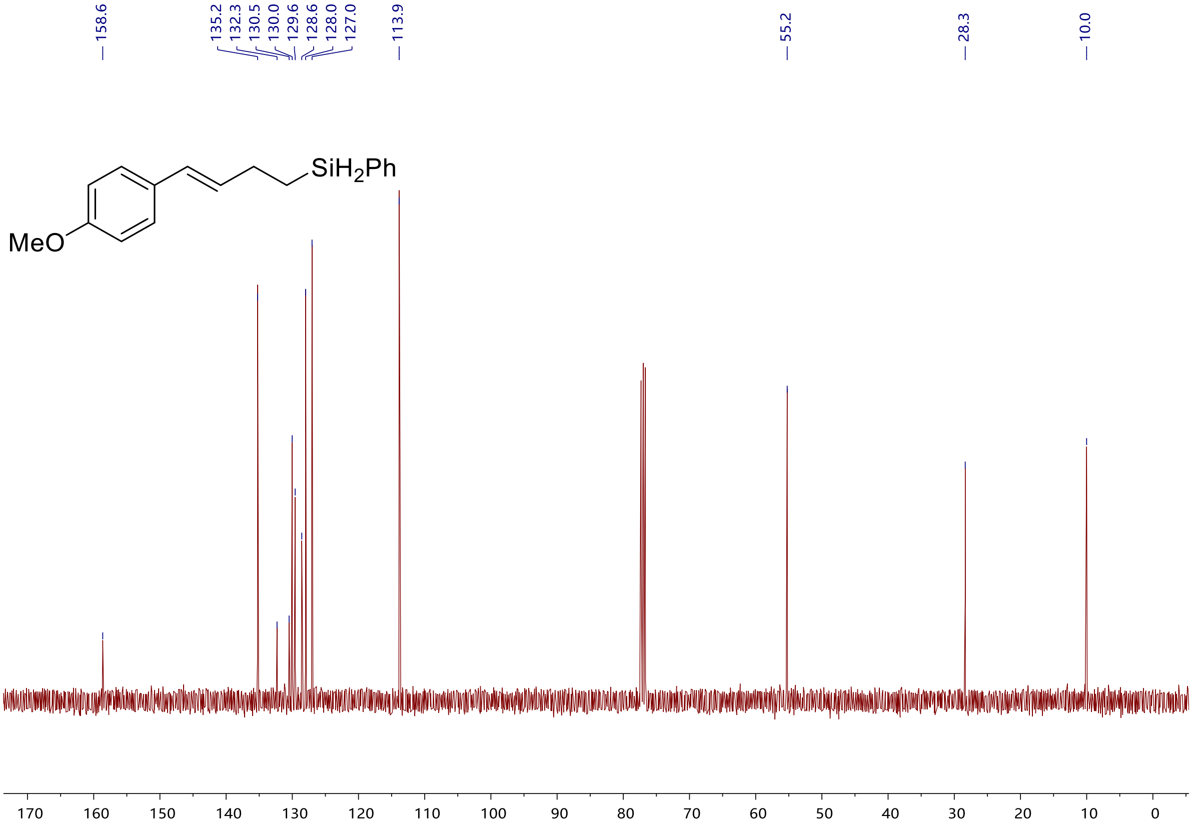

(*E*)-N,N-dimethyl-4-(4-(phenylsilyl)but-1-en-1-yl)aniline (3fa)

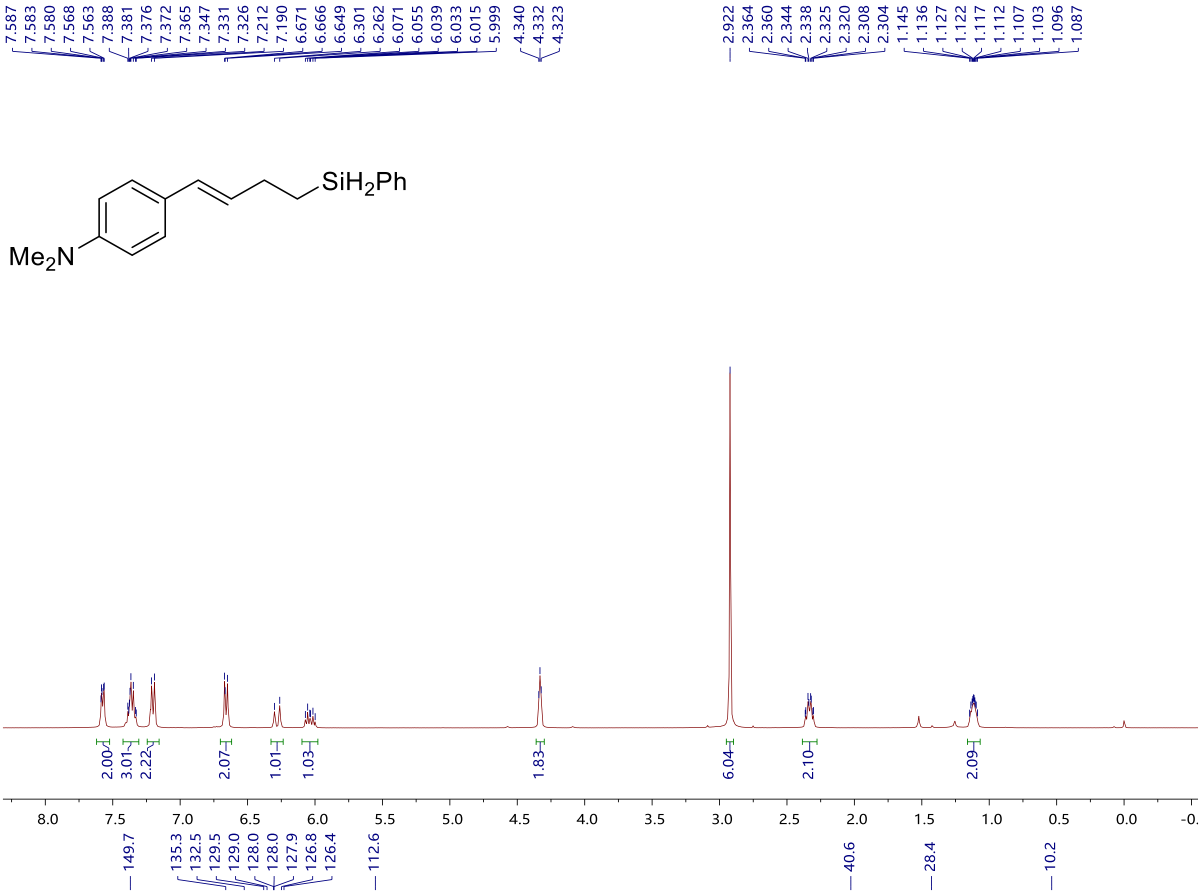

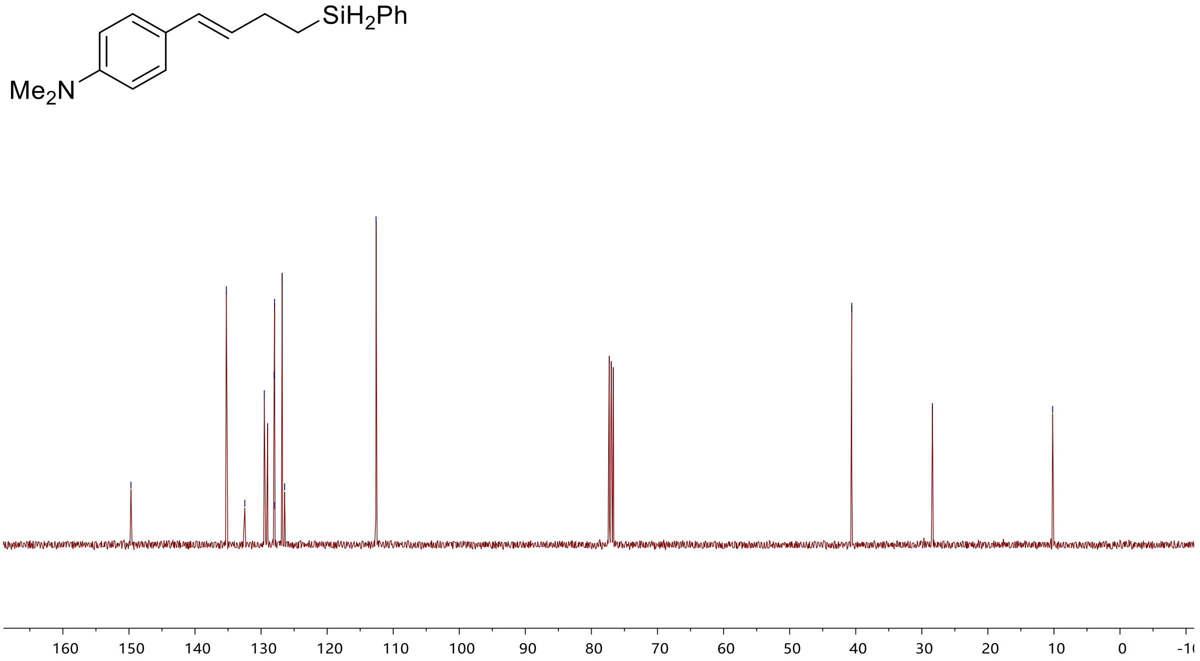

(*E*)-(4-(4-fluorophenyl)but-3-en-1-yl)(phenyl)silane (3ga)

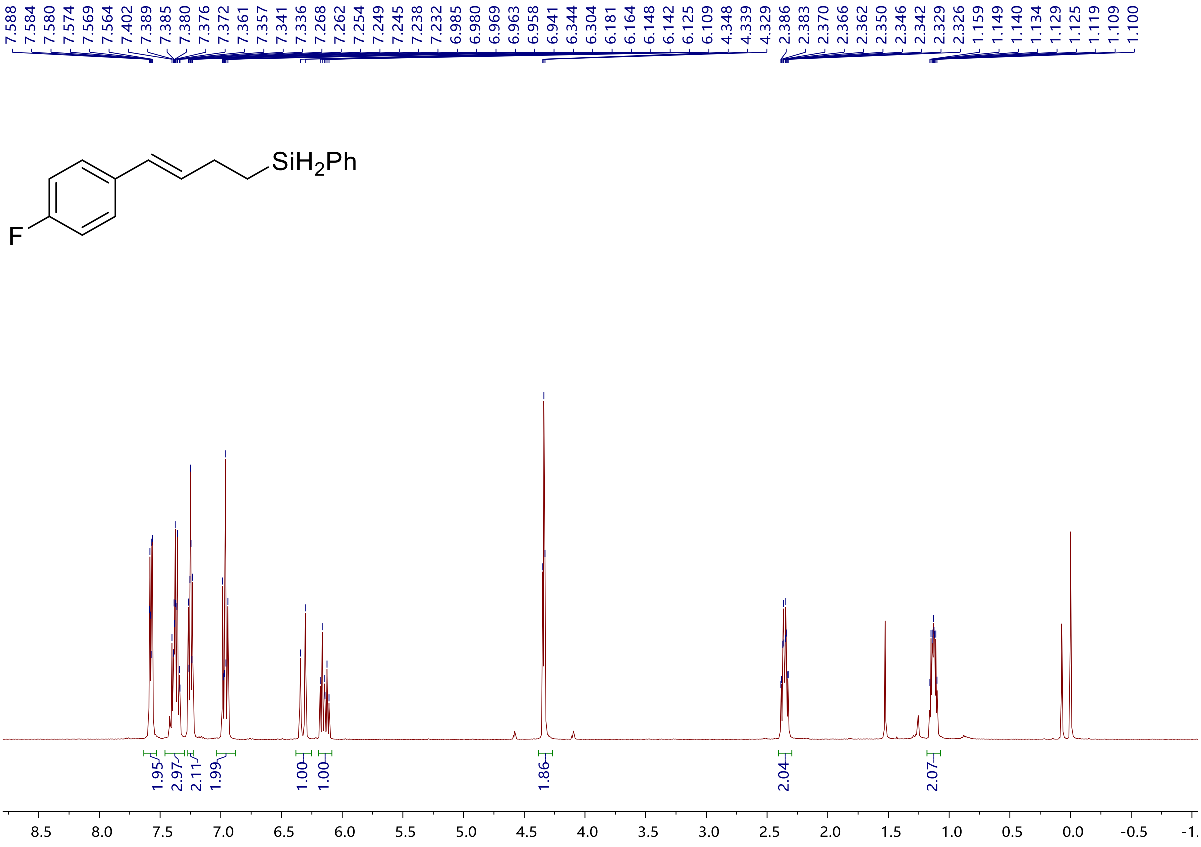

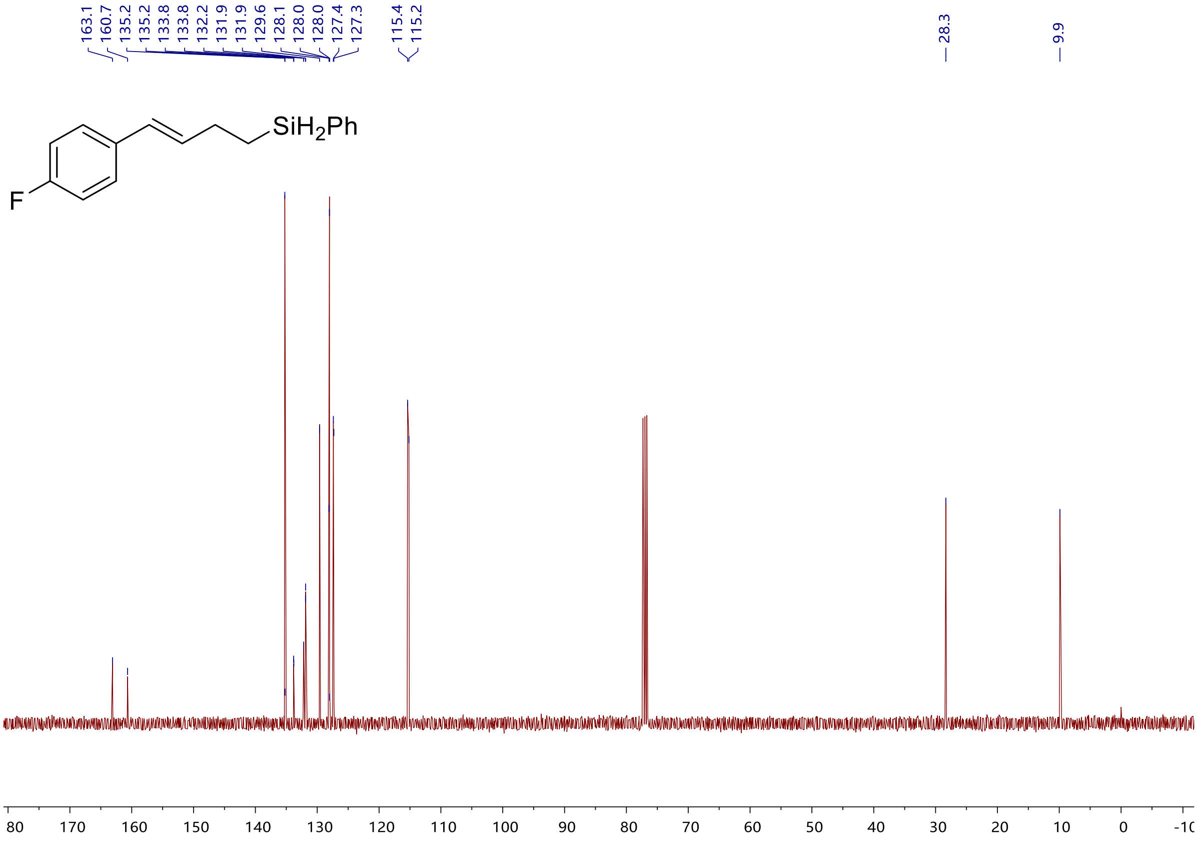

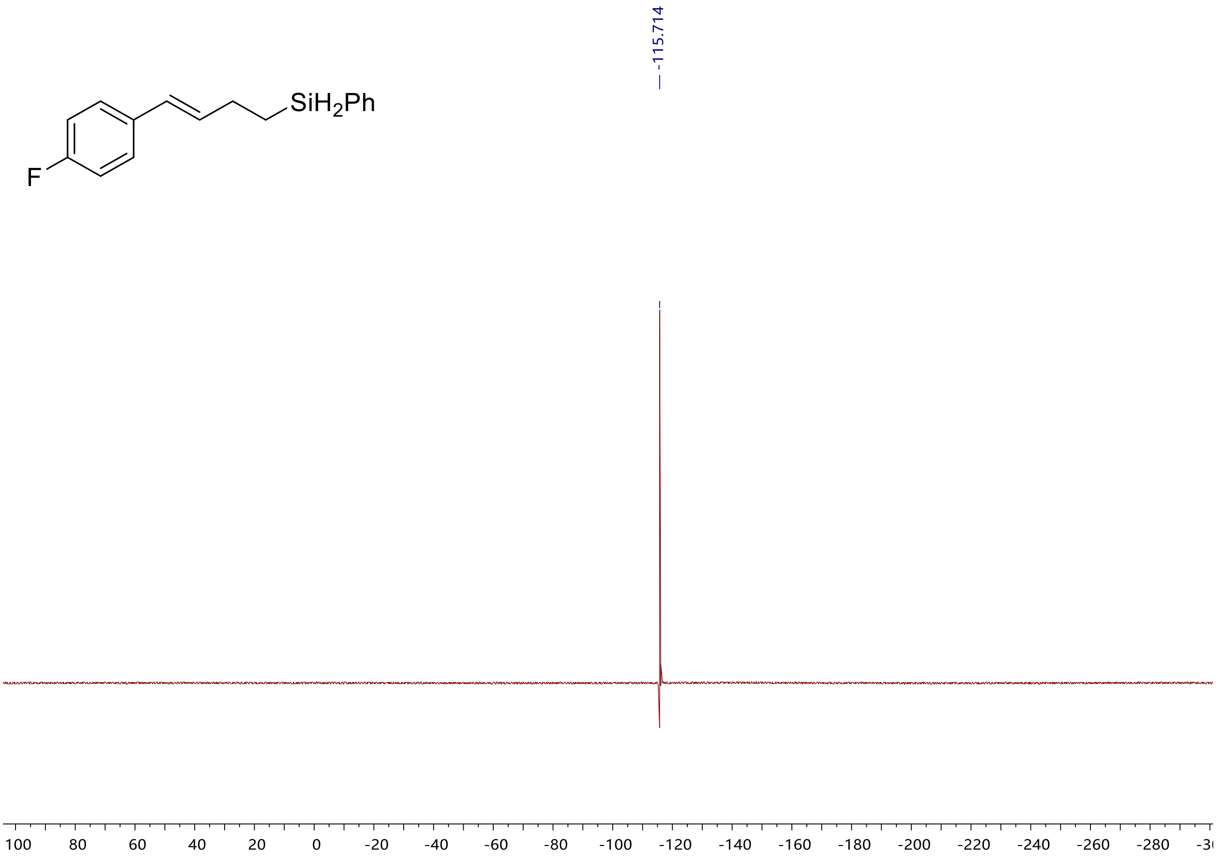

-115.714
SiH2Ph
F
100
80
60
40
20
0
-20
-40
-60
-80
-100
-120
-140
-160
-180
-200
-220
-240
-260
-280

(*E*)-(4-(4-chlorophenyl)but-3-en-1-yl)(phenyl)silane (3ha)

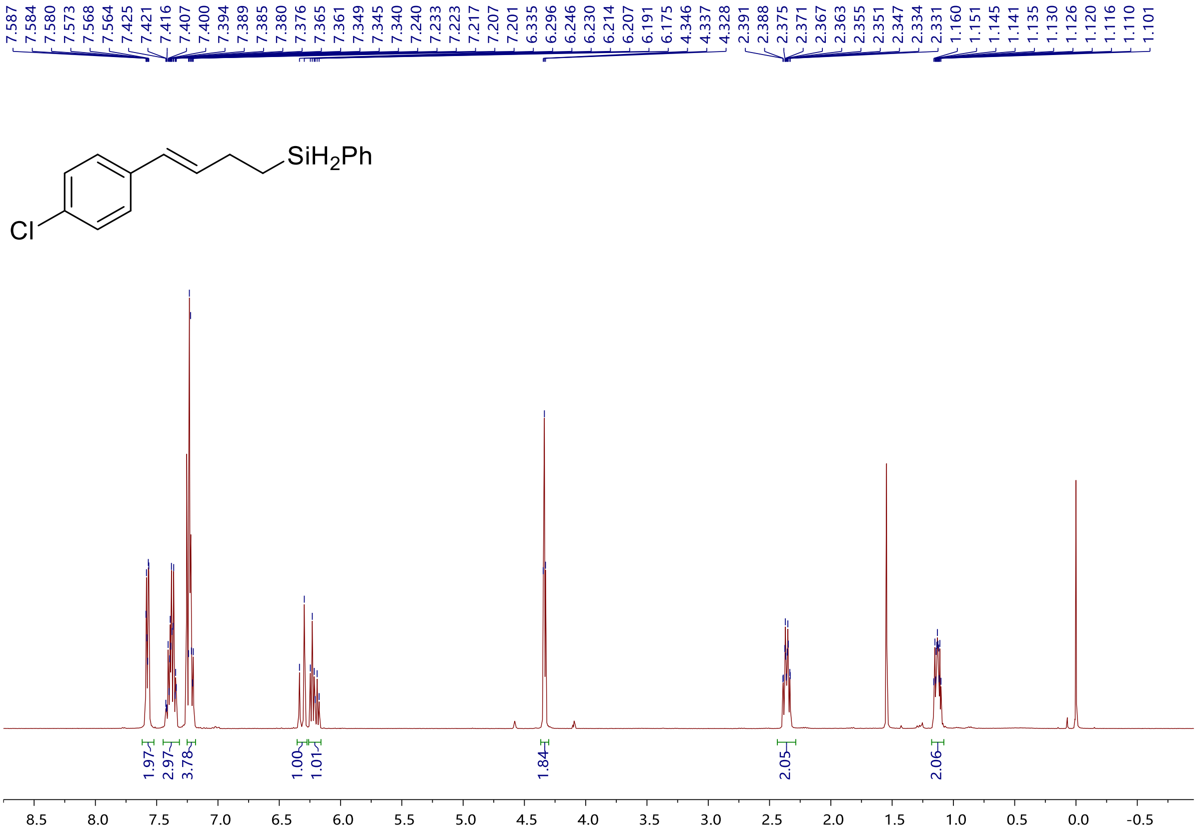

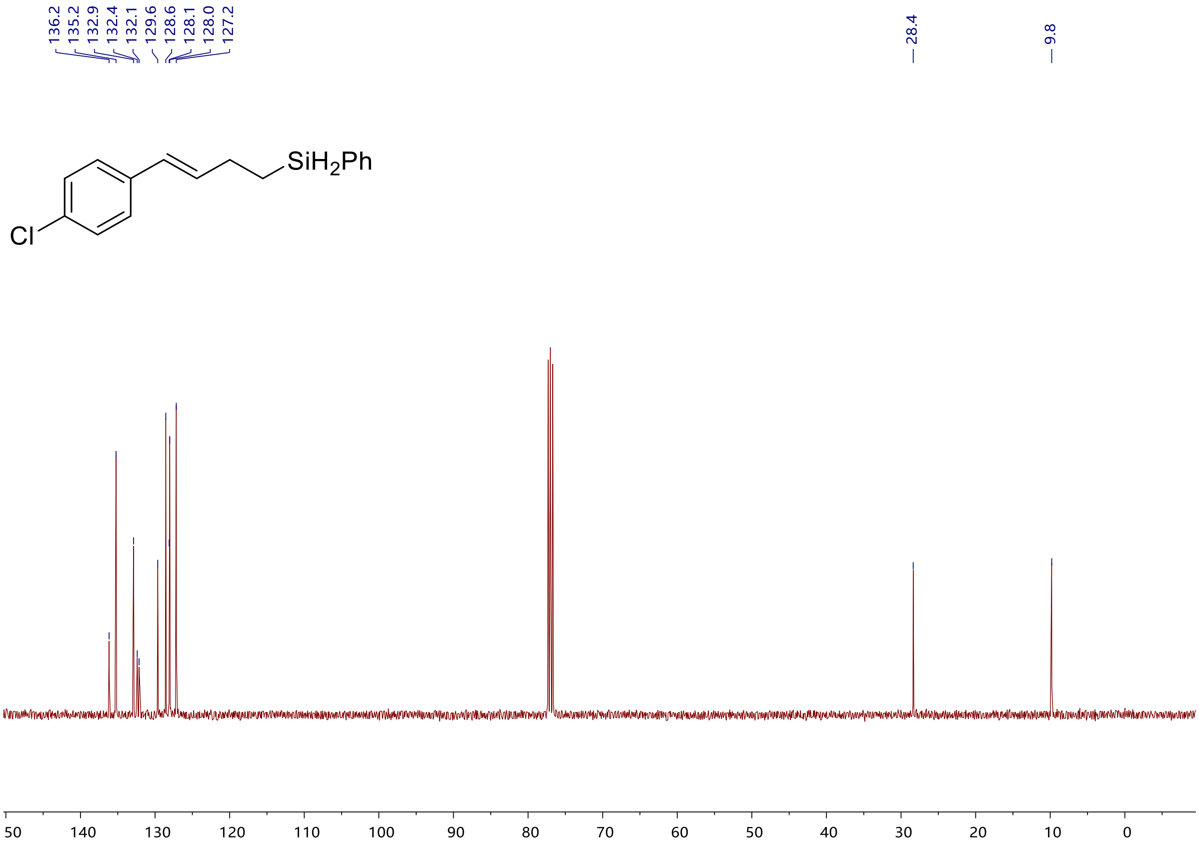

(*E*)-(4-(naphthalen-2-yl)but-3-en-1-yl)(phenyl)silane (3ia)

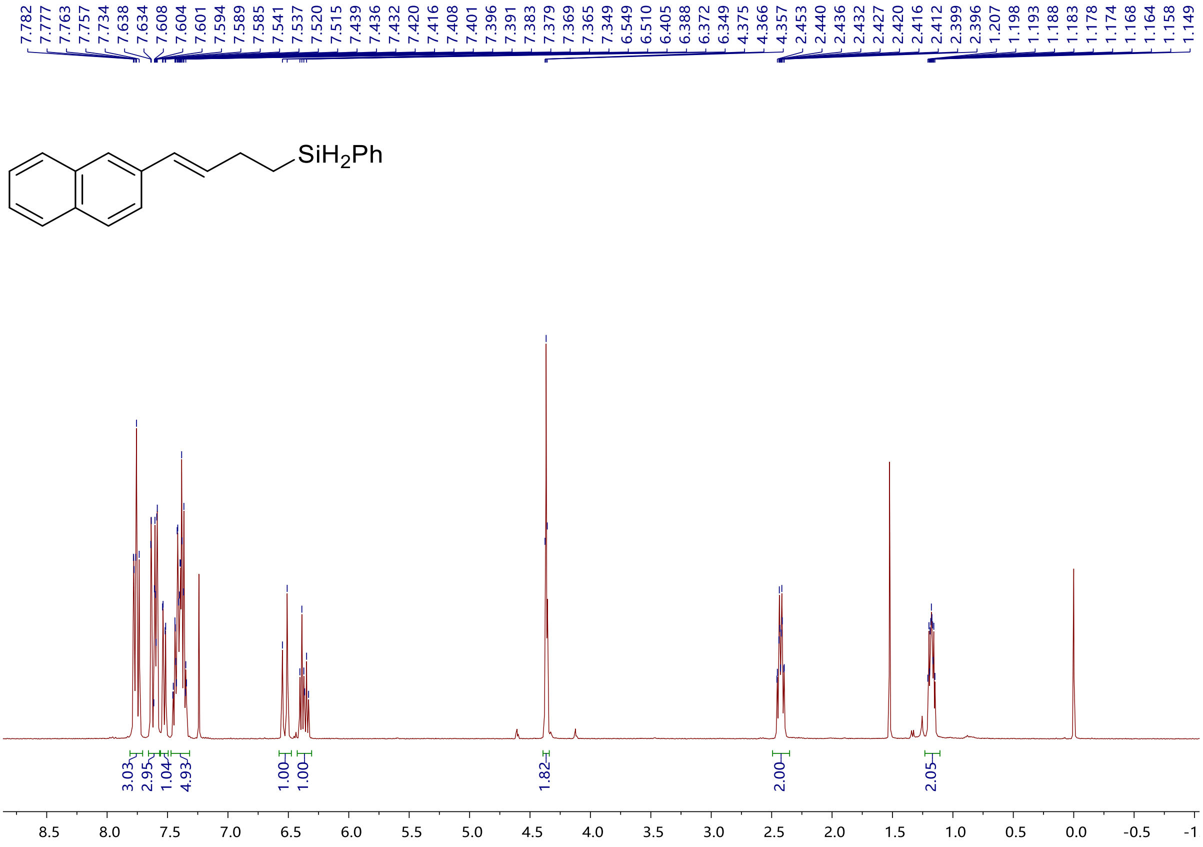

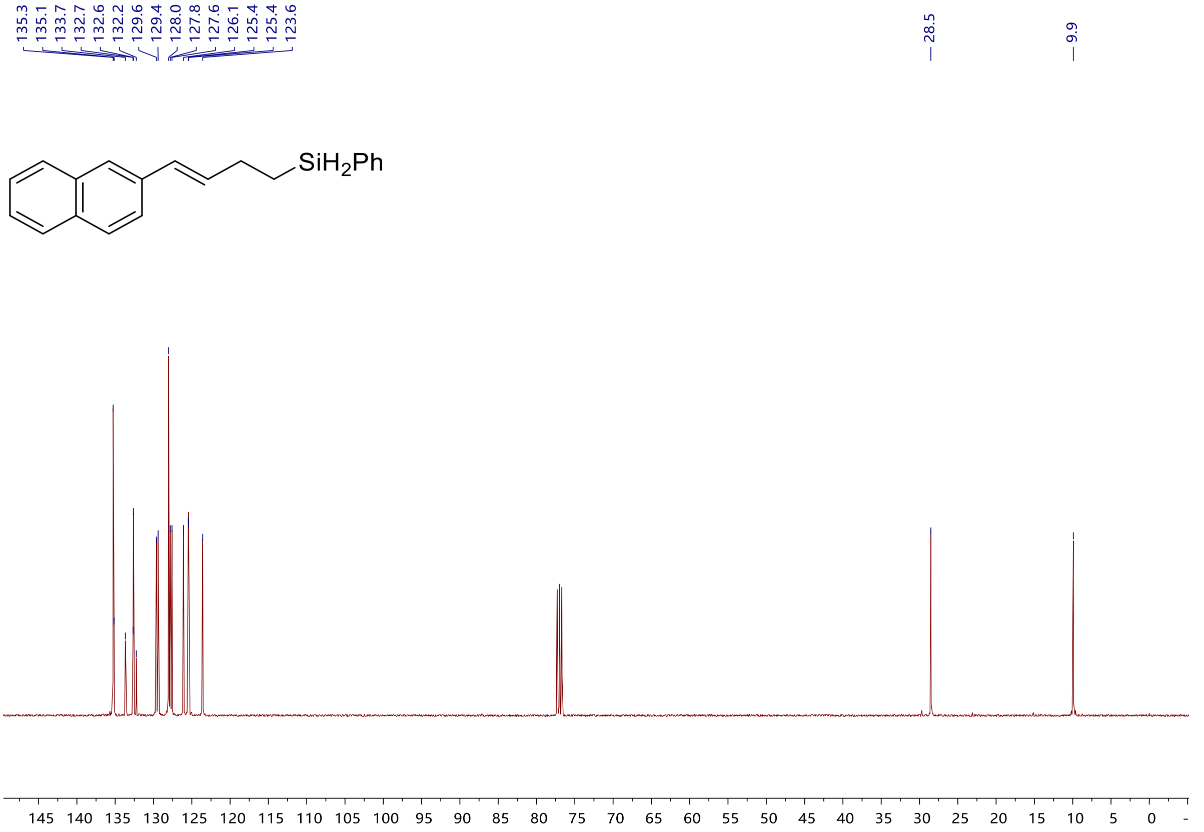

(*E*)-(4-(benzo[d][1,3]dioxol-5-yl)but-3-en-1-yl)(phenyl)silane (3ja)

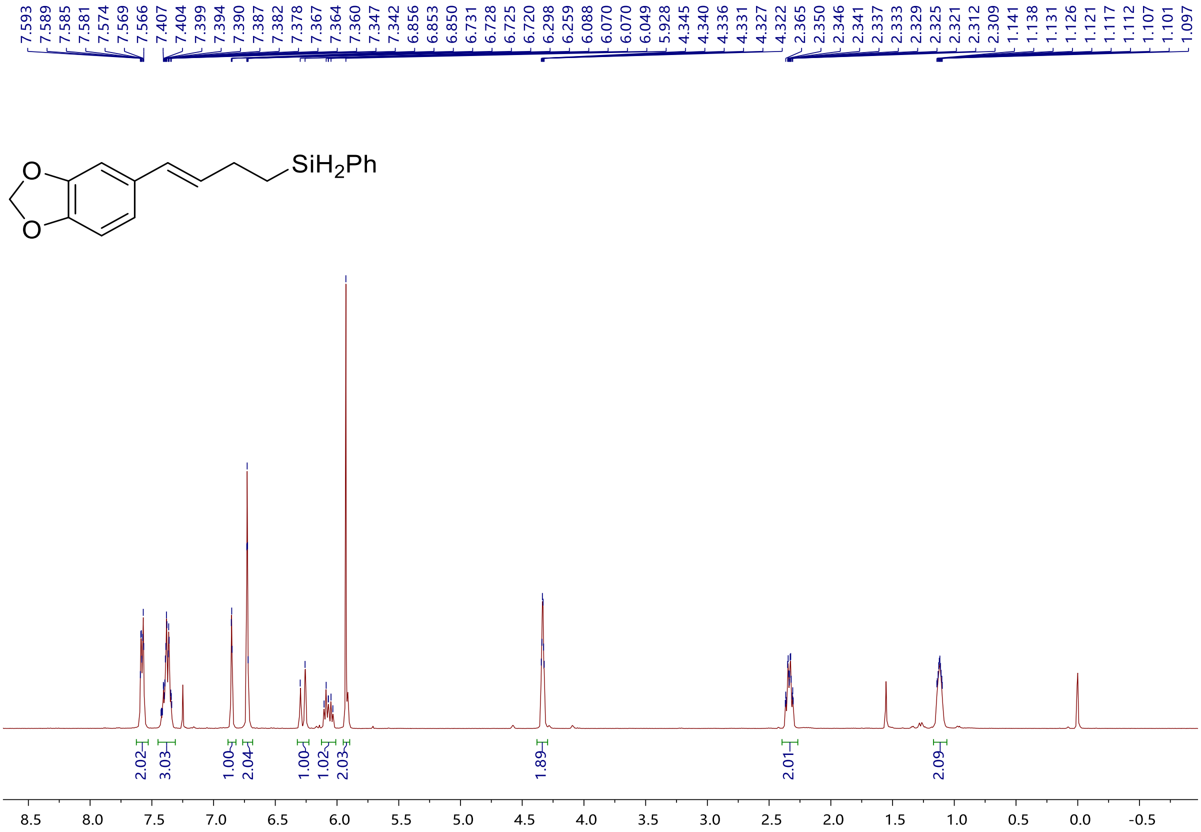

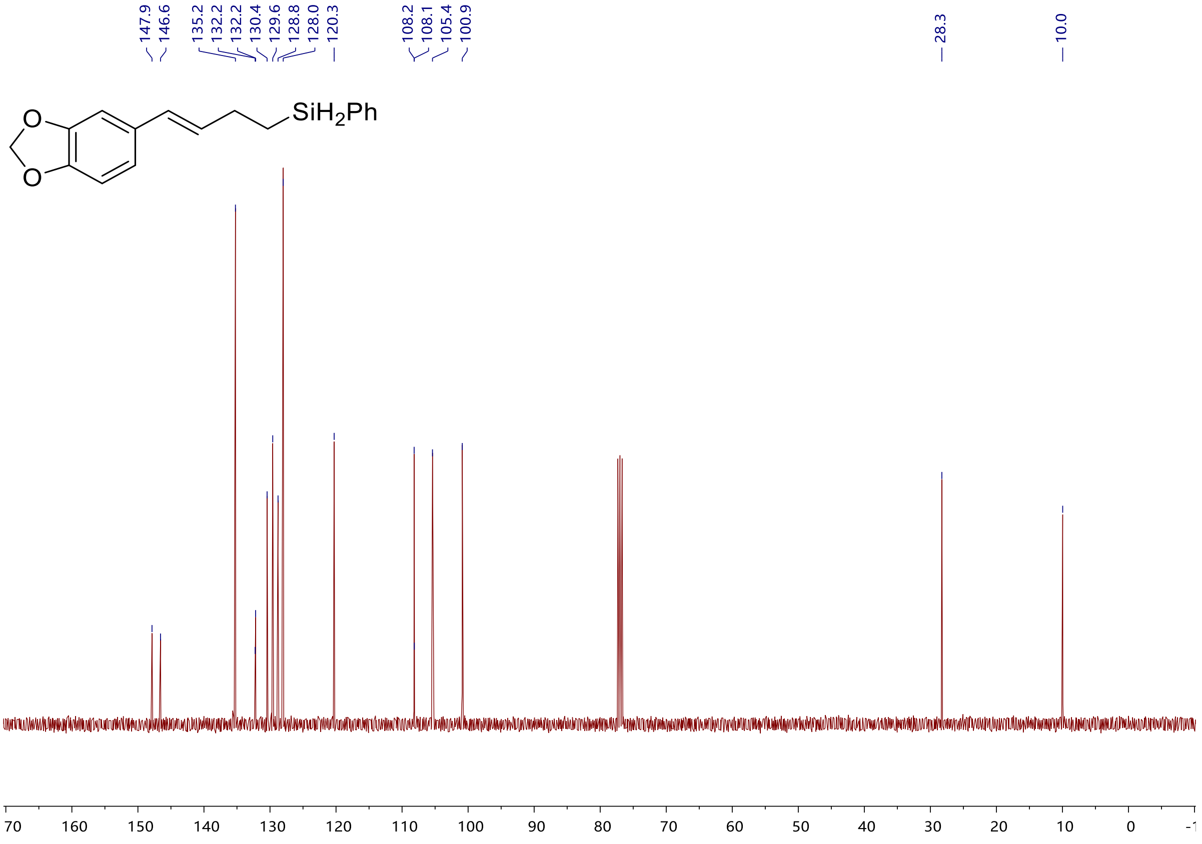

(*E*)-(4-(furan-2-yl)but-3-en-1-yl)(phenyl)silane (3ka)

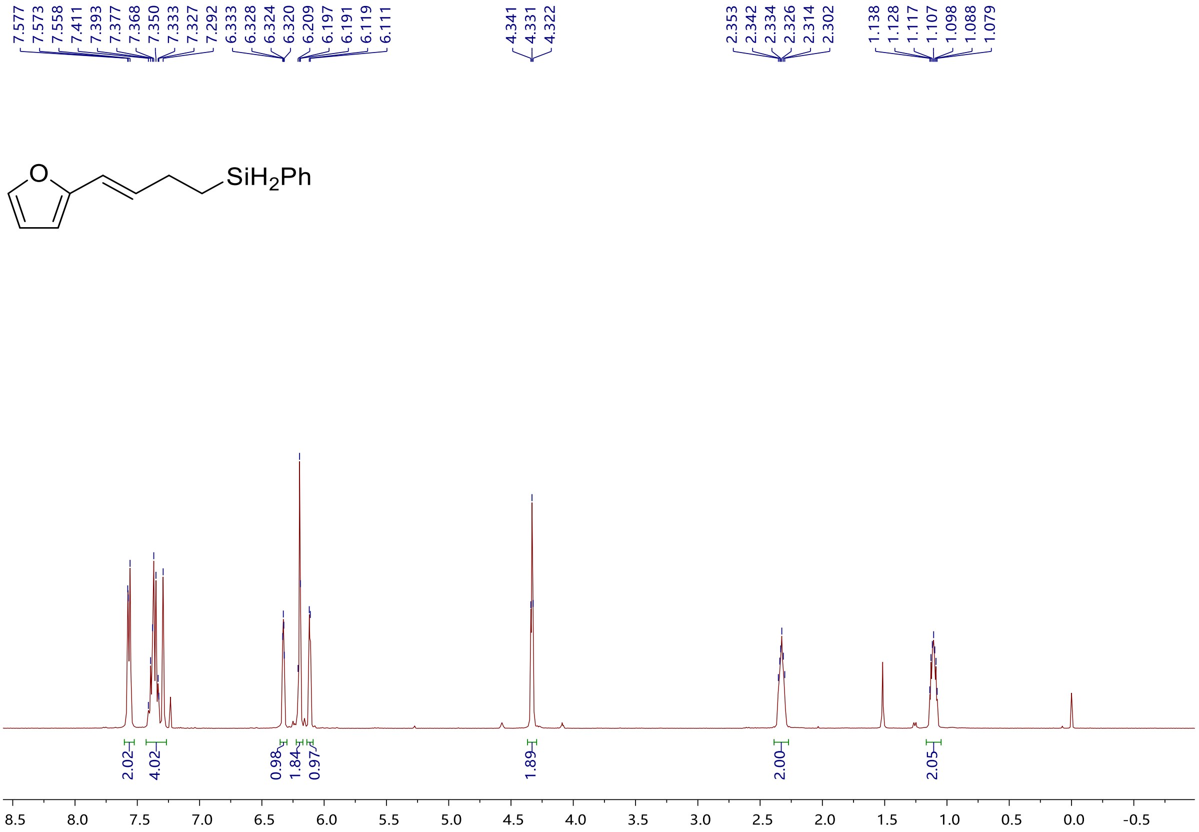

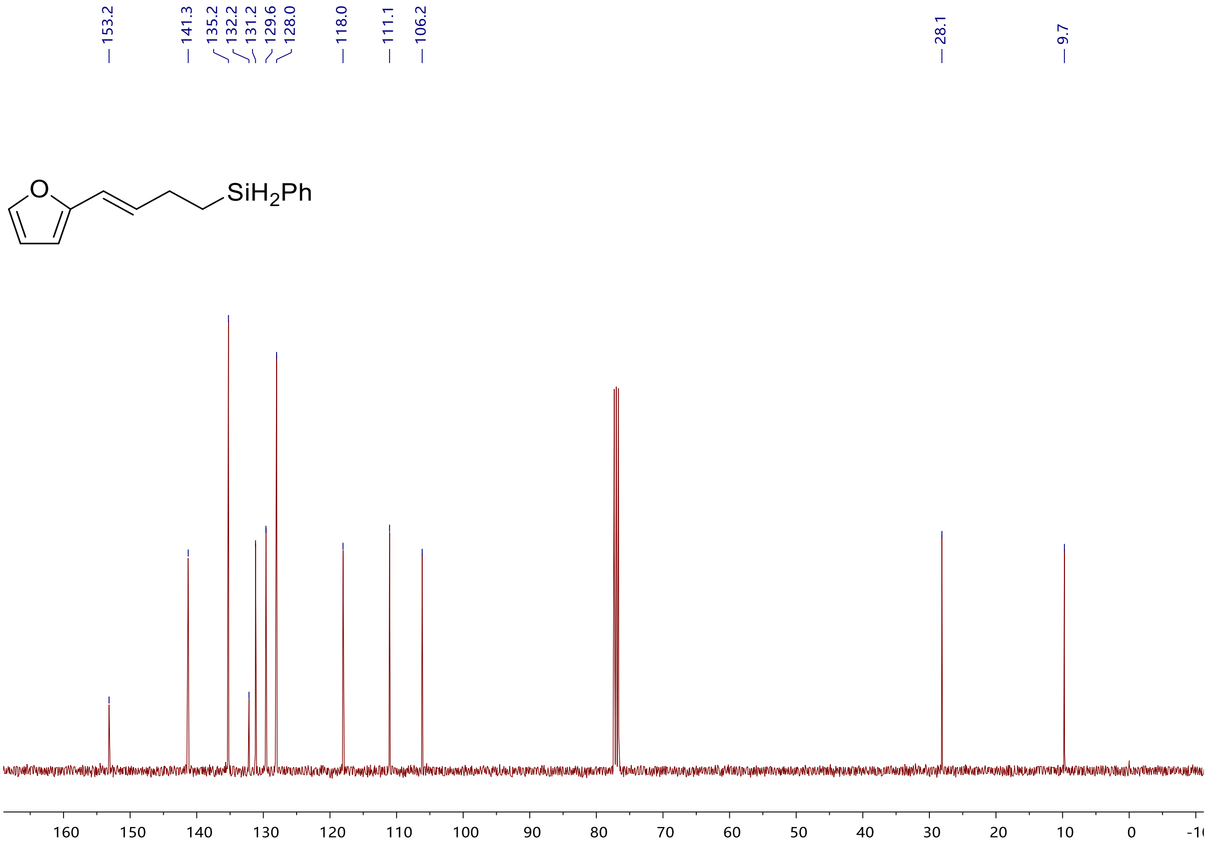

(*E*)-phenyl(4-phenylpent-3-en-1-yl)silane (3la)

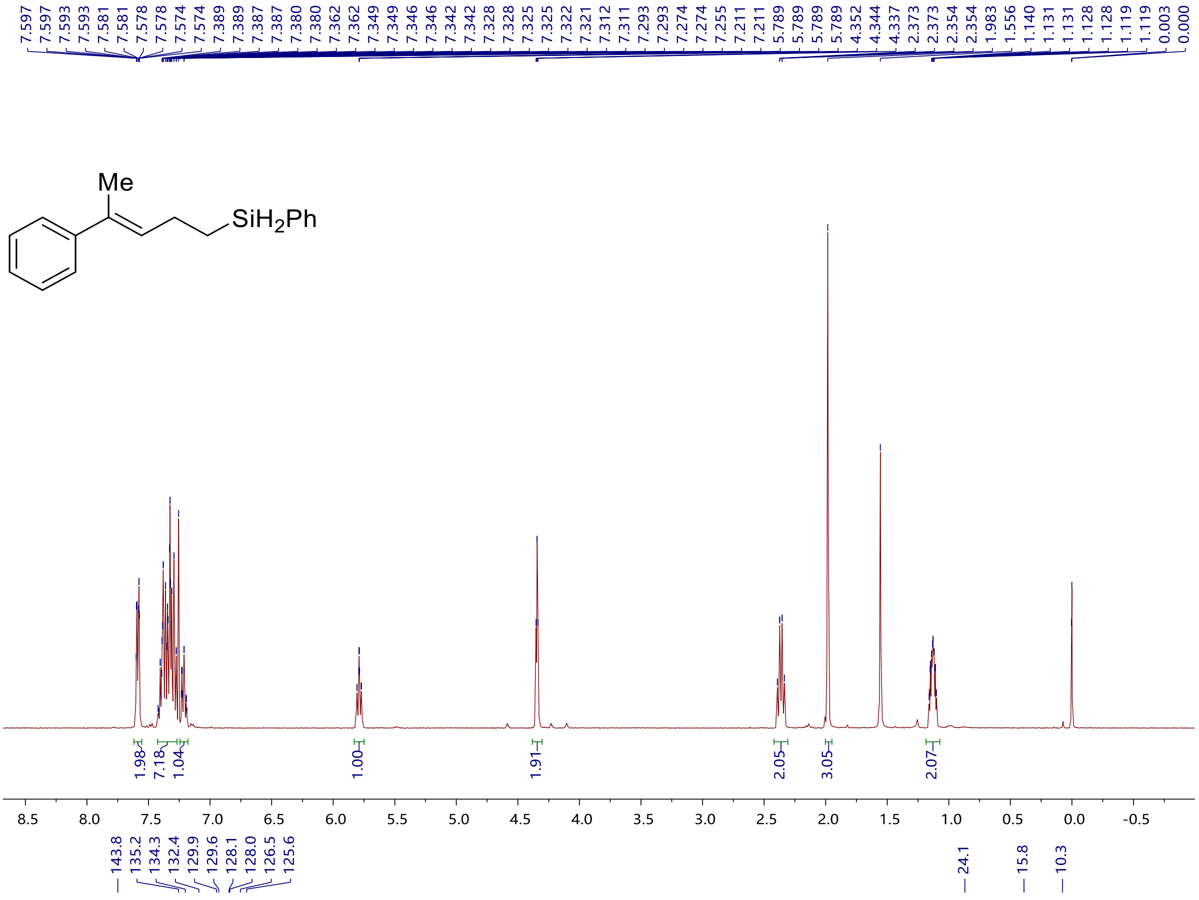

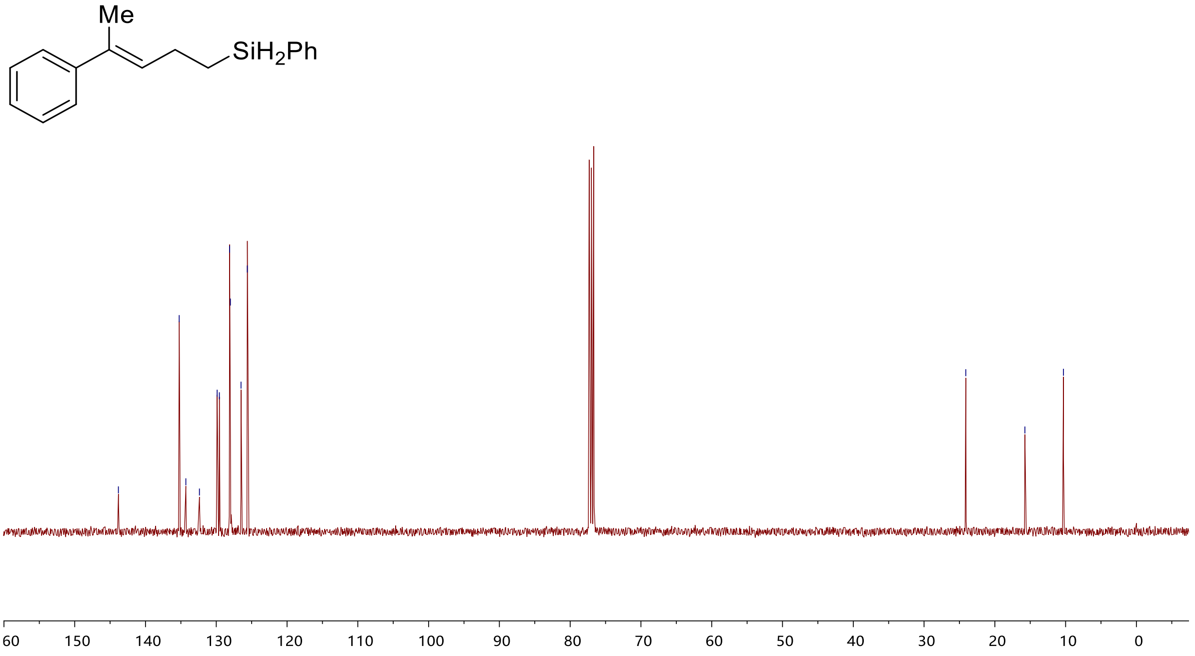

(4,4-diphenylbut-3-en-1-yl)(phenyl)silane (3ma)

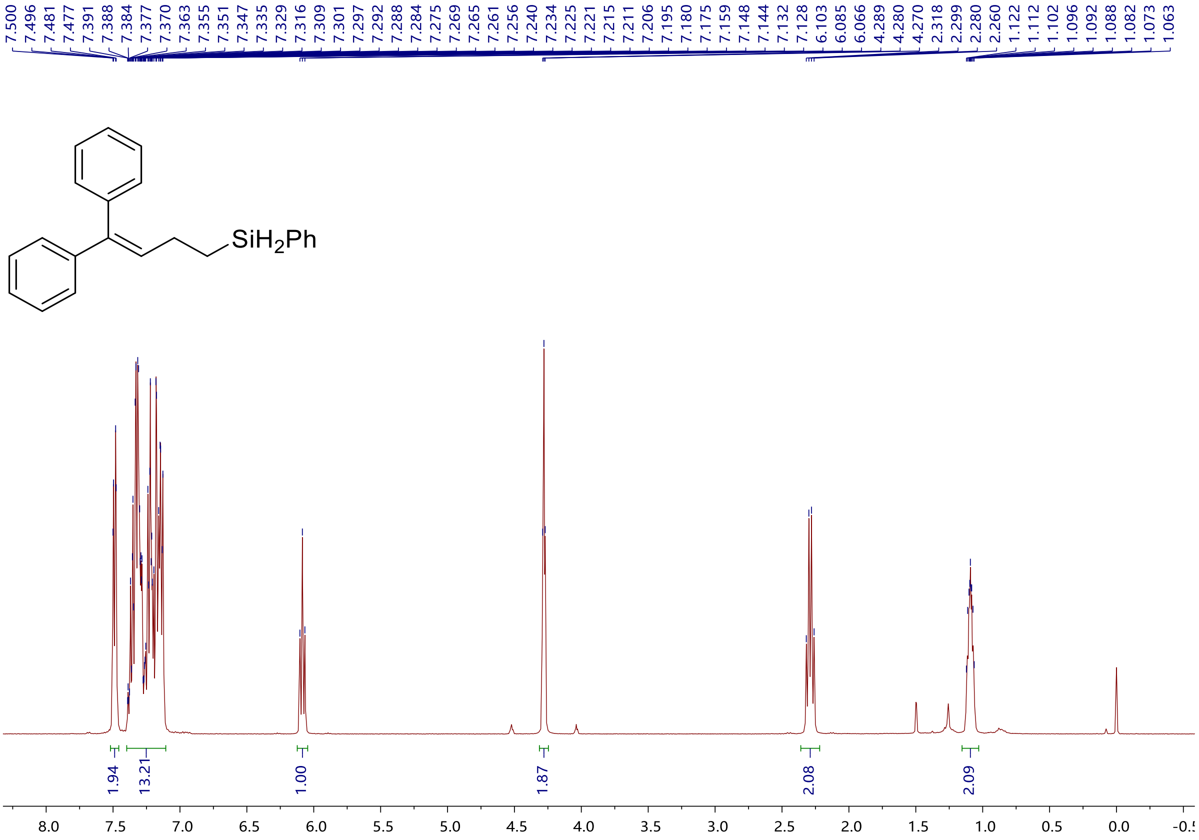

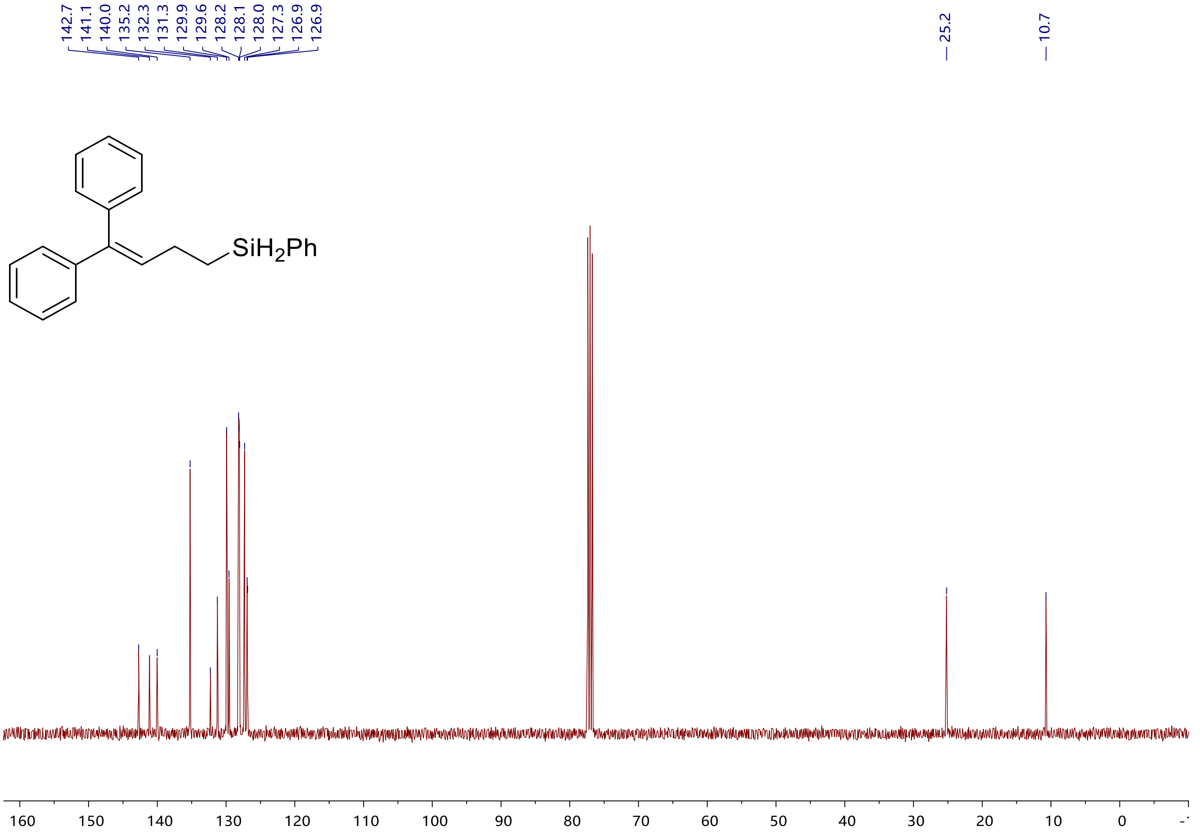

(*E*)-(3-methyl-4-phenylbut-3-en-1-yl)(phenyl)silane (3na)

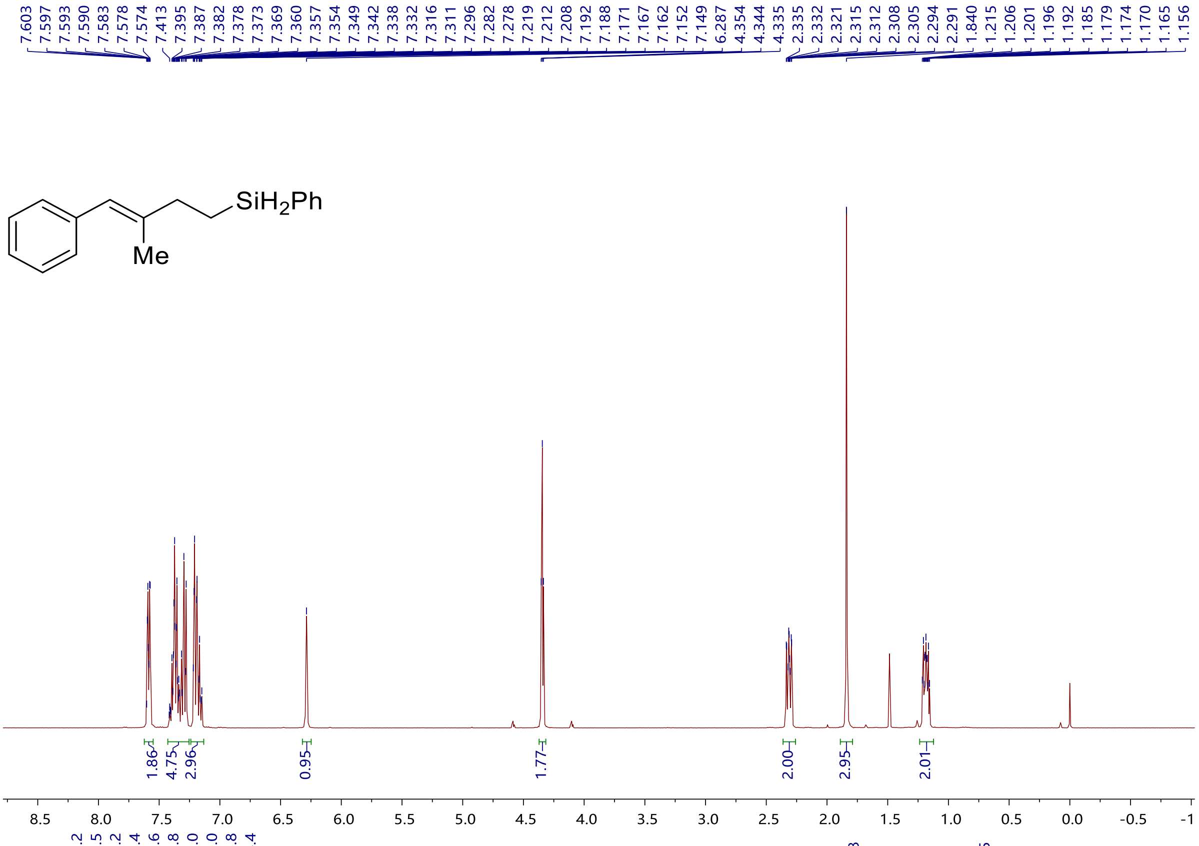

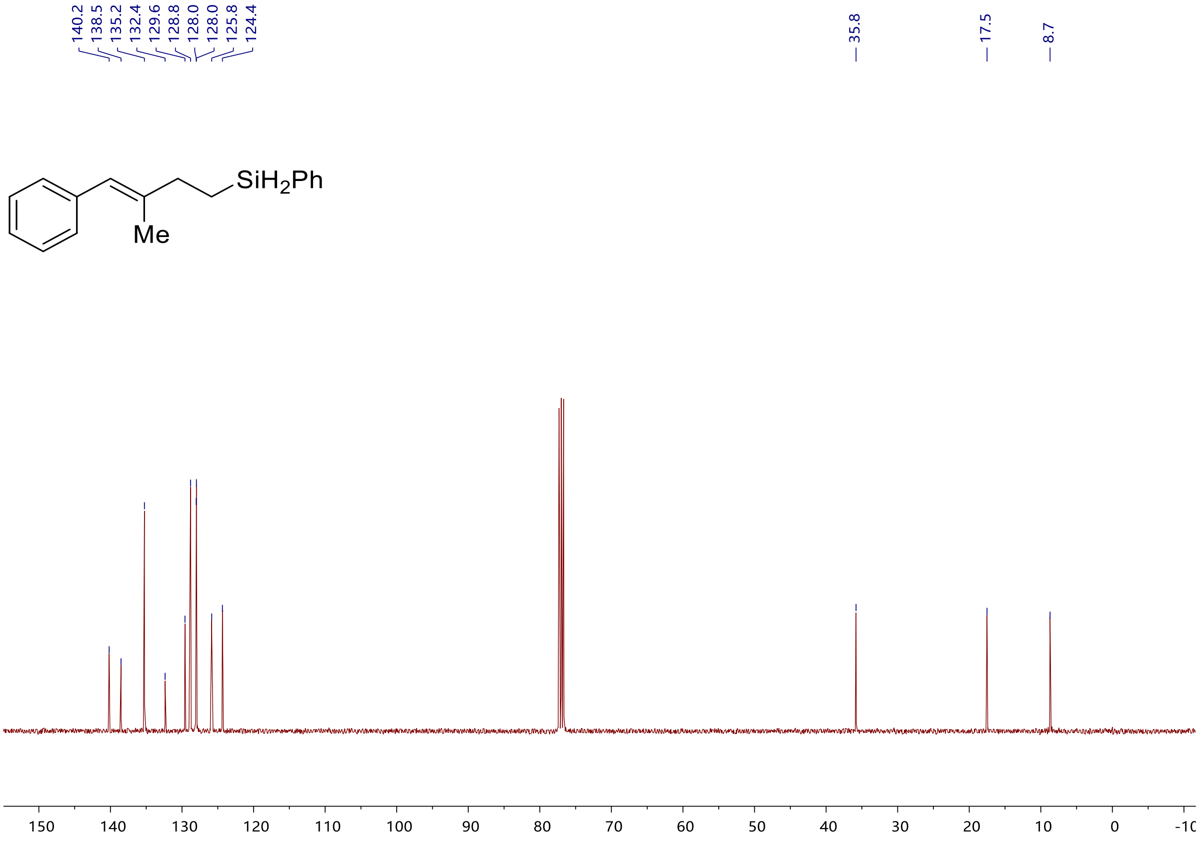

(*Z*)-phenyl(3-phenylpent-3-en-1-yl)silane (3oa)

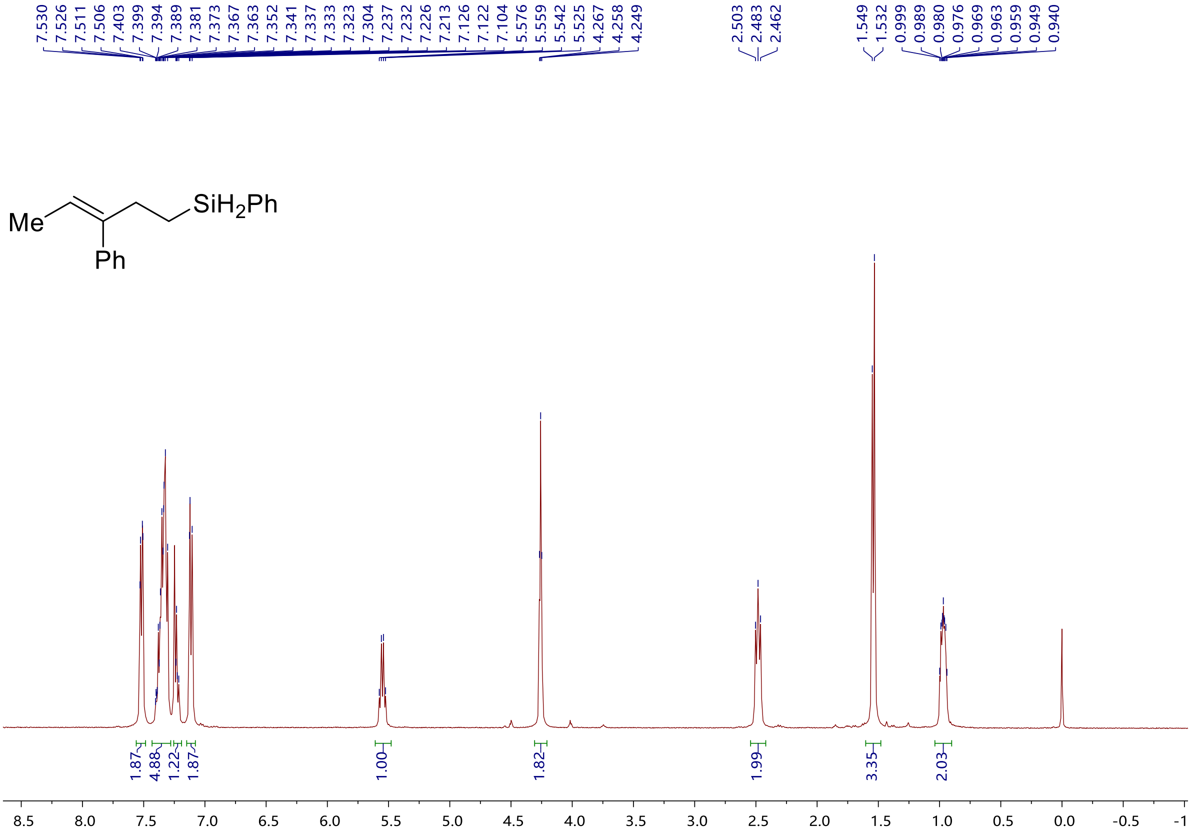

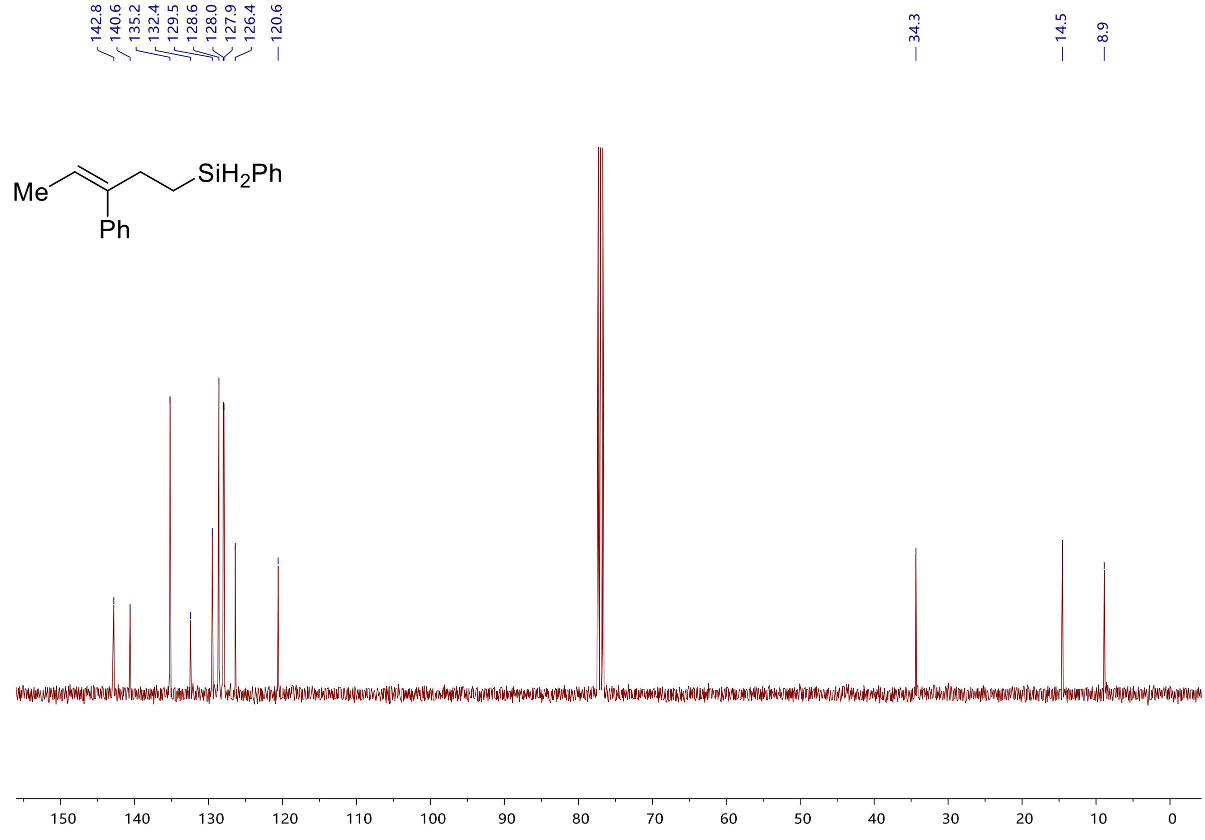

(*E*)-non-3-en-1-yl(phenyl)silane (3pa)

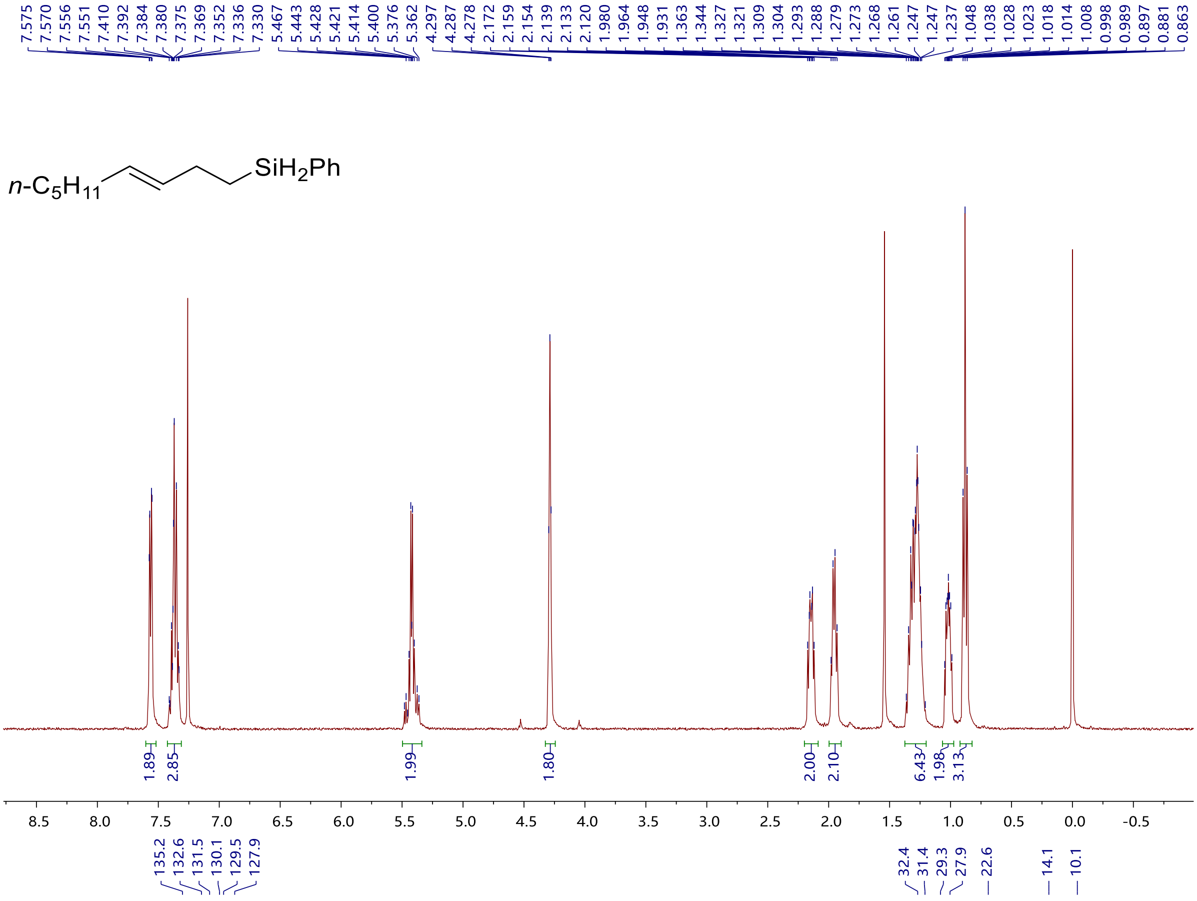

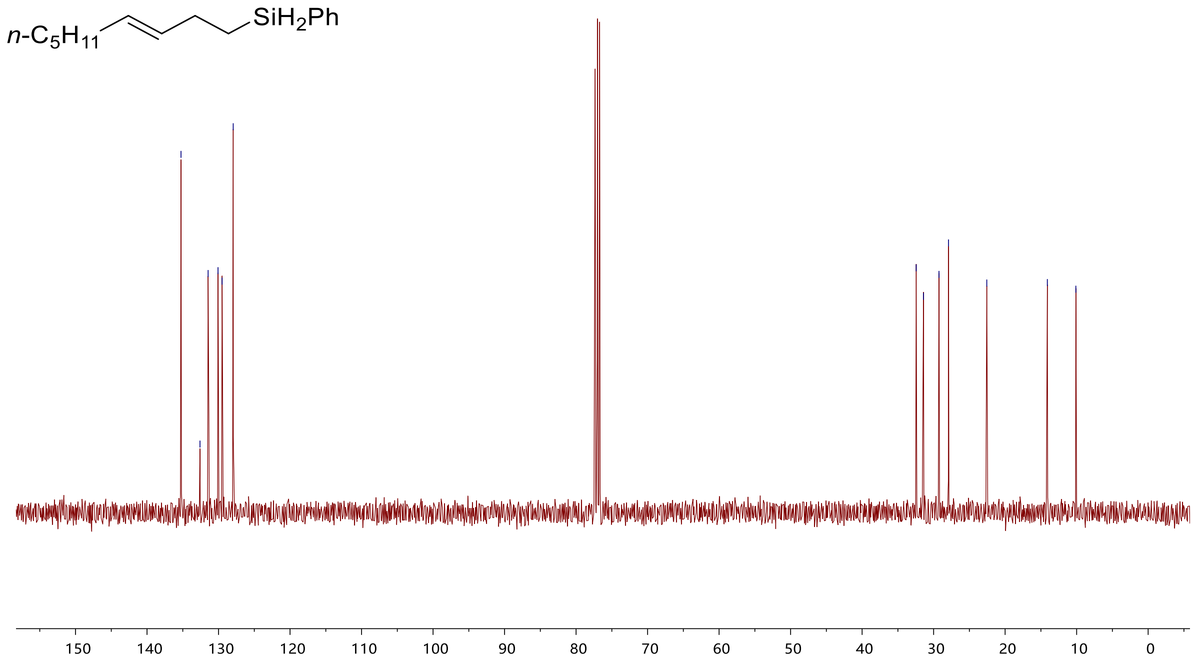

(*E*)-(4-cyclohexylbut-3-en-1-yl)(phenyl)silane (3qa)

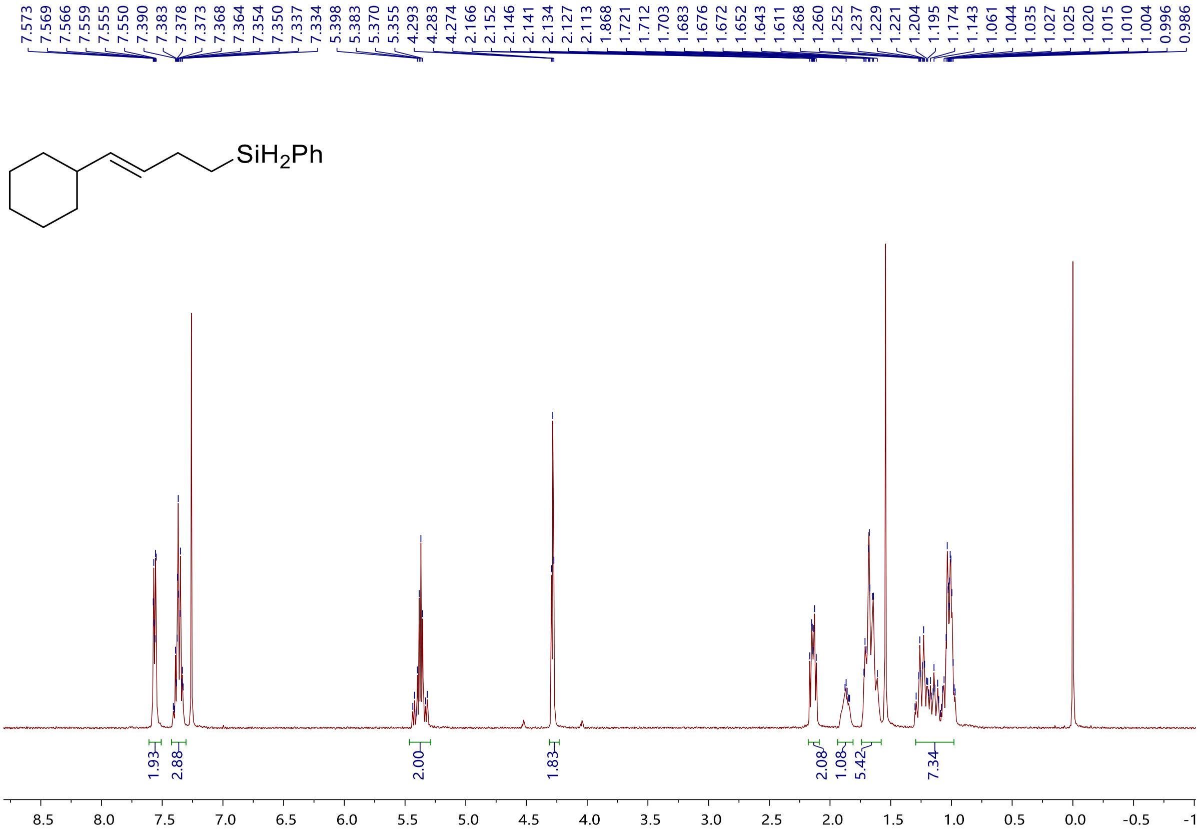

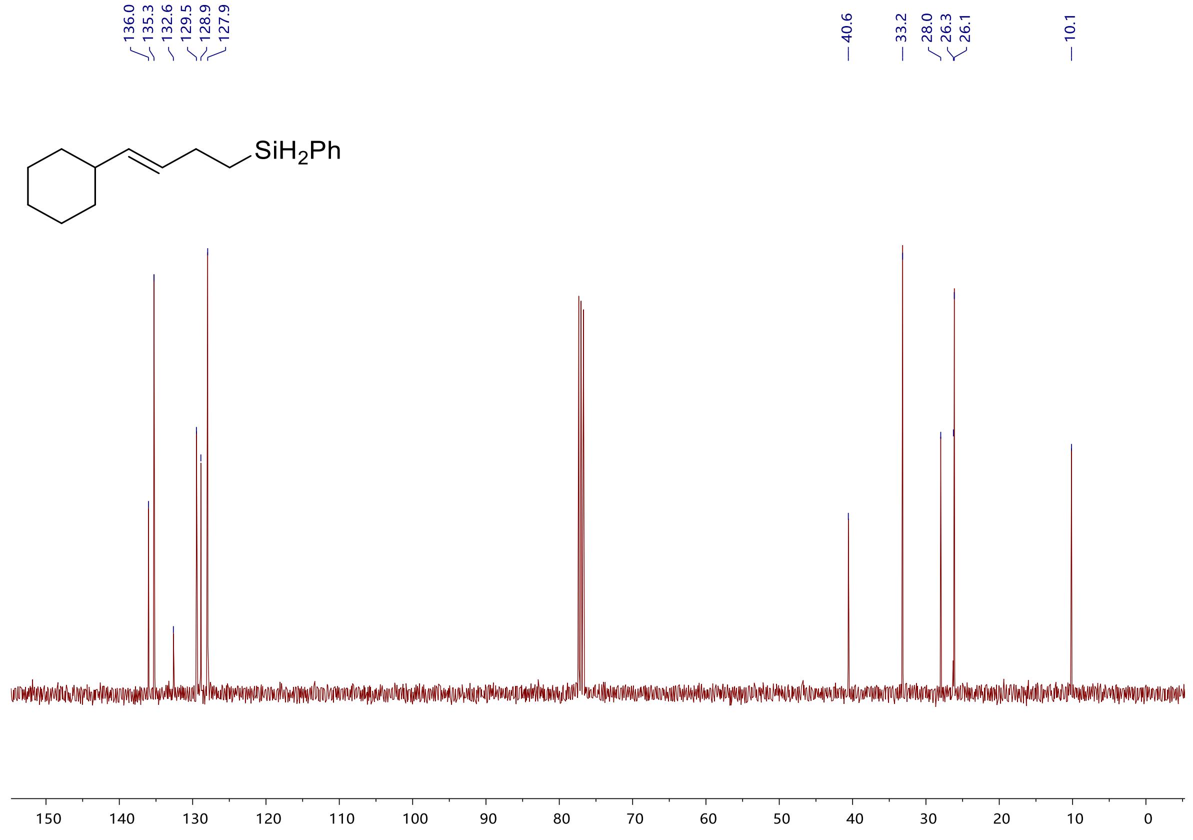

(4-(*tert*-butyl)phenyl)(3-methylbut-3-en-1-yl)silane (3re)

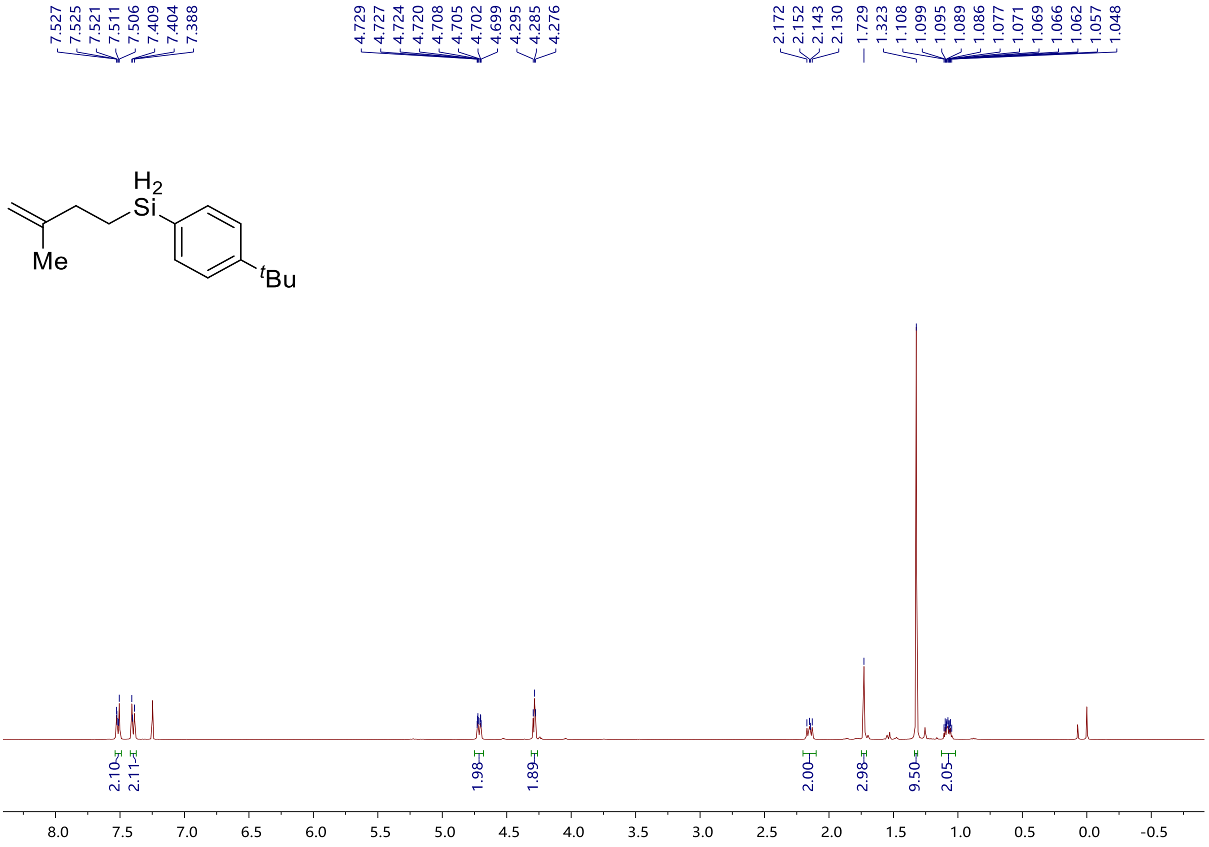

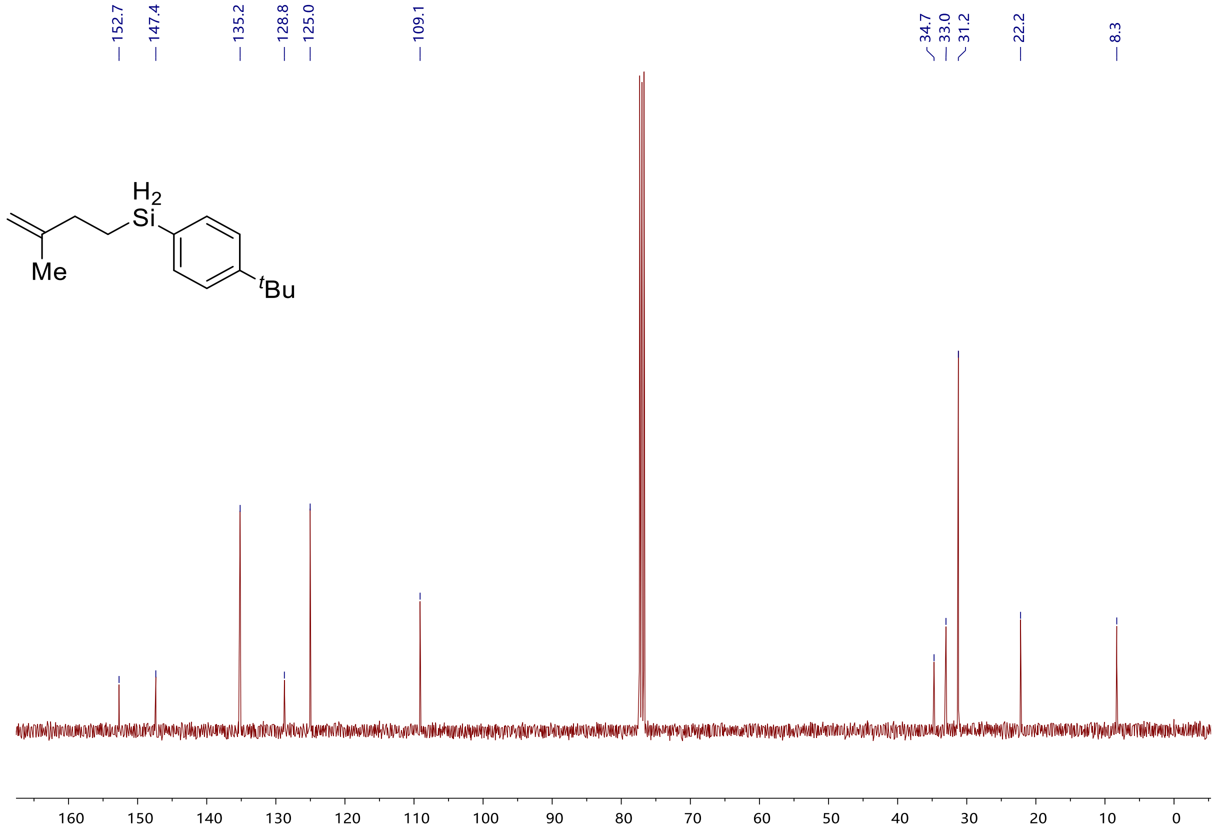

(7-methyl-3-methyleneoct-6-en-1-yl)(phenyl)silane (3sa)

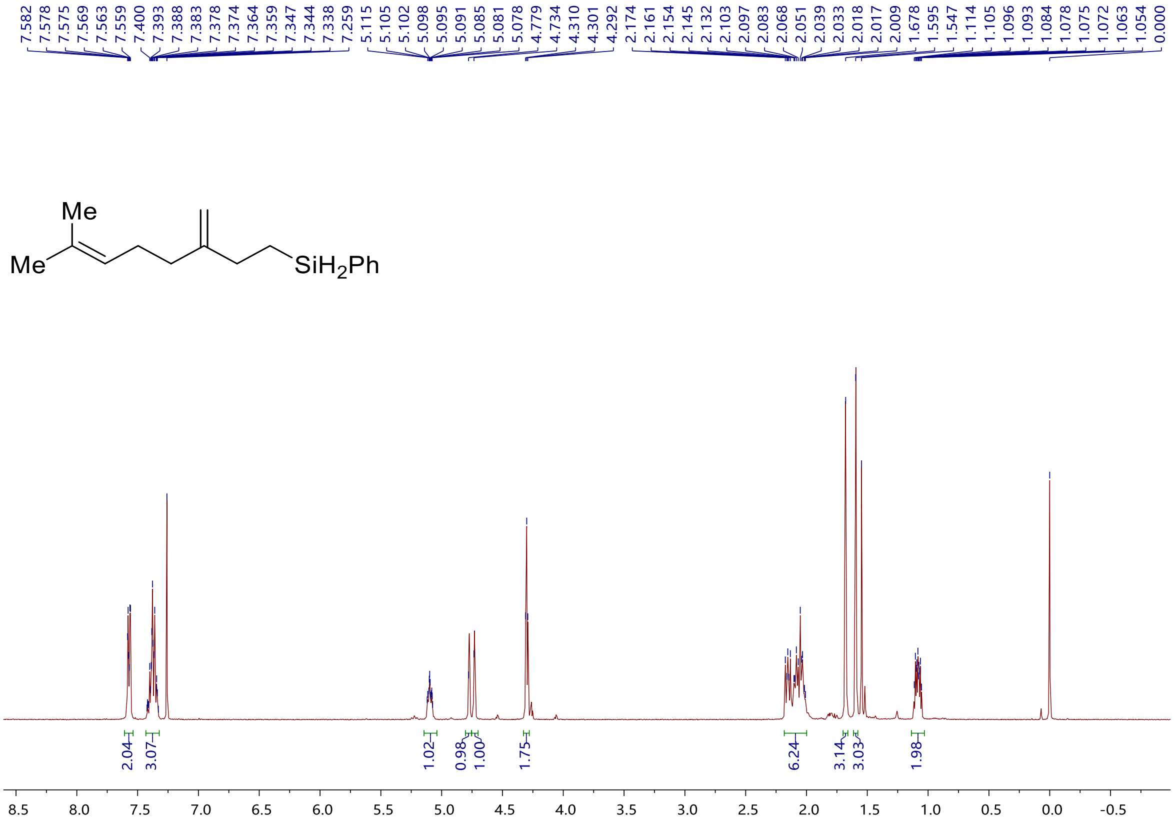

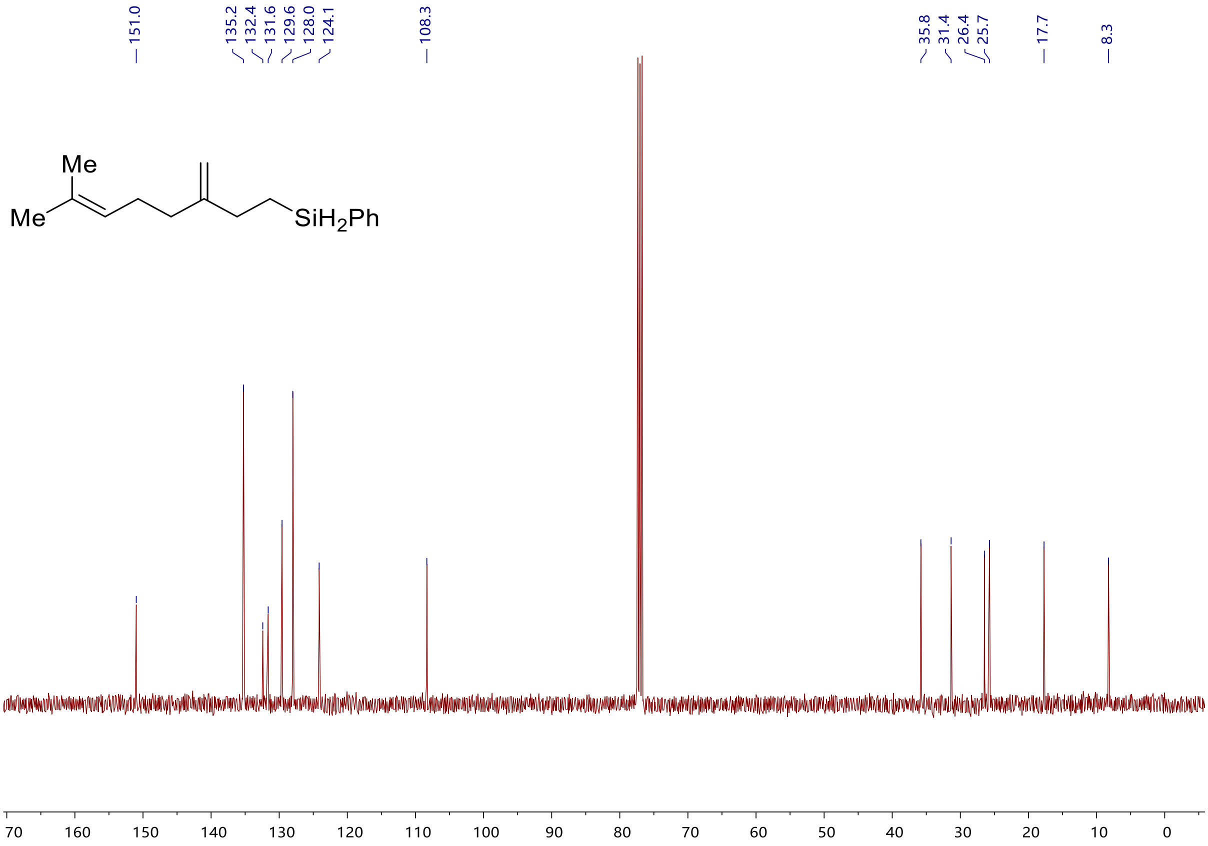

(3-cyclohexylidenepropyl)(phenyl)silane (3ta)

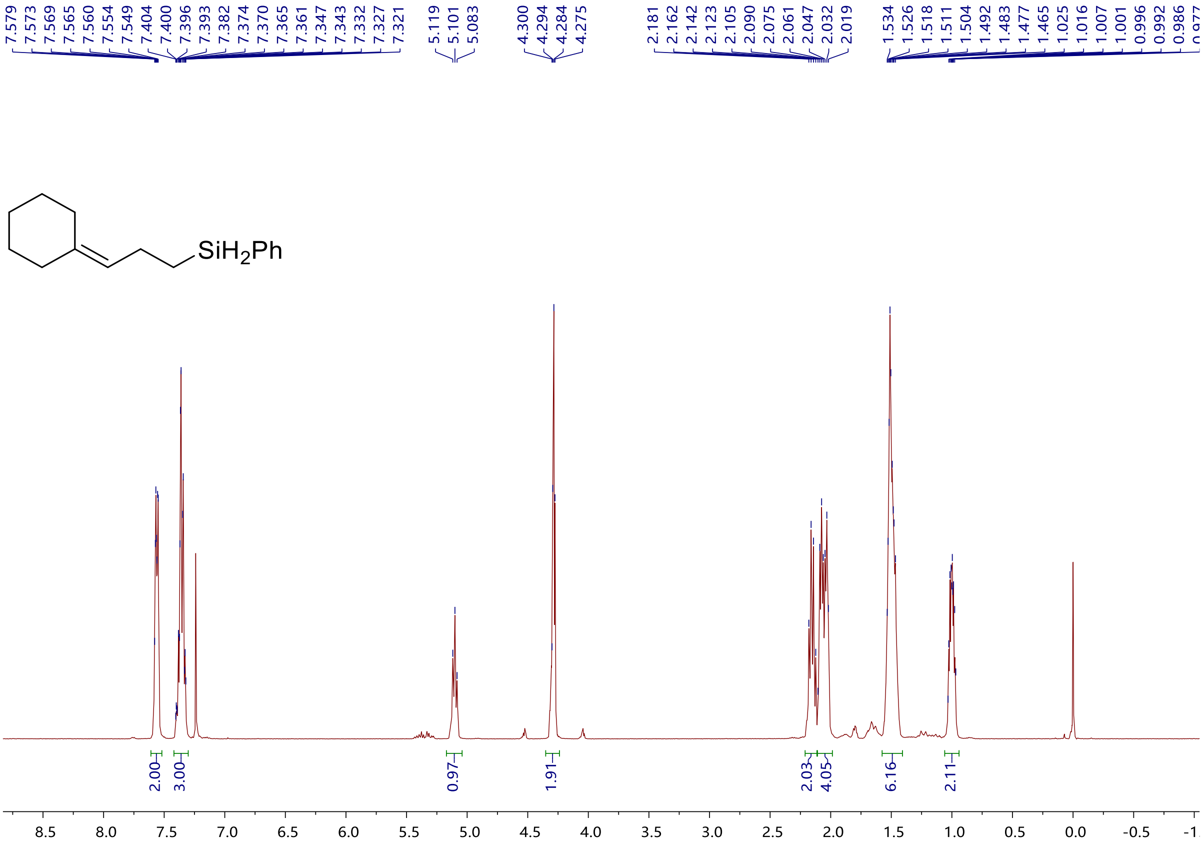

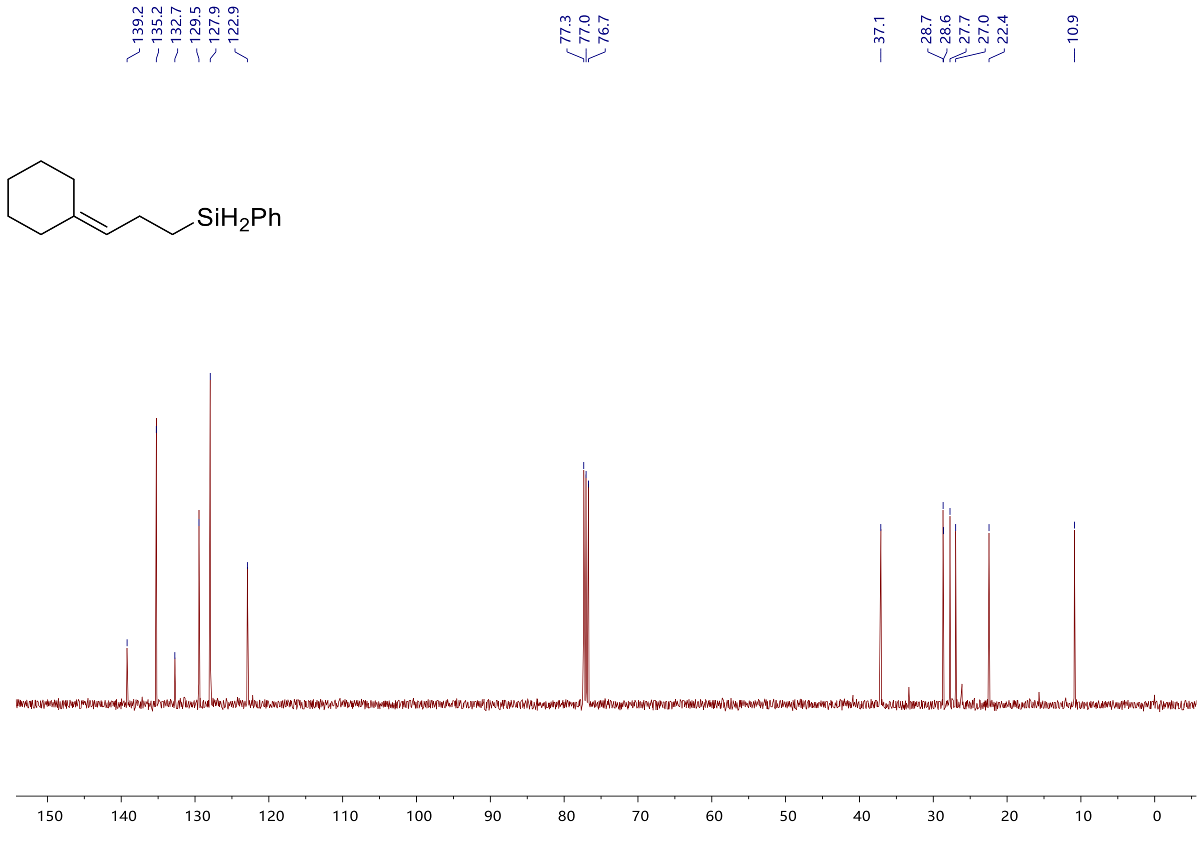

(*E*)-(4,8-dimethylnona-3,7-dien-1-yl)(phenyl)silane (3ua)

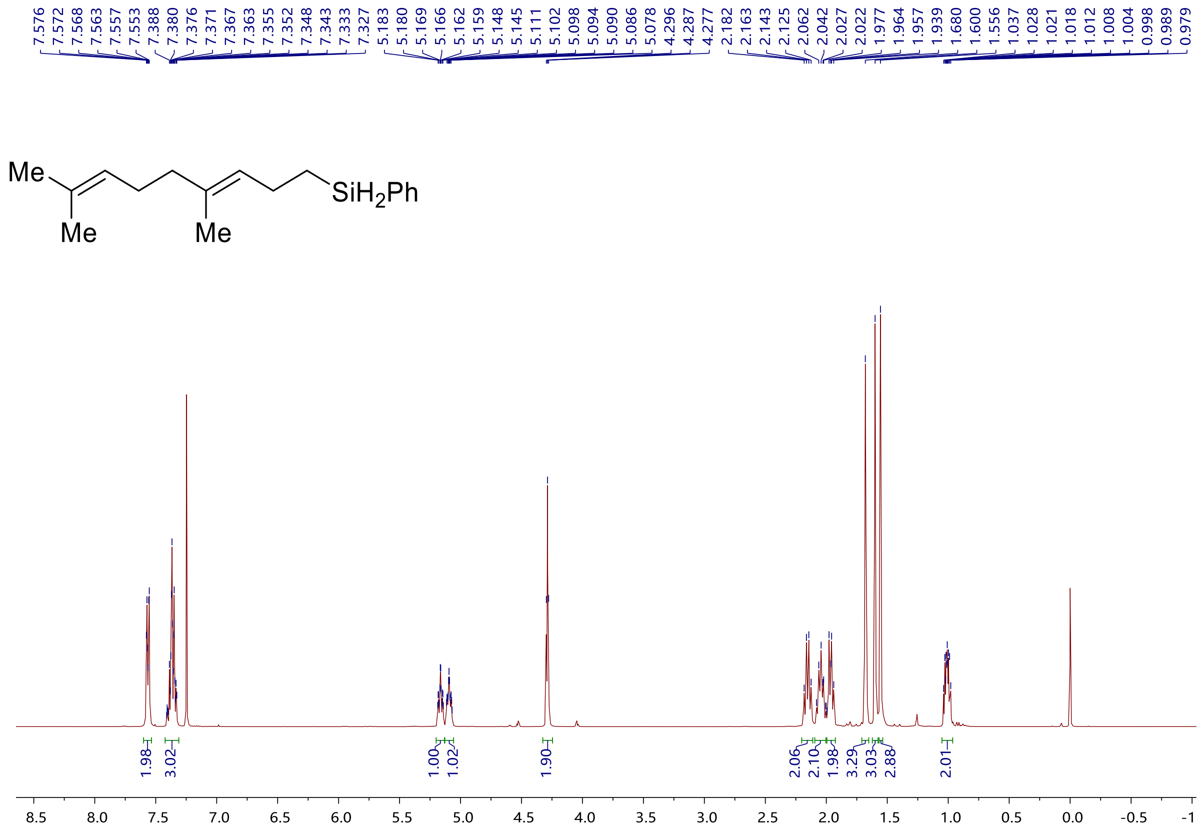

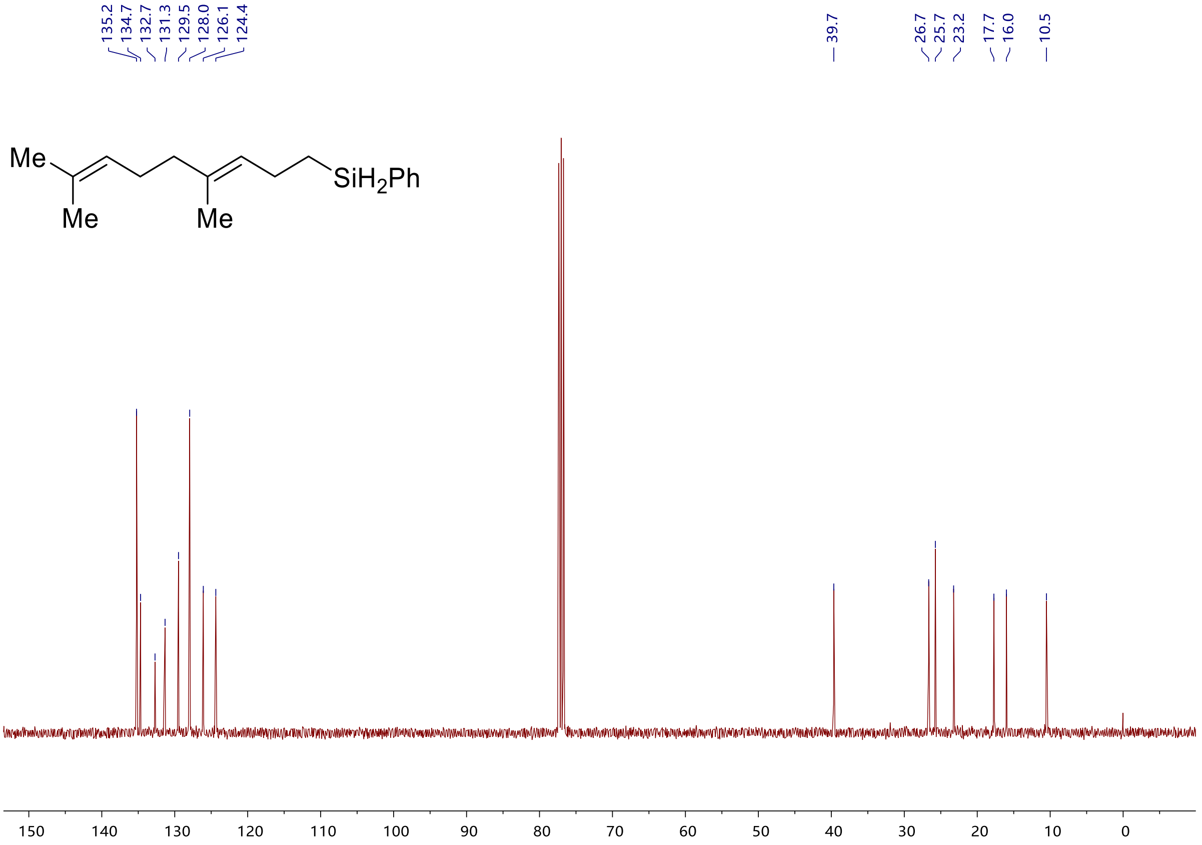

((3*E*,5*E*)-4-methyl-6-(2,6,6-trimethylcyclohex-1-en-1-yl)hexa-3,5-dien-1-yl)(phenyl)silane (3va)

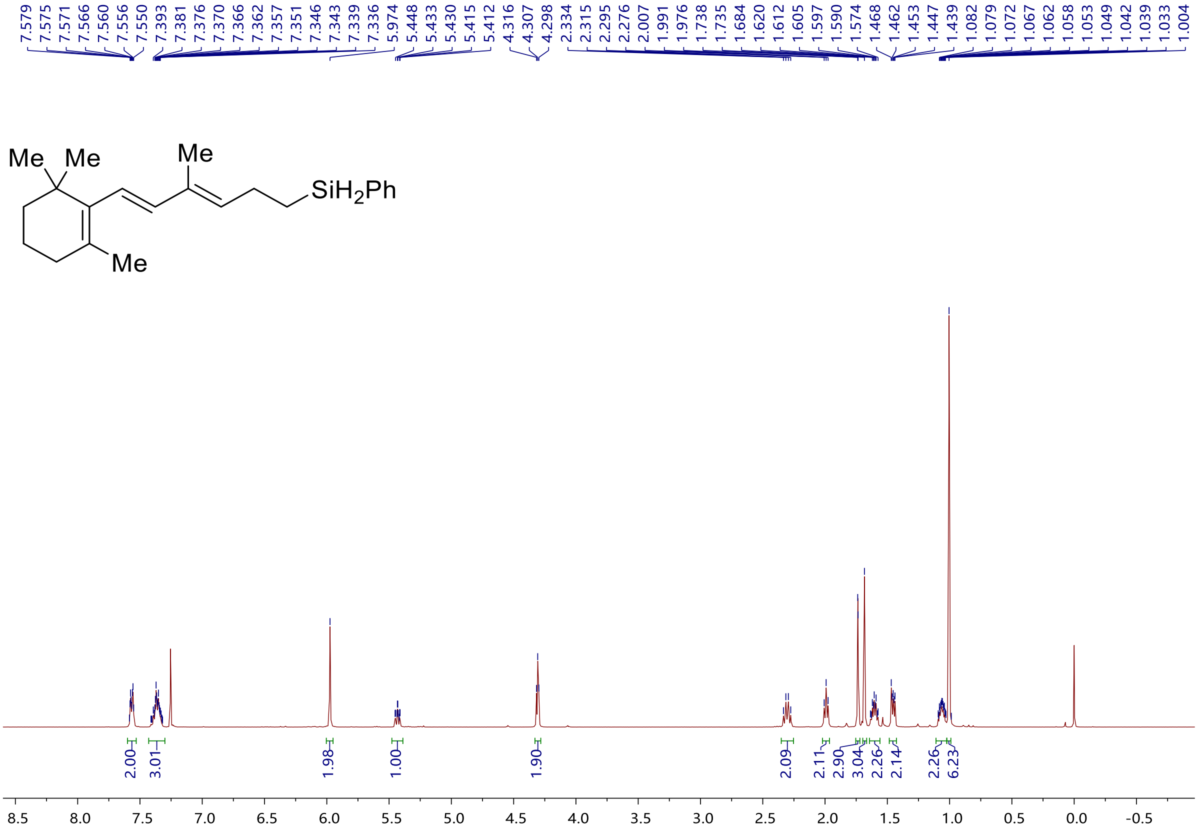

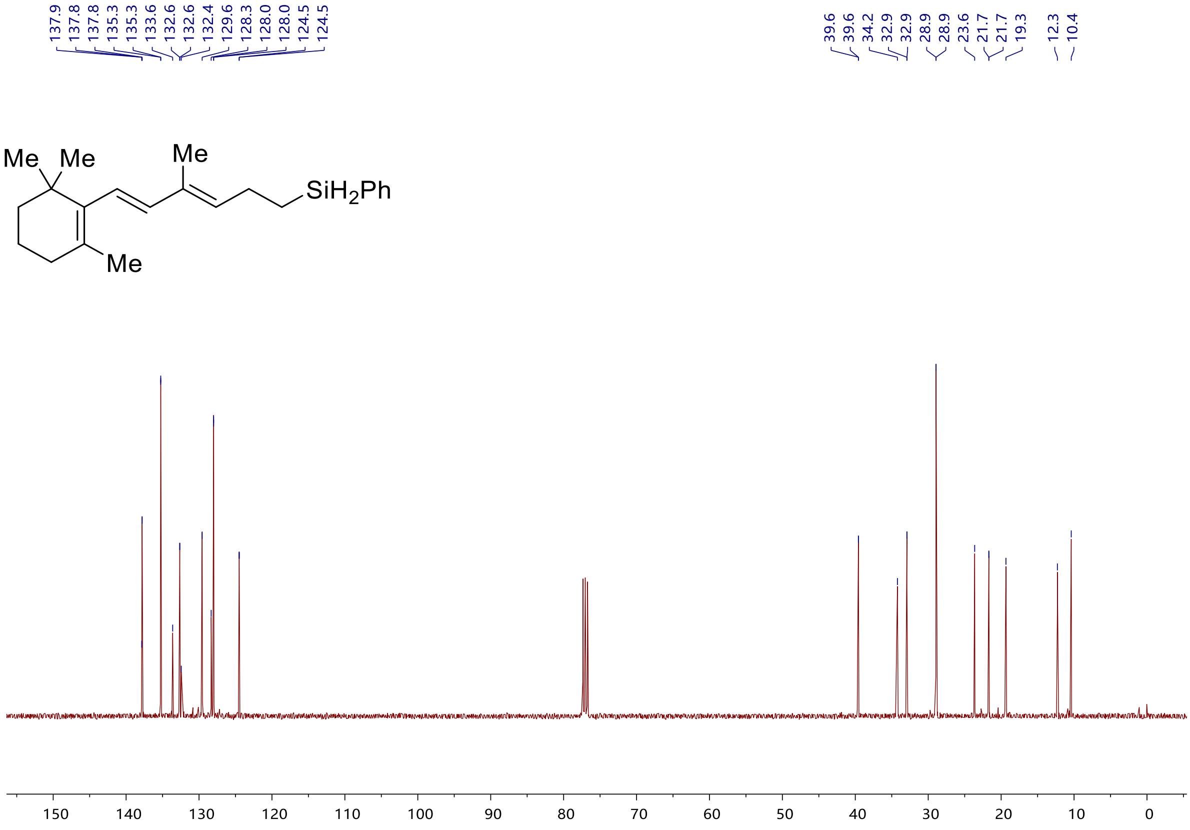

(*S*)-phenyl(2-(4-(prop-1-en-2-yl)cyclohex-1-en-1-yl)ethyl)silane (3wa)

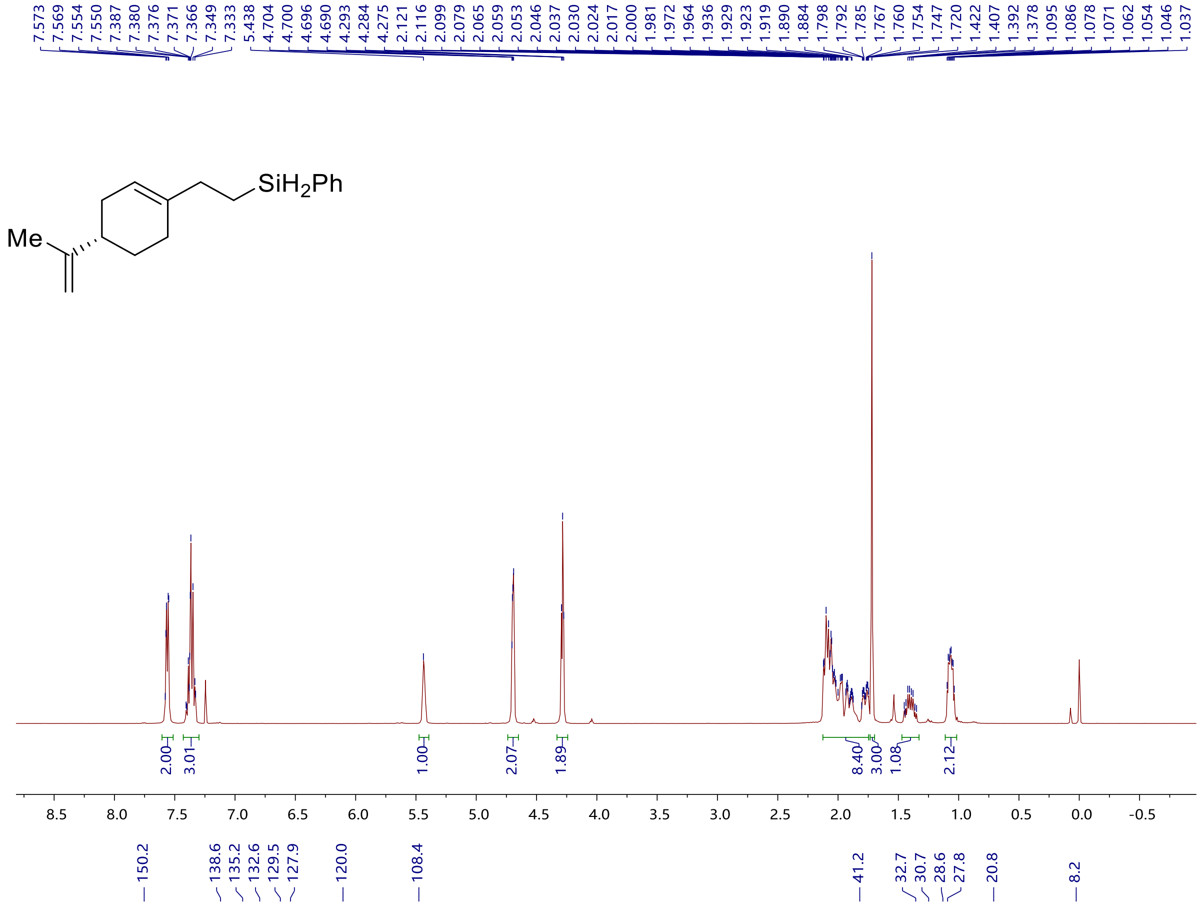

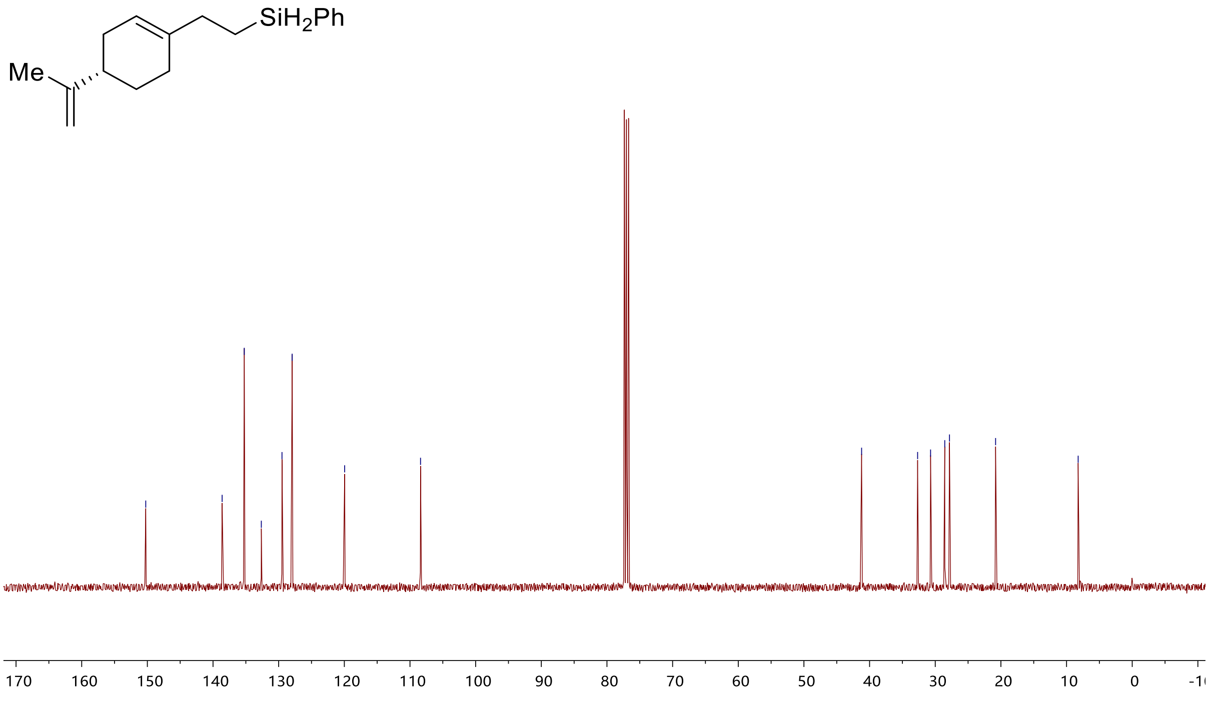

(2-(cyclohept-1-en-1-yl)ethyl)(phenyl)silane (3xa)

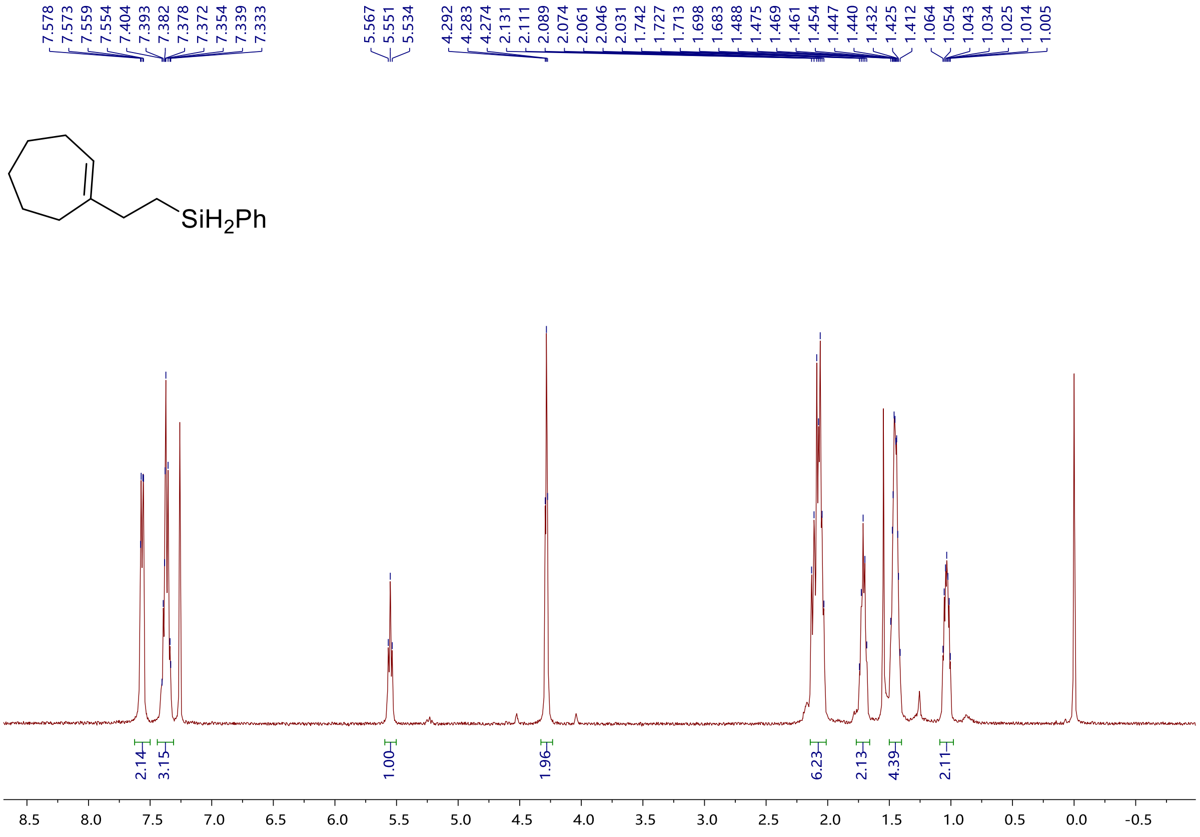

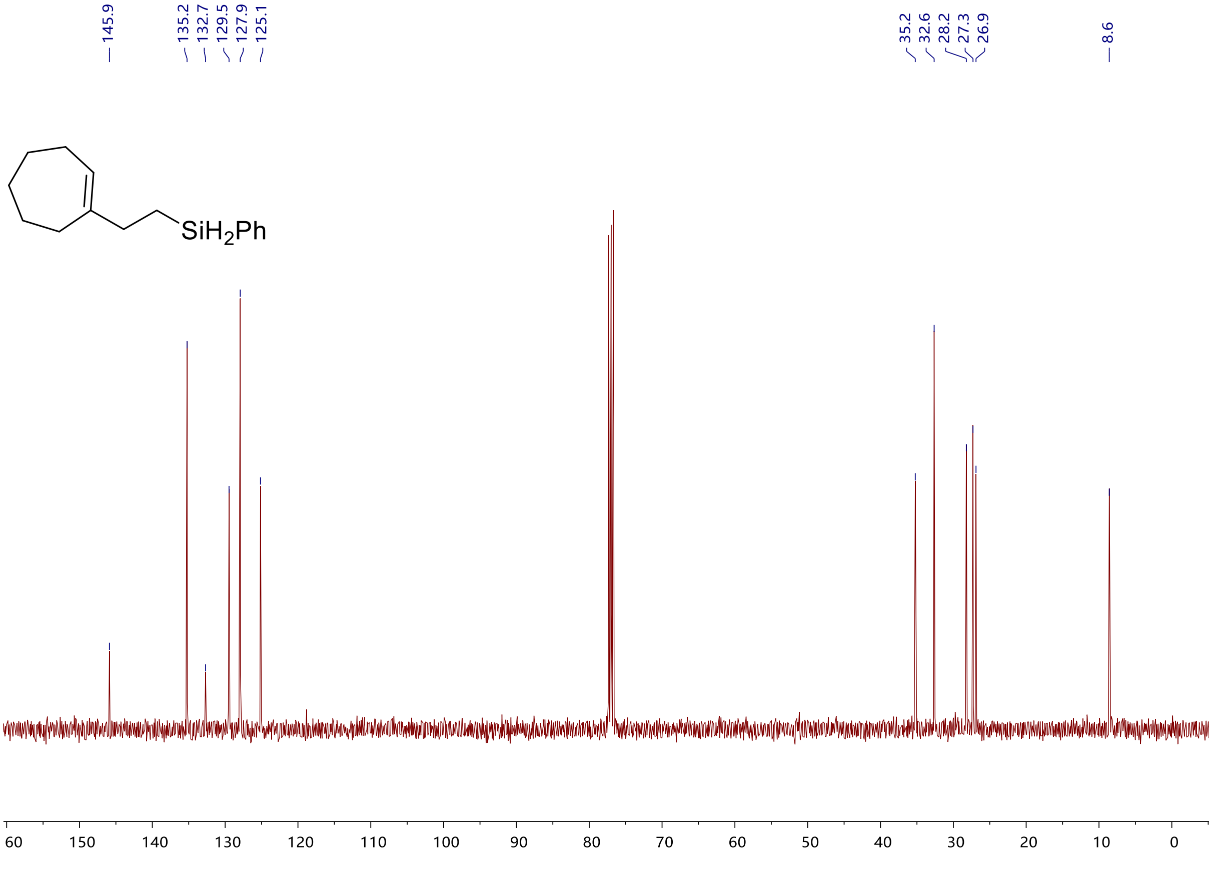

phenethyl(phenyl)silane (8aa)

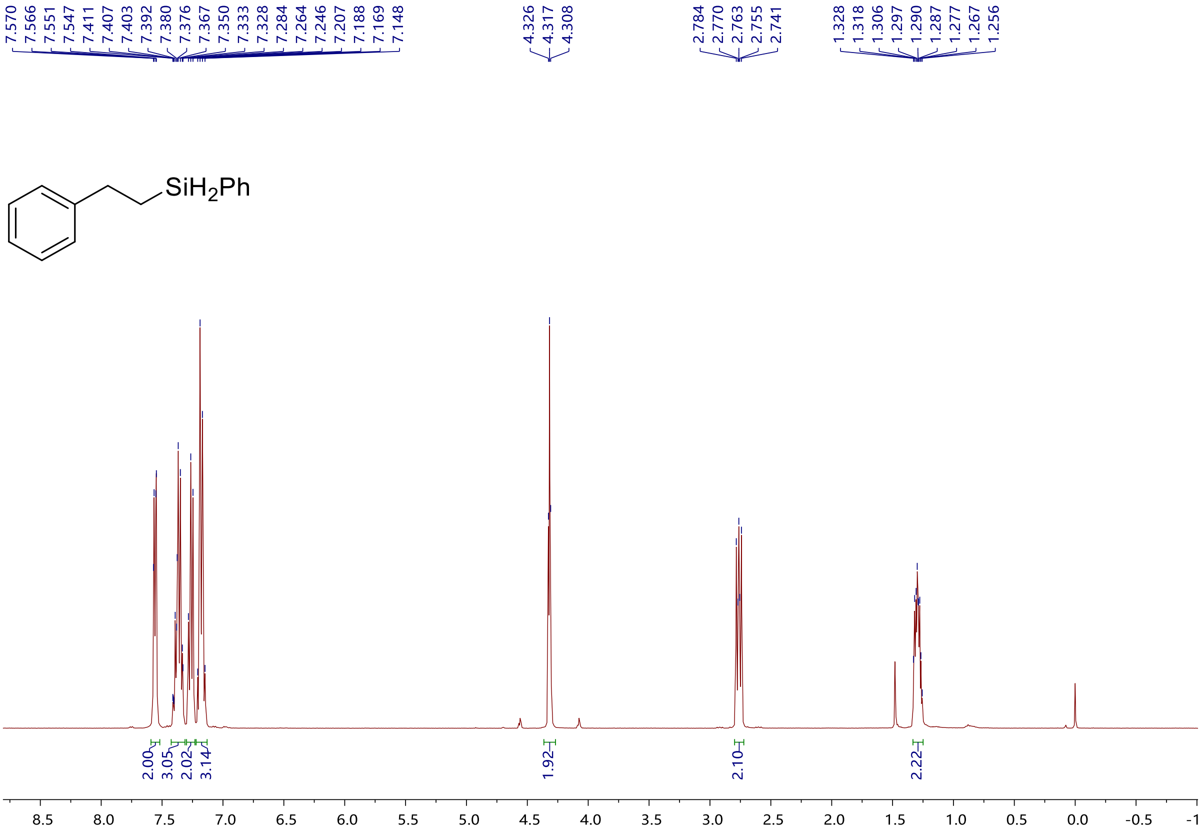

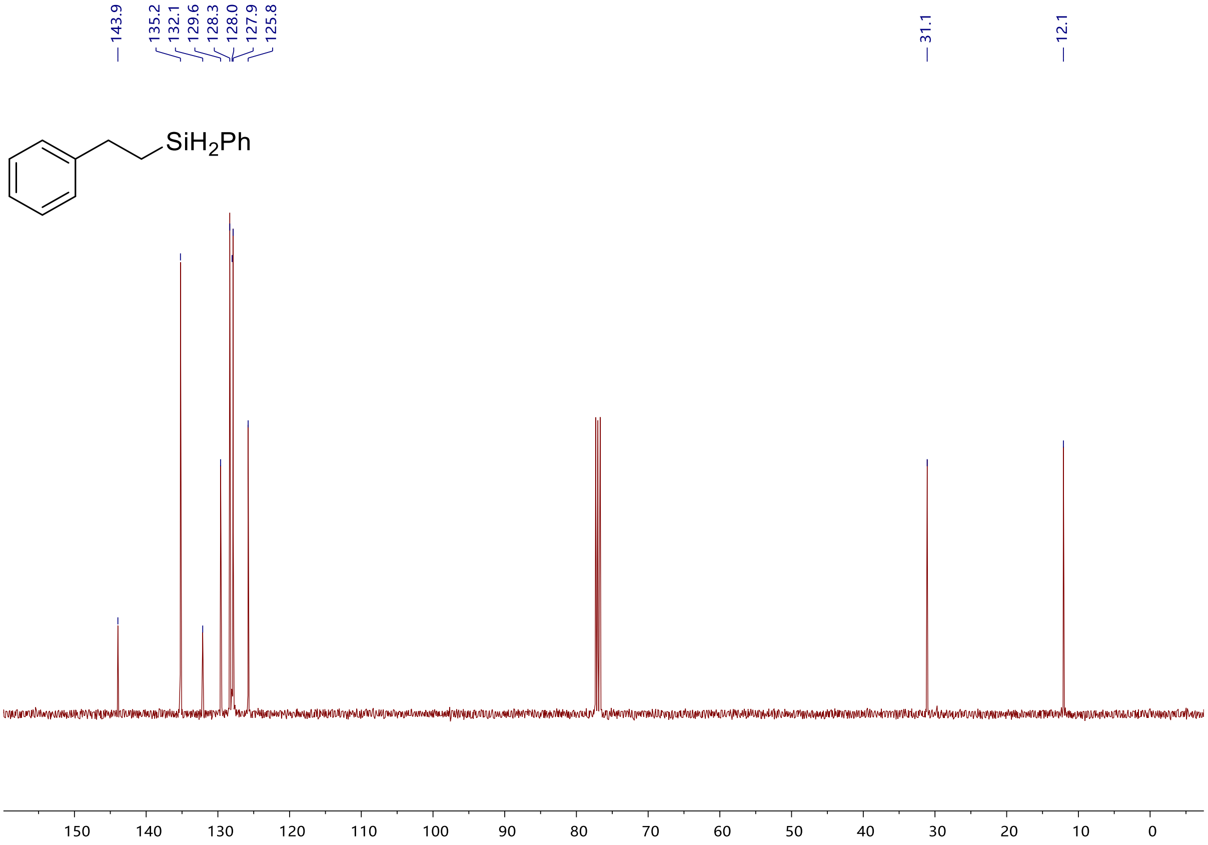

(3-methylphenethyl)(phenyl)silane (8ba)

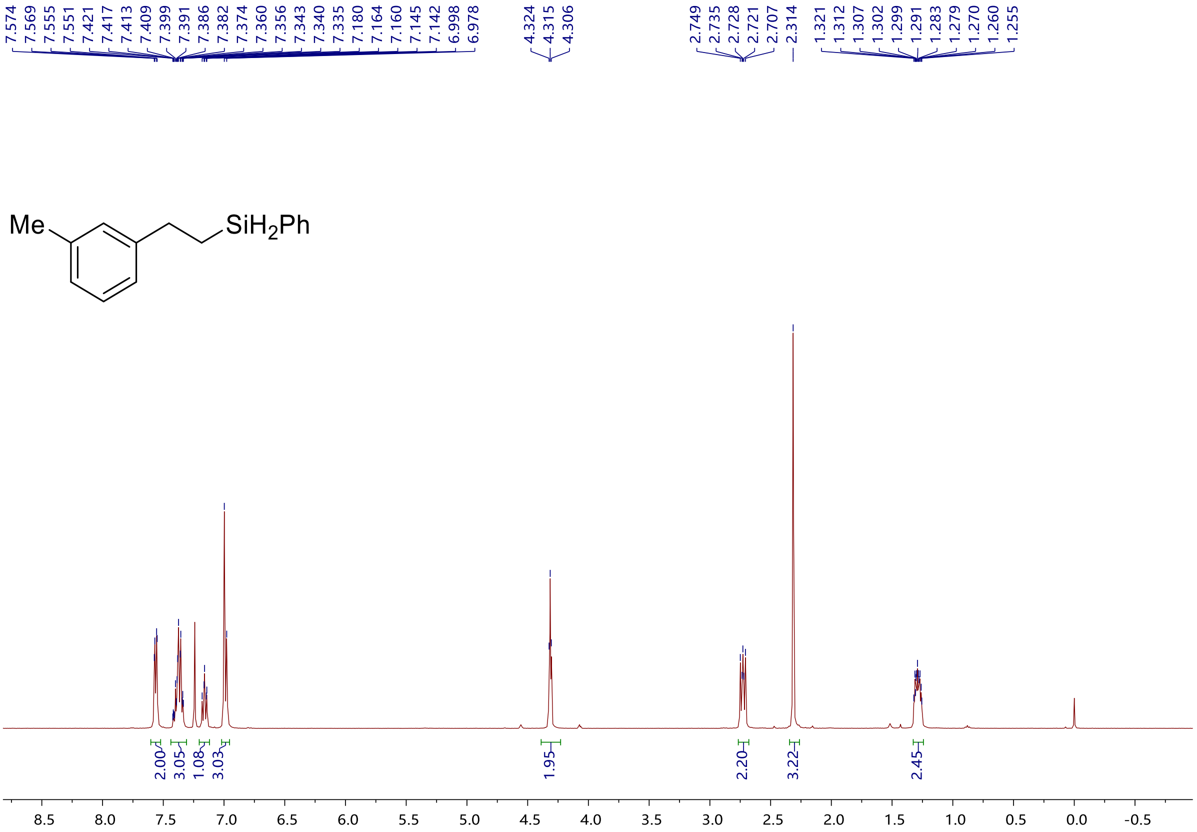

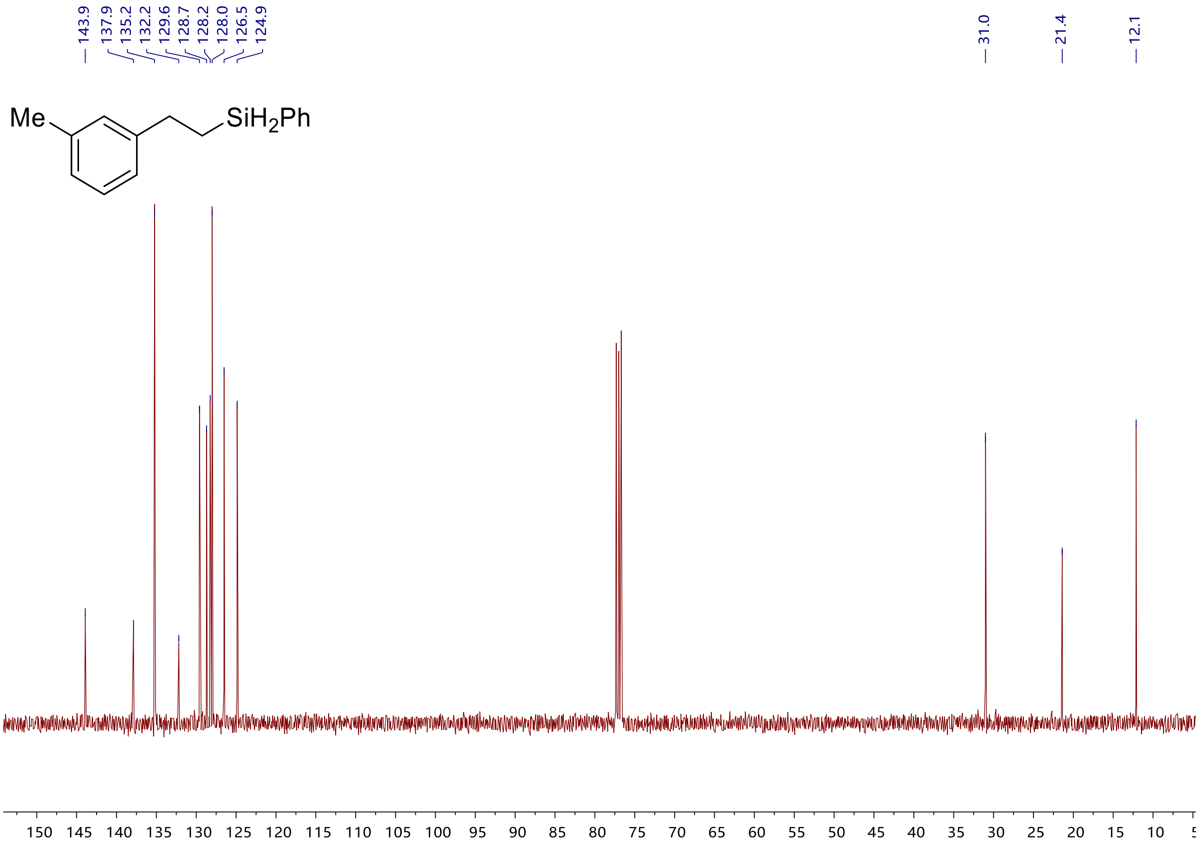

(4-fluorophenethyl)(phenyl)silane (8ca)

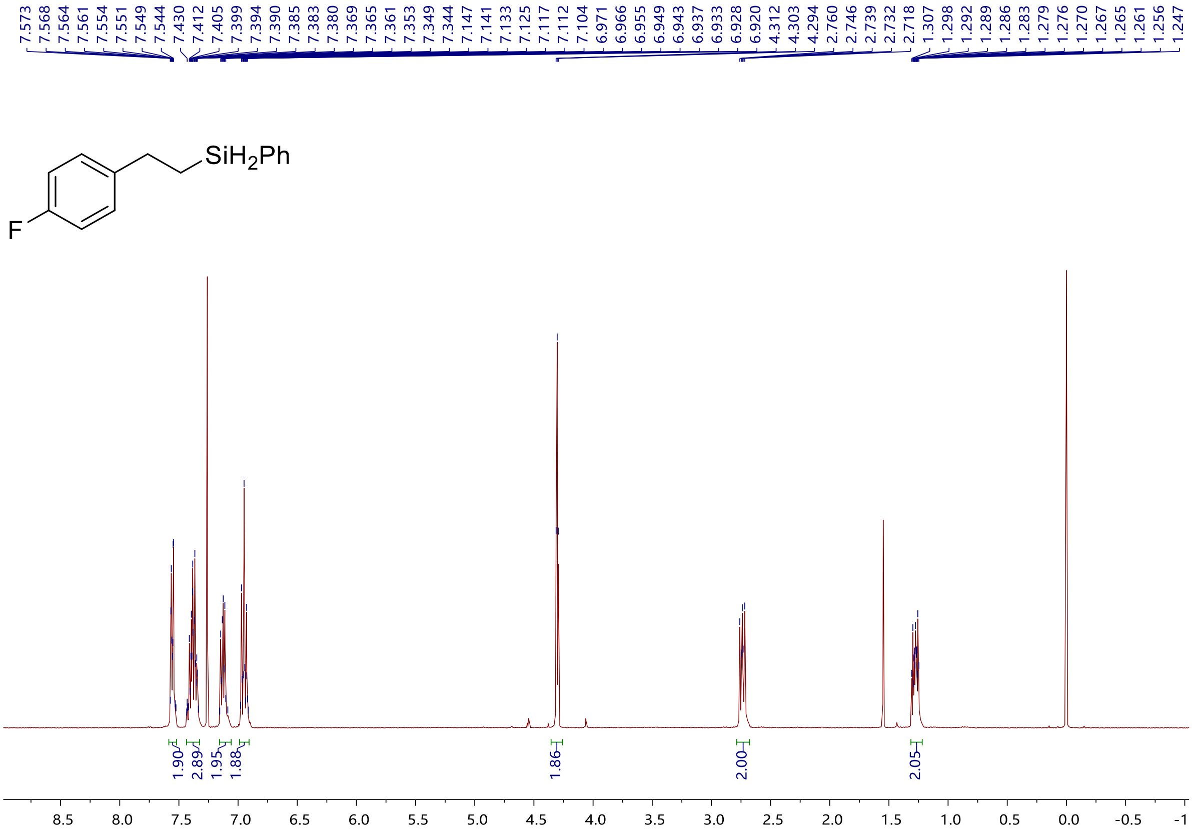

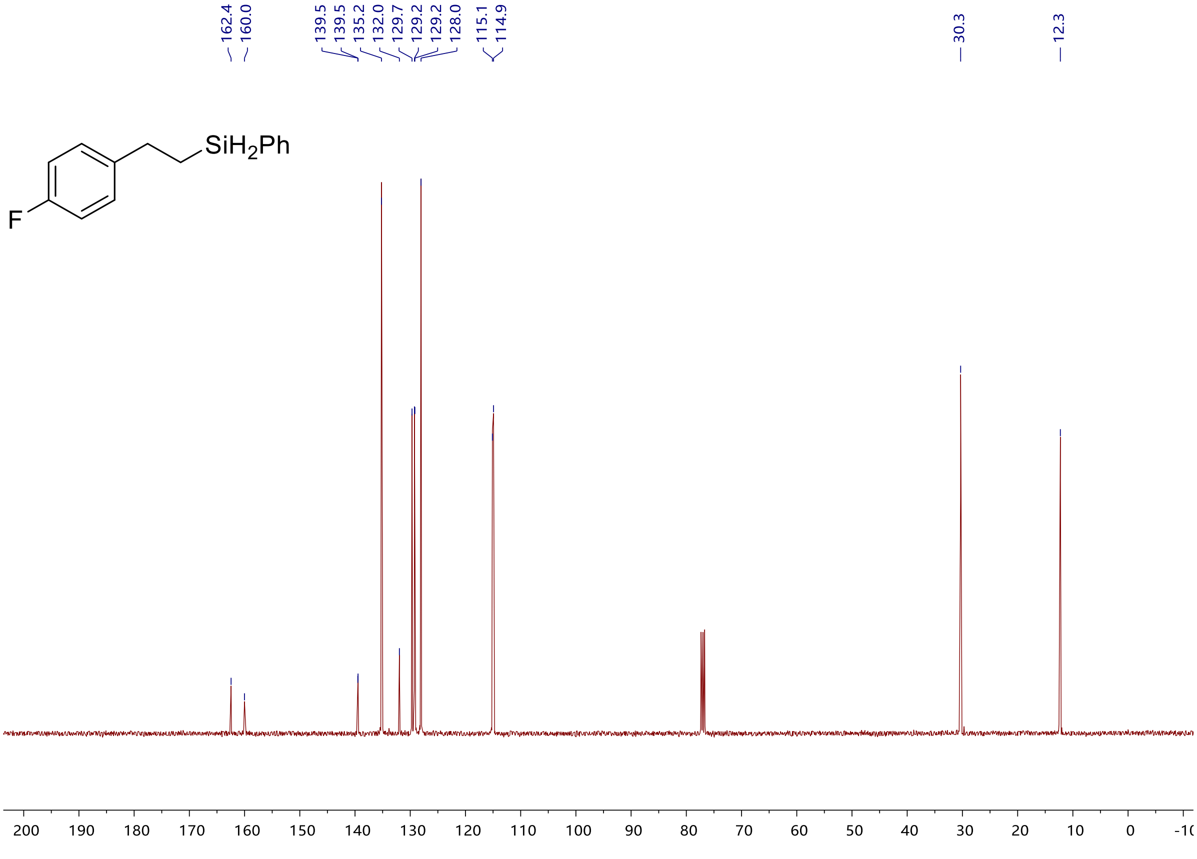

(4-methoxyphenethyl)(phenyl)silane (8da)

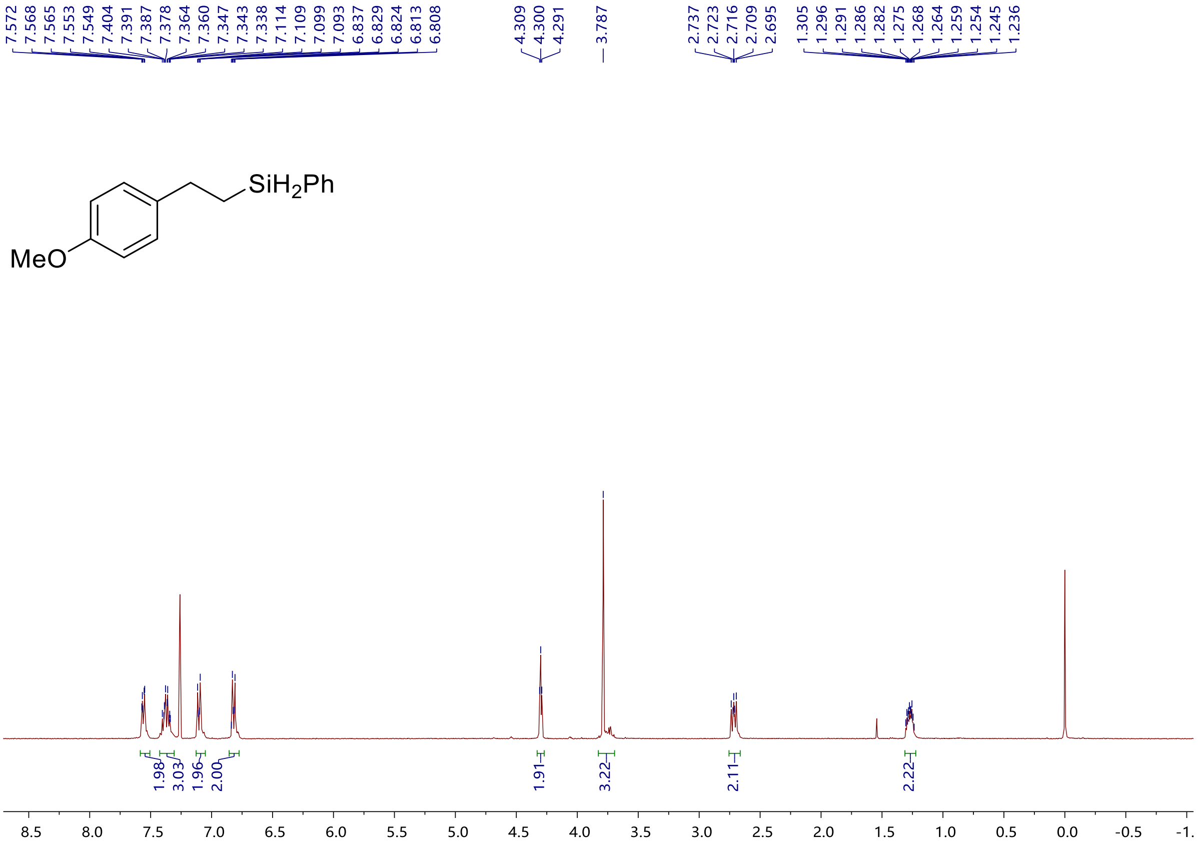

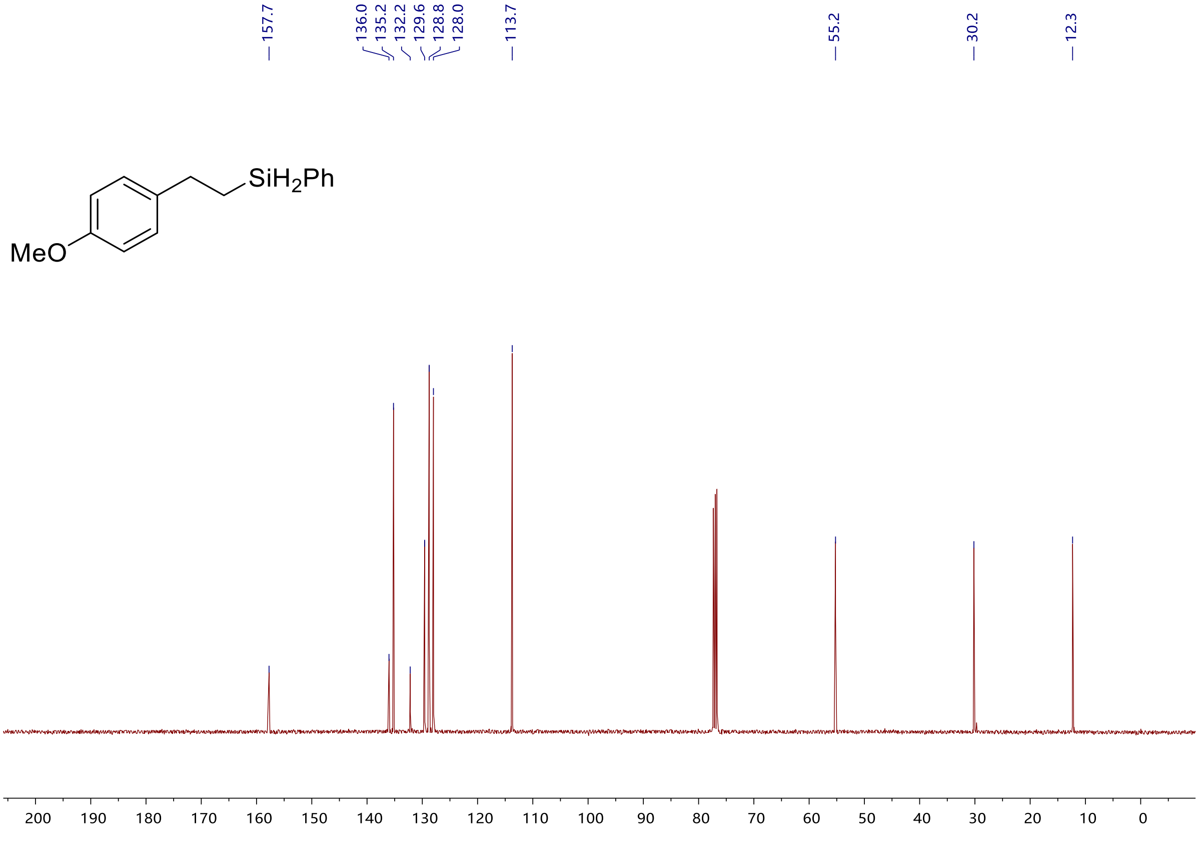

(4-(*tert*-butyl)phenethyl)(phenyl)silane (8ea)

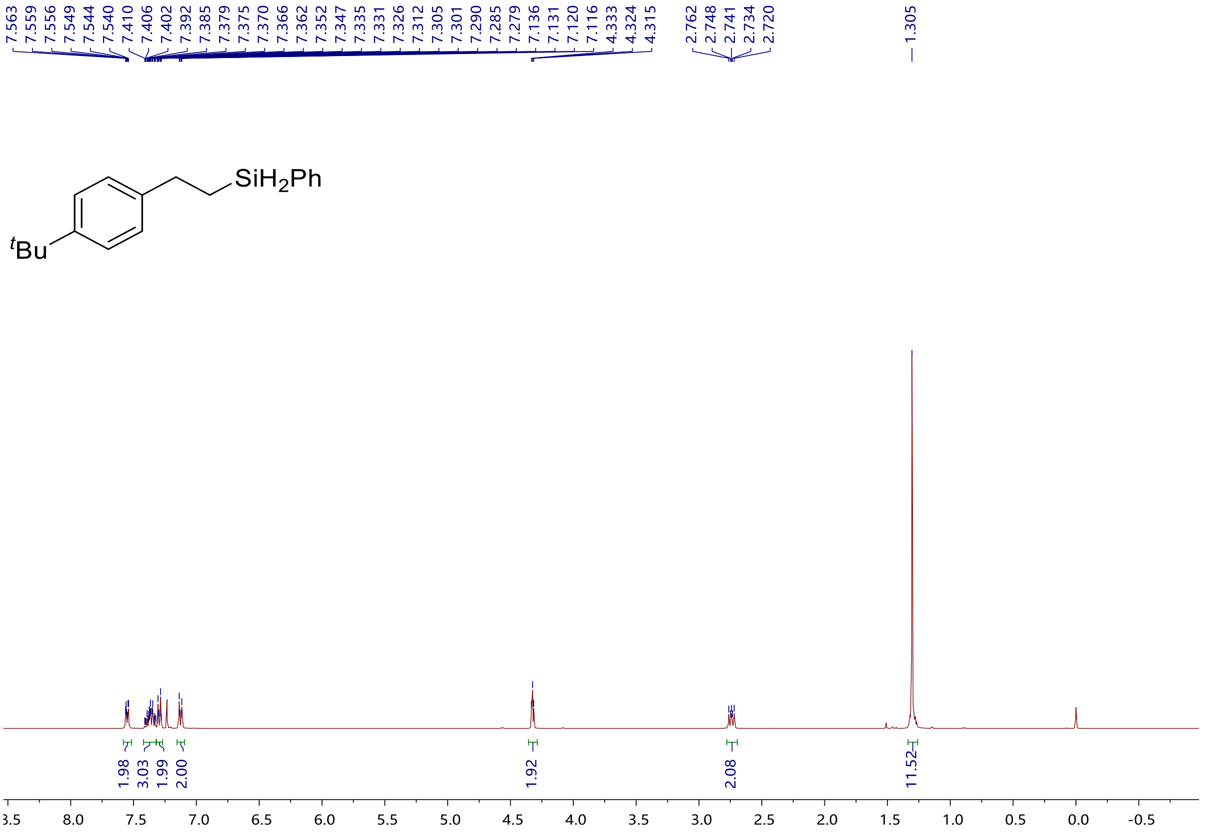

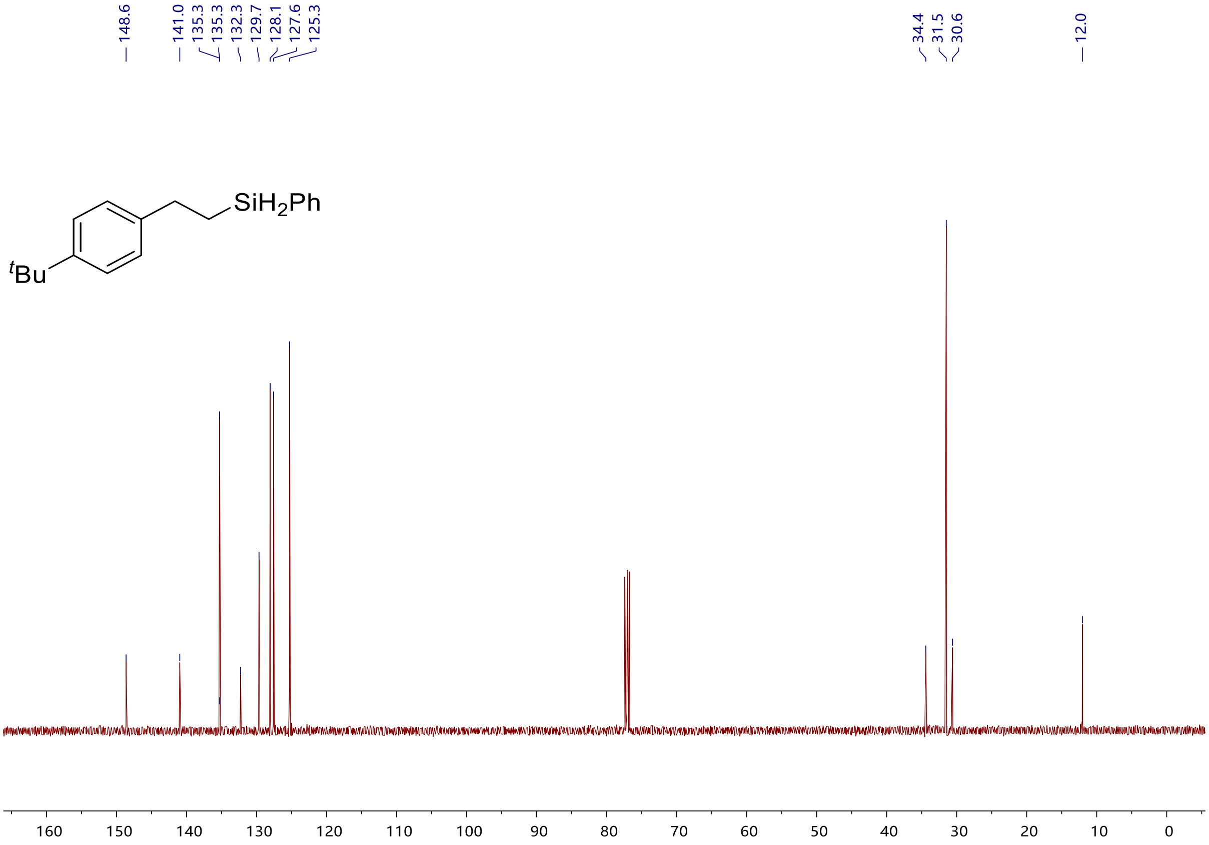

(2-([1,1'-biphenyl]-4-yl)ethyl)(phenyl)silane (8fa)

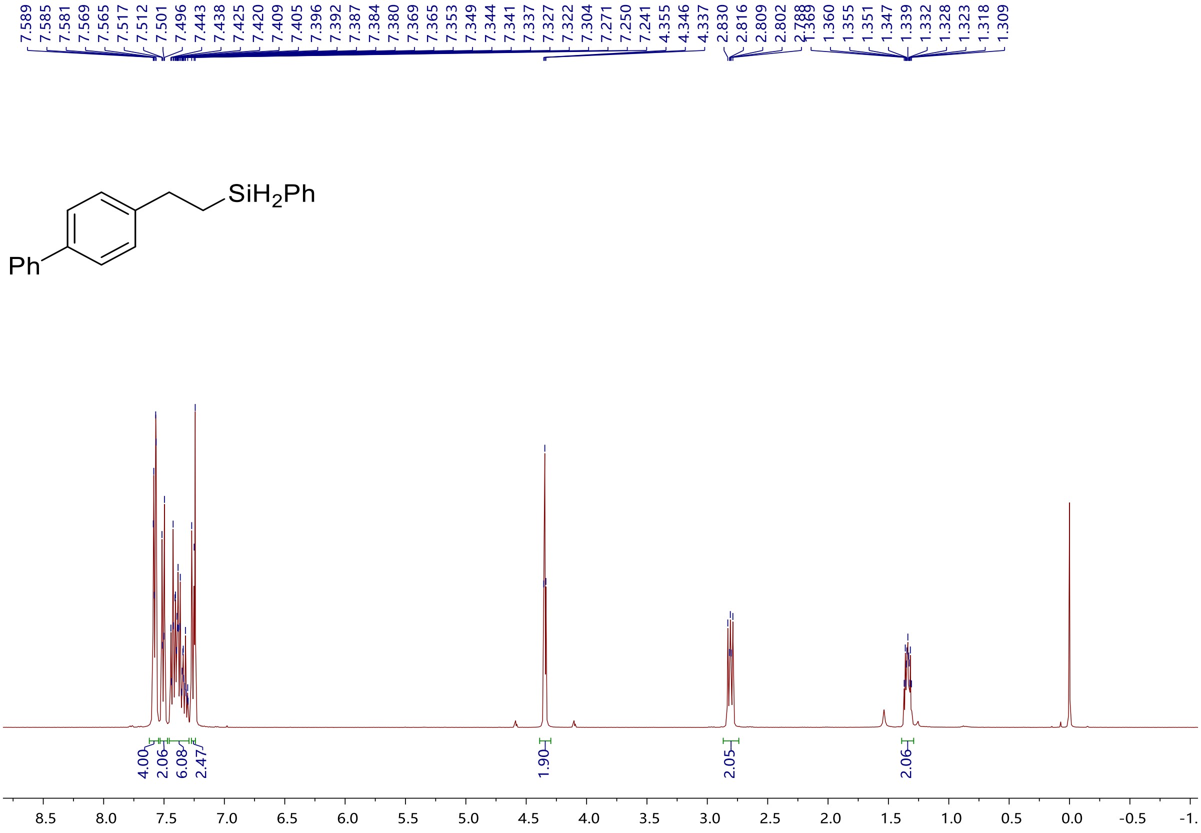

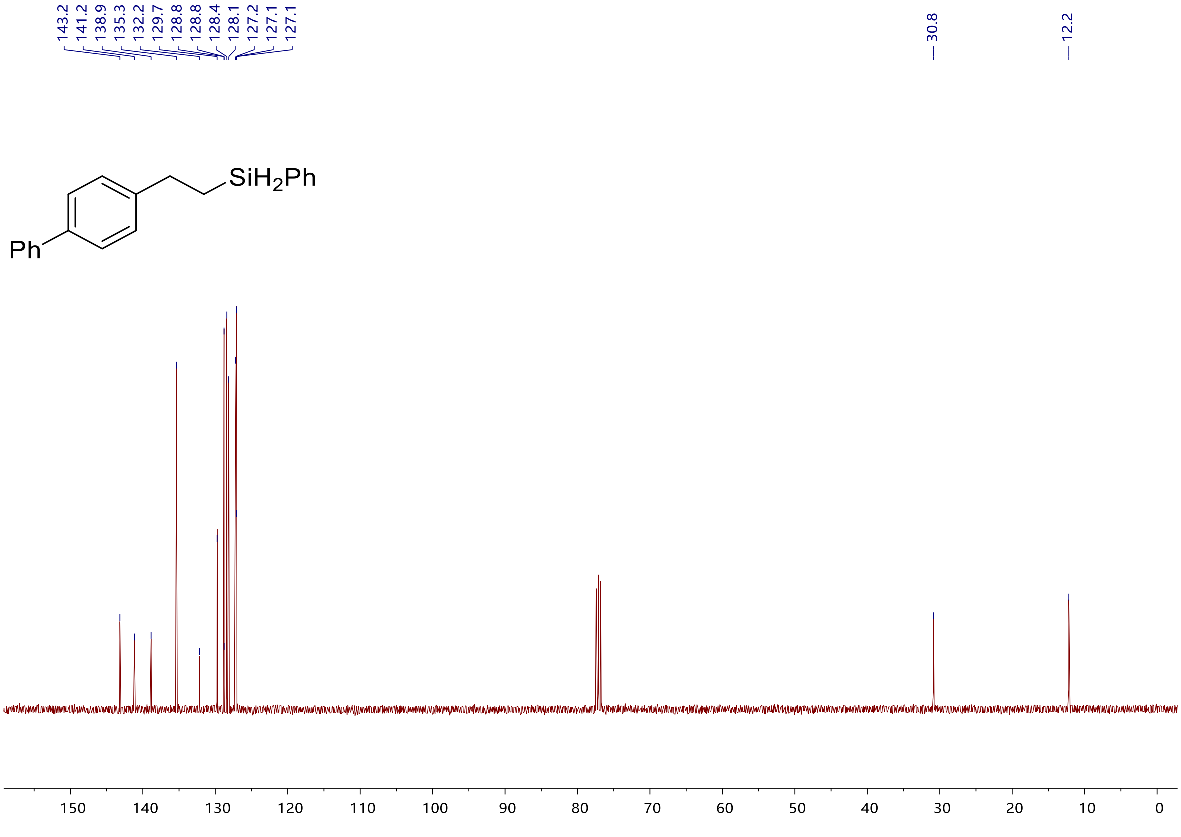

phenyl(6-phenylhexyl)silane (8ga)

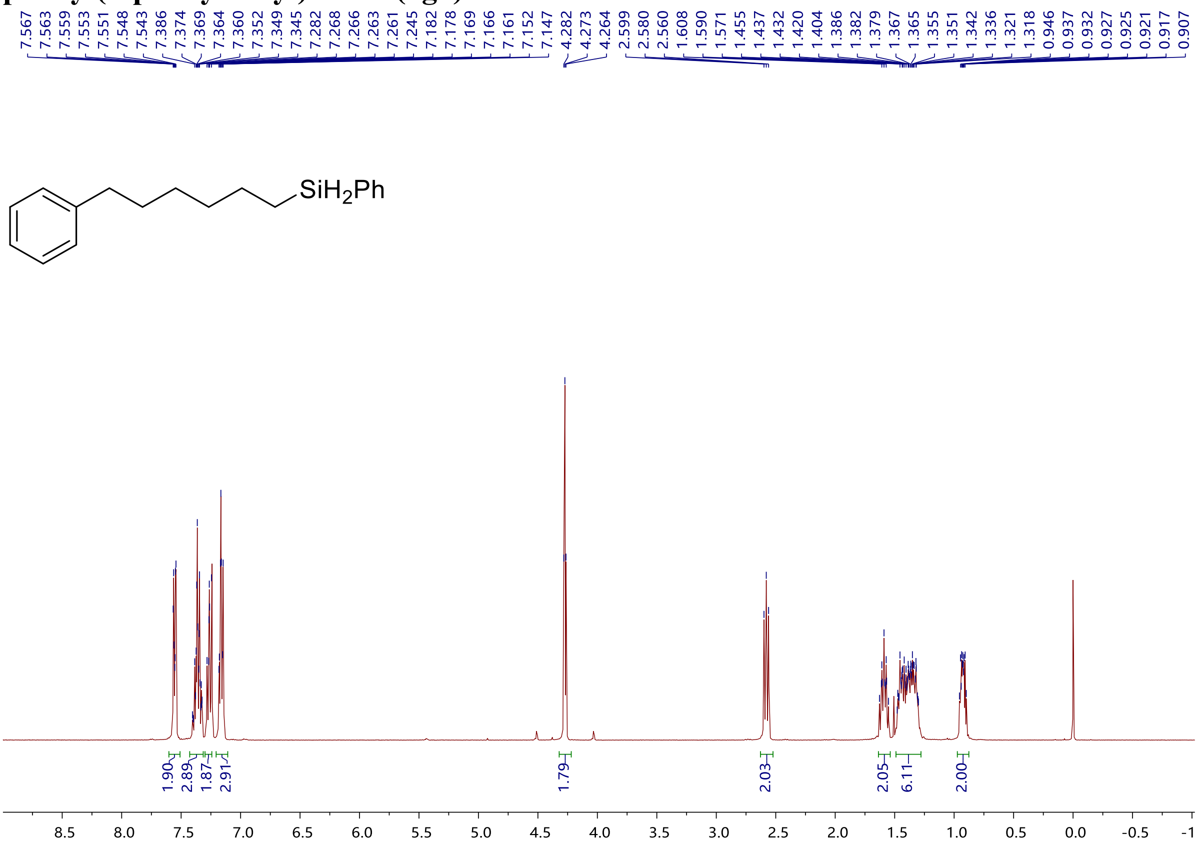

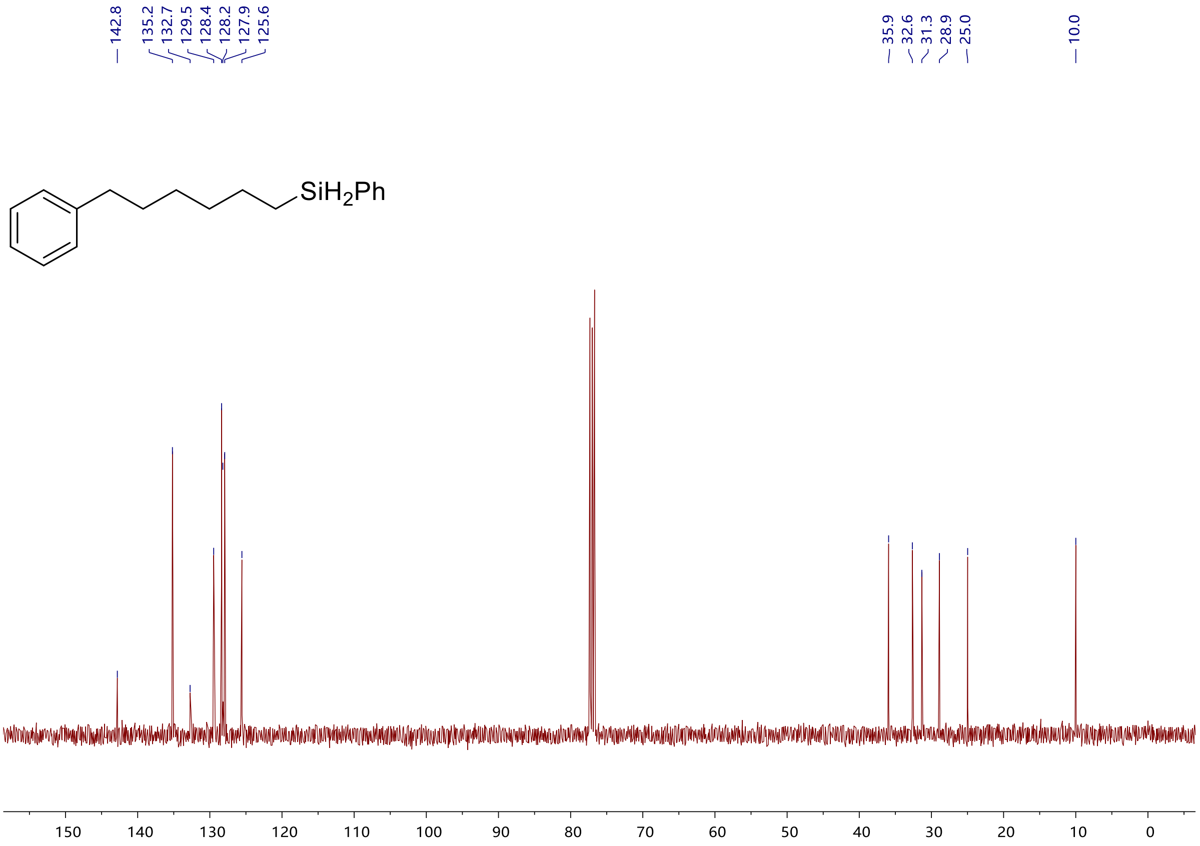

(6-chlorohexyl)(phenyl)silane (8ha)

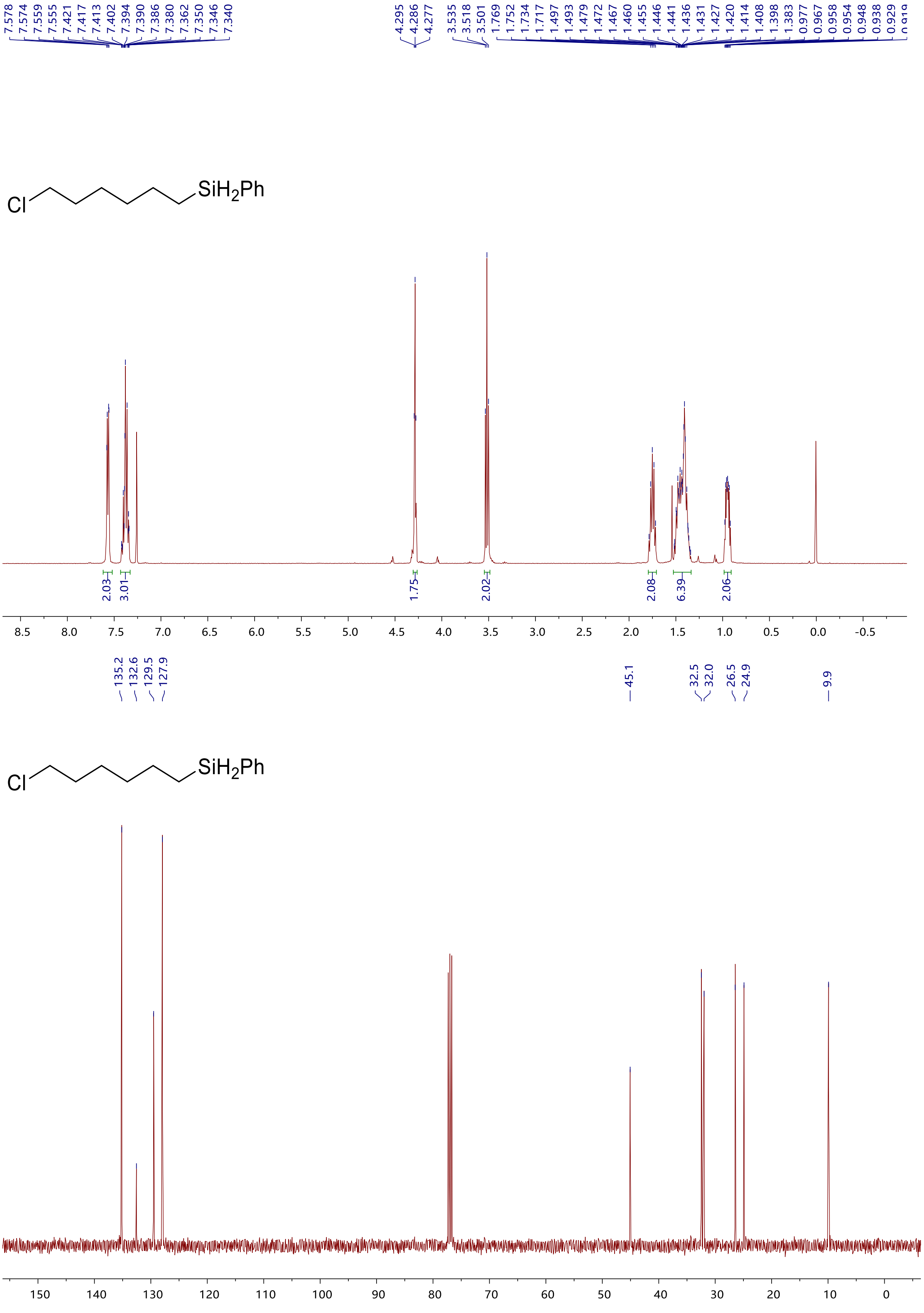

1-(6-(phenylsilyl)hexyl)piperidine (8ia)

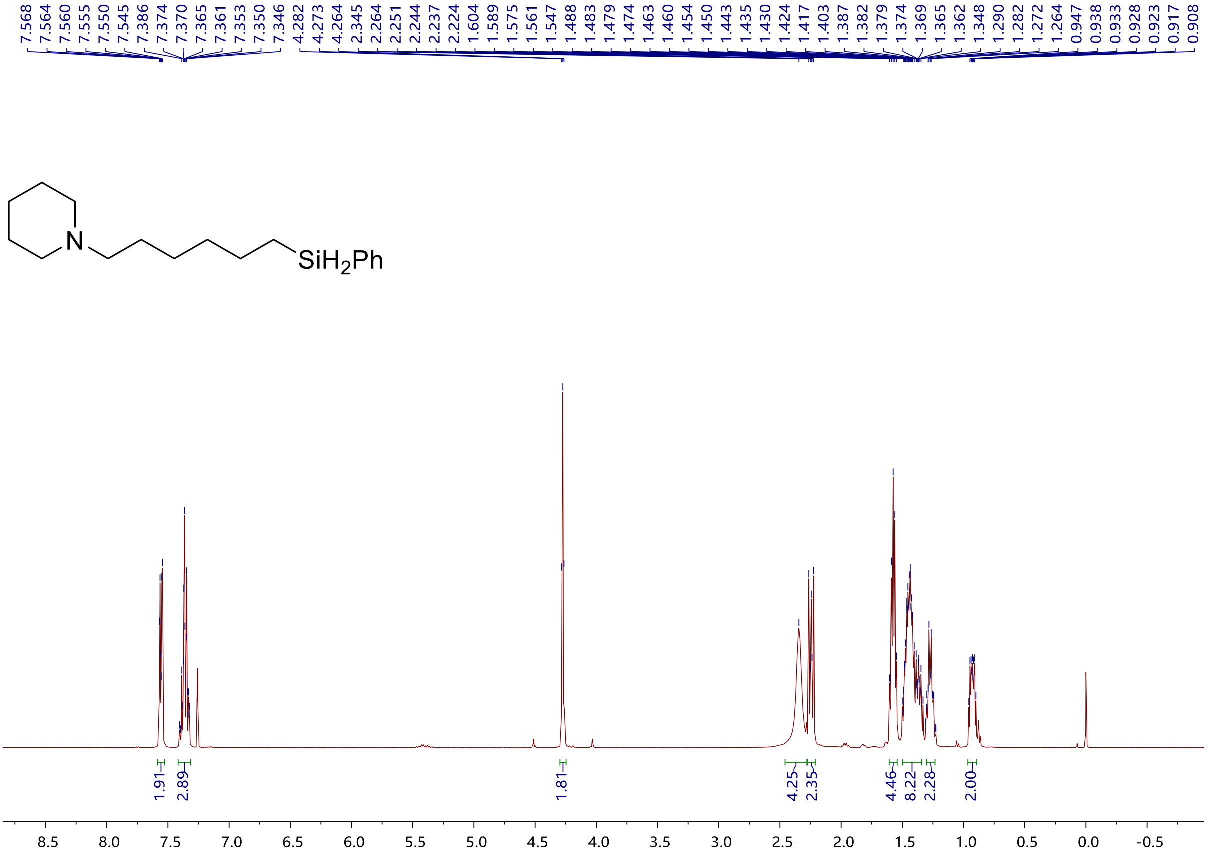

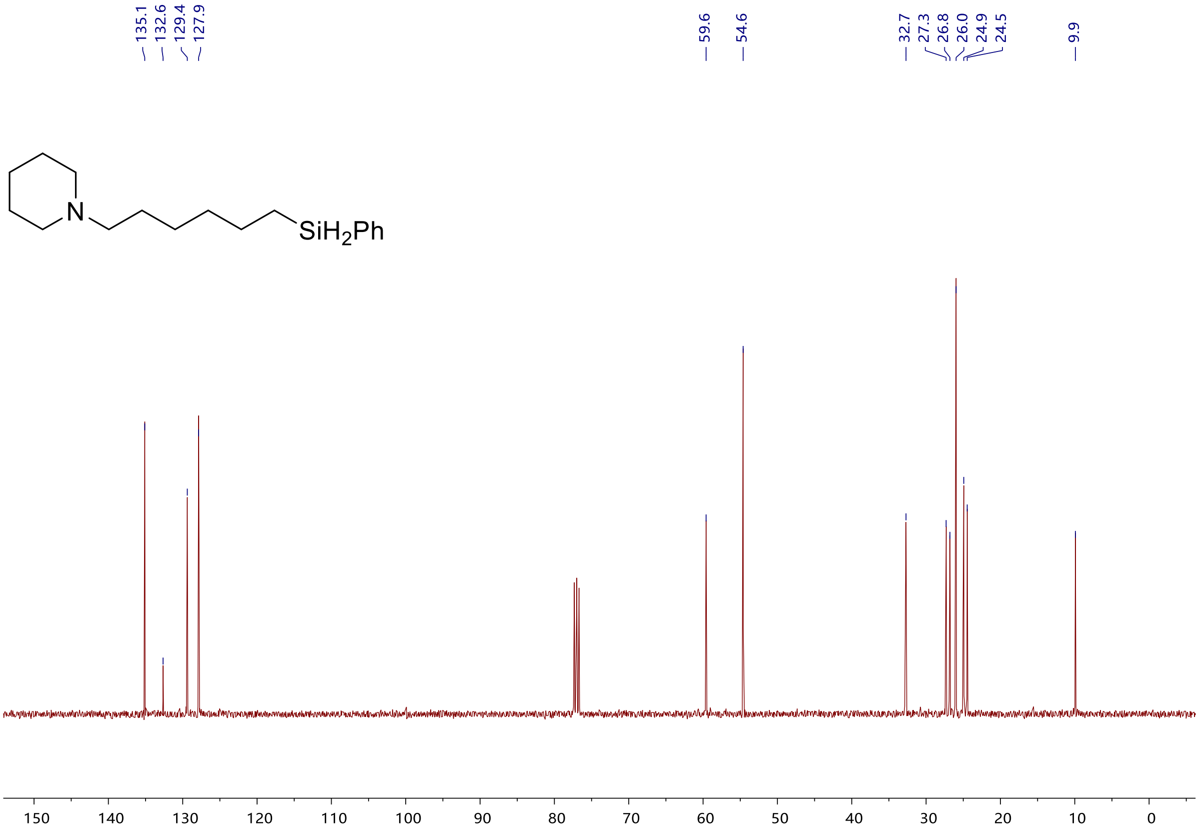

4-(6-(phenylsilyl)hexyl)morpholine (8ja)

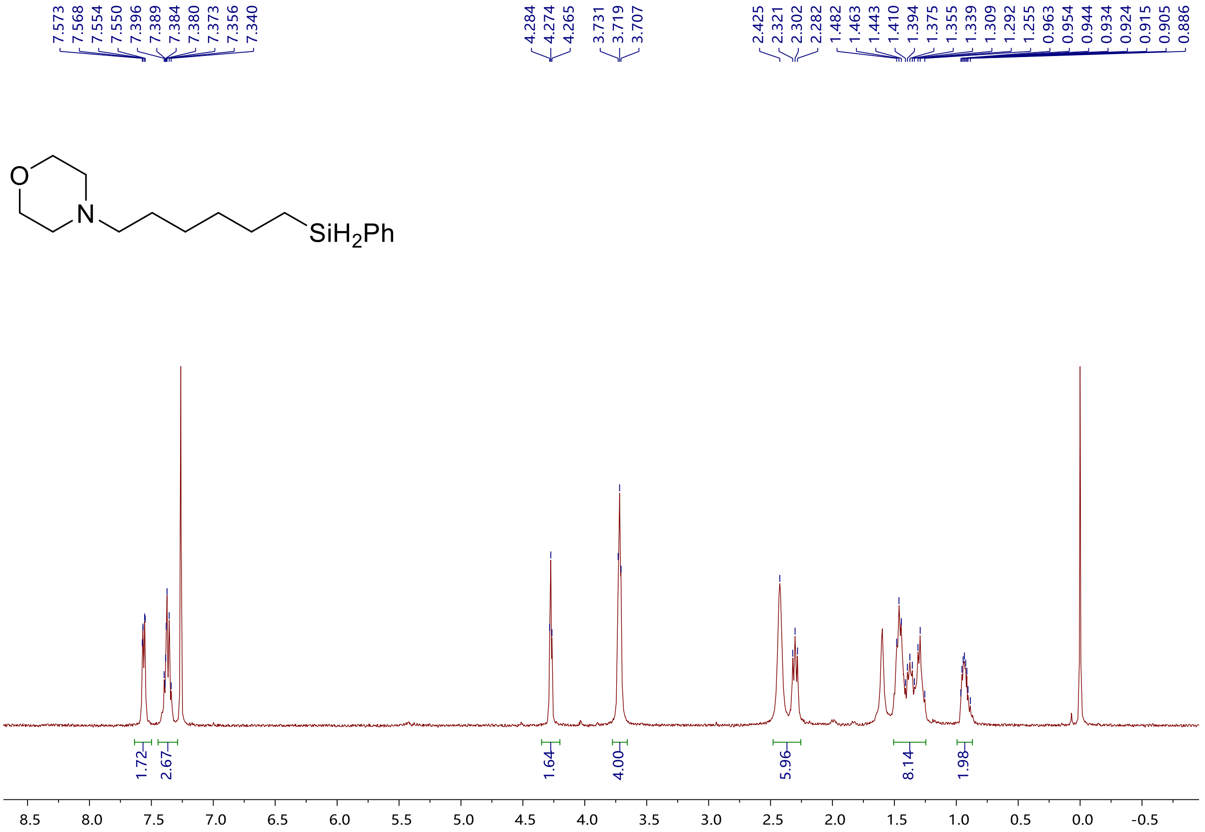

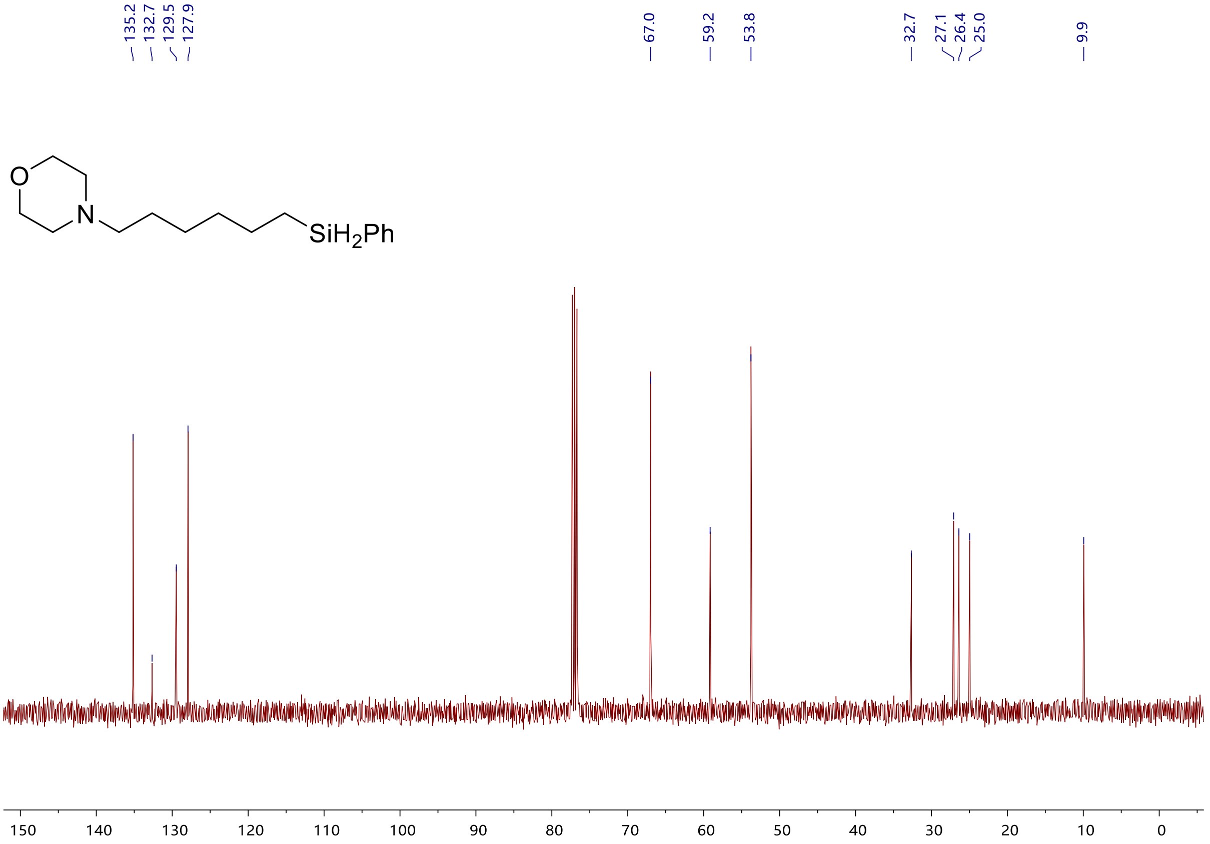

N,N-dimethyl-3-(phenylsilyl)propan-1-amine (8ka)

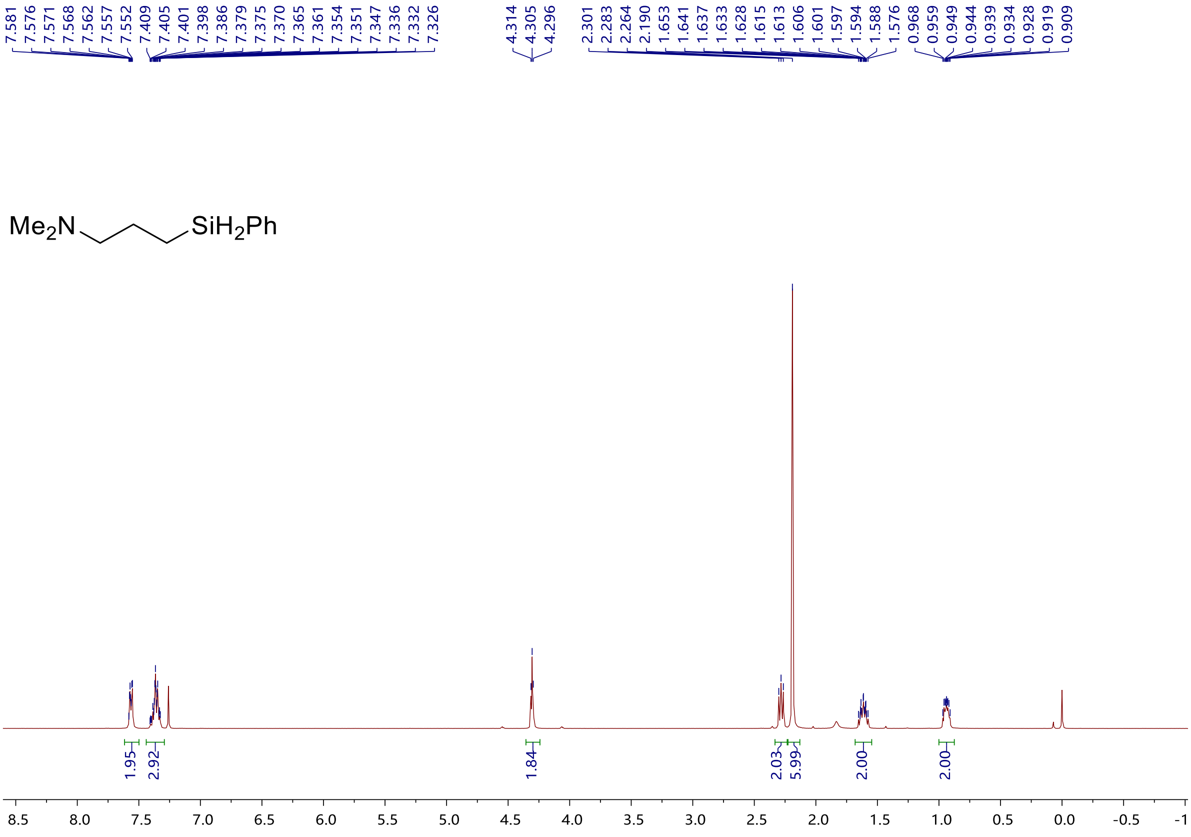

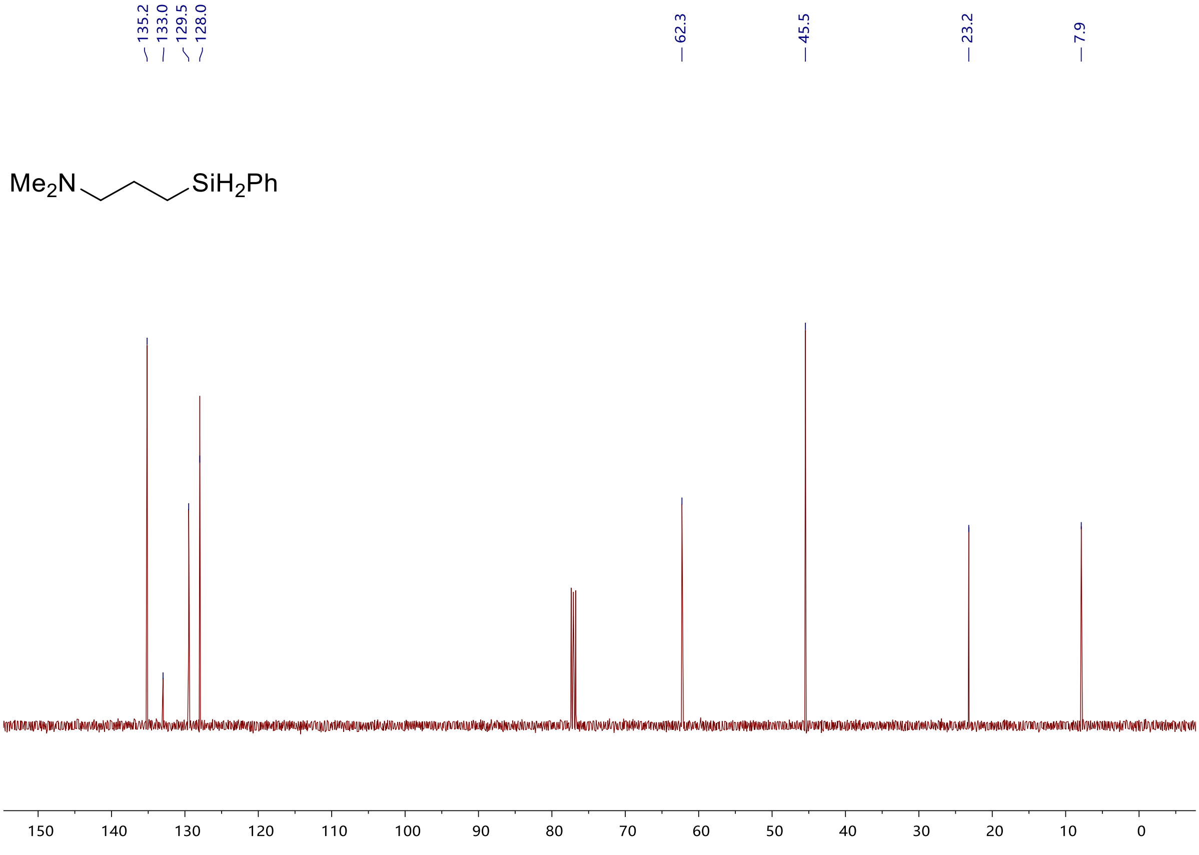

(2-phenoxyethyl)(phenyl)silane(8la)

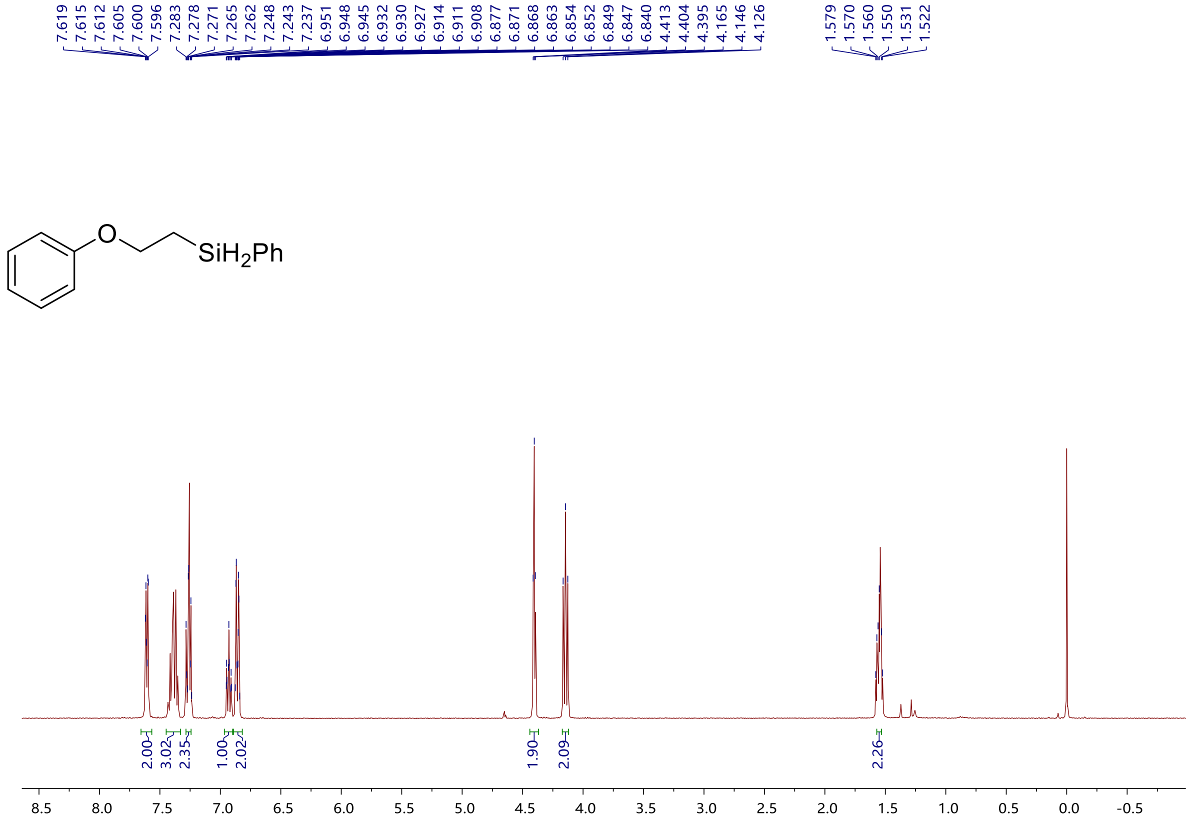

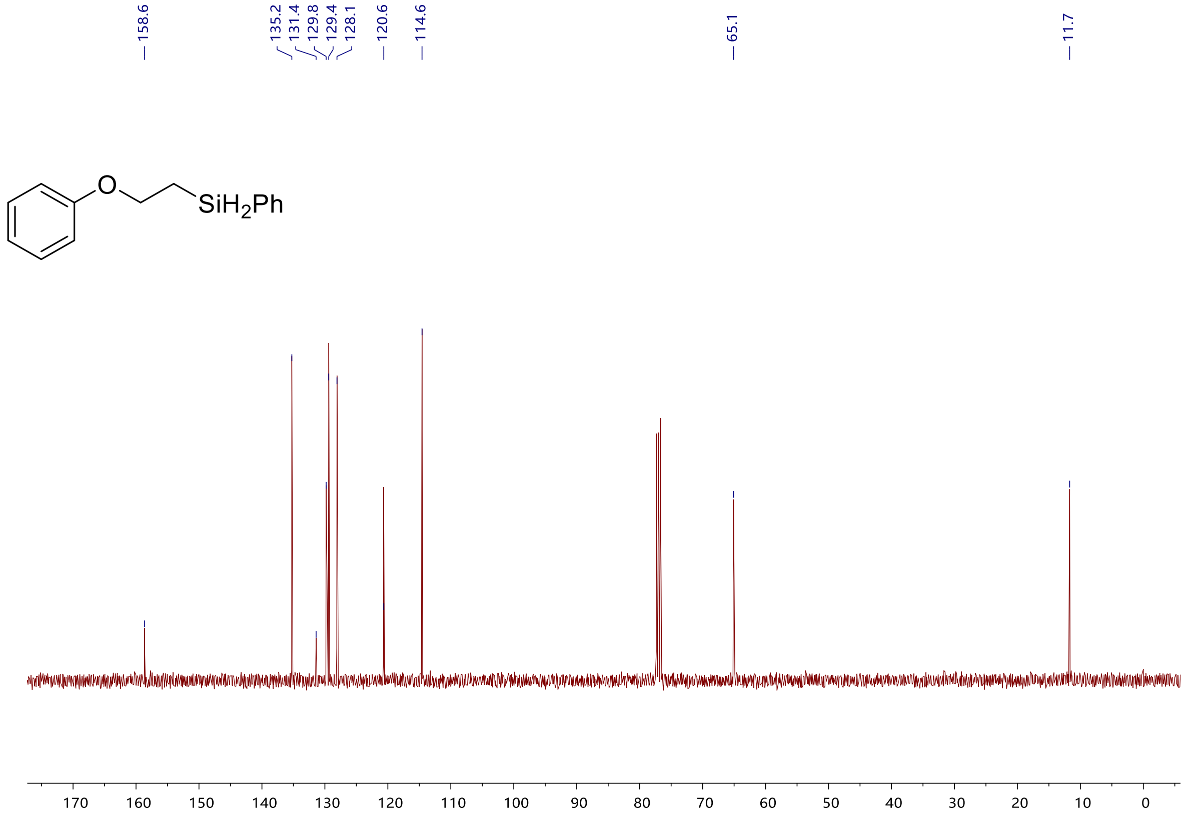

(2-butoxyethyl)(phenyl)silane (8ma)

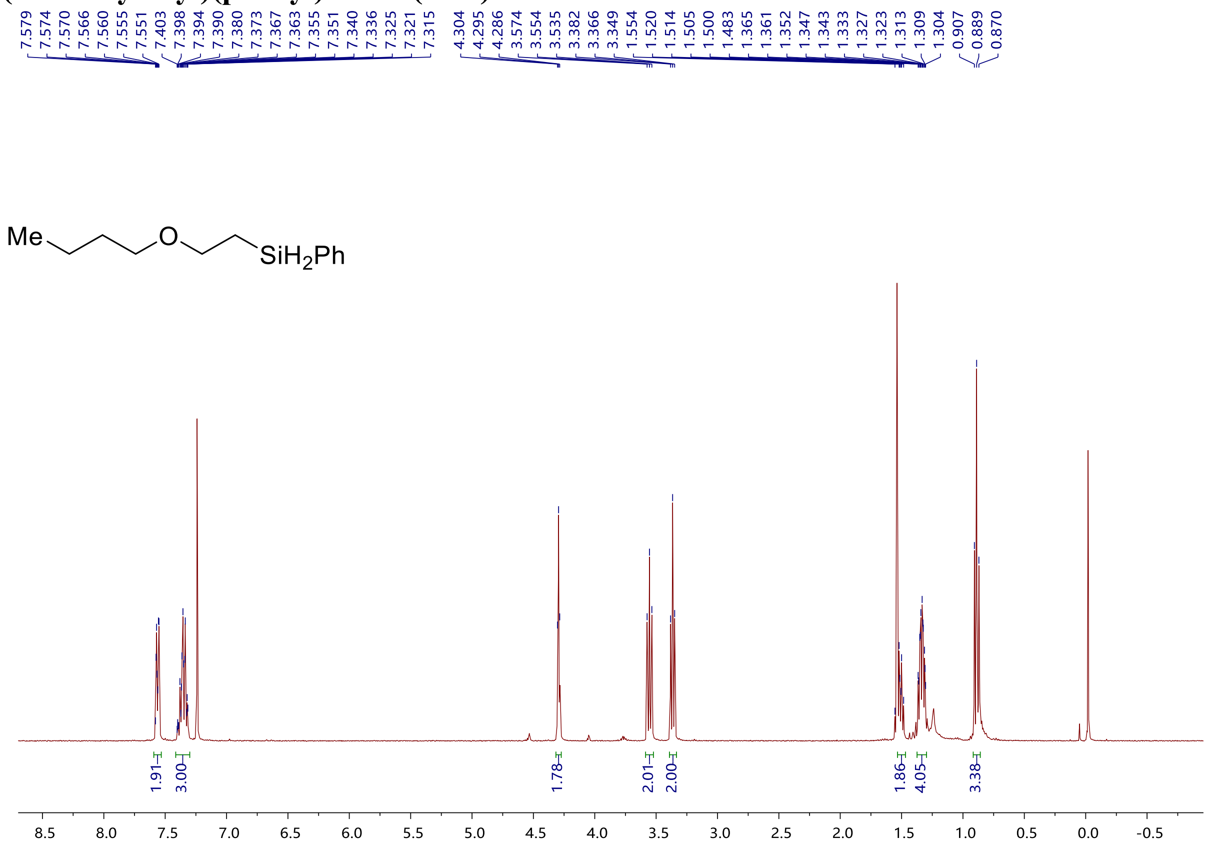

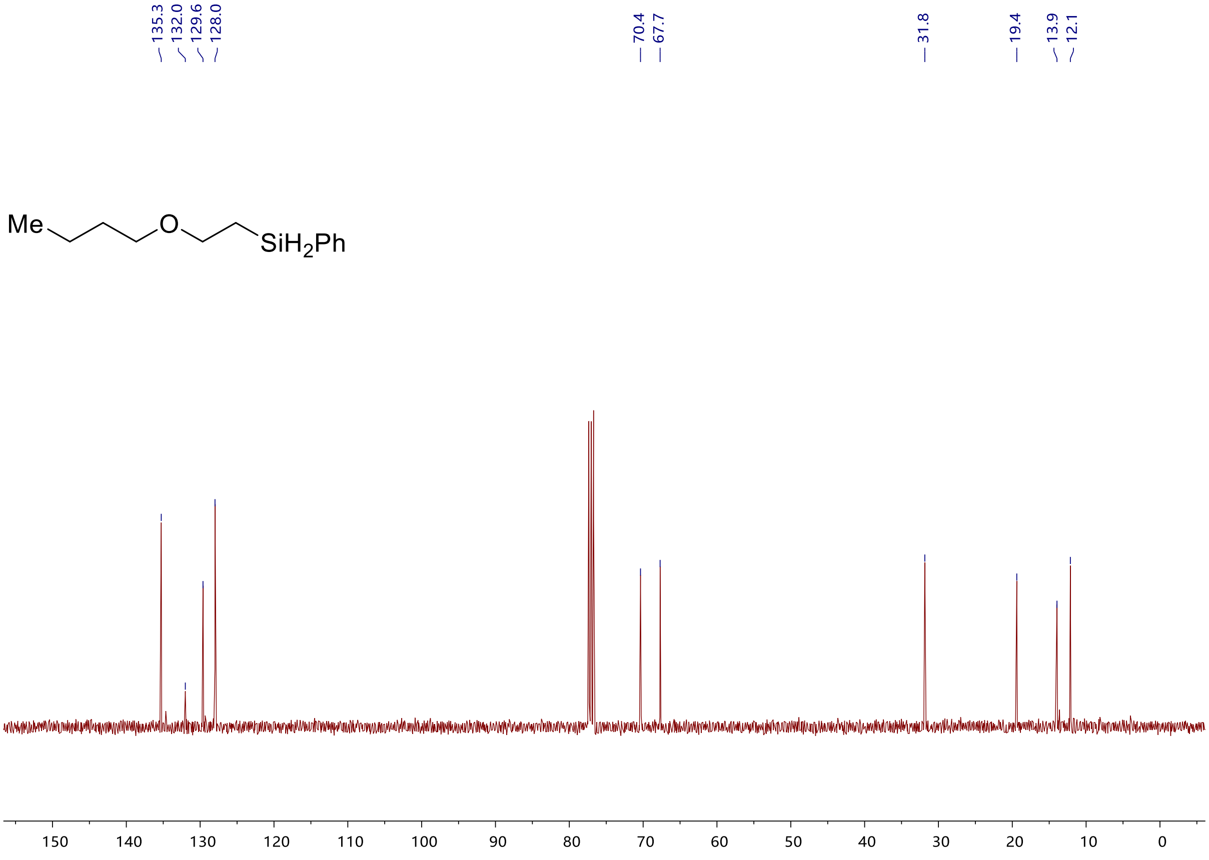

trimethyl(2-(phenylsilyl)ethyl)silane (8na)

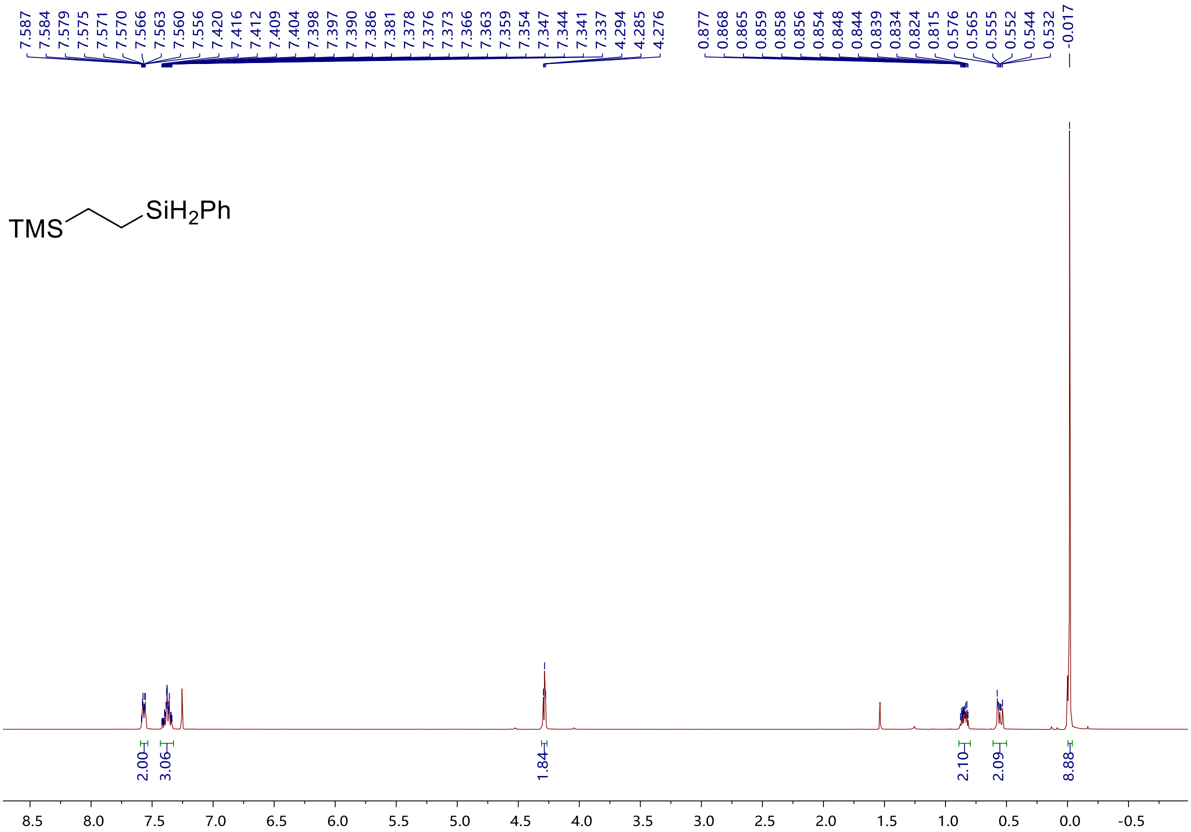

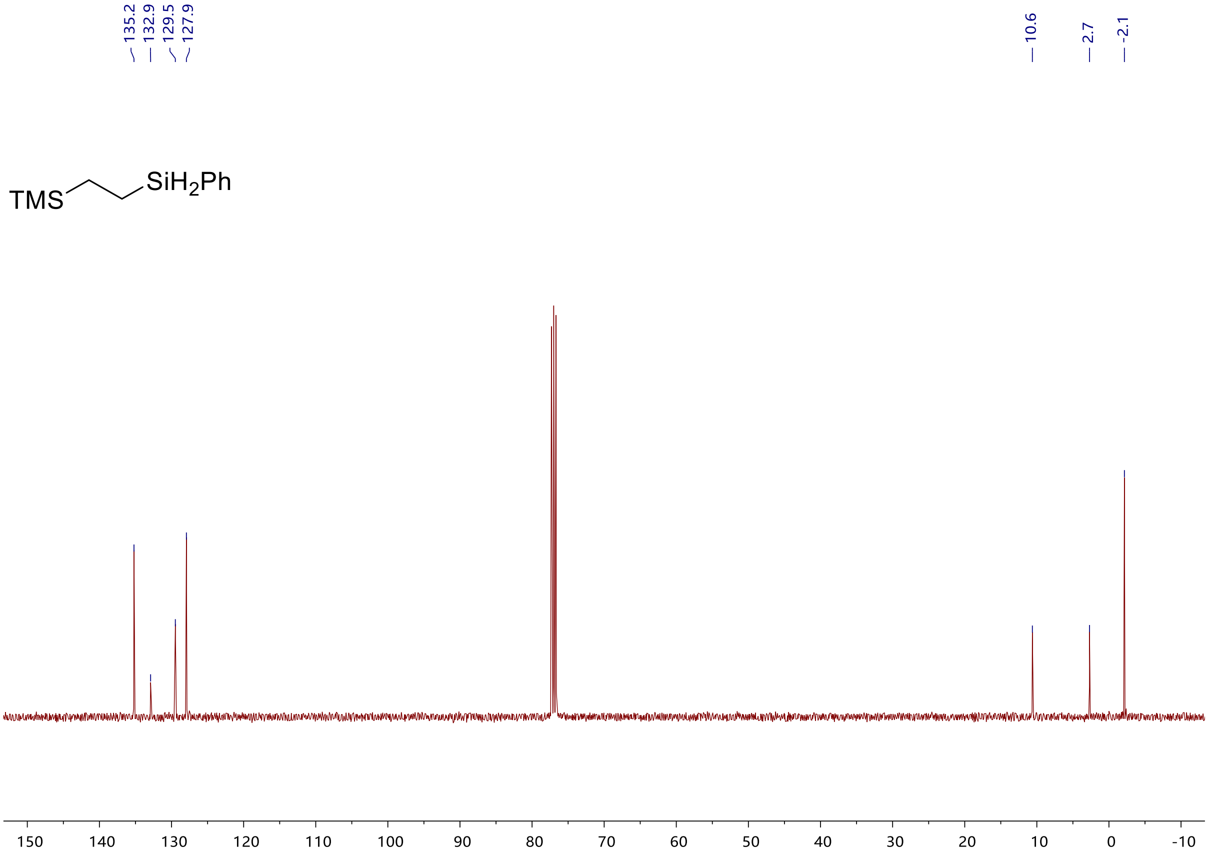

dimethyl(phenyl)(2-(phenylsilyl)ethyl)silane (8oa)

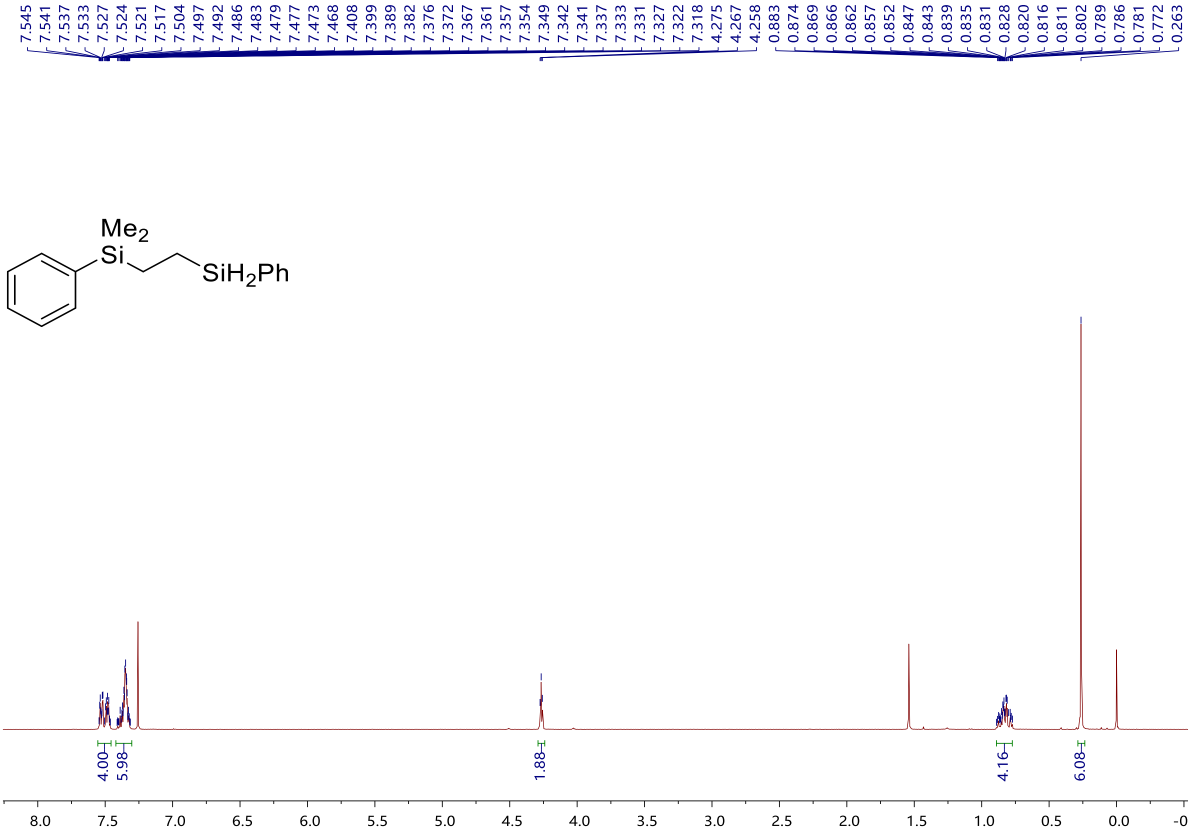

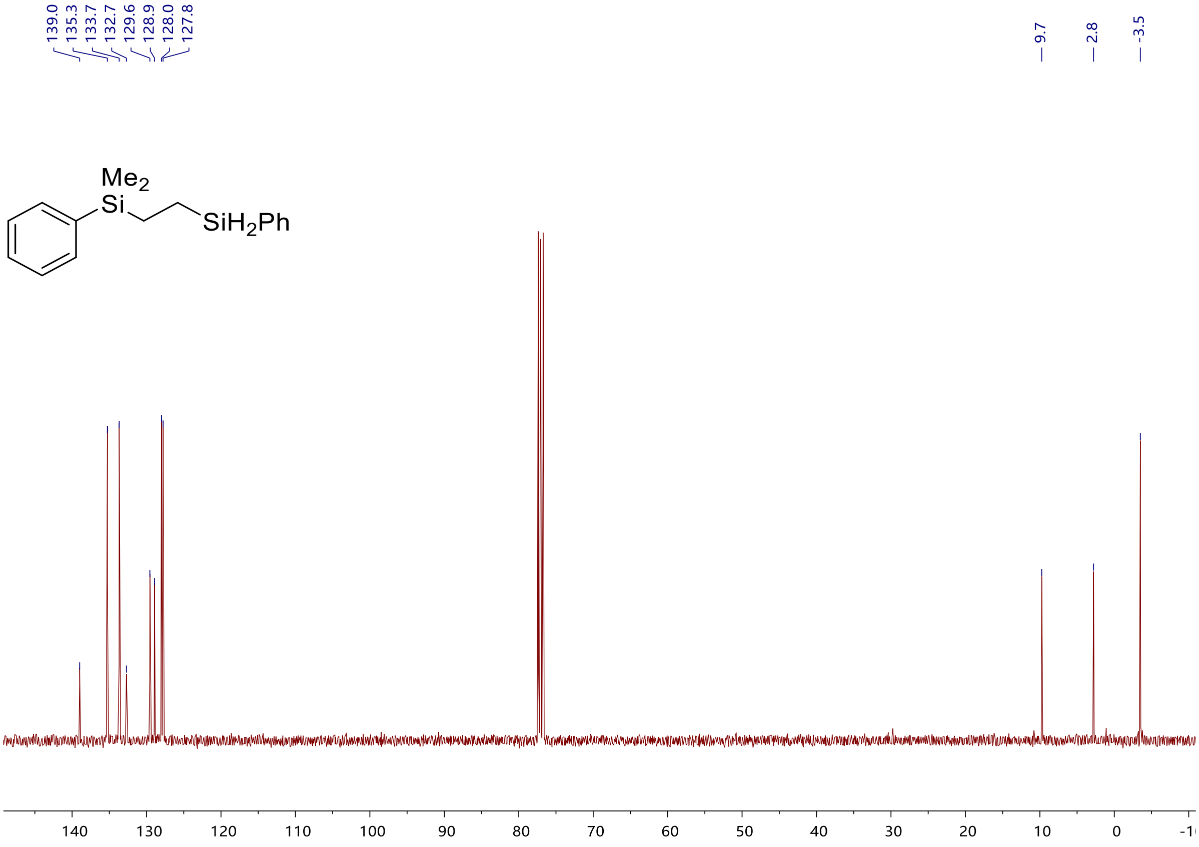

(*E*)-dimethoxy(phenyl)(4-phenylbut-3-en-1-yl)silane (9)

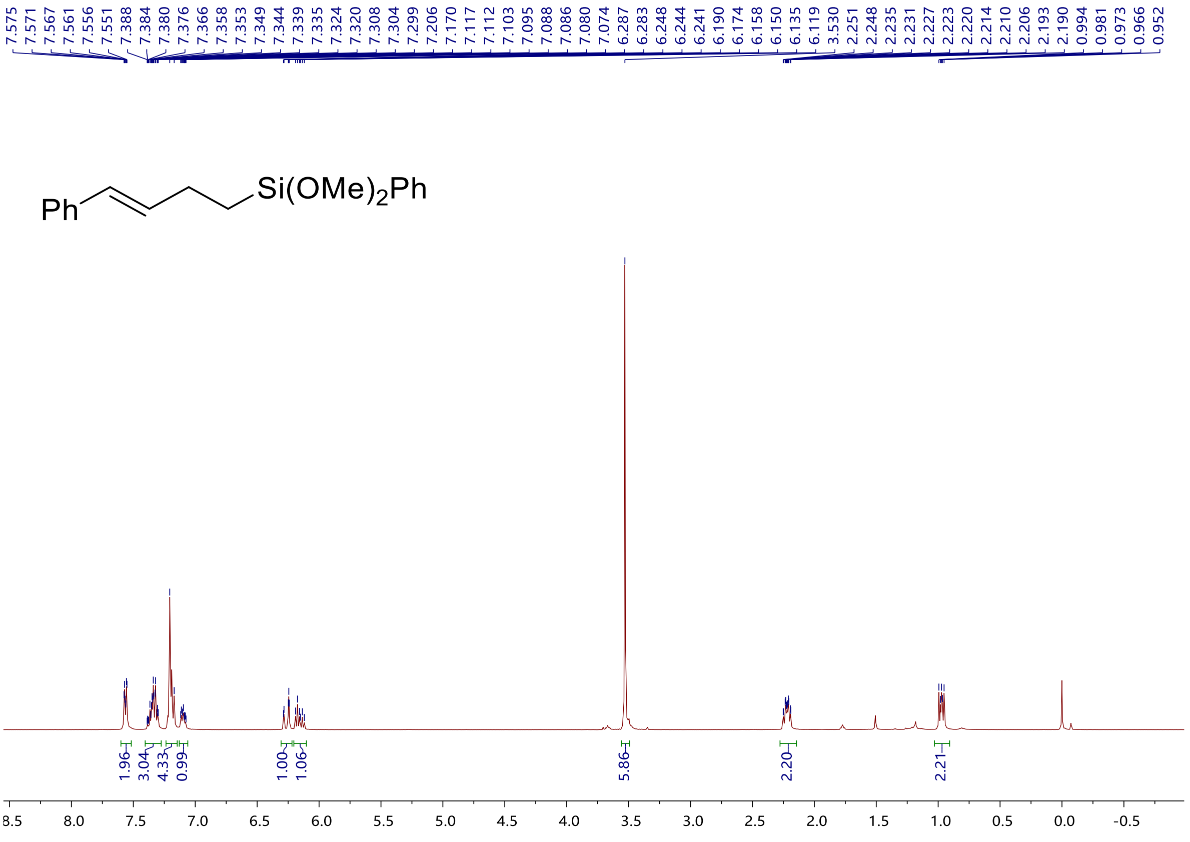

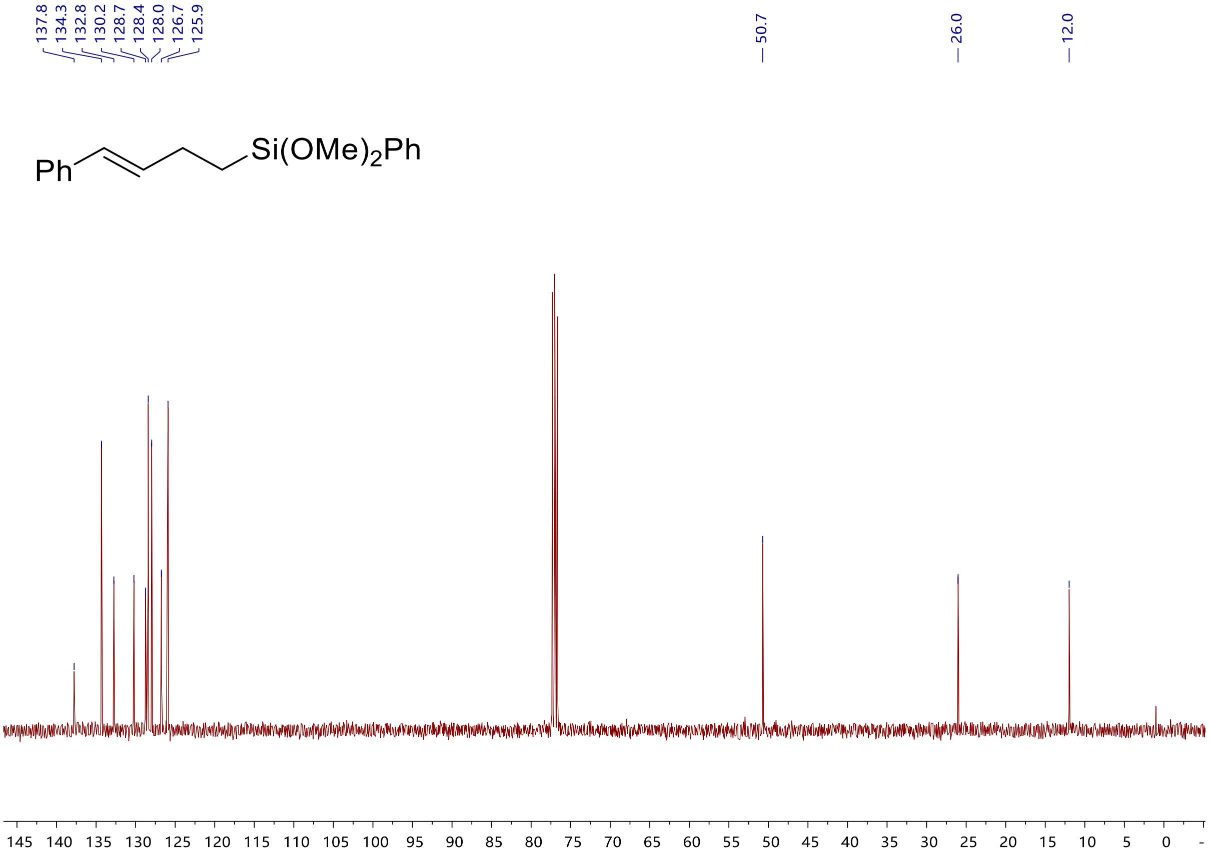

(*E*)-difluoro(phenyl)(4-phenylbut-3-en-1-yl)silane (10)

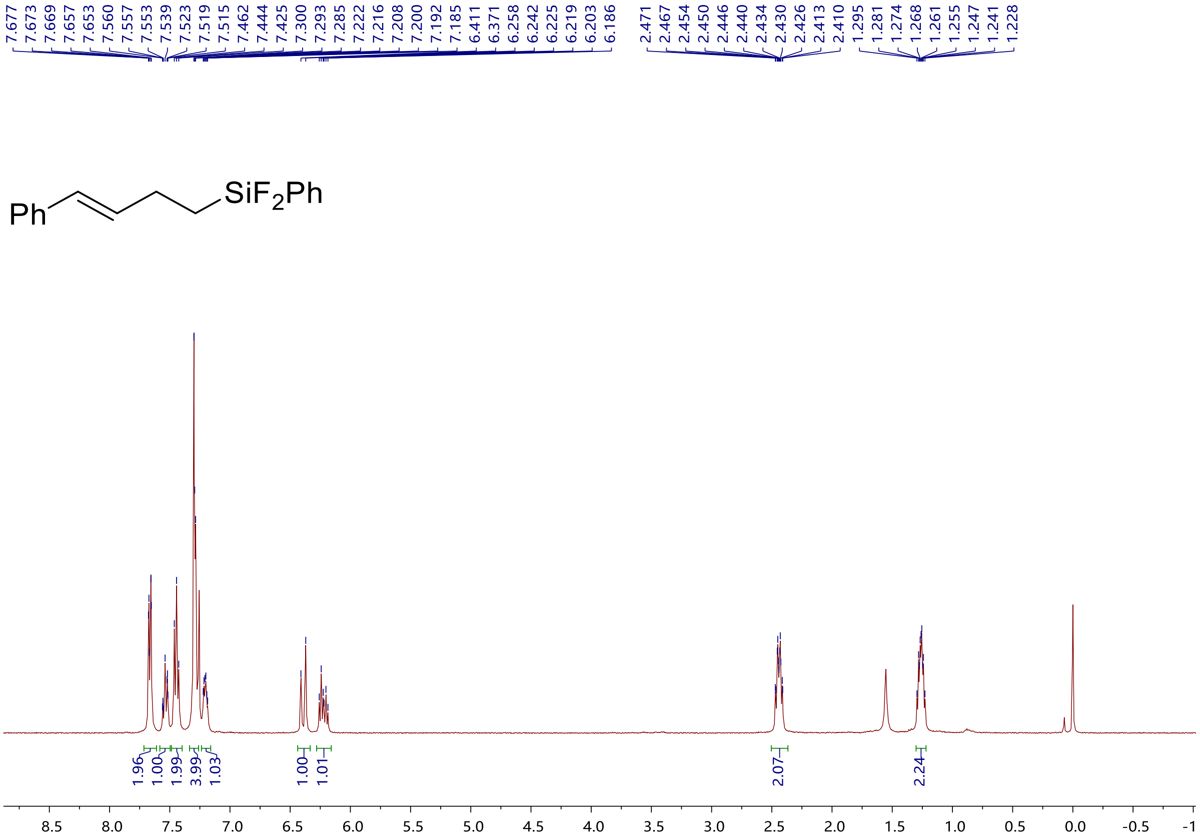

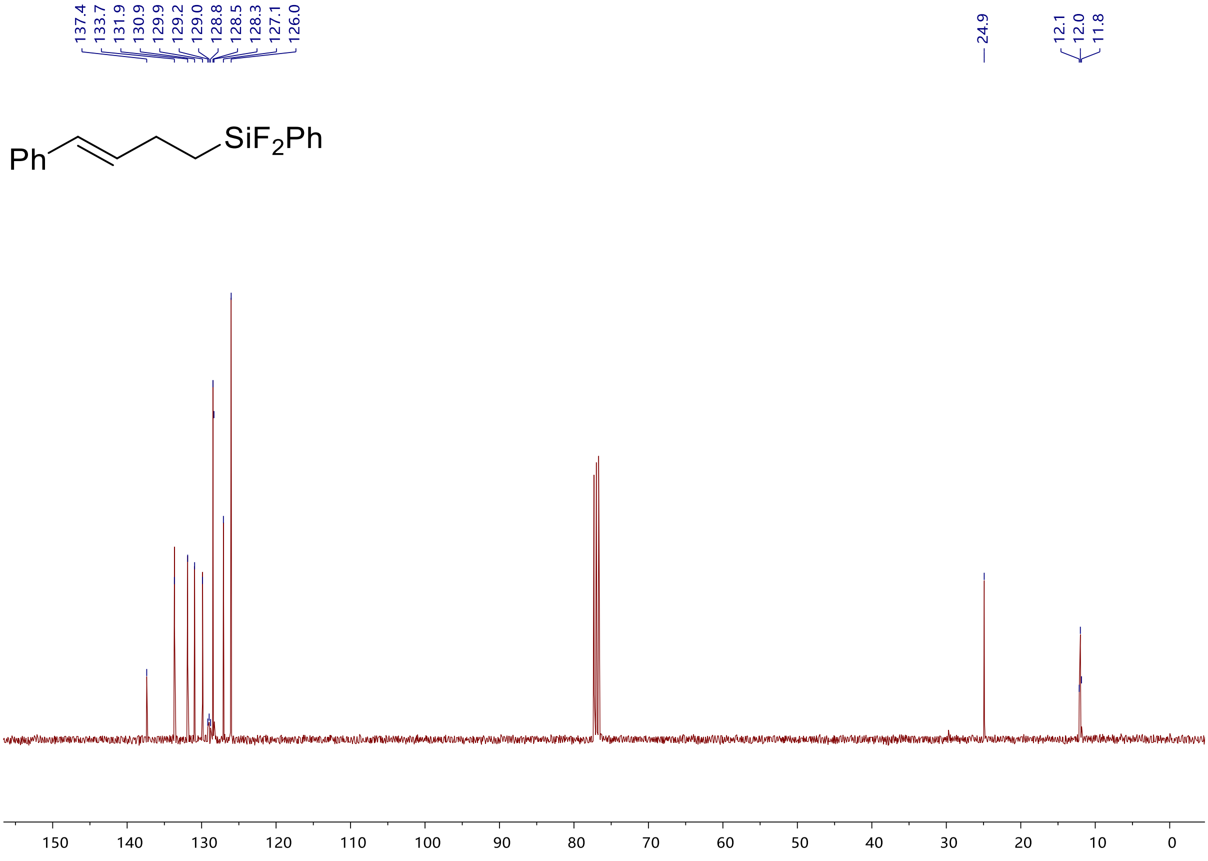

(*E*)-4-phenylbut-3-en-1-ol (11)

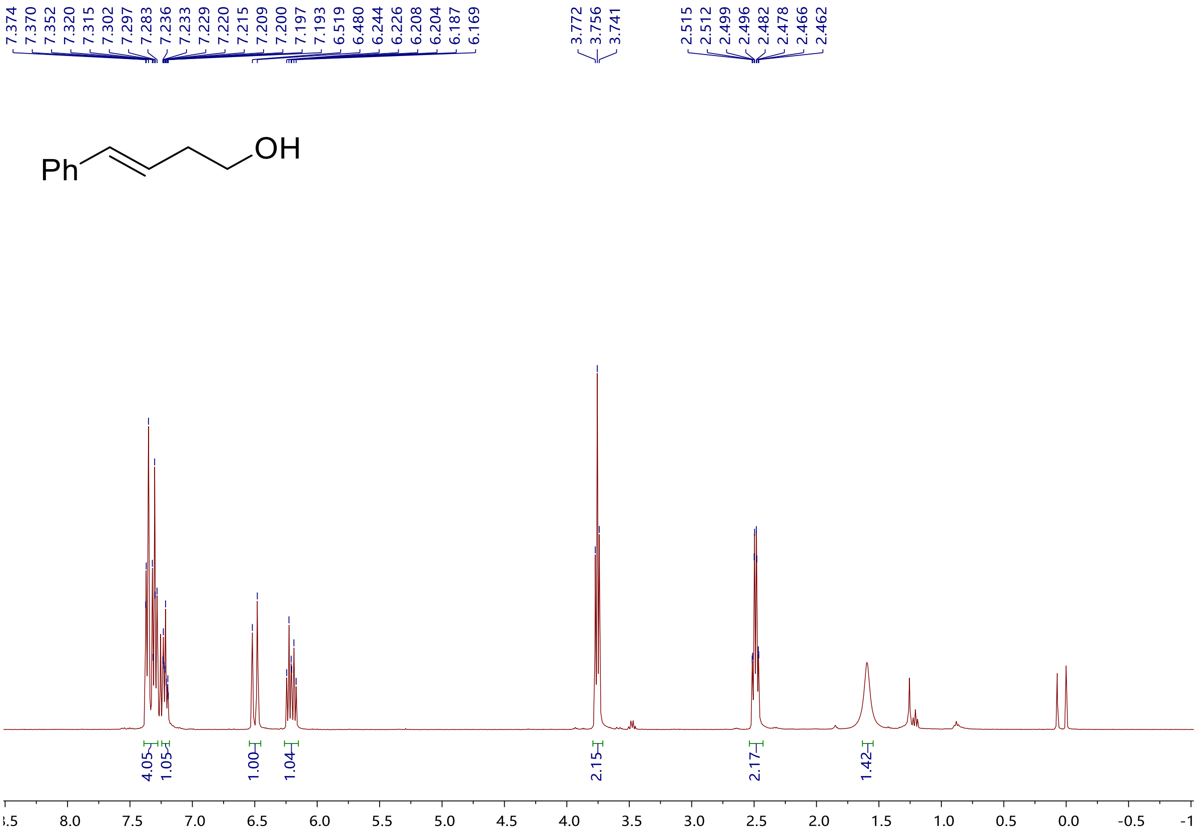

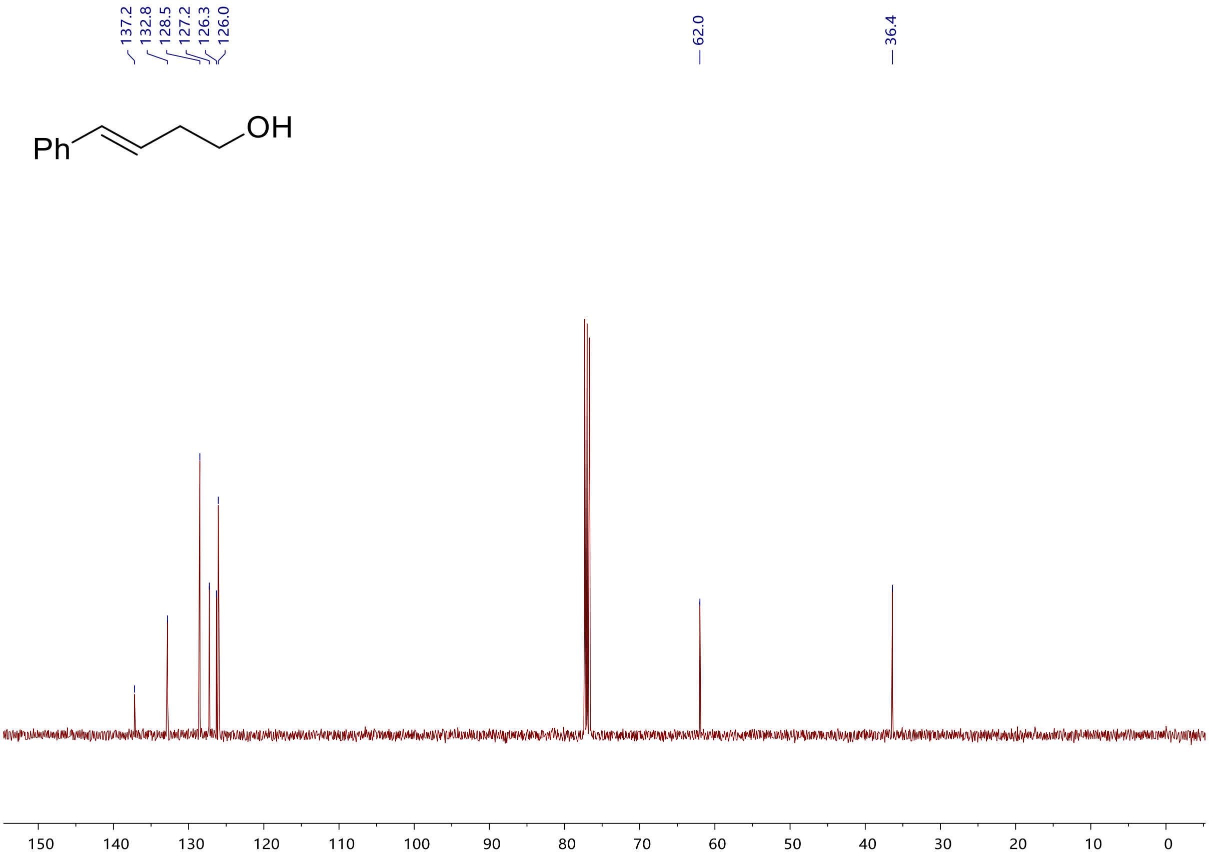

phenyl((*E*)-4-phenylbut-3-en-1-yl)((*E*)-styryl)silane (12)

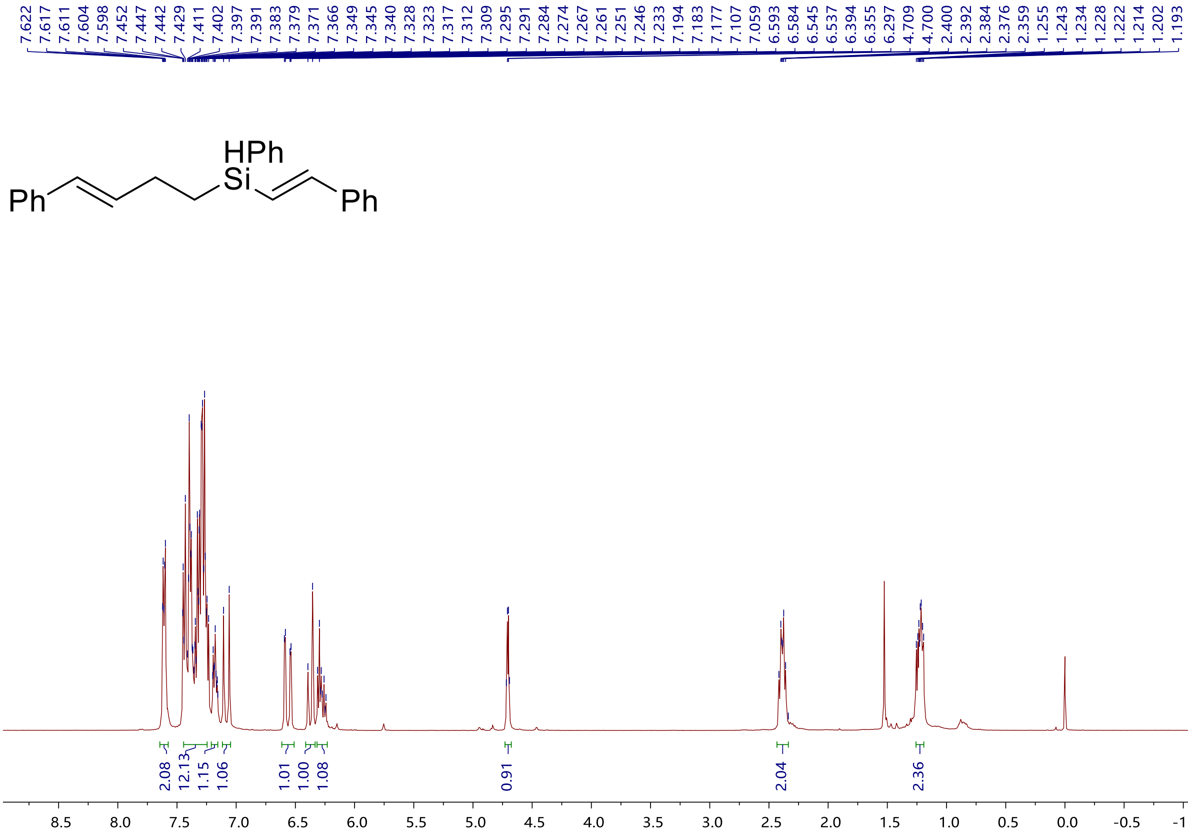

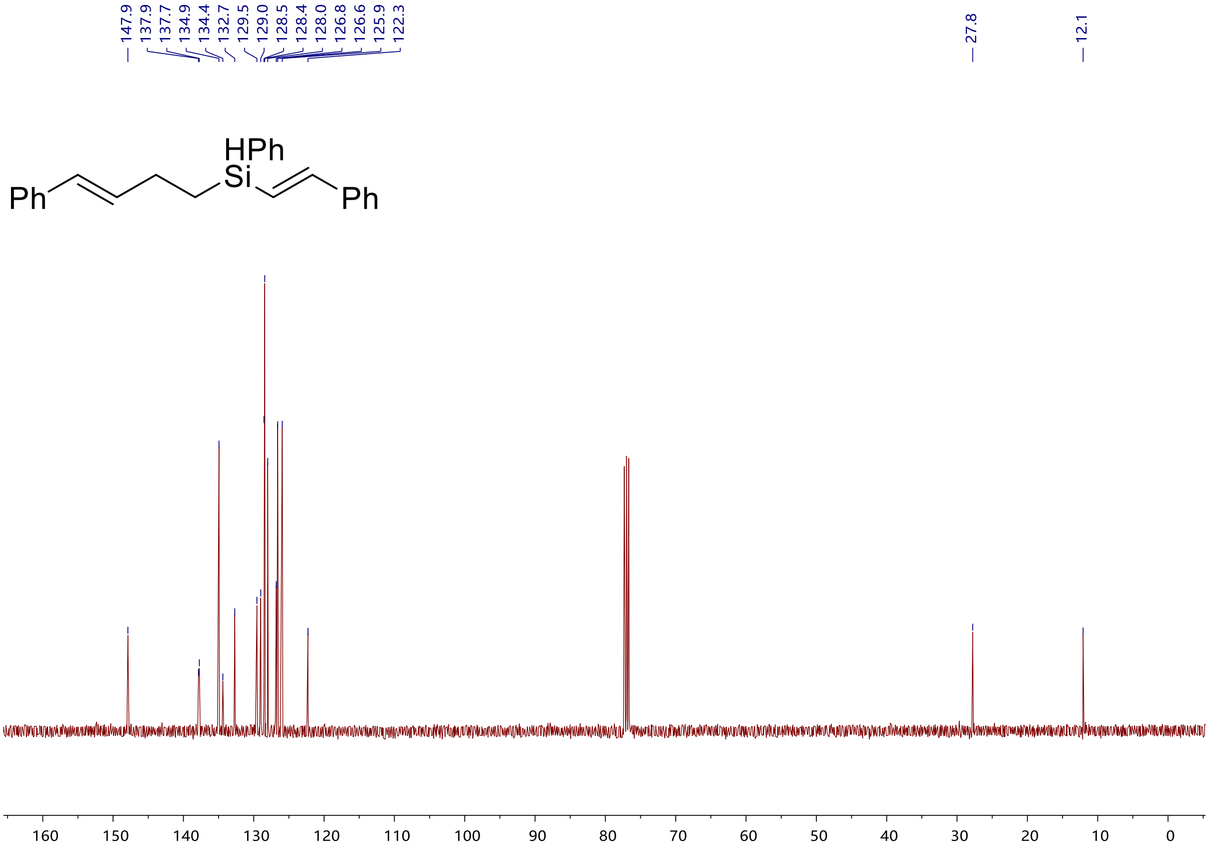

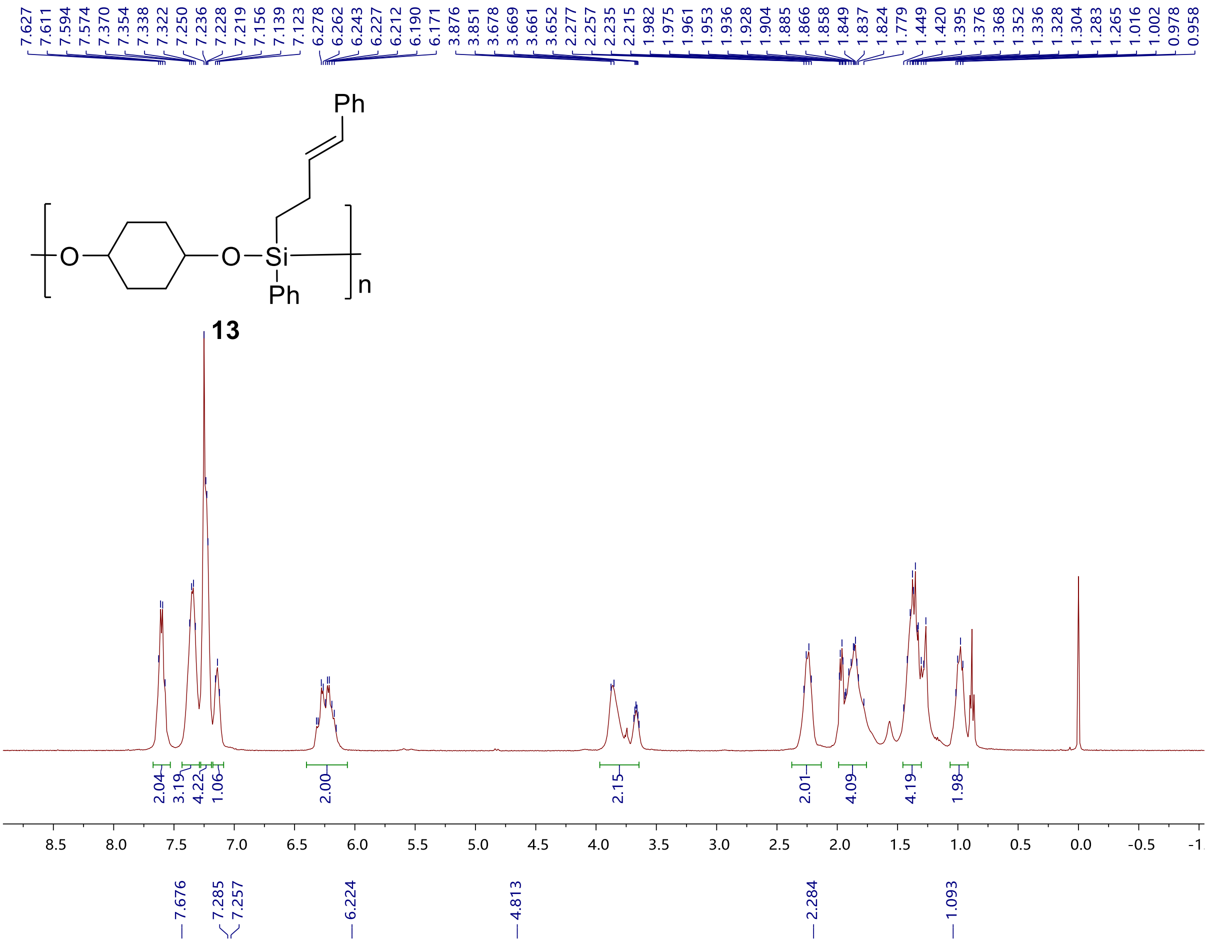

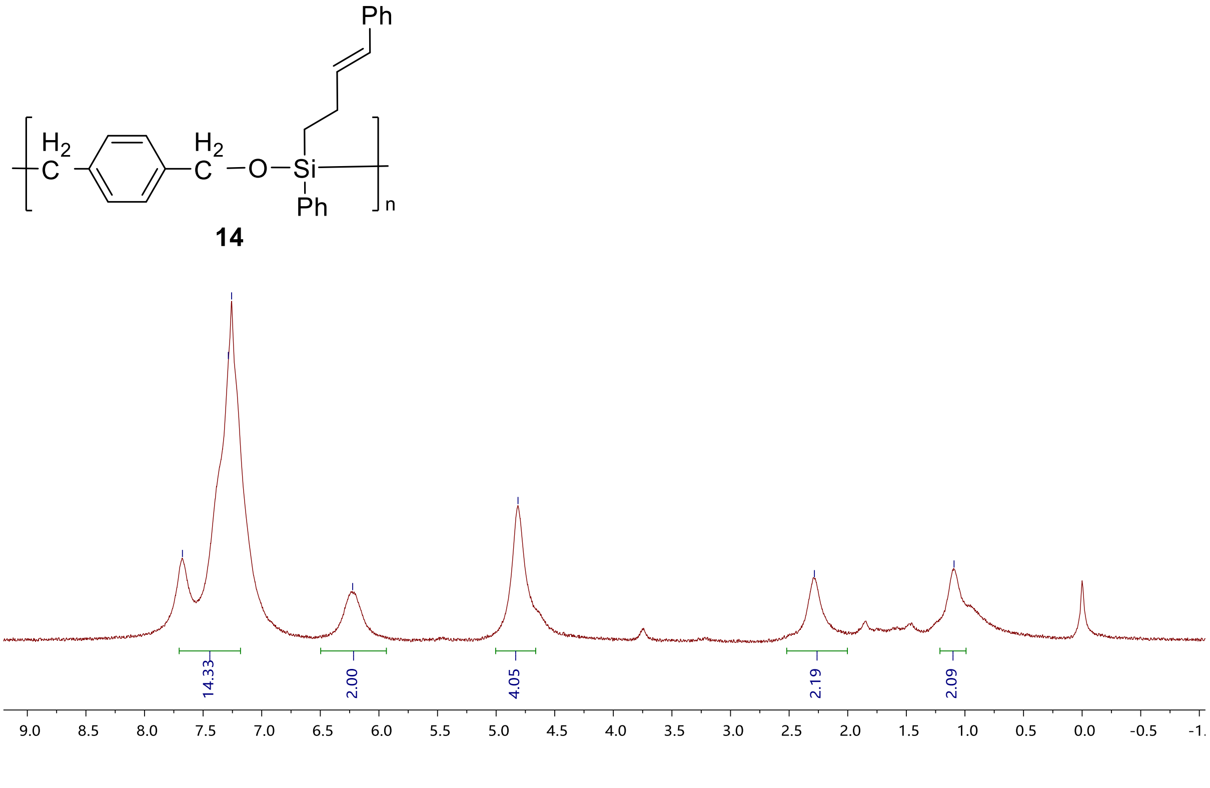

Phenyl(4-phenylbut-3-en-1-yl)silane (3aa')

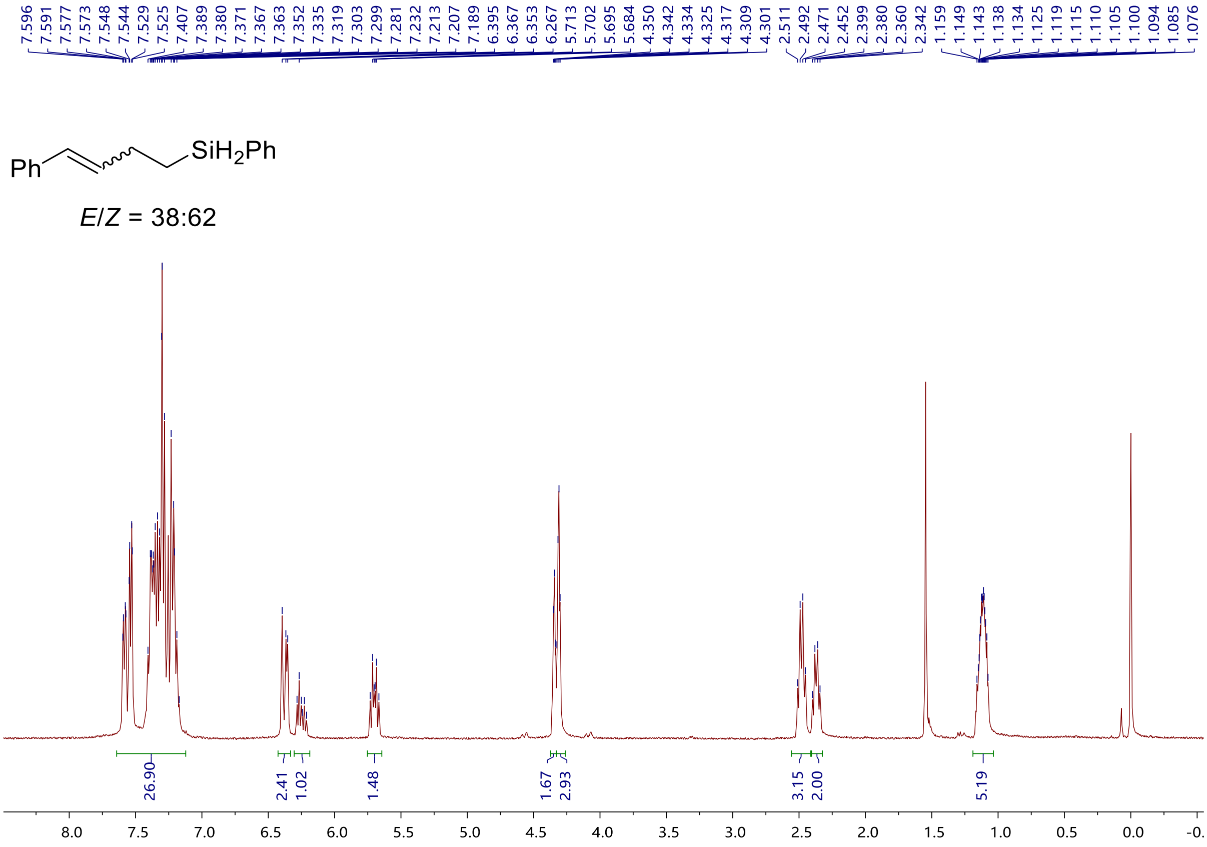

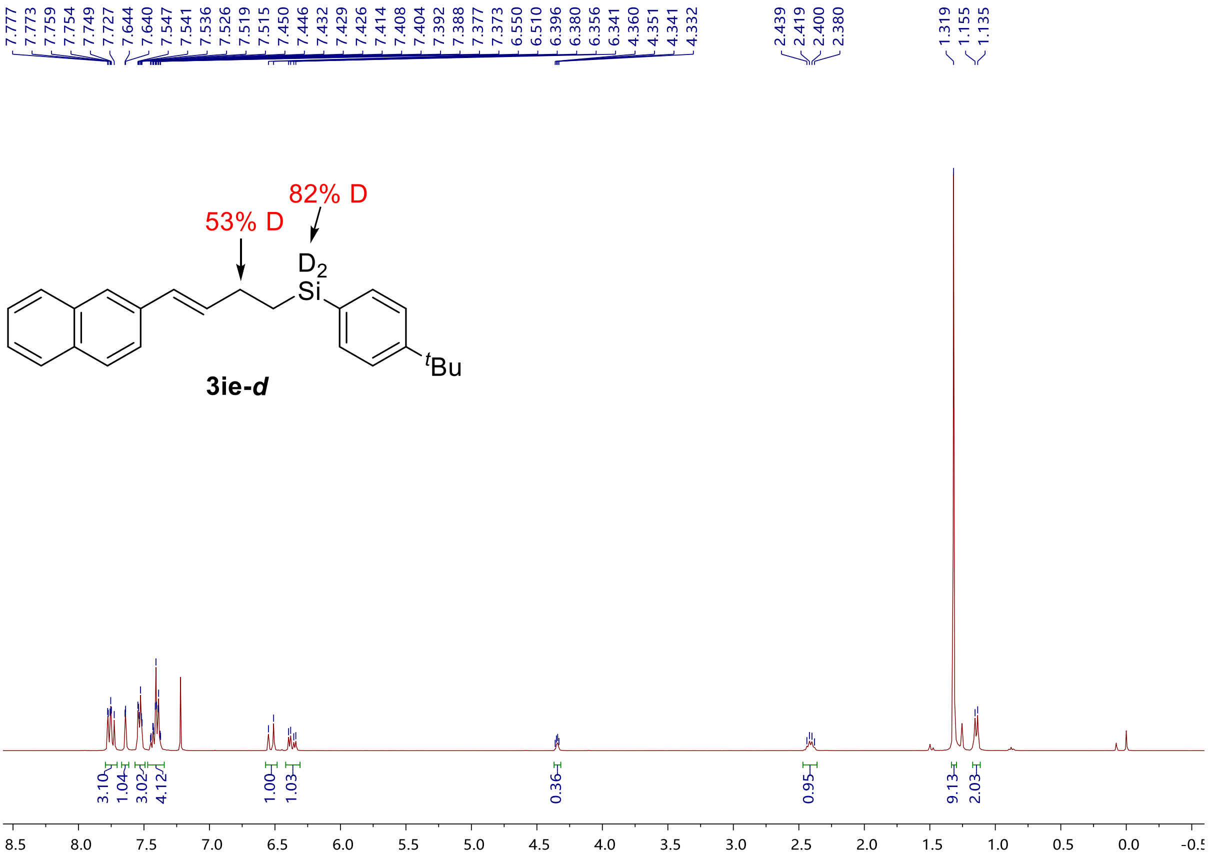
7.777
7.773
7.759
7.754
7.749
7.727
7.644
7.640
7.547
7.541
7.536
7.526
7.519
7.515
7.450
7.446
7.432
7.429
7.426
7.414
7.408
7.404
7.392
7.388
7.377
7.373
6.550
6.510
6.396
6.380
6.356
6.341
4.360
4.351
4.341
4.332
2.439
2.419
2.400
2.380
1.319
1.155
1.135
82% D
53% D
D2
Si
tBu
3ie-d
3.10
1.04
3.02
4.12
1.00
1.03
0.36
0.95
9.13
2.03
8.5
8.0
7.5
7.0
6.5
6.0
5.5
5.0
4.5
4.0
3.5
3.0
2.5
2.0
1.5
1.0
0.5
0.0

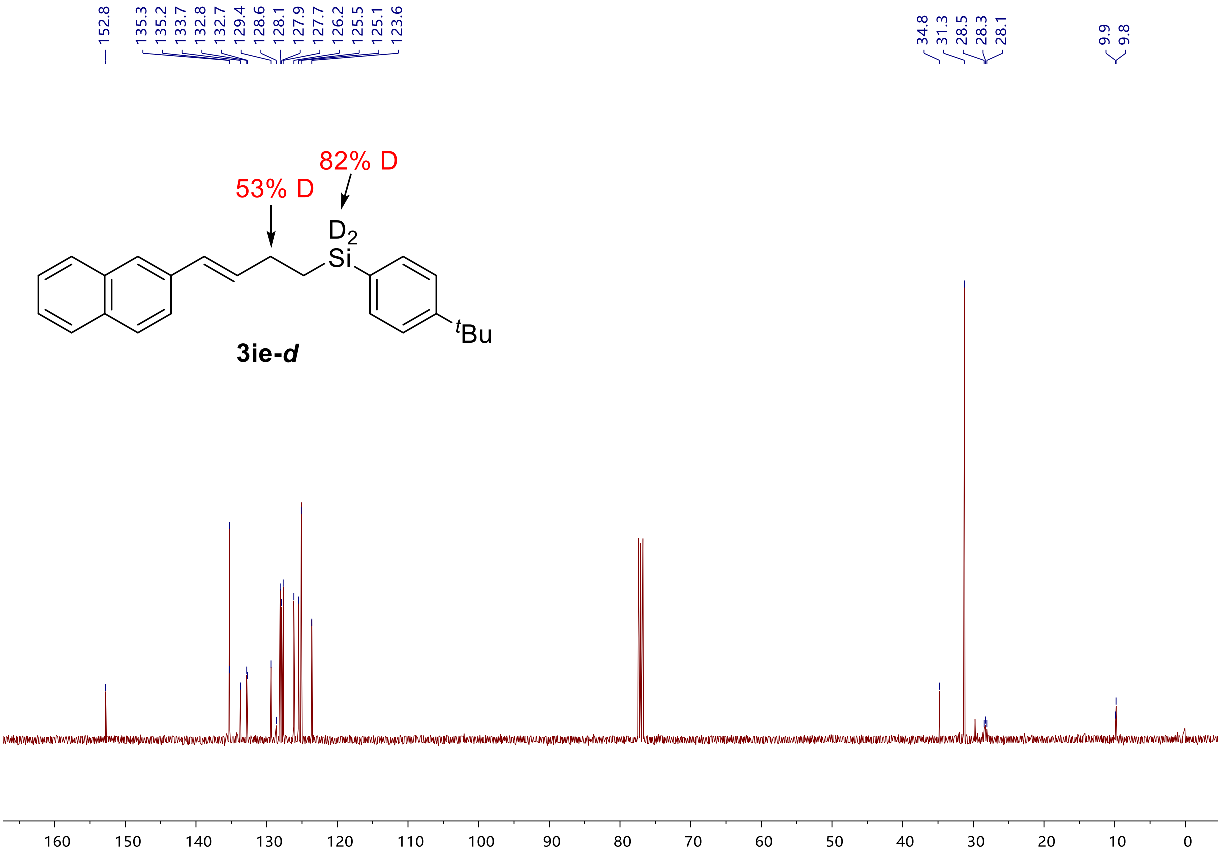
152.8
135.3
135.2
133.7
132.8
132.7
129.4
128.6
128.1
127.9
127.7
126.2
125.5
125.1
123.6
34.8
31.3
28.5
28.3
28.1
9.9
9.8
82% D
53% D
D2
Si
tBu
3ie-d
160
150
140
130
120
110
100
90
80
70
60
50
40
30
20
10
0

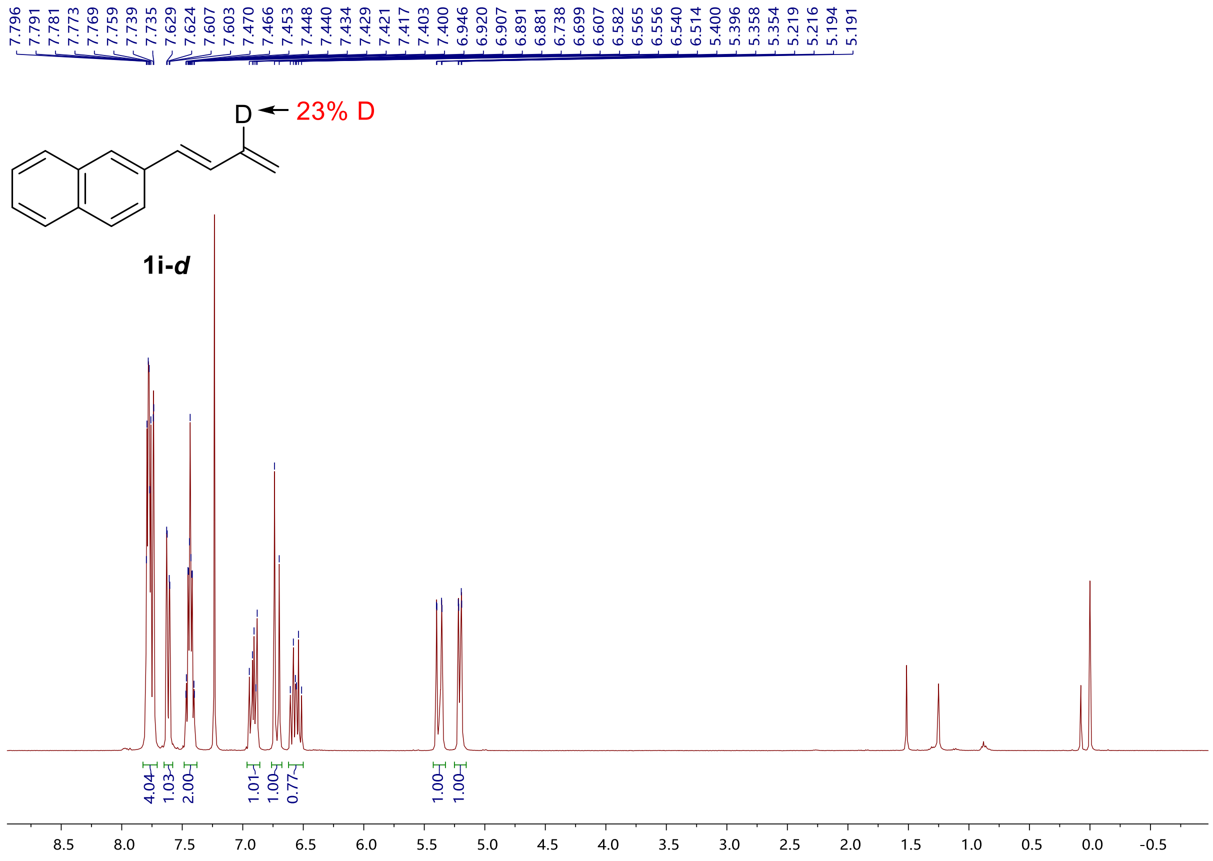
23% D
D
1i-*d*

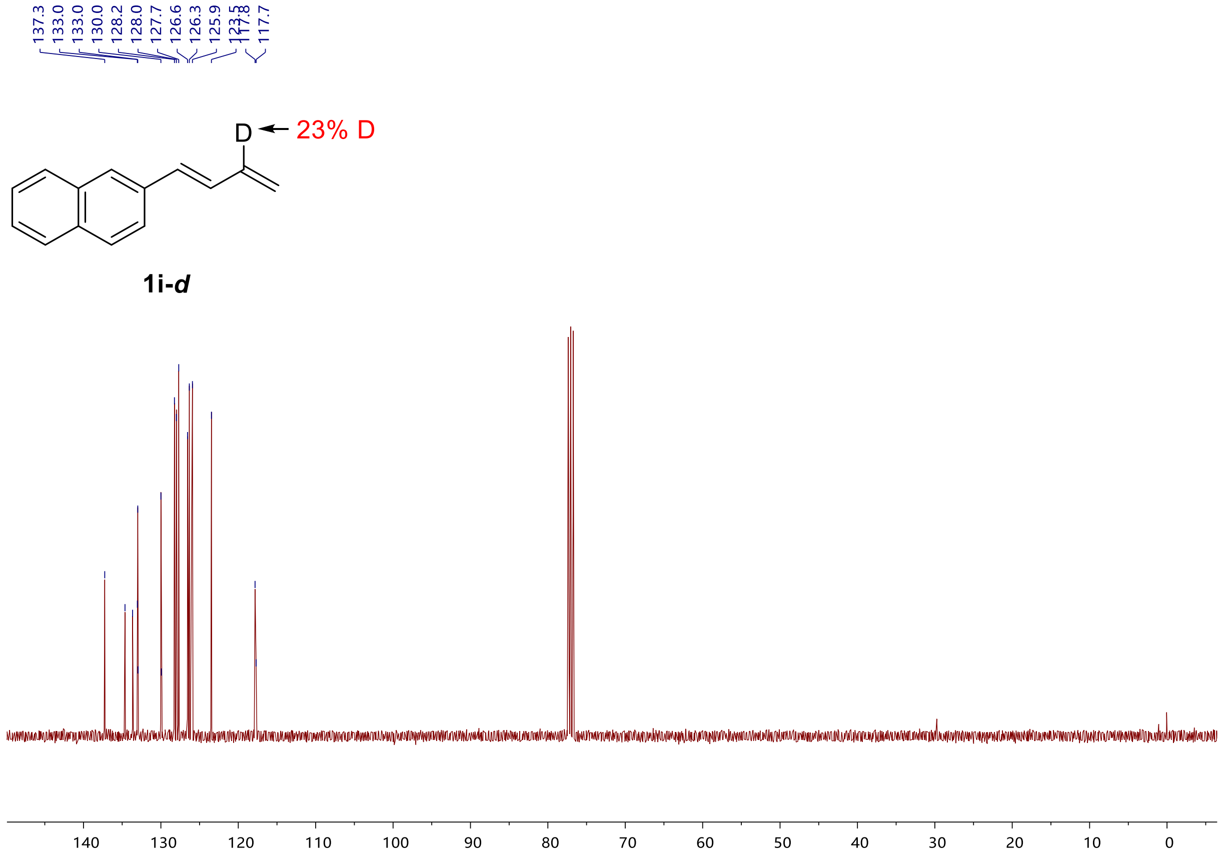
23% D
D
1i-*d*

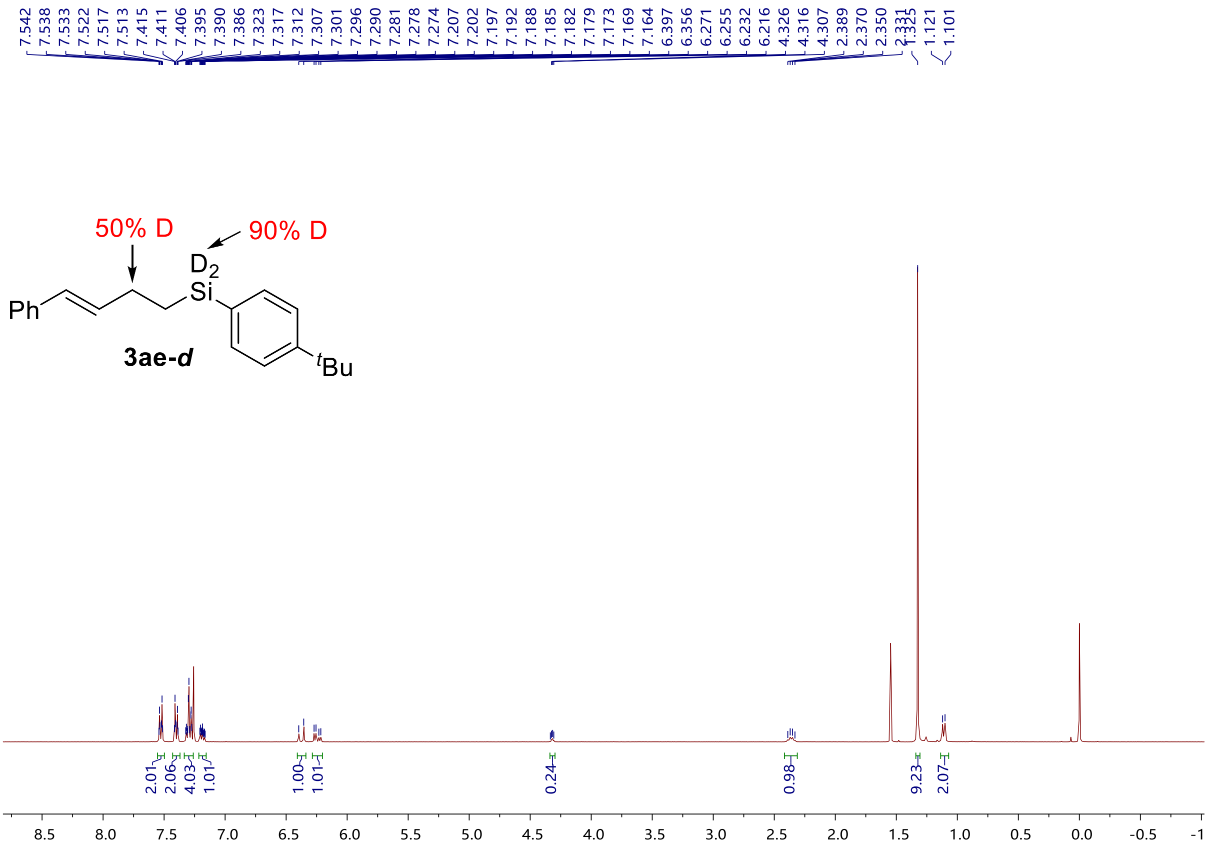

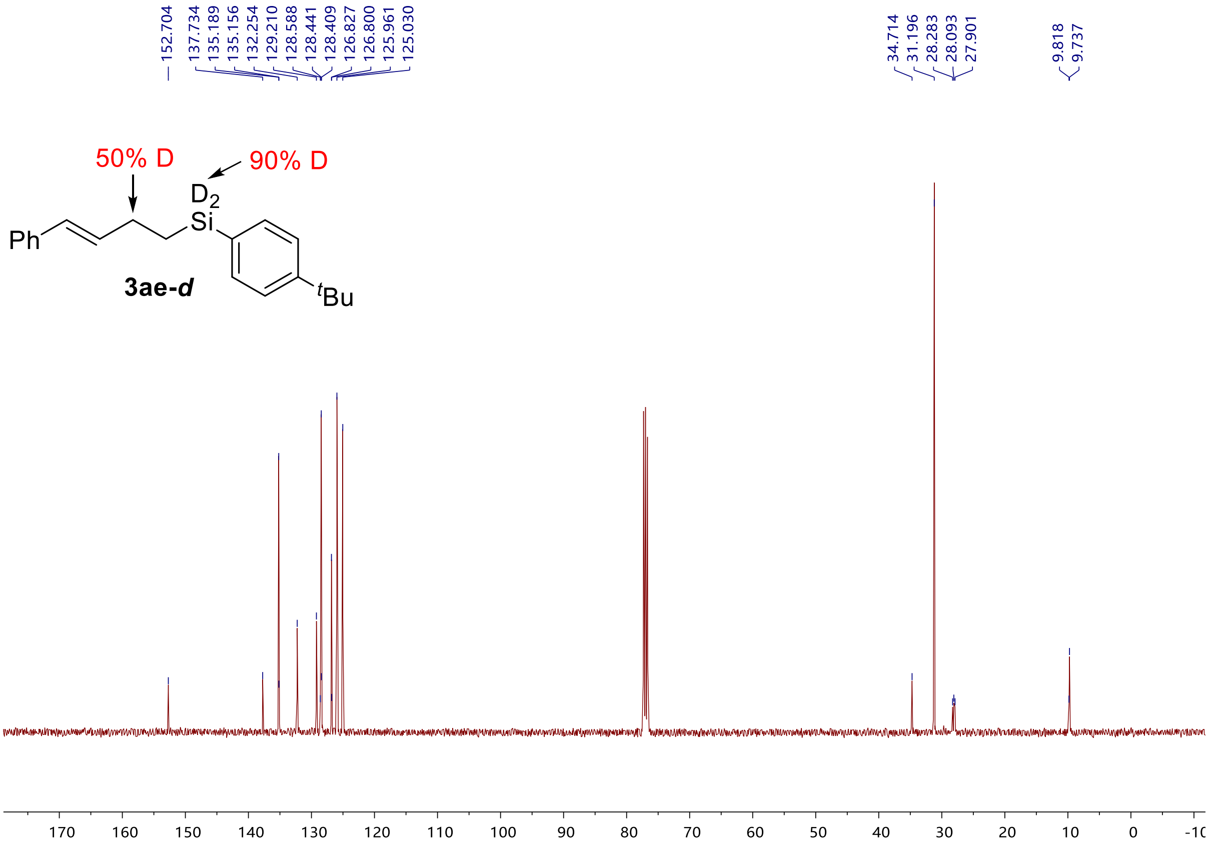

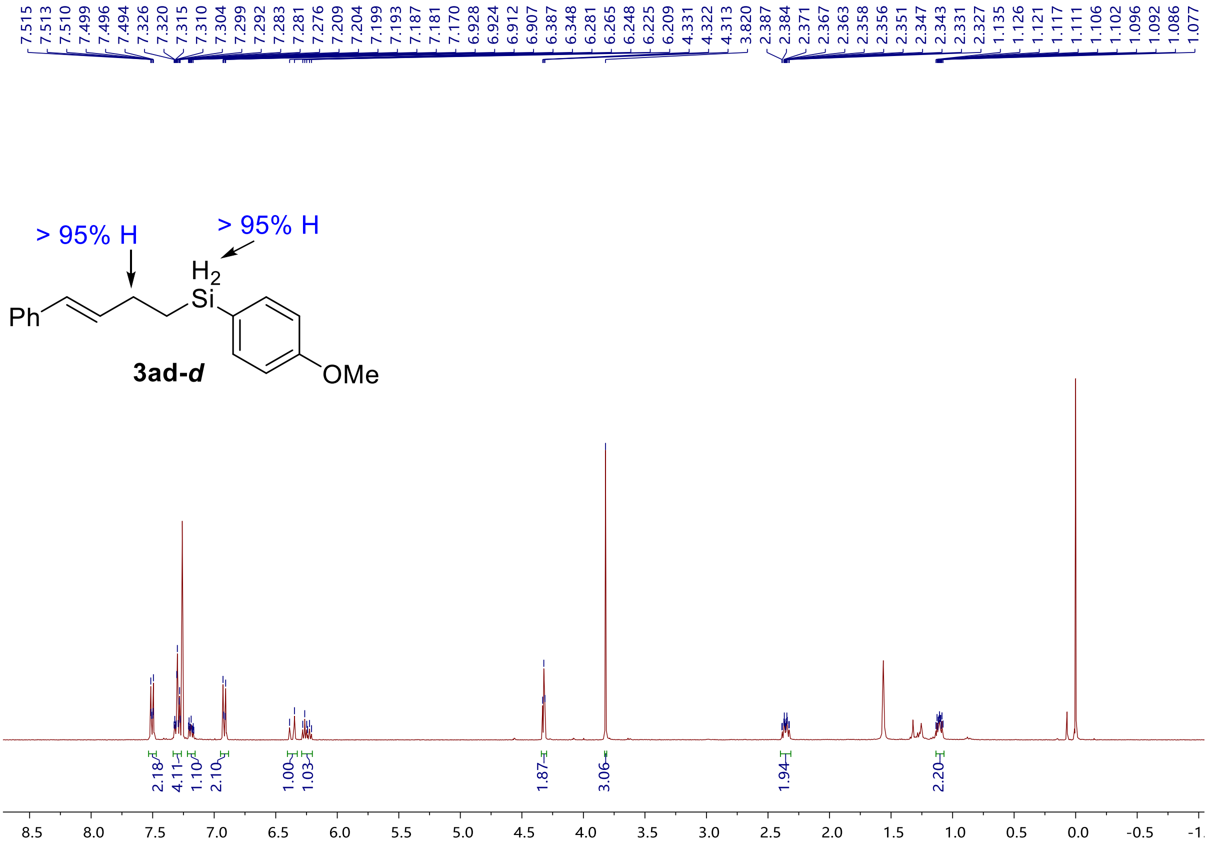

7.515
7.513
7.510
7.499
7.496
7.494
7.326
7.320
7.315
7.310
7.304
7.299
7.292
7.283
7.281
7.276
7.209
7.204
7.199
7.193
7.187
7.181
7.170
6.928
6.924
6.912
6.907
6.387
6.348
6.281
6.265
6.248
6.225
6.209
4.331
4.322
4.313
3.820
2.387
2.384
2.371
2.367
2.363
2.358
2.356
2.351
2.347
2.343
2.331
2.327
1.135
1.126
1.121
1.117
1.111
1.106
1.102
1.096
1.092
1.086
1.077
> 95% H
> 95% H
H2
Si
Ph
OMe
3ad-d
2.18
4.11
1.10
2.10
1.00
1.03
1.87
3.06
1.94
2.20
8.5
8.0
7.5
7.0
6.5
6.0
5.5
5.0
4.5
4.0
3.5
3.0
2.5
2.0
1.5
1.0
0.5
0.0
-0.5

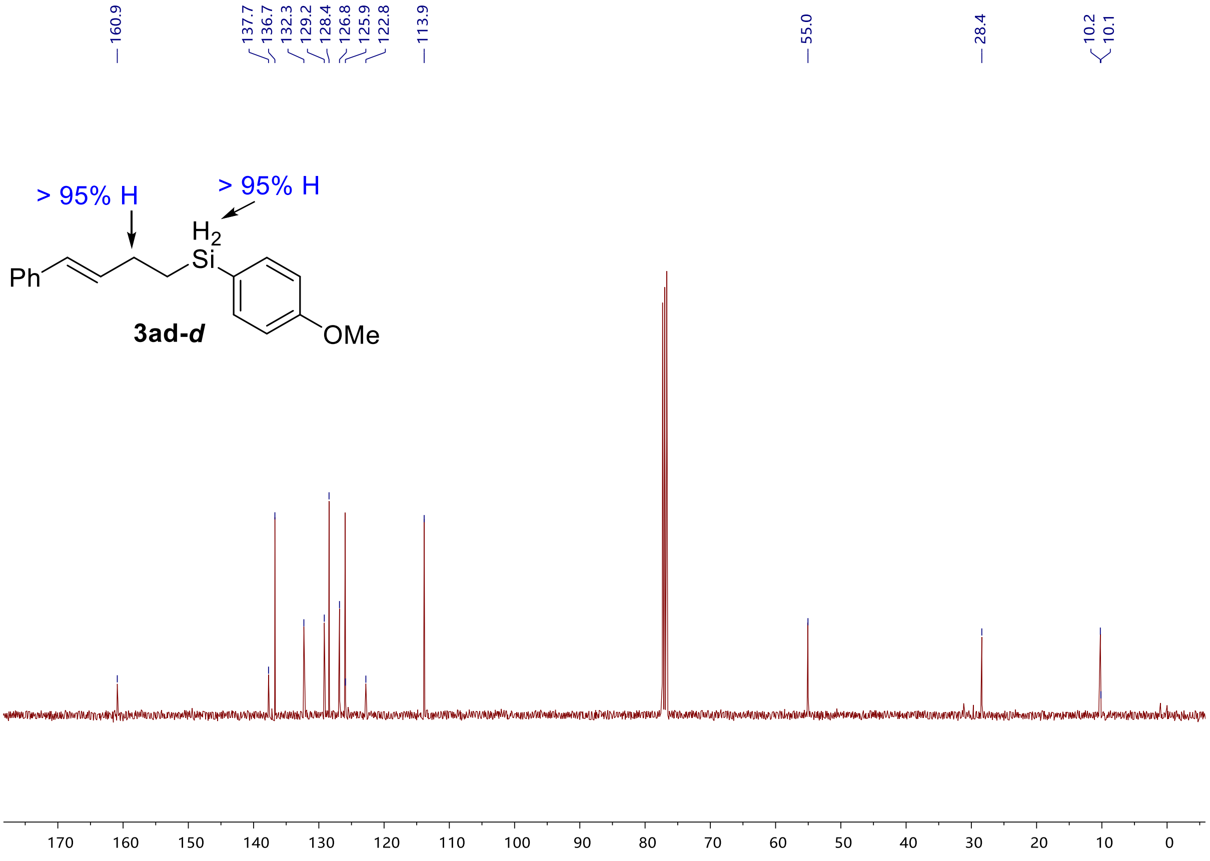

160.9
137.7
136.7
132.3
129.2
128.4
126.8
125.9
122.8
113.9
55.0
28.4
10.2
10.1
> 95% H
> 95% H
H2
Si
Ph
OMe
3ad-d
170
160
150
140
130
120
110
100
90
80
70
60
50
40
30
20
10
0

9. References

(1) R. J. Maza, E. Davenport, N. Miralles, J. J. Carbo and E. Fernandez, *Org. Lett.,* 2019, **21**, 2251.

(2) N. T. Patil and V. Singh, *Chem. Commun.,* 2011*,* **47***,* 11116.

(3) M.-Y. Hu, Q. He, S.-J. Fan, Z.-C. Wang, L.-Y. Liu, Y.-J. Mu, Q. Peng and S.-F. Zhu, *Nat. Commun.,* 2018, **9**, 221.

(4) T.-Q. Wang, Y.-Y. Hu and S.-L. Zhang, *Org. Biomol. Chem.,* 2010, **8**, 2312.

(5) K. Clinch, C. J. Marquez, M. J. Parrott and R. Ramage, *Tetrahedron,* 1989, **45**, 239.

(6) L. T. Kliman, S. N. Mlynarski, G. E. Ferris and J. P. Morken, *Angew. Chem. Int. Ed.* 2012, **51**, 521.

(7) L.-H. Liao, R.-Z. Guo and X.-D. Zhao, *Angew. Chem. Int. Ed.,* 2017, **56**, 3201.

(8) W. Herz and R. R. Juo, *J. Org. Chem.,* 1985, **50**, 618.

(9) A. M. Schmidt and P. Eilbracht, *J. Org. Chem.,* 2005, **70**, 5528.

(10) M. D. Visco, J. M. Wieting and A. E. Mattson, *Org. Lett.,* 2016, **18**, 2883.

(11) M. Kuritani, S. Tashiro and M. Shionoya, *Inorg. Chem.,* 2012, **51**, 1508.

(12) M. D. Greenhalgh, D. J. Frank and S. P. Thomas, *Adv. Synth. Catal.,* 2014, **356**, 584.

(13) B. Raya, S. Jing, V. Balasanthiran and T. V. RajanBabu, *ACS Catal.,* 2017, **7**, 2275.

(14) D.-J. Peng, Y.-L. Zhang, X.-Y. Du, L. Zhang, X.-B. Leng, M. D. Walter and Z. Huang, *J. Am. Chem. Soc.,* 2013, **135**, 19154.

(15) A. J. Challinor, M. Calin, G. S. Nichol, N. B. Carter and S. P. Thomas, *Adv. Synth. Catal.,* 2016, **358**, 2404.

(16) C. Chen, M. B. Hecht, A. Kavara, W. W. Brennessel, B. Q. Mercado, D. J. Weix and P. L. Holland, *J. Am. Chem. Soc*., 2015, **137**, 13244.

(17) P.-F. Fu, *J. Mol. Catal. A-Chem.,* 2006, **243**, 253.

(18) C.-Z. Wu, W. J. Teo and S.-Z. Ge, *ACS Catal.,* 2018, **8**, 5896.

(19) M.-Y. Hu, P. He, T.-Z. Qiao, W. Sun, W.-T. Li, J. Lian, J.-H. Li and S.-F. Zhu, *J. Am. Chem. Soc.,* 2020, **142**, 16894.

(20) R. Y. Kong and M. R. Crimmin, *J. Am. Chem. Soc.,* 2020, **142**, 11967.
